# Crosstalk with lung fibroblasts shapes the growth and therapeutic response of mesothelioma cells

**DOI:** 10.1038/s41419-023-06240-x

**Published:** 2023-11-08

**Authors:** Yakinthi Chrisochoidou, Rajat Roy, Pooyeh Farahmand, Guadalupe Gonzalez, Jennifer Doig, Lukas Krasny, Ella F. Rimmer, Anne E Willis, Marion MacFarlane, Paul H. Huang, Neil O. Carragher, Alison F. Munro, Daniel J. Murphy, Kirill Veselkov, Michael J. Seckl, Miriam F. Moffatt, William O. C. Cookson, Olivier E. Pardo

**Affiliations:** 1 Division of Cancer, Imperial College, Du Cane Road, London, W12 0NN UK; 2 Institute of Cancer Sciences, University of Glasgow, Glasgow, G12 8QQ UK; 3 Department of Computing, Faculty of Engineering, Imperial College London, London, SW7 2AZ UK; 4 Molecular and Systems Oncology, The Institute of Cancer Research, Sutton, SM2 5NG UK; 5 MRC Toxicology Unit, Tennis Ct Rd, Cambridge, CB2 1QR UK; 6 Cancer Research UK Scotland Centre, Institute of Genetics and Cancer, University of Edinburgh, Edinburgh, EH4 2XR UK; 7 Department of Environmental Health Sciences, Yale School of Public Health, New Haven, CT USA; 8 National Heart and Lung Institute, Imperial College, Dovehouse St, London, SW3 6LY UK

**Keywords:** Cancer microenvironment, Mechanisms of disease

## Abstract

Mesothelioma is an aggressive cancer of the mesothelial layer associated with an extensive fibrotic response. The latter is in large part mediated by cancer-associated fibroblasts which mediate tumour progression and poor prognosis. However, understanding of the crosstalk between cancer cells and fibroblasts in this disease is mostly lacking. Here, using co-cultures of patient-derived mesothelioma cell lines and lung fibroblasts, we demonstrate that fibroblast activation is a self-propagated process producing a fibrotic extracellular matrix (ECM) and triggering drug resistance in mesothelioma cells. Following characterisation of mesothelioma cells/fibroblasts signalling crosstalk, we identify several FDA-approved targeted therapies as far more potent than standard-of-care Cisplatin/Pemetrexed in ECM-embedded co-culture spheroid models. In particular, the SRC family kinase inhibitor, Saracatinib, extends overall survival well beyond standard-of-care in a mesothelioma genetically-engineered mouse model. In short, we lay the foundation for the rational design of novel therapeutic strategies targeting mesothelioma/fibroblast communication for the treatment of mesothelioma patients.

## Introduction

Mesothelioma is a rare but highly aggressive cancer arising from the neoplastic transformation of the mesothelial tissue monolayer lining body cavities. While mesothelioma can develop from any serosal surface, ~70% of all cases are of pleural origin (MPM). Approximately 80% of all cases are attributed to exposure to a group of naturally occurring minerals, collectively referred to as asbestos [1]. While the new use of asbestos is banned in most industrialised countries, the incidence and mortality of MPM continue to increase worldwide because of the long latency of the disease (commonly 40 years), persistence of asbestos in many buildings, and continued use of asbestos in a large number of developing countries.

Unresectable MPM is treated with combinations of cisplatin and pemetrexed. Innate drug resistance results in poor responses and overall patient survival ranging from 6 to 21 months, a prognosis that remains unchanged since 1982. Identification of novel therapeutic regimens are urgently needed to improve these outcomes. However, the relatively low mutational burden of this disease is largely defined by loss-of-function in tumour suppressors rather than gain-of-function in classic driver oncogenes [2], so mesothelioma has not benefitted from the use of targeted therapies that has revolutionised management of other lung malignancies [3].

MPM tumourigenesis is driven by the chronic inflammatory response initially generated to contain pro-inflammatory asbestos fibres and tumours are accompanied by an extensive fibrosis that is a major driver of pain and respiratory insufficiency.

In several other cancers, fibrosis drives changes in the stiffness of the extracellular matrix (ECM) that enhances tumour growth, survival and dissemination while limiting accessibility to therapeutic intervention [4]. In mesothelioma many cell types participate in ECM remodelling and fibrosis in the tumour microenvironment (TME), but cancer-associated fibroblasts (CAFs) play a central role and are associated with tumour progression and prognosis [5–7].

In addition to driving ECM remodelling, CAFs regulate the biology of tumour cells. This occurs through both cell-cell contact and the secretion of numerous factors that promote tumour growth directly or indirectly through modulating other cell-types in the TME, including T-lymphocytes [8]. Hence, they have attracted attention as potential therapeutic targets to hinder tumour progression [8].

Despite the clear involvement of CAFs in the pathogenesis of MPM, there has only been limited attempts at studying in detail the crosstalk between mesothelioma cells and lung fibroblasts [9] and the therapeutic possibilities that this may yield [7].

Here, using several proteomic approaches and co-cultures between eight patient-derived mesothelioma cell lines and lung fibroblasts, we characterise changes in cytokine/chemokines secretions and ECM deposition by fibroblasts upon activation as well as signalling crosstalk between the two cell types. This approach reveals recurring targetable molecular changes associated with MPM/Activated Fibroblast (AF) interaction and proposes novel drug combinations that far exceed the efficacy of current first-line treatment in vitro. Finally, we prioritised compounds to be tested in vivo using AI-based network propagation and show that single-agent SRC inhibition with saracatinib extend survival of our mesothelioma genetically-engineered mouse model well beyond the cisplatin/pemetrexed combination.

## Results

### Mesothelioma cells show a profibrotic expression profile for cytokine, chemokines and acute phase proteins

To maximise the relevance of our data to disease, we used patient-derived cell lines that were previously shown to better represent MPM tumours than currently available commercial counterparts [10]. Seven such cell lines (Meso 8, 12, 23, 27, 33, 60 and 70) were selected and their characteristics and phenotypes compared to that of the immortalised pleural cell line Met-5A. The principal mutational patterns of the patient-derived cell lines were previously published by us and cover the range of genetic lesions characteristic of MPM [2] and is summarised in Supplementary Fig. 1A.

The MPM cell lines all proliferated in 2D culture at a rate similar to that of Met-5A cells, with the notable exception of Meso 33 which showed increased proliferative capabilities (Supplementary Fig. 1B). This may reflect the biphasic histology of this cell line which is associated with a more aggressive progression of the disease compared to the epithelioid subtype.

All MPM cell lines, as well as Met-5A cells, were also able to grow as 3D spheroids, although differences in growth rate were more pronounced in this format (Supplementary Fig. 1C). Meso 33 cells again showed a significant growth advantage at 72 h as compared to other cell lines (Supplementary Fig. 1C).

**Fig. 1:**
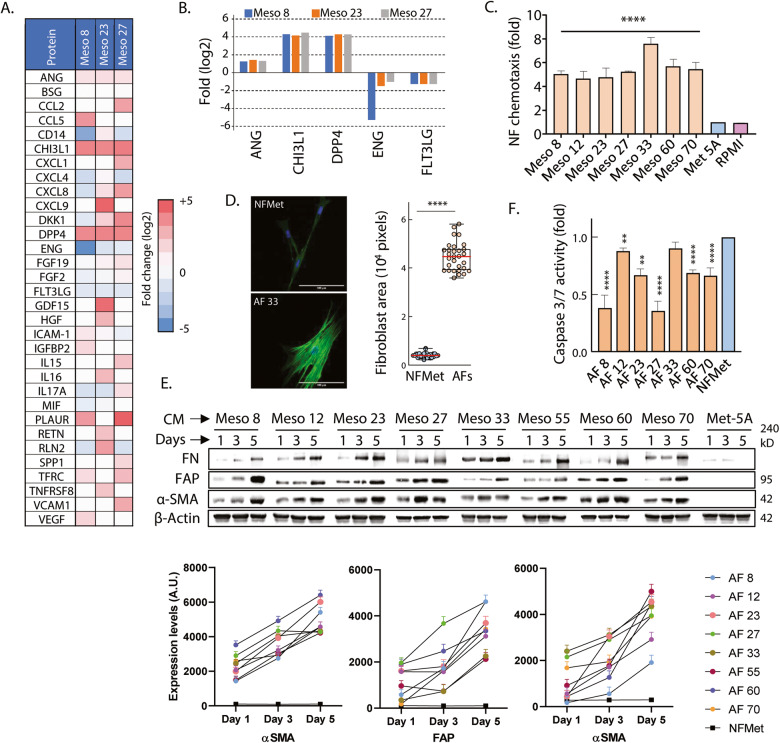
MPM cells secretion chemoattract and activate lung fibroblasts. **A** Cytokine,- chemokine and acute phase proteins profiling was performed on three patient-derived MPM cell lines and log2 fold changes in expression over mesothelial cells (Met-5A) represented as a heatmap. **B** Consistent log 2-fold changes in 5 cytokines from (A) were represented as bar graphs. Data shown are from a representative repeat. **C** MRC-5 fibroblasts were placed in the upper part of a Boyden chamber with conditioned media from the indicated MPM cell lines placed in the bottom chambers. Data shown are the mean ± SD of 3 biological replicates normalised to results obtained with CM from Met-5A cells. Two-way ANOVA with Dunnett’s multiple comparison tests were performed to assess statistical significance (****P < 0.001). **D** MRC-5 fibroblasts incubated with CM from individual MPM cell lines (ie AF 33 are NFs treated with CM from Meso 33) or Met-5A cells (NFMet) for 72 h were stained with Alexa488-Phalloidin (green) and Hoechst (blue) prior to confocal microscopy. Left panel: exemplar image. Scale bar: 100μm. Right panel: Cell area in pixels was calculated for *N* = 30 cells using Image J and plotted as a Boxplot. Red line represent median. Statistics: Unpaired t-test (****P < 0.001). **E** MRC-5 cells were seeded in CMs from the indicated MPM cell lines (Meso) or Met-5A cells for the indicated time. Cell lysates were analysed by Western Blotting for the indicated proteins. Upper panel: Data shown are representative of 4 biological replicates. Lower panel: Optical densitometry quantification of 4 biological replicates. **F** MRC-5 cells seeded for 72 h in CMs from indicated cell lines were subjected to a Caspase 3/7 substrate-based activity assay in 3D cultures. Data are mean ± SEM from 3 biological repeats. Statistics: Two-way ANOVA with multiple comparisons (*P < 0.05, **P < 0.01, ****P < 0.001).

To assess the profibrotic potential of secretions from MPM cells, we first profiled the cytokines, chemokines and acute phase proteins produced by three MPM cell lines that showed contrasting 3D growth profiles (Meso 8, 23 and 27) as well as Met5A cells. The levels of 68 molecules were measured and 7 of these showed increased expression in at least two MPM cell lines as compared to Met-5A cells (Fig. 1A). In particular, the ECM remodelling molecule chitinase 3 Like 1 (CHI3L1), angiogenin (ANG) and dipeptidyl peptidase 4 (DPPIV) were increased in all three MPM cell lines (Fig. 1B).

In contrast, two molecules were downregulated in all MPM cell lines; the TGFβ co-receptor endoglin (ENG) and regulator of dendritic cells differentiation FLT3LG (Fig. 1B). Interestingly, all three upregulated molecules have previously been implicated in fibrosis [11–13] and chemotaxis of various cell types including fibroblasts [14–17]. In contrast, ENG has been previously shown to inhibit fibroblast recruitment through its action on the TGFβ pathway [18] and its decreased expression here may indicate a de-repression of this brake. Hence, this proteomic profile suggested that our MPM cells lines may chemoattract fibroblasts during initiation of the fibrotic response.

### Secretions from MPM cells chemoattract and activate lung fibroblasts

This hypothesis was tested using MRC-5 human lung fibroblasts, which were previously used to study lung fibrosis [19] and the onset of cancer-associated fibroblasts in several cancers including mesothelioma [7, 20–22]. Boyden chamber assays confirmed that conditioned media from all seven MPM cell lines were capable of chemoattracting MRC-5 human normal lung fibroblasts within 24 h (Fig. 1C, Supplementary Fig. 2A). This was associated with phenotypic changes in the fibroblasts that included increase in cell area, stellate appearance and formation of actin stress fibres (Fig. 1D, Supplementary Fig. 2B). These changes have previously been associated with fibroblast activation [23], and so we investigated if fibroblasts exposed to the condition media from our MPM cells expressed recognised markers of fibroblast activation. Indeed, we found that within days of exposure, MRC-5 fibroblasts displayed increased expression of Fibroblast activation protein (FAP), alpha-smooth muscle actin (αSMA) and the profibrotic protein fibronectin (FN) (Fig. 1E, Supplementary Fig. 2C), all recognised markers of activation [23]. In comparison, no such change occurred following exposure of fibroblasts to the conditioned medium from Met-5A cells (Fig. 1E), showing that these changes were specifically induced by cancer cells. These activated fibroblasts will thereafter be referred to as Activated Fibroblasts (AFs) followed by the number of their activating mesothelioma cell line.

Similar changes were observed in fibroblasts exposed to conditioned media from the commercial MPM cell lines, despite our data demonstrating that they poorly represented clinical disease [10] (Supplementary Fig. 2E), suggesting that fibroblast activation is a generalisable outcome of fibroblast/MPM cell interaction. Moreover, as previously reported [24, 25], the activated phenotype was irreversible, as increased expression of FN, FAP and αSMA was still observed following 11 subsequent passages in normal culture medium devoid of secretions from MPM cells (Supplementary Fig. 2F).

### Fibroblast activation is associated with increased survival of both AFs and MPM cells

As mesothelioma is accompanied by a large accumulation of CAFs in the tumour microenvironment, we next investigated whether MPM AFs showed increased proliferation. However, 2D growth assays revealed that AFs proliferated at a similar rate to MRC-5 cells or MRC-5 cells exposed to conditioned medium from Met-5A cells (NFMet) (Supplementary Fig. 3A) and this was associated with an overall unchanged cell cycle profile (Supplementary Fig. 3B). This lack of increased proliferation in AFs as compared to naive or Met-5A-exposed fibroblasts was also observed in 3D culture (Supplementary Fig. 3C). This suggested that increased proliferation may not be at the source of fibroblasts accumulation in MPM. Therefore, we next investigated whether AFs showed markers of improved survival. This revealed that all but one of our AF cell lines (AF activated by Meso 33, aka AF 33) showed decreased baseline caspase 3 and 7 activity, suggesting improved survival abilities (Fig. 1F). This was accompanied by increased expression of the antiapoptotic proteins XIAP and/or BCL2 in AFs together with increased inactivation of proapoptotic BAD through phosphorylation (Supplementary Fig. 3D). Hence, increased survival may contribute to the accumulation of CAFs at the tumour site.

### Cytokine and growth factor profiling of AFs suggest an inflammatory, profibrotic and chemotactic phenotype

**Fig. 2:**
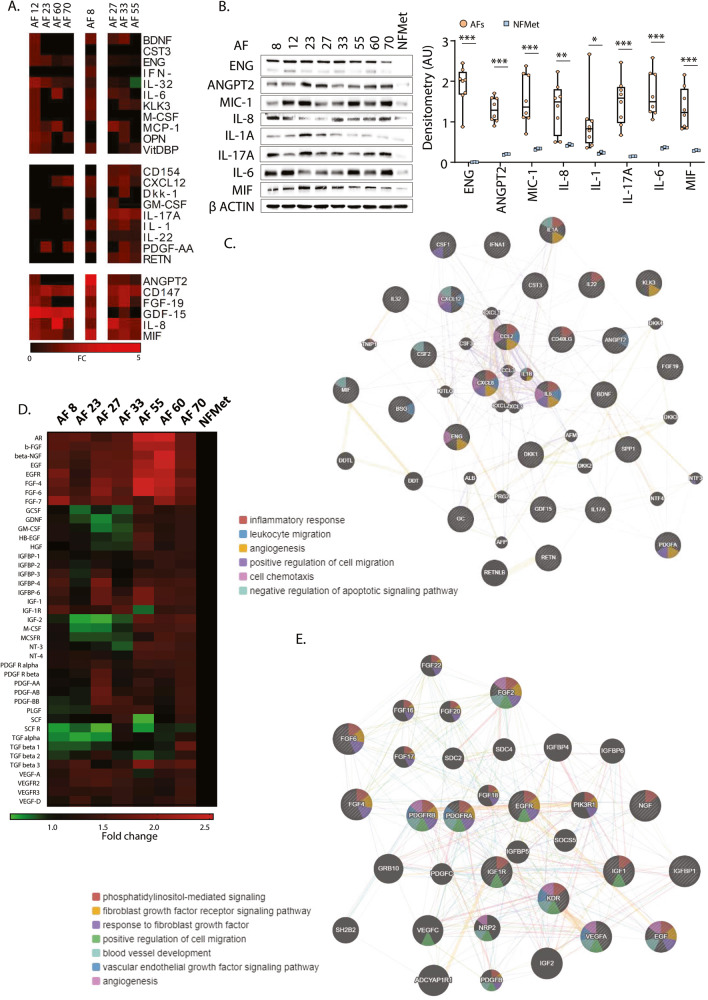
AFs show a pro-inflammatory and chemotactic secretion profile. **A** Cytokine,- chemokine and acute phase proteins profiling was performed on AFs obtained from incubation of MRC-5 fibroblasts with the CMs of the indicated MPM cell lines or Met-5A cells. Heatmap represent fold changes over treatment with Met-5A CM and K-mean clustering was performed on both proteome and CM conditions using ClusterMaker under Cytoscape. **B** Lysates from AFs and MRC-5 cells incubated with CM from Met-5A cells (NFMet) were analysed by SDS-PAGE/western blotting for the indicated proteins. Detection of βActin was used as a loading control. Left panel: representative blots. Right panel: Optical densitometry quantification of 4 biological replicates. Each dot represents an individual AF cell line. Statistics: Student *t*-test (*P < 0.05, **P < 0.01, ***P < 0.005). **C** Proteome profiling results from (**A**) were analysed by DAVID (https://david.ncifcrf.gov/) for functional interaction network and gene ontology. **D** Heat map of growth factor profiling assays performed on AFs obtained from incubation of NFs with MPM cell lines or Met-5A cells (NFMet). **E** Growth factors profiling results from (**D**) were analysed by DAVID (https://david.ncifcrf.gov/) for functional interaction network and gene ontology.

In order to better understand the effect that MPM CAFs may have on the tumour microenvironment, we compared the cytokine/chemokine/acute phase proteins and growth factor profiles of our AFs with that of MRC-5 cells exposed to condition medium from Met-5A cells (NFMet). This revealed an increased expression in MPM AFs of a mixture of pro-inflammatory molecules, such as IL1, IL6 and CXCL12, characteristic of pro-tumorigenic inflammatory CAFs (iCAF) as opposed to myofibroblastic CAFs (myCAFs) [26, 27] (Fig. 2A–C). In addition, AFs showed decreased expression of TGFβ, but increased levels of the TGFβ signalling inhibitor ENG and of platelet derived growth factor receptors (PDGFRs), all changes reported to be inhibitory of myCAF differentiation (Fig. 2A, B, D).

The pro-tumorigenic activity of iCAFs is in part mediated through the secretion of growth factors and cytokines that increase the viability of tumour cells and promote tumour progression. In agreement with these functions, our MPM AFs showed increased expression of IL-6, IL-17A, PDGFs, various FGFs and IGF-1 (Fig. 2A, B, D), all molecules reported to improve the survival and chemoresistance of tumour cells through their cross-talk with CAFs [28].

Also increased was CXCL-12, which was shown to promote tumour progression through various mechanisms including metastasis and chemoresistance [29]. In addition, gene ontology analysis of functional networks built from cytokines and growth factors overexpressed in our AFs revealed overrepresentation of molecules associated with positive regulation of cell migration, negative regulation of apoptosis and angiogenesis, all processes required for the tumorigenic process (Fig. 2D, E).

Finally, the importance of using disease-representative MPM cell lines was underlined by the lack of overlap in the cytokines and growth factors profiles of fibroblast activated by our cells versus commercial MPM cell lines (NCI-H2052 and MSTO-211H) (Supplementary Fig. 3E).

### MPM AFs chemoattract and activate naive fibroblast

**Fig. 3:**
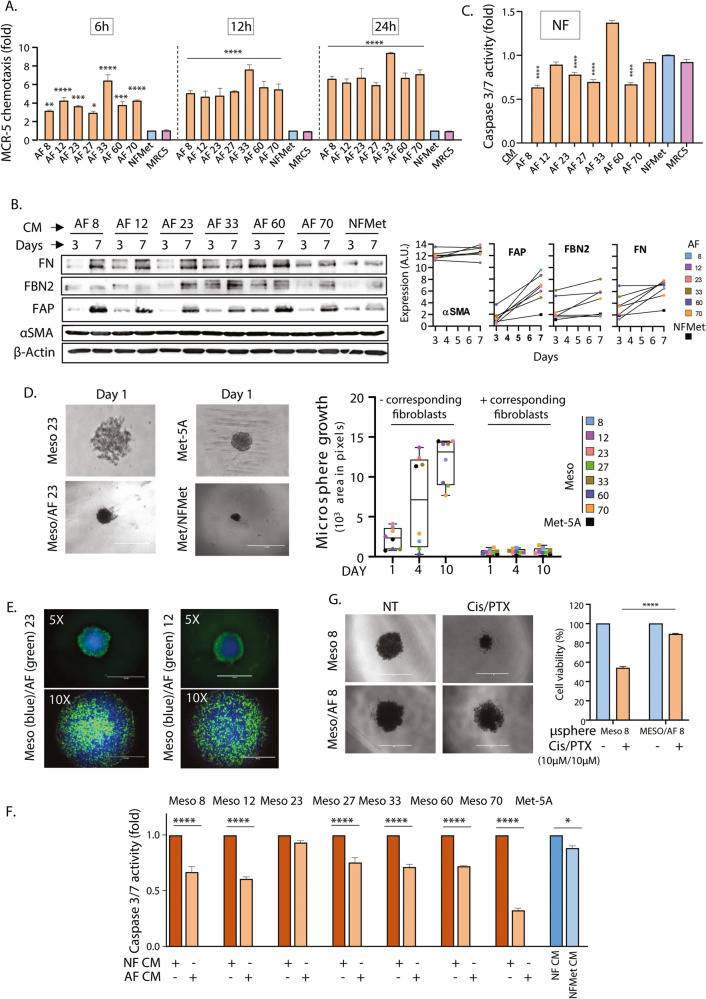
AFs chemoattract and activate NF and promote MPM cell chemoresistance. **A** MRC-5 cells were placed in the upper part of a Boyden chamber with condition media from the indicated AFs, NFMet or MRC-5 cultures placed in the bottom chambers for the indicated time. Data shown are the mean ± SD of 3 biological replicates normalised to results obtained with NFMet CM treatment. Two-way ANOVA with Dunnett’s multiple comparison tests were performed to assess statistical significance (*P < 0.05, **P < 0.01, ***P < 0.005, ****P < 0.001). **B** MRC-5 cells were seeded in CM from the indicated AFs or NFMet for the indicated time. Cell lysates were analysed by western blotting for the indicated proteins. Left panel: Data shown are representative of 4 biological replicates. Right panel: Optical densitometry quantification of 4 biological replicates. **C** MRC-5 cells seeded for 72 h in CM from indicated AFs, NFMet or MRC-5 cells were subjected to a Caspase 3/7 substrate-based activity assay in 3D cultures. Data are mean ± SEM from 3 biological repeats. **D** Comparison of growth of 3D spheroids composed of either MPM or Met-5A cells alone or in co-culture with the corresponding AFs or NFMet, respectively. Left: Representative brightfield pictures acquired at Day 1 of the assay (24 h post seeding). Scale bar: 1 mm. Right: ImageJ-based quantification of the size of coculture microspheres at the indicated timepoints. **E** Representative fluorescence microscopy images of 3D cocultures of differentially-labelled MPM and corresponding AF cell lines. Scale bar: 1 mm. **F** MPM cells were seeded for 72 h in CM from the corresponding AFs, NFMet or MRC-5 cells and subjected to a Caspase 3/7 substrate-based activity assay in 3D cultures. Data are mean ± SEM from 3 biological repeats. **G** 3D spheroids made of either Meso 8 cells alone or cocultures of Meso 8 and AF 8 were treated with cisplatin/pemetrexed or diluent (NT). Left panel: representative images of *n* = 3. Scale bar: 1 mm. Right panel: Cell viability was determined using the Cell TiterGlo 3D assay. Data are mean ± SEM of *n* = 4. Statistics: Two-way ANOVA with multiple comparisons (*P < 0.05, **P < 0.01, ****P < 0.001).

Amongst the cytokine and growth factors overexpressed by our AFs (Fig. 2A, B, D), several, including MCP-1, ANGPT2, PDGF, MIF, CXCL12 and IL17A, have been reported to trigger fibroblasts chemotaxis and activation as well as fibrosis [13, 30–34]. Hence, we next investigated whether our AFs could themselves recruit and activate additional fibroblasts. Boyden chamber assays demonstrated that conditioned media from our AFs was able to chemoattract MRC-5 fibroblasts (Fig. 3A). This process was faster than the chemoattraction of fibroblasts by MPM cells, with significant fibroblast recruitment already observed at the 6 h timepoint (Fig. 3A, Supplementary Fig. 2A). This was accompanied by fibroblast activation as demonstrated by increased expression of FAP and the fibrosis markers Fibrillin 2 (FBN2) and FN (Fig. 3B). As for fibroblast activation downstream of MPM cells, activation using conditioned media from AF led to a decrease in the baseline level of caspase 3/7 activity in recipient fibroblasts, suggesting inhibition of apoptotic processes (Fig. 3C).

Taken together, our data demonstrate that fibroblast activation downstream of MPM cells is self-propagating and that fibroblast accumulation in MPM is likely the result of continued chemotaxis of naive fibroblasts and improved survival of CAFs.

### AF/MPM crosstalk results in increased survival and drug resistance of MPM cells

Several cytokines and growth factors expressed by our AFs, such as CXCL-12, IL6, FGFs and VEGFs, were shown to participate in CAFs/cancer cells crosstalk, and promote tumour progression [24, 32, 35]. We therefore investigated the biological effect of interaction between our AFs and their corresponding MPM cell lines.

Using 3D co-cultures, we first assessed whether the presence of AFs impacted the growth rate of MPM cells. First, we noticed that the microspheres established in the presence of fibroblasts were more compact than those formed by MPM or Met-5A cells alone (Fig. 3D-left), suggesting that fibroblasts may change the dynamic of aggregation resulting in more condensed spheroids. Second, we found that the presence of fibroblasts decreased the growth of MPM and Met-5A cells as compared to spheroids made of MPM or Met-5A cells alone (Fig. 3D-right). The growth restraining effect of CAFs has been reported previously to accompany the early stages of tumorigenesis [36], and may be due to the positioning of the fibroblasts on the outside of the MPM/AF spheroids as we demonstrated by microscopic observation using differentially-labelled cell types in co-cultures (Fig. 3E).

However, conditioned media from AFs improved the survival of the corresponding MPM cell lines, as demonstrated by decreased baseline caspase 3/7 activity in these cells (Fig. 3F). This was associated with drug resistance of the resulting co-cultures, with the efficiency of the cisplatin/pemetrexed combination being significantly (*p* < 0.0001) decreased in AF/MPM co-cultures as compared to spheroids made on MPM cells alone (Fig. 3G). As this drug combination is the standard-of-care for MPM, these results suggest that the presence of CAFs is detrimental to the therapeutic response of patients.

### MPM CAFs produce a profibrotic extra-cellular matrix

**Fig. 4:**
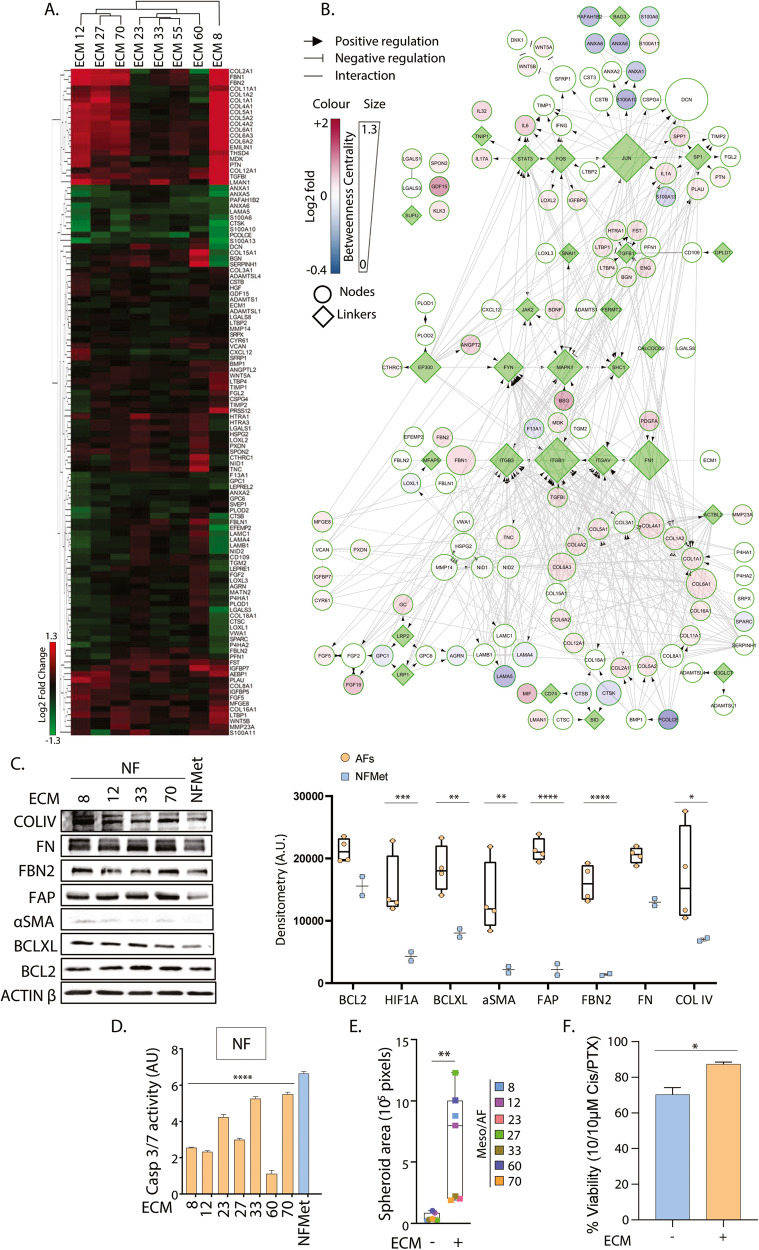
ECM from AFs is capable of activating fibroblasts and promoting growth and viability of MPM/AF cocultures. **A** ECMs produced from the corresponding AF cell lines (see numbers) or NFMet were subjected to quantitative proteomic analysis by mass-spectrometry. Heat map for the log2 fold changes in expression over ECM from NFMet were analysed by hierarchical clustering using ClusterMaker under Cytoscape. **B** A functional Interaction network based on the combined cytokine/growth factor profiling and ECM mass spectrometry results was built under Cytoscape using the Reactome FI plugin. Continuous colour mapping was used to represent log2 fold changes in expression while node size represent Betweeness centrality measurements as indicated in the legend. **C** Lysates from MRC-5 cells cultured over either AF cell lines (see numbers) or NFMet-derived ECM for 72 h were analysed by SDS-PAGE/Western Blotting for the expression of the indicated proteins. Data representative of 3 independent biological repeats. **D** The activity of caspases 3/7 in spheroids of MRC-5 fibroblasts embedded in ECM derived from either AF cell lines (see numbers) or NFMet cells was assessed using a substrate-based assay. Data are the mean ± SEM from 3 independent biological replicates. Statistics: Two-way ANOVA with multiple comparisons (****P < 0.001). **E** MPM/AF coculture spheroids grown in the presence or absence of ECM derived from the corresponding AF cell line for 72 h were imaged in brightfield (see Supplementary Fig. 4C) and their size determined using the FIJI ImageJ software. Each dot is the mean of *n* = 3 biological repeats for a particular coculture. Statistics: Student t-test (**P < 0.01). **F** MPM/AF spheroids were incubated in the presence or absence of ECM produced by the corresponding AFs and cell viability tested 72 h following treatment with cisplatin/pemetrexed (Cis/PTX). Data shown represent the average response ± SEM of biological triplicates for Meso 23, 70 and 8 cell lines and associated AFs. Statistics: Student t-test (*P < 0.05, **P < 0.01, ***P < 0.005, ****P < 0.001).

In order to understand the role of CAFs in shaping the MPM tumour microenvironment, we next analysed the composition of the extra-cellular matrix (ECM) produced by our AFs. Following decellularization, the ECM deposited by our AFs was analysed by mass-spectrometry (MS) and its composition compared to that produced by MRC-5 fibroblasts incubated with condition medium from Met-5A cells (Fig. 4A and Supplementary Excel file 1). This revealed an overall increased deposition by AFs of collagen family members (COLs, including COL4A1 and COL11A1), fibrillins (FBN1 and 2) and tenascin (TNC) (Fig. 4B), previously involved in lung fibrosis [37, 38] and deposited by CAFs in other cancers [39, 40].

In addition, decreased levels of some laminins (LAMAs) were also observed, and prior literature suggests that this may be aggravating pulmonary fibrosis [41]. In agreement with our previous data in AFs which proposed a decrease in signalling through TGFβ (Fig. 2A, B, D), several inhibitors of this molecule showed increased expression in the ECM from CAFs, including EMILIN1 [42], HTRA1 [43], FST [44], ENG [18], as well as two TGFβ binders LTBP1 and 4 [45, 46] (Fig. 4B), suggesting sequestration of latent TGFβ.

This hypothesis was confirmed by gene ontology analysis (GOA), with “Sequestering of TGFbeta in extracellular matrix” being one of the most significant biological processes (BPs) associated with our differentially expressed ECM constituents (Supplementary Fig. 4A, B). In addition, GOA highlighted that BPs associated with positive regulation of cell-substrate adhesion, angiogenesis, cell migration and cell division were over-represented amongst our overexpressed matrix constituents.

### The ECM from MPM AFs is capable of activating naive fibroblasts

We have already shown above that AFs are able to chemoattract and activate naive fibroblasts through secreted factors. Because our ECM appeared to trap large numbers of inducers of fibroblast activation such as IL1α [47], IL6 and IL17A we investigated the possibility that this matrix may in itself be capable of activating naive fibroblasts.

Indeed, fibroblasts exposed to the AF-derived ECM demonstrated an activated profibrotic phenotype, with increased expression of FAP, αSMA, collagen IV (COLIV) and FN as compared to fibroblasts treated with ECM from Met-5A-exposed MRC-5 fibroblasts (Fig. 4C). In addition, they displayed higher expression of the antiapoptotic molecules BCL2 and BCL-X_L_ (Fig. 4C) which was associated with decreased baseline caspase 3/7 activity (Fig. 4D), suggesting improved survival abilities.

### The ECM from MPM AFs promotes growth and drug resistance of MPM/AF spheroids

We then tested how the AF ECM impacts on the growth and therapeutic response of MPM spheroids. These experiments showed that, unlike matrix produced by fibroblasts exposed to Met-5A cells, the ECM produced by MPM AFs promoted the growth of corresponding embedded MPM/AF spheroids (Fig. 4E and Supplementary Fig. 4C). This was in agreement with the results of our above gene ontology analysis (Supplementary Fig. 4A), which suggested positive regulation of cell growth and proliferation. The disseminated appearance of the cocultures is compatible with the suggested “positive regulation of cell migration”-related processes.

Finally, we assessed whether inclusion of the AF ECM could further impact the response of MPM cells to therapy. While the inclusion of AFs to the microsphere already reduced the drug response of MPM cells (Fig. 3G), we now show that addition of the corresponding AF ECM further decreases the efficacy of the cisplatin/pemetrexed combination in all MPM/AF co-cultures (Fig. 4F). Hence, the ECM produced by CAFs in MPM is likely to promote tumour progression.

### Therapeutically targetable signalling changes occur as the result of crosstalk between AFs and MPM cells

We have identified several cytokines and growth factors in the conditioned media of both AFs and MPM cells and shown that exposure to these affects the reciprocal survival of these cell types. We next performed experiments to highlight the signalling events that accompany these effects as these may reveal potential therapeutically-targetable events.

**Fig. 5:**
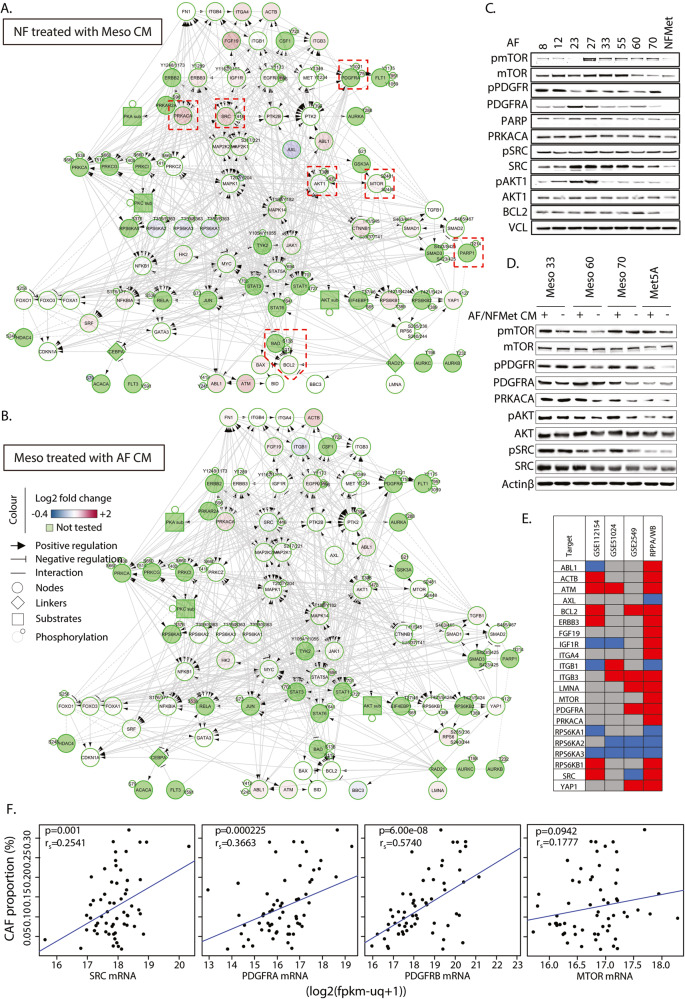
MPM/AF crosstalk results in the activation of a number of conserved signalling pathways. Lysates from MRC-5 cells incubated for 3 days with CM from MPM cells (**A**) or from MPM cells treated for 3 days with CM of the corresponding AFs (**B**) were analysed by RPPA. Log2 fold changes in protein expression or post-translational modification over NFMet (**A**) or MPM cells treated with CM from NFMet (**B**) were averaged over all cell lines and data imported into Cytoscape to build a functional interaction network using the Reactome FI plugin. Lysates from the indicated AFs or NFMet (**C**) or from MPM or Met-5A cells treated or not for 3 days with CM of the corresponding AFs or NFMet, respectively (**D**) were analysed by SDS-PAGE/western blotting for the expression of the indicated proteins. Data representative of 3 independent biological repeats. **E** Three publicly-available microarray datasets were analysed for changes in the mRNA expression of the indicated targets between normal mesothelial and mesothelioma tissue samples (see corresponding Supplementary Fig. 9). Results were summarised as a heatmap with red indicating statistically significant increase, blue statistically significant decrease and grey no change in expression. These results are here compared with those of our RPPA and Western blotting data (same colour code). **F** The expression of SRC, PDGFRA, PDGFRB and MTOR was extracted from the TCGA Mesothelioma GDC RNA-Seq dataset and correlated with the proportion of fibroblast infiltration deduced by cellular deconvolution of bulk transcriptional data using the Kassandra algorithm. The *p*-values obtained from linear regression models and the Spearman correlation coefficients are shown.

Hence, we treated fibroblasts with conditioned media from MPM cells and MPM cells with the conditioned media of the resulting AFs for 1 or 3 days and analysed the changes in expression and/or post-translational modification of various signalling molecules using reverse phase protein array (RPPA) (Supplementary Fig. 5). This revealed rapid changes in the expression and phosphorylation of many signalling molecules by 24 h of exposure (Supplementary Fig. 6A, B) which mostly persisted on the third day of incubation (Fig. 5A, B).

Common changes in both fibroblasts and MPM cells included increased expression of the catalytic subunit of protein kinase A (PRKACA), ABL1, beta-actin (ACTB), Ataxia Telangiectasia Mutated (ATM) and FGF19. We also observed increased phosphorylation of Fms Related Receptor Tyrosine Kinase 1 (FLT1), Protein Kinase B (AKT), PDGFR, mammalian target of rapamycin (mTOR) and Epithelial Growth Factor Receptor (EGFR) on residues that promote their activity.

Overall changes were more pronounced in AFs than in MPM cells and some of these were specific to AFs, such as increased expression of the non-receptor tyrosine kinase SRC and mTOR; and decreased expression of p90 ribosomal S6 kinases (RPS6KA1, 2 and 3) and the receptor tyrosine kinase AXL (Fig. 5A).

In addition to validating many of these changes, western blotting also revealed that both AFs and MPM cells treated with condition media from AFs overexpress PDGFRA when compared to NFMet cells or NFMet-treated Met-5A cells, respectively (Fig. 5C, D, Supplementary Fig. 6C, D). Analysis of gene ontology revealed that the changes observed by RPPA were associated with common biological processes (BPs) in MMP cells and AFs, including BPs linked to cell adhesion/migration, hypoxic response and negative regulation of apoptotic processes (Supplementary Figs. 7 and 8), supporting the mutual increase in cell viability observed in co-culture experiments. The latter is further supported by the increased activity of the PI3K/mTOR signalling pathway, revealed by our RPPA (Fig. 5A, B), Western blotting (Fig. 5C, D) and gene ontology analysis (Supplementary Figs. 7 and 8), which is known to control cell survival [48].

Interestingly, TGFβ signalling was an over-represented BP in AFs at day 3 (Supplementary Fig. 7), corresponding to increased SMAD1/2 phosphorylation and expression in AFs (Fig. 5A) and increased production of TGFβ in MPM cells (Fig. 5B). As this pathway was switched off at Day 1 of MPM/AF interaction (Supplementary Fig. 6A, B), this likely reflects long-term adaptation of the signalling crosstalk. This evolution may be relevant to long term fibrotic response as TGFβ is heavily involved in pulmonary fibrosis [49].

We next investigated whether our observed changes in protein expression were consistent with those at mRNA levels between normal mesothelial and mesothelioma tissue in publicly-available microarray datasets. Three datasets, GSE112154, GSE51024 and GSE2549 were selected based on the number of available cases and analysis revealed that some of our target showed similar expression changes at mRNA levels as those seen in our cells at protein levels. This included RPS6KA3, RPS6KA2, ITGB3, BCL2 and ATM with consistent changes observed in two or more of the three datasets (Fig. 5E, Supplementary Fig. 9). This observation supports a wider relevance of our findings to mesothelioma in general. Finally, using the mesothelioma transcriptional data available through The Cancer Genome Atlas (TCGA) in conjunction with the cellular deconvolution algorithm Kassandra [50] revealed that expression of SRC, PDGFRA and PDGFRB (Fig. 5F) significantly correlated with the proportion of fibroblasts within the tumour microenvironment, further suggesting the relevance of these signalling changes to the productive phenotype of this disease.

### Therapeutic combinations targeting MPM/AF crosstalk show superior efficacy to standard-of-care cisplatin/pemetrexed

In view of the pro-tumorigenic BPs associated with our combined results, we tested whether targeting the proteins overexpressed or hyperactivated in response to the MPM/AF crosstalk could be of therapeutic benefit. We selected 11 clinically relevant drugs targeting these molecules (Supplementary Fig. 10) and compared their efficacy with that of cisplatin/pemetrexed, used here as standard-of-care reference.

In view of the known crosstalk between collagens and the Hedgehog (Hh) pathway in fibrosis [51] and the role of Hh signalling in cancer cell survival [52], we also selected the SMO inhibitor Vismodegib as an additional drug to be tested in our experiments (Supplementary Fig. 10).

**Fig. 6:**
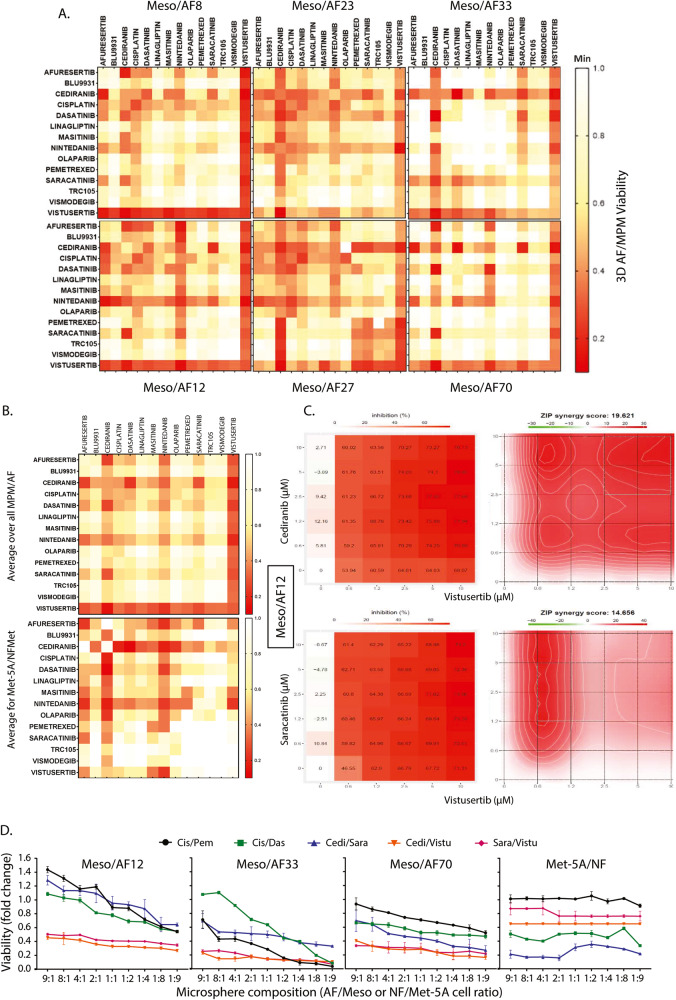
Combination of small molecule inhibitors for MPM/AF crosstalk signalling is superior to standard-of-care. **A** MPM/AF 3D spheroids were treated with combinations of the indicated compounds used at 10 µM and cell viability determined 72 h later using the Cell TiterGlo 3D assay prior to results being presented as heatmaps. Results are the average of *n* = 4 biological repeats. **B** Viability results of the combinations were averaged over all MPM/AF cell line pairs (upper panel) and compared with those obtained on Met-5A/NFMet co-cultures (lower panel). **C** Drug concentration range matrices for cediranib and saracatinib in combination with vistusertib in Meso/AF 12 cocultures were analysed for synergistic interaction using the ZIP method in SynergyFinder (https://synergyfinder.fimm.fi/). Left panels: the percentage of growth inhibition is represented as a heat map. Right panel: Contour line graph for ZIP scores. **D** MPM/AF or Met-5A/NFMet spheroids made using the indicated fibroblast to MPM ratios were treated with drug combinations for 72 h and the viability determined by the Cell TiterGlo 3D assay. Data points are the mean ± SEM from 3 independent biological replicates. Cis/Pem cisplatin/pemetrexed, Cis/Das cisplatin/dasatinib, cedi/vistu cediranib/vistusertib, sara/vistu saracatinib/ vistusertib.

We first tested our compounds individually in MPM/AF co-cultures, at a single dose of 10 µM (except for TRC105 used at 50 µM) as this dose was previously reported to efficiently inhibit the corresponding targets in cells [53–64]. These experiments revealed that most compounds had limited efficacy when used as single agents with the notable exception of the mTOR inhibitor, Vistusertib, which had single agent efficacy superior to that of the cisplatin/pemetrexed combination in all co-cultures (Fig. 6A, Supplementary Fig. 11A).

The results also highlighted the importance of testing the compounds on MPM/AF 3D co-cultures rather than on spheroids made solely of MPM cells. Indeed, increased resistance of spheroids to our targeted agents was observed when in presence of AFs, which mirrors results obtained earlier for the cisplatin/pemetrexed combination (Fig. 3G). As an example, Supplementary Fig. 11B shows that while Meso 70-alone spheroids were very sensitive to treatment with the AKT inhibitor, Afuresertib, co-culture of these cells with the corresponding AFs rendered these spheroids significantly (*p* < 0.0001) more resistant to the targeted agent.

Finally, these experiments revealed that co-culture with long-term activated AFs passaged 11 times in MPM condition media (P11 AFs) further limited the efficacy of some of the compounds. Indeed, we show that intracellular signalling evolved with time so that, while mTOR and SRC were still hyperphosphorylated in P11 AFs as compared to Met-5A-exposed MRC-5 fibroblasts (Supplementary Fig. 11C, D), the increase in AKT phosphorylation observed in day-3 AFs (Fig. 5A–C) had disappeared at this late time-point (Supplementary Fig. 11C). This was associated with a loss of activity of the AKT inhibitor Afuresertib in most co-culture cell line pairs (Supplementary Fig. 11E). Hence, all subsequent drug testing was performed in cocultures with P11 AFs.

Because of the limited activity of most compounds used as single agents, we next investigated pairwise drug combinations. This revealed many combinations that were either superior or as potent as the standard-of-care cisplatin/pemetrexed (Fig. 6A). When the effects over all MPM/AF co-cultures were averaged and compared to those on NF/Met-5A cocultures, two combinations showed superior activity over cisplatin/pemetrexed in the malignant co-cultures while having limited toxicity on mesothelial cells (Fig. 6B): cediranib/vistusertib and saracatinib/vistusertib. These combinations were synergistic as determined by the zero interaction potency (ZIP) model, showing maximal synergy at high nanomolar concentrations of all drugs with corresponding >50% loss of cell viability (Fig. 6C and Supplementary Fig. 12A). Moreover, this synergy was still observed with Meso/AF co-cultures using AFs under long-term culture (>2months, 20 passages) in conditioned media from mesothelioma cells, suggesting that these treatments could be applicable to late-stage disease (Supplementary Fig. 12B).

Because disease in patients can present with varying degree of fibroblast infiltration and AFs cause drug resistance when co-cultured with MPM cells, we tested to what extent the AF to MPM ratio could influence response to our two selected combinations. While high AF proportion in coculture spheroids lead to resistance to cisplatin/pemetrexed, the efficacy of the cediranib/vistusertib and saracatinib/vistusertib combinations were conserved at all AF/MPM ratios (Fig. 6D). This difference was not linked to the use of targeted compounds, as the combination between cediranib and saracatinib and that of cisplatin with dasatinib appeared sensitive to AF ratio similarly to cisplatin/pemetrexed (Fig. 6D). In contrast, the ratio of NFMet to Met-5A cells had no impact on the efficacy of all drugs combination, showing again the prosurvival effect to be specific to AFs (Fig. 6D). These experiments also further confirmed that cediranib/vistusertib and saracatinib/vistusertib had limited toxicity on the NF/Met5A co-cultures, providing a therapeutic window for the use of these drug combinations.

Finally, as fibrosis is a major cause of morbidity in mesothelioma patients, we investigated whether vistusertib, saracatinib and cediranib could inhibit the activation state (expression of αSMA and FAP) or the productive phenotype (expression of FN) of fibroblasts. We tested the impact of drug treatment of mesothelioma cells (Supplementary Fig. 13A) and AFs (Supplementary Fig. 13B) on their ability to activate naive fibroblasts. Conversely, we tested whether treatment of MRC-5 fibroblasts prevented their activation by mesothelioma cells and AFs (Supplementary Fig. 13C). These experiments revealed that treatment of mesothelioma cells had limited effect on their ability to activate productive fibroblasts, except with vistusertib that decreased the expression of FN in all conditions tested. In contrast, treatment of AFs impacted their ability to activate productive fibroblasts, with our compounds inhibiting the expression of one or more markers in all conditions. Similarly, treatment of MRC-5 fibroblasts prevented their activation into productive AFs by mesothelioma cells or AFs with several markers being impacted in all conditions. Importantly, the same markers were not always impacted by the same compounds between conditions and a single drug usually failed to inhibit all markers, further suggesting the expected therapeutic benefit of drug combinations.

### Graph-based network propagation helps guide in vivo testing

**Fig. 7:**
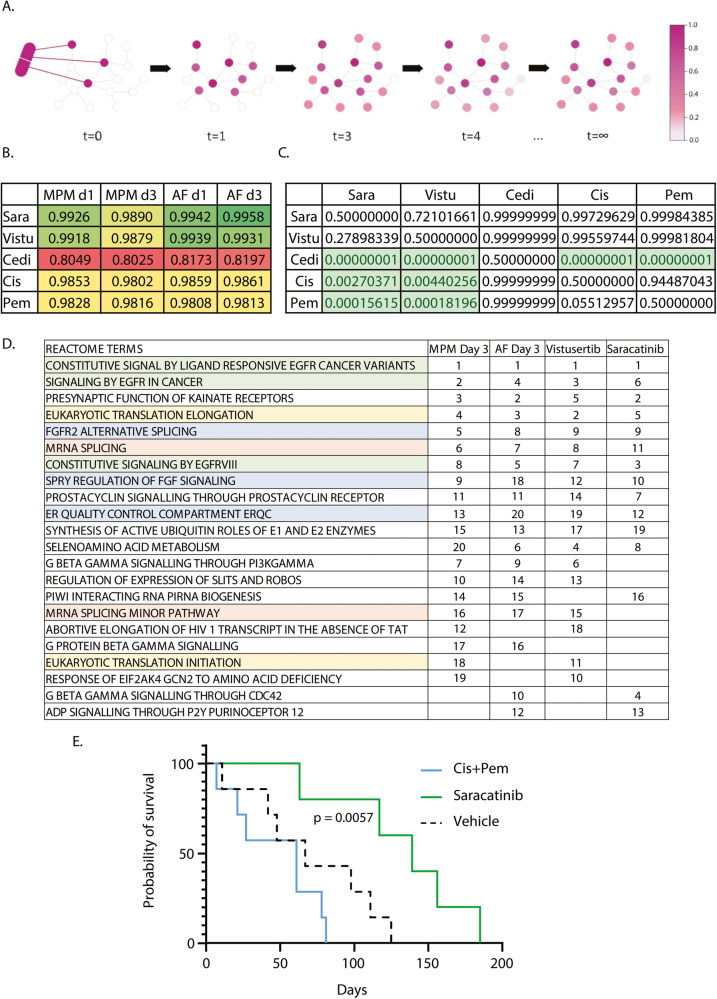
Dynamic network guides selection of therapeutic intervention in mouse model. **A** Diagram representing the generation of diffusion profiles for drugs and phenotypes using a random walk-based network propagation algorithm. Upon convergence, the diffusion profile measures how often each node in the network is visited, revealing the most affected proteins. **B** Results of Spearman correlation between drugs and phenotypes diffusion profiles. Green colour highlights higher, and red colour lower, correlation. Sara saracatinib, Vistu vistusertib, Cedi cediranib, Cis cisplatin, Pem pemetrexed. **C** Table of *p*-values of correlations in (**B**). Each cell contains the *p*-value that correlations between a drug in row and phenotypes are lower than correlations between a drug in column and phenotypes. *P*-values confirm that cediranib, cisplatin, and pemetrexed have significantly lower correlation with phenotypes than saracatininb and vistusertib. **D** 20 first Reactome terms from GSEA analysis associated with the network propagation for Vistusertib, Saracatinib and the proteomic data for MMP or AF at day 3 of CM treatment. Numbers represent the ranking of the process for each category. **E** Saracatinib treatment increases the survival of asbestos-exposed *Nf2/Bap1/Cdkn2a* triple-floxed mice over cisplatin/pemetrexed (Cis + Pem) combination. Statistics: Log-rank Mantel–Cox test between Vehicle and Cis + Pem.

To learn how drugs and phenotypes affect proteins overall, we leveraged a random walk network propagation algorithm (Fig. 7A). Network propagation propagates the effects of a drug or phenotype across a protein–protein interaction network generating *diffusion profiles* that reveal the most affected proteins. Random walks start at the differentially abundant proteins in drug treatment/phenotype. Upon convergence, the diffusion profile measures how often each node in the network is visited, revealing the most affected proteins. We computed Spearman correlation between diffusion profiles and drugs (Fig. 7B) which revealed that saracatinib and vistusertib affect to a significantly higher degree than cediranib, cisplatin or pemetrexed proteins like those impacted by the phenotypic changes undertaken by the MPM cells and AFs at day 1 and 3 of interaction (Fig. 7C). Indeed, a gene set enrichment analysis revealed extensive overlap of enriched gene sets between phenotypes, saracatininb and vistusertib (Fig. 7D).

Based on these results, we tested in vivo the efficiency of the saracatinib/vistusertib combination in prolonging survival of *Nf2/Bap1/Cdkn2a* triple-floxed mice [65] exposed to asbestos. This mouse model develops tumours positive for WT1, a marker for mesothelioma, 75 days post induction (Supplementary Fig. 14A). While administration of vistusertib alone did not significantly improve survival of the mice as compared to vehicle only and its combination with saracatinib proved toxic to the animals, saracatinib alone significantly (*p* < 0.05) improved median survival of the mice by 72 days (Supplementary Fig. 14B–D) and was significantly (*p* = 0.0057) more effective than cisplatin/pemetrexed which did not significantly improve overall survival in this model (Fig. 7E). Hence our data suggest that saracatinib could prove potent as a single agent in the treatment of mesothelioma in the clinic.

## Discussion

Pleural mesothelioma (MPM) is accompanied by extensive fibrosis which is a major cause of symptoms and death. The microenvironment of MPM includes cancer-associated fibroblasts (CAFs), endothelial cells, macrophages and other immune cells. CAFs are the main component of the MPM microenvironment [7] and in general are the principal contributor to fibrosis in cancer [66]. High expression of CAF-associated markers in MPM patients correlates with poor prognosis [5], as it has in other cancers [67]. Despite this, studies on the role of CAFs in the biology of the disease and their crosstalk with mesothelioma cells have been limited [5–7, 10, 68, 69]. A few studies have suggested potential therapeutic targets for MPM based on crosstalk between lung fibroblasts and mesothelioma cells. For instance, MPM cells promoted production of hepatocyte growth factor (HGF) by lung fibroblasts, which then drove invasiveness of MPM cells [6]. Similarly, two other studies highlighted the role of fibroblast-secreted HGF in promoting the growth of MPM cells [7, 69], and inhibitors of c-MET, the receptor for this growth factor, prevented this effect. In addition, Ries et al. showed that inhibition of PI3 kinase and WNT signalling in MPM/fibroblasts co-cultures achieved similar effects [69]. Li et al. also showed that inhibition of signalling through FGF2 and PDGF secreted by MPM cells to recruit/activate fibroblasts prevented MPM tumour growth in vivo [7]. However, in contrast with our results, except for the study by Mathilakathu et al. demonstrating CAF-induced changes in the activity of the MAP kinase pathway in MPM [22], none of these works highlighted that fibroblasts promoted therapy resistance in MPM co-cultures. This may be explained by their use of commercial MPM cell lines which we found to profoundly differ from patient-derived cell lines in terms of their secretion profile. The same reason may be behind the overall failure of targeting these signalling changes to improve clinical outcome [70–76].

We studied here the interaction between eight patient-derived mesothelioma cell lines [10] and lung fibroblasts. We found that MPM cells were able of both chemoattracting and activating fibroblasts, as demonstrated by the expression of CAF markers (Fig. 1C–E). We also show that these processes are self-propagating, with newly activated fibroblasts being able in turn to chemoattract and activate naive fibroblasts more efficiently than MPM cells (Fig. 3A, B). Self-perpetuating fibroblast activation has previously been described in cardiac fibrosis [77], but to our knowledge this is the first demonstration of this process in the cancer setting. Importantly, we did not in our experiments notice differences in the phenotype of AFs (as seen in Supplementary Fig. 2B) in relation to the genetic background of the activating mesothelioma cell line (Supplementary Fig. 1A). Similarly, examining Fig. 2A, D in view of Supplementary Fig. 1A, it is clear that the cytokines profile of AF cell lines did not cluster based on the genomic background of the activating mesothelioma cells. Moreover, it is clear that fibroblasts activated by the two biphasic mesothelioma cell lines used in this study (Meso-33 and Meso-70) did not cluster together in Fig. 2A, D. Hence, it appears that the subtype of the mesothelioma cells did not influence the nature of the secretions of downstream activated fibroblasts. However, we recognise that our failure to reveal such association may be due to an insufficient number of cell lines representative of each possible genetic background or mesothelioma subtypes.

We also demonstrated that AFs improve baseline survival of MPM cells and decrease their responsiveness to drug treatment (Fig. 3F, G and Supplementary Fig. 11B). Hence, we proposed that understanding the mediators of the crosstalk between CAFs and MPM cells may provide ways to improve therapeutic response in patients.

We initially profiled MPM cells and corresponding AFs for secreted cytokines/chemokines/acute phase proteins and growth factors. For the MPM cell lines, the profiles were compared to that of Met-5A mesothelial cells. Indeed, despite these cells showing several mutated tumour suppressor genes [78], they are often used as untransformed counterparts to MPM cells [79–82]. In contrast to previous reports, we identified ANG, CHI3L1 and DPP4 to be increased while ENG and FLT3LG were downregulated in the MPM cell lines tested as compared to the Met-5A mesothelial cells. ANG, CHI3L1, DPP4 and ENG have been involved in chemoattraction and/or activation of fibroblast and may mediate these in our system. Future experiments will focus on investigating the relative role of these factors in our observed phenotypes. Changes in the cytokine and growth factors profile of AFs were more pronounced, with increased expression of a large number of molecules known to promote fibroblast chemotaxis, activation and proliferation, including various FGFs, PDGFs, IGF1 and IGFBPs [83–85] (Fig. 2D). This was consistent with our observation that AFs are able to attract and activate naive fibroblasts (Fig. 3A, B).

We did not find a proliferative advantage of AFs over their naive counterparts, in contrast to what is reported in other cancers [26, 27]. Lack of proliferative advantage and the restraining activity of AFs on the growth of MPM/AF co-cultures may explain the very long pathogenesis of MPM. Finally, the cytokine/chemokine profile of AFs suggested an inflammatory phenotype that is consistent with the aetiology of the disease and the substantial immune infiltration observed in MPM [86].

It is a concern that factors previously reported as being secreted by MPM cells, such as FGF2, HGF, PDGF-AA and VEGF [7, 87] validated poorly across patient-derived and commercial mesothelioma cell lines in our study (Fig. 1A and Supplementary Fig. 2D). Previous data were generated using commercial cell lines with multiple passages in culture (MSTO-211H, Y-Meso-14, IST-Mes1, IST-Mes2, IST-Mes3, and MPP89 cells) that may poorly reflect clinically-relevant biology [10]. In our study we have tested multiple low-passage patient-derived cell lines and found a consistent set of cytokines, chemokines, acute phase proteins and growth factors to be produced as part of AF/MPM communication.

The relative roles of the fibrotic response in disease progression and suppression in MPM, and cancer in general, has often been debated [88, 89]. Our results are consistent with both roles. While we show that AFs are capable of restricting the growth of MPM cells in 3D spheroids (Fig. 3D), we also found that the ECM deposited by AFs activates naive fibroblasts and promotes 3D coculture spheroids growth and drug resistance (Fig. 4C, E, F). Hence, the reported dual role of CAFs may represent two subsequent stages, with initial fibroblast recruitment responding to disease spread, while production of a modified extracellular matrix later promotes disease progression.

Targeted therapy has been generally unsuccessful in the clinical management of MPM. This may be explained by the general lack of oncogenic drivers in this disease [2] as compared to other types of cancers, including lung cancers [90]. However, many autocrine signalling molecules reportedly produced by mesothelioma have been identified using cell lines that incompletely represent the clinical disease [7, 10, 87, 91]. Our results now identify several secreted factors that are consistently upregulated in our MPM and AF cell lines, and these are associated with activation of corresponding signalling pathways following crosstalk between MPM cells and fibroblasts (Fig. 5A–D).

This crosstalk leads to phenotypic changes associated with cancer progression, such as profibrotic activation of fibroblast by MPM cells and AF-induced drug resistance of MPM cells. Consequently, targeting these signalling pathways could be effective in controlling the disease. We find that combinations of small molecules targeting our observed intracellular signalling changes are more efficient than the current standard-of-care cisplatin/pemetrexed combination in decreasing the viability of MPM/AF co-cultures (Fig. 6A, B). Interestingly, a number of these show specificity for MPM/AF cocultures over that of the corresponding mesothelial cells/naive fibroblasts cocultures (Met-5A/MRC-5) (Fig. 6B, D). This may provide an opportunity rapid clinical translation, as the compounds used here have already all been tested in clinical settings. It has been shown that targeting mTORC1 and 2 reduces cell growth in human patient explants and increases survival in mouse models of mesothelioma [92]. However, clinical trial results revealed the lack of efficacy of single-agent mTOR inhibitors in MPM patients [93, 94] and so combination of these compounds with other targeted therapeutics may be more effective. Similarly, clinical trials failed to demonstrate activity of SRC inhibition in MPM patients [74, 95], although none of these used the later-generation inhibitor, Saracatinib. Here we show that combinations of the mTOR pathway inhibitor vistusertib with either the PDGFR inhibitor cediranib or the SRC family inhibitor saracatinib are synergistic at doses that are more efficacious than cisplatin/pemetrexed across our various MPM/AF cocultures (Fig. 6B, C). These three small molecules have been shown to be well tolerated in patients, even as part of therapeutic combinations [96–98].

Some caution is suggested by the toxicity of the combination of saracatinib and vistusertib in our genetically-engineered mouse model. In future experiments, toxicity may be addressed through the dose of each compound, better scheduling or combination with new generation improved SRC inhibitors [99]. Nevertheless, our results show that saracatinib as single agent provides superior activity in this model than the current standard-of-care, extending the medium survival of asbestos-treated animals by 72 days without noticeable toxicity. As this molecule is currently used in various trials (clinicaltrials.org) and dosage that is well-tolerated has already been determined in patients with lung cancer [100], we propose that saracatinib should be next tested in patients with mesothelioma.

In conclusion, this research has identified clinically actionable signalling crosstalk between MPM cells and AFs that triggers phenotypes associated with disease progression. Compounds targeting the various signalling changes are available and their combination shows superior efficacy in vitro to that of current standard-of-care. While additional research is now needed to understand how these could be efficiently translated in the clinic, our findings suggest that these offer hope for the better management of MPM, a disease in urgent need of novel more potent therapeutic strategies.

## Material and methods

### Cell culture

The foetal naive lung fibroblast MRC-5 cell line was obtained from the American Type Culture Collection (ATCC) (Manassas, VA, USA). All patient-derived human mesothelioma cell lines were developed at the MRC Toxicology Unit and have previously been reported [10]. These were cultured for ≤20 passages, a length of culture previously demonstrated not to be accompanied by additional genomic instability [10]. The SV40-immortalised mesothelial cell line Met-5A cells and mesothelioma cell lines NCI-H2052 and MSTO-211H were obtained from the ATCC. Cells were maintained at 37 °C in a humidified environment at 5% CO_2_. All cell lines (commercial and patient-derived) were maintained in RPMI (Roswell Park Memorial Institute)-1640 Medium (25 mM HEPES and NaHCO_3_) (Sigma Life Sciences, MO, USA), supplemented with 10% (v/v) foetal calf serum (FCS), 100 U/ml penicillin, 100 µg/ml streptomycin, 2 mM l-glutamine (Sigma Life Sciences, MO, USA).

### 3D spheroids

Three dimensional spherical organoids of cells were generated using the Nunclon Sphera 96-well, U-Shaped-Bottom Microplate system (Thermo Fisher Scientific, MA, USA). Each spheroid was composed of 1 × 10^4^ cells unless otherwise stated. Upon addition of the cells, plates were centrifuged at 300 × *g* for 1 minute to induce cell aggregation. Subsequently, spheroids were maintained at 37 °C for 24 h to allow for spheroid formation prior to further experimentation. The centrifugation step was repeated following the addition of any liquid. The spheroids were suspended in either medium alone or extracellular matrix derived from the corresponding cancer-associated fibroblasts. In co-culture experiments both cell types were seeded in 100 μl RPMI at a 1:1 ratio for a total of 1 × 10^4^ cells per microsphere.

### Live cell labelling

Prior to cell seeding, cells were differentially labelled with fluorescent dyes. The violet Cell Proliferation Dye eFluor 450 (Thermo Fischer Scientific, MA, USA) was used for mesothelial and mesothelioma cells, while the Vybrant CFGA SE Cell Tracer (Thermo Fischer Scientific, MA, USA) green dye was used for the labelling of fibroblasts. Cells were suspended in fluorescent dye diluted in PBS at a final concentration of 1 μg/ml and incubated at 37^o^C, 5% CO_2_ for 20 min. Subsequently, the cells were resuspended in fresh pre-warmed RPMI and incubated 37^o^C, 5% CO_2_ for 30 min to allow the dye to undergo acetate hydrolysis before being used for experiments.

### Conditioned media preparation and use

Medium from cells grown to 70–80% confluency was harvested and filtered through a 0.22 μm Minisart Syringe Filter (Sartorius, Epson, UK) to remove cell debris. Conditioned medium was always diluted 1:1 with fresh RPMI-1640 (supplemented with 10% FCS and 1% PSG) before use in seeding cells, to ensure the presence of adequate levels of nutrients.

### Actin cytoskeleton staining

Cells in 96-well plates were fixed with a 4% PFA for 20 min, permeabilised with 0.1% Triton X-100 in PBS for 5 min, and blocked using 3% Bovin Serum Albumine (BSA) in PBS. Actin was stained with Alexa Fluor 488-Phalloidin (Molecular Probe) and nuclear DNA revealed using DAPI (Molecular Probe). Images for 36 fields per well were acquired using an ImageXpress high-content imager (Molecular Devices).

### Extracellular matrix production and decellularization

5 × 10^5^ MRC-5 fibroblasts were seeded in 10 cm dishes in conditioned medium acquired from mesothelioma or mesothelial cells. Cells were maintained in culture for 15 days, with conditioned media renewals every 72 h. Following this, the medium was aspirated and the dishes washed with PBS. Decellularization was initiated by addition of pre-warmed extraction buffer (20 mΜ ΝΗ_4_ΟΗ, 0,5% (v/v) Triton X-100 in PBS) for 2 min to induce cell lysis. Subsequently, the extraction buffer was aspirated partially and the collagenous matrix underwent a PBS washing step, prior to all liquid being aspirated out of the dish. All residual DNA was removed from the matrix via incubation with 10 μg/ml DNase I (Sigma-Aldrich, Dorset, UK) for 30 min at 37 °C, 5% CO_2_. Subsequently, the matrix was washed twice with PBS and harvested into 2 ml collection tubes and diluted with 1 ml PBS. The collected ECM was mildly sonicated for 30 min, to ensure dissolution of collagenous aggregates, prior to storage at −80 °C.

### Cytokine, chemokine and acute phase proteins profiling

The Proteome Profiler Human XL Cytokine Array Kit (R&D systems, Minneapolis, MN, USA) was used for the relative evaluation of corresponding proteins in cell lysates, according to the manufacturer’s protocol. MRC-5 fibroblasts were treated with conditioned media derived from individual mesothelioma cell lines for 7 days with culture medium changed every 2 days. “Control” MRC-5 fibroblasts were cultured in conditioned media derived from the mesothelial Met-5A cell line. The same course of treatment was implemented for mesothelioma cells grown in conditioned media derived from the corresponding cancer associated fibroblasts with the control condition being Met-5A cells grown in conditioned media derived from MRC-5 fibroblasts. Total protein was extracted and quantified. All components of the Proteome Profiler Array were equilibrated to room temperature (RT) prior to use. Firstly, the nitrocellulose membranes, each containing 105 different capture antibodies in duplicate, were placed in incubation trays and blocked with 2 ml of 1× Array Buffer 6. This incubation step was performed on an orbital shaker for 1 h at RT. The membranes were then incubated overnight at 4 °C with 250 μg of total protein diluted in 1.5 ml of Array Buffer 6. The membranes were then incubated thrice for 10 min on an orbital shaker with 1× Wash Buffer. Subsequently, the membranes were incubated with 30 μl of Detection Antibody Cocktail diluted in 1.5 ml of 1× Array Buffer 4/6 for 1 h on an orbital shaker at RT, followed by washing as above. Each membrane was then incubated with 2 ml of the 1× Streptavidin-HRP reagent for 30 min on an orbital shaker at RT, followed by washing as above. The antibody arrays were then developed using the Pierce ECL Western Blotting Substrate (Thermo Fisher Scientific, MA, USA) and imaged on the FUSION SOLO quantitative luminescence imaging system (Analis, Suarlée, Belgium). Relative quantification of each analyte was done by measuring pixel density using ImageJ software (National Institutes of Health, MD, USA). Results were subjected to hierarchical clustering using the ClusterMaker plugin under the Cytoscape platform.

### Growth factor profiling

The RayBio C-Series Human Growth Factor Antibody Array C1 (RayBiotech, GA, USA) was used for the semi-quantitative detection of 41 growth factor molecules in the proteome of AFs, according to the manufacturer’s protocol. Naive MRC-5 fibroblasts were treated with conditioned media from individual mesothelioma cell lines for 7 days, with media changes every 2 days. “Control” MRC-5 fibroblasts were cultured in conditioned media derived from Met-5A cells. Subsequently, protein was extracted and quantified. All reagents of the Growth Factor Array kit were equilibrated to RT prior to use. Firstly, the antibody arrays were placed into incubation trays and blocked with 2 ml of Blocking Buffer for 30 min at RT. Subsequently, the arrays were incubated overnight at 4 °C with 250 μg of total protein diluted in 2 ml of Blocking Buffer. The arrays were then washed for 5 min thrice with 2 ml of 1× Wash Buffer on an orbital shaker and then washed twice for 5 min with 2 ml of 1× Wash Buffer II on an orbital shaker. The arrays were then incubated overnight at 4 °C with 1 ml of the 1× Biotinylated Antibody Cocktail prior to washing with Wash Buffers I and II, as described above. The arrays were then incubated overnight at 4 °C with 2 ml of the 1× HRP-Streptavidin concentrate, followed by the washing with Wash Buffers I and II, as above. Finally, the arrays were developed using the Pierce ECL Western Blotting Substrate (Thermo Fisher Scientific, MA, USA) and imaged on the FUSION SOLO quantitative luminescence imaging system (Analis, Suarlée, Belgium). Relative quantification of analytes was done by measuring pixel density using the FIJI ImageJ software (National Institutes of Health, MD, USA) and hierarchical clustering performed using the ClusterMaker plugin under the Cytoscape platform.

### Reverse phase protein array (RPPA)

MRC-5 fibroblasts, Met-5A cell or mesothelioma cells (1 × 10^6^ cells per condition) were incubated with the relevant conditioned media, as described above, for 24 h and 72 h. Cell lysates were mixed with 4× SDS sample buffer without bromophenol blue and supplemented with 10% 2-β-mercaptoethanol, at a final concentration of 2 mg/ml and final volume of 150 μl. Samples were stored at −80 °C prior to further analysis. The Reverse Protein Array analysis was performed at the Cancer Research UK Scotland Centre’s Host and Tumour Profiling Unit. Samples were denatured by heating to 95 °C for 5 min prior to printing as serial dilutions (1.50 mg/ml, 0.75 mg/ml, 0.375 mg/ml and 0.1875 mg/ml) in arrays consisting of 36 × 12 spots at a 500 μm spot-to-spot distance on the Aushon 2470 Arrayer Platform (Quanterix, MA, USA) using 8 × 185 μM pins, with 2 deposition rounds per feature. Each sample dilution series was spotted on all arrays with 8 arrays per slide on single pad Supernova nitrocellulose slides (Grace BioLabs, OR, USA). Sample loading on the slides, for normalisation purposes, was determined with Fast-Green dye staining and scanning using the InnoScan 710 slide scanner (Innopsys, Carbonne, France) at 800 nm. The RPPA slides were then washed with deionized water for 4 × 15 min on a platform shaker, prior to a 15 min incubation with Antigen Retrieval Reagent (1× Reblot strong). The slides then underwent two more 5 min washing steps with deionized water. Subsequently, the RPPA slides were placed in a ProPlate chamber (Grace BioLabs, OR, USA) in fresh deionized water and washed with 1× PBS twice for 5 min. The slides were then incubated for 10 min in the Superblock T20 Blocking Buffer (Pierce, Thermo Fisher Scientific, MA, USA), prior to TBST washing twice for 5 min. The RPPA slides were then incubated for 60 min with the 120 primary antibodies all diluted at 1:250 in Superblock buffer prior to two more TBST washes (5 min each) and a second 10 min incubation with the Blocking buffer, as described above. The RPPA slides were then incubated for 30 min with the DyLight-800-labelled anti-species secondary antibody diluted at 1:2500 in Superblock, followed by two 5 min washes in TBST and a final rinse with deionized water. Non-specific background signal was determined for each slide by performing the primary antibody incubation step solely with Superblock, without antibodies, on 1 array followed by fluorescent tracer secondary antibody. The slides were dried for 10 min at RT prior to imaging with an InnoScan 710 slide scanner (Innopsys, Carbonne, France). Microarray images were analysed using the Mapix software (Innopsys, Carbonne, France). The spot diameter of the grid was set to 270 μm. Background intensity was determined for each spot individually and subtracted from the sample spot signal, thus generating a net signal for each spot. Relative quantification of each analyte was determined for each spot by measuring fluorescence intensity. The validity of the serial dilutions was ensured by generating a linear fit curve from the 4-point dilution series for all samples, on all arrays, using a flag system where an *R*^2^ value was generated and *R*^2^ > 0.9 values were deemed good. Relative fluorescence intensity (RFI) values corresponding to relative abundance of total and phosphorylated proteins across the samples set were calculated and normalised to total protein by calculating the ratio of Antibody RFI/Fast-Green RFI for all samples.

### Liquid chromatography/mass spectrometry analysis (LS–MS/MS)

The decellularized extracellular matrix was dissolved in 300 μl of Urea Buffer (8 M urea, 100 mM ammonium bicarbonate, 5 mM dithiothreitol) by shaking for 20 min at 4 °C. Total protein concentration for each sample was measured using the Pierce 660 nm Protein Assay Kit (Thermo Fisher Scientific, MA, USA). Subsequently, 33 μg of total protein per condition were incubated with 10 mM TCEP (tris(2-carboxyethyl)phosphine); Sigma Aldrich, Dorset, UK) for 1 h at 56 °C to reduce disulphide bonds and alkylated with 100 mM iodoacetamide, a 40 min incubation at RT. Samples were then diluted to a final concentration in 2 M urea and 100 mM ammonium bicarbonate prior to protein digestion with 1.3 μg of trypsin (Promega, Southampton, UK) overnight at 37 °C. Peptides were then desalted on a Sep-Pak C18 Light Cartridge (Waters Corporation, MA, USA) and dried in a SpeedVac concentrator (Thermo Scientific, Thermo Fisher Scientific, MA, USA). Samples were resuspended in 33 μl of 100 mM tetraethylammonium bicarbonate buffer prior to labelling with 1/3 rd of Tandem Mass Tag (TMT) 10-plex Isobaric Mass Tag Labelling Reagents (Thermo Scientific, Thermo Fisher Scientific, MA, USA) as per manufacturer’s protocol. Briefly, TMT labelling reagents were equilibrated to RT and dissolved in 42 μl of LC–MS grade anhydrous acetonitrile (Thermo Scientific, Thermo Fisher Scientific, MA, USA). A volume of 14 μl of each TMT labelling reagent was added per sample (33 μg of digested protein per condition) and incubated for 1 h at RT. Reactions were quenched by addition of 5% (w/w) hydroxylamine (H_3_NO) and incubated for 15 min at RT. Labelled peptides for each condition were pooled and desalted on a Sep-Pak C18 Plus Cartridge and dried using in a SpeedVac. Dried samples were resuspended in 2% (v/v) acetonitrile, 0.1% (v/v) formic acid and aliquots equivalent to 6 μg of peptides were analysed in triplicates by Liquid Chromatography/Mass Spectrometry (LC–MS/MS) using the UltiMate 3000 RSLCNano Liquid LC system coupled to the Q-Exactive Plus Hybrid Quadrupole-Orbitrap Mass Spectrometer (Thermo Scientific, Thermo Fisher, MA, USA). Peptides were separated on 75 μm × 50 cm Acclaim PepMap100 C18, 3 μm column over 120 min. Acquired LC–MS/MS data were processed by the MaxQuant quantitative proteomics software package (Version 1.6.2.6; Max Planck Institute of Biochemistry, Munich, Germany) and searched against the human protein database Swissprot (20,317 protein sequences, downloaded on 03/08/2018) using the Andromeda search engine and the following parameters: (1) MS-2 reported ion quantification with reported ion mass tolerance of 0.003 kDa, (2) maximum of 2 miscleavages, (3) fixed modification of carbamidomethylation of cysteins, TMT label on lysin and peptide N-terminus, (4) variable modification of deamidation of glutamine and asparagine, oxidation of methionine, (5) calibration search peptide tolerance of 20 ppm and 6) 1% FDR threshold on peptide and protein level. Results from three replicates were further combined and processed using the Perseus Software (Version 1.5.6.0; Max Planck Institute of Biochemistry, Munich, Germany). Raw protein quantity values were Log2 transformed and each sample was median-centred. Proteins identified in only one technical replicate were removed from the dataset while quantity of the remaining proteins was calculated as average across all three replicates. The values obtained for proteins in the AF-derived ECM were subsequently normalised to those in the NFMet-derived ECM sample (NFMet). The proteomics datasets are available on Proteomexchange with assession number PXD040492.

### Functional interaction network building

Data tables containing protein names and corresponding expression/phosphorylation log2 fold changes normalised to the MRC-5 cells treated with the conditioned medium from Met5A cells (NFMet) condition were imported into Cytoscape (https://cytoscape.org/) and directed functional interaction networks built using the ReactomeFI plugin (https://reactome.org/tools/reactome-fiviz). Linkers were introduced to maximise network connectivity. The networks were then subjected to gene ontology analysis under ReactomeFI and significantly enriched biological processes selected based on a false discovery rate <0.001.

### DAVID and STRING analysis

The Database for Annotation, Visualisation and Integrated Discovery (DAVID; Version 6.8) and the online database STRING (STRING CONSORTIUM: Swiss Institute of Bioinformatics, Novo Nordisk Foundation Protein Research Centre & European Molecular Biology Laboratory; https://string-db.org; Version 11.0) were used for functional annotation, enrichment analysis and KEGG and Reactome pathway mapping of the cytokine and growth factor array results.

### Bioinformatics analysis of publicly available data

GEO microarray datasets (GSE112154, GSE51024 and GSE2549) comparing the gene expression profiles of normal mesothelial and mesothelioma tissue were downloaded from the NCBI GDS website. The gene probes used for the analysis are indicated in the corresponding figures. GDC TCGA RNA-sequencing data from the TCGA were downloaded from the Xena browser and the corresponding Kassandra cellular deconvolution results from BostonGene website (science.bostongene.com). The Data were analysed in R using linear modelling and Spearman correlation and plots generated using the *lattice* package and base R.

### Drug treatment of 3D spheroids

With the exception of cisplatin, all small-molecule inhibitors were reconstituted upon purchase with Dimethyl sulfoxide (DMSO) to create a 10 mM drug stock. TRC105 neutralising antibody which was kindly provided by TRACON pharmaceuticals in liquid form. All stock solutions were aliquoted upon reconstitution and stored at −80 °C. A 100 mM stock concentration of Cisplatin (Sigma Life Sciences, MO, USA) was freshly prepared in DMSO prior to use. Afuresertib, Blu9931, Dasatinib, Linagliptin, Masitinib, Nintedanib, Olaparib and Pemetrexed were purchased from Selleckchem (Houston, TX, USA). Cediranib and Saracatinib were from Generon (Slough, UK). Vismodegib and Vistusertib were from ApexBio (Stratech, Cambridge, UK). Spheroids were treated 24 h post cell seeding with 10 μΜ (50 μΜ for TRC105) of drug for 72 h, prior to measurement of cell viability using the Cell Titre-Glo 3D assay (Promega). Microscopic images of the spheroids were acquired using the EVOS Core Cell Imaging System (Thermo Fisher Scientific, MA, USA). Graphs were generated using the Prism GraphPad software.

### Synergy analysis

Synergy analysis was performed using the SynergyFinder Application (https://synergyfinder.fimm.fi). Systematic evaluation of pairwise drug combination efficacy was assessed based on the Zero Interaction Potency (ZIP) model.

### Cell proliferation assay using CFSE staining

Cells were resuspended at a concentration of 1 × 10^6^ cells/ml in 0.1% BSA/PBS and labelled with 10 μM CFSE for 5 min at 37 °C. Five times volume of ice cold DMEM was added to the cells for 5 min on ice in order for dye quenching followed by three washes with 1× DMEM to remove the excess dye before re-plating onto 6 cm dishes. At each point cells were harvested, washed once with 1× PBS and fixed with 4% paraformaldehyde at room temperature for 15 min. Finally, cells were washed three times with PBS, re-suspended in 1 ml PBS and kept at 4 °C before flow cytometry analysis on a BD FACS Canto.

### Cell cycle analysis by propidium Iodide staining

Cells were harvested, washed once with PBS and fixed using drop-wise addition of ice cold 70% ethanol under vortexing followed by 30 min incubation at 4 °C. Pellets were washed twice with PBS prior to addition of 50 μl of ribonuclease (100 μg/ml) (Sigma-Aldrich) for 15 min. Following addition of 50 μg/ml propidium iodide (Sigma-Aldrich) the DNA profile was acquired using flow cytometry on a BD FACS Canto.

### Western blotting

Cellular proteins were extracted using a Radio immunoprecipitation assay buffer (RIPA) (50 mM Tris-Cl, pH 7.4, 0.1% SDS, 0.1% sodium deoxycholate, 1% Triton X-100, 150 mM NaCl, 2 mM EDTA, 5% Glycerol supplemented with protease inhibitors cocktail tablets (Roche Diagnostics), 10 mM βGlycerophosphate, 1 mM sodium orthovanadate, 10 mM sodium fluoride). Equal protein amounts were diluted in 2× Laemmli buffer, boiled for 5 min and analysed by SDS-PAGE/Western blotting using the relevant primary antibodies and HRP-conjugated secondary antibodies. Immunoreactivity was revealed using Pierce ECL or SuperSignal substrates. Blots were visualised using the quantitative Fusion Solo Chemiluminescence Imager and image analysis was performed using FIJI.

### Antibodies

Antibodies against AKT (#9272), Phospho-AKT (Ser473) (#9271), αSMA (#14968S), Phospho-BAD (Ser136) (#9295), BCL-xL (#2764), Β-Catenin (#8480), Caspase 3 Cleaved (Asp175) (#9661), DPP4 (#67138), FAK (#3285), FGF19 (#83348), IL-6 (#12153S), mTOR (#2983), Phospho-mTOR (Ser2481) (#2974), p70S6K (#9202) were from Cell Signalling Technology. Antibodies against BCL2 (sc-509), CXCL10 (sc-101500), FBN2 (sc-393968), IL-8 (sc-8427), MIC-1 (GDF-15) (sc-377195), MIF (sc-271631) were from Santa Cruz Biotechnology. Antibodies against ANGPT2 (#6763), MCP1 (CCL2) (#7201), IL-17A (#4887) were from ProSci. Antibodies against AR (ab45089), COL4A3 (ab6586), ENG (ab169545), FAP (ab207178), IL-1A (ab227482), IL-8 (ab18672) were from Abcam. Antibodies against FN (#610078) and HIF-1A (#610959) were from BD Transduction Labs. The antibody against Phospho-BAD (Ser112) (#9291) was from Biolabs.

### Caspase 3/7 activity assay

The Caspase-Glo 3/7 Assay (Promega) was performed according to the manufacturers’ instructions. Briefly, 100 µl of Caspase-Glo 3/7 reagent was added per 96-well and luminescence measured after 15 and 60 min of incubation at room temperature (PHERAstar microplate reader).

### Transwell migration assay

All transwell migration experiments were conducted using the Sarstedt 8 μm TC Hanging Inserts for Tissue Culture Plates (Sarstedt, Numbrecht, Germany). One hundred thousand MRC-5 fibroblasts were seeded within the Boyden chamber insert in 200 μl of serum-free RPMI. The inserts were then placed in a 24-well plate, where each well contained 750 μl of conditioned media derived either from mesothelial cells, mesothelioma cells, cancer-associated fibroblasts or naive fibroblasts. At the appropriate time point (6, 12 or 24 h), all media were aspirated from the chambers prior to gently scrapping any remaining cells from the upper side of the inserts’ membrane with a cotton swab. Subsequently, inserts were placed in 750 μl of 4% (v/v) paraformaldehyde (Sigma Aldrich, Dorset, UK) in PBS per well in a 24-well plate for 10 min. The chambers were then air-dried for 15 min and transferred to a 24-well plate containing 750 μl per well of 0.5% (w/v) crystal violet in 25% (v/v) methanol for 10 min. Any residual stain was then gently cleaned with a cotton swab and the chambers air-dried overnight prior to microscopic acquisition.

### Dynamic network analysis

We extracted a protein–protein interaction network and drug-protein connections as previously described in our work [101, 102]. In brief, we constructed a human genome network of 20,256 proteins using data extracted from STRING (string-db.org), UniProt (uniprot.org), COSMIC (cancer.sanger.ac.uk/cosmic), and NCBI Gene (ncbi.nlm.nih.gov/gene/) public databases. We filtered protein–protein connections to keep only those recorded with high confidence (confidence score greater than 900). We extracted drug-protein connections from STITCH database (http://stitch.embl.de/), and we filtered connections to only keep those connections with high confidence (confidence score greater than 700). First, from the protein abundance profiles for each of the phenotypes, we computed differentially-abundant proteins as those with an average log(treated vs control) lower than −0.5 or higher than 0.5, and with a *q*-value ≤ 0.05 (Benjamini–Hochberg correction). Using differentially abundant proteins as seeds, we perform Random Walk with Restarts [103] on the protein–protein interaction network. To select an appropriate value for the restart probability, we followed the strategy detailed in [104]. We first ran the random walk algorithm until convergence with a restart probability of zero. We took the highest score across all proteins and ran the random walk algorithm with a restart probability equal to 4 times the highest value recorded. We computed random walk profiles of tested drugs following the same procedure. For each phenotype and drug random walk profile, we ran the PreRanked module of Gene Set Enrichment Analysis software version 4.1.0 (GSEA) to find over-represented pathways in the top-scoring proteins. We additionally measured Spearman correlation between drug and phenotype random walk profiles and used Student’s *t*-test to compare drug-phenotype correlations.

### Animal experiment

All experiments involving mice were approved by the local ethics committee and conducted in accordance with UK Home Office license number 70/7950 (Murphy). Mice were housed on a constant 12 h light/dark cycle; fed and watered ad libitum. *Nf2/Bap1*/*Cdkn2a* floxed mice were described previously [65]. To induce allele recombination, administration of lentiviral vector carrying CRE recombinase was performed on adult mice, aged 8–10 weeks, via single intrapleural injection of 3 × 10^7^ viral particles per mouse. Asbestos (amosite) was administered via single intrapleural injection of 25 μg fibres, per mouse, 10 days post CRE administration, as previously described [92]. A minimum of 6 mice per treatment arm was used as prior research had found this number to be sufficient to generate statistically significant differences [105]. Mice were randomly allocated to treatment groups by technical staff blinded to the treatments administered. Mice started treatment 75 days post CRE administration. Saracatinib (HY-10234) and Vistusertib (HY-15247) were each dissolved in 0.5% HPMC + 0.1%Tween 80 in H_2_O (vehicle). Saracatinib only was administered at 10 mg/kg/daily, Vistusertib only was administered at 15 mg/kg/day for 3 weeks (7 days on, 2 days off, 5 days on, 2 days off, 5 days on). Where mice were treated with Saracatinib+Vistusertib in combination, drugs were administered at 3.75 mg/kg/day and 5 mg/kg/day respectively, with the same dosing schedule. Drugs were administered via intraperitoneal injection. The control arm received vehicle (IP) on the same schedule. Routine health monitoring was performed by facility personnel without knowledge of experimental details. Humane end points were defined as exhibition of 2 or more symptoms: moderate (10–19%) weight loss, elevated breathing, hunched appearance and/or overall malaise. All mice were sacrificed using a schedule 1 procedure.

### Statistics

Statistical testing was performed using Prism (GraphPad) or Microsoft Excel (Microsoft). Two-by-two comparisons for biological experiments were statistically analysed using two-tailed unpaired Student’s *t*-test as the data is expected to be normally distributed. Multiple comparisons were analysed by ANOVA Benjamini-Hochberg correction. Animal experiments data was analysed using the Log-rank (Mantel–Cox) test. Variance between groups were compared to guaranty that they were similar enough to allow the use of the proposed statistical tests. For all statistical analyses, significance was accepted at the 95% confidence level (*P* < 0.05) and significance levels indicated.

### Reporting summary

Further information on research design is available in the Nature Research Reporting Summary linked to this article.

### Supplementary information

Reporting Summary

Supplementary Figures

Original blots

Supp Excel file

## Data Availability

The proteomics datasets corresponding to the mass-spectrometry of the extracellular matrix deposited by the fibroblasts used in this study are available on Proteomexchange with assession number PXD040492.
