# Supplementary material for: Crosstalk with lung fibroblasts shapes the growth and therapeutic response of mesothelioma cells
Source: Cell Death Dis. 2023 Nov 8;14(11):725. doi: 10.1038/s41419-023-06240-x (PMC10632403; doi:10.1038/s41419-023-06240-x)
Supplement: Supplementary file 2 — Supplementary Figures [file 41419_2023_6240_MOESM2_ESM.pdf]

Supp Fig 1

A.

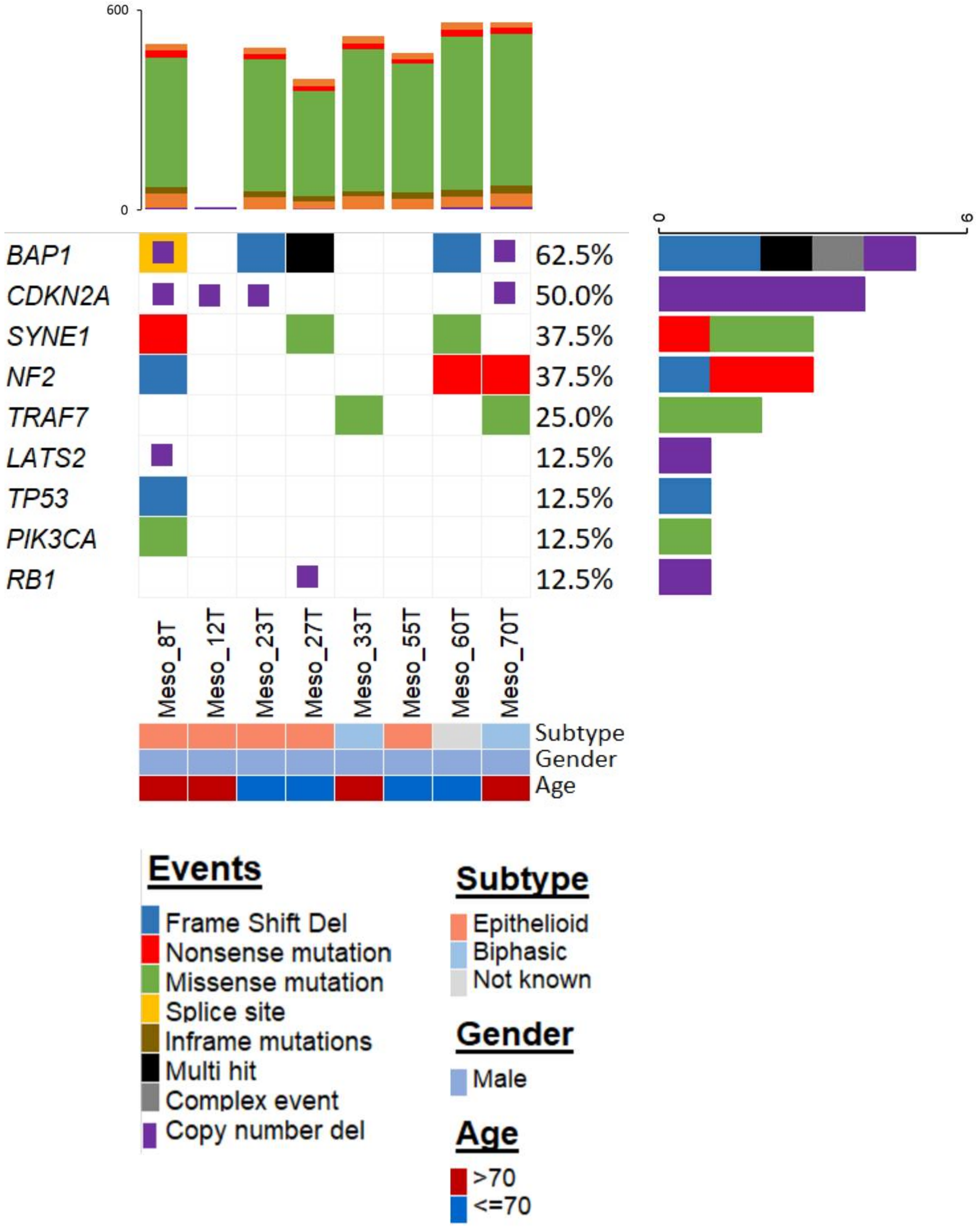

B.

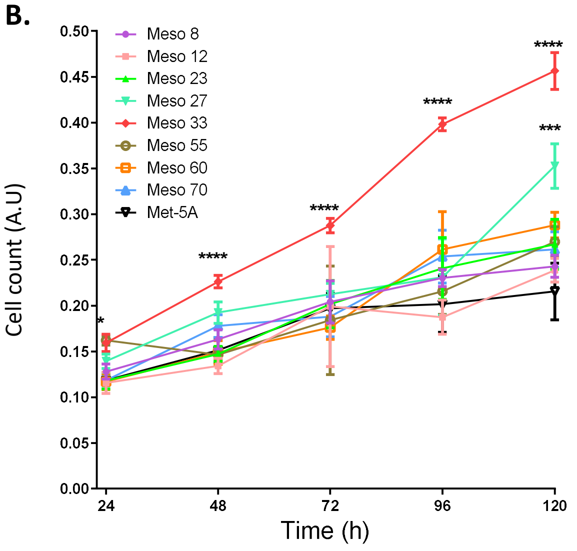

C.

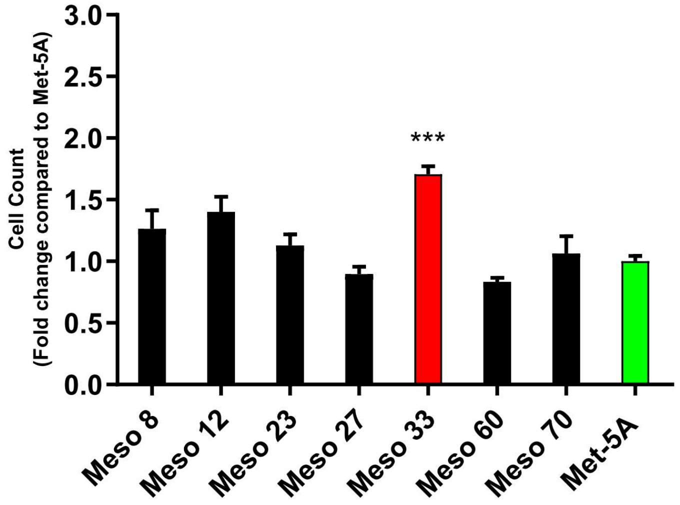

Supp Figure 1: (A) Oncoplot showing sub-type and genomic alterations for the patient-derived cell lines used in our study. **(B-C)** The indicated mesothelioma cell lines or mesothelial cells (Met-5A) were cultured in 2D (A) or 3D (B-D). **(B)** Cell growth was determined by Crystal Violet staining. **(C)** Microsphere growth at Day 10 was determined using the Cell Titer Glo assay with results normalised to those for Met-5A. Statistics: two-way ANOVA with multiple comparison (*; p<0.05, ***; p<0.005, ****; p<0.001). Data represent mean ± SD of 3 biological replicates.

Supp Fig 2

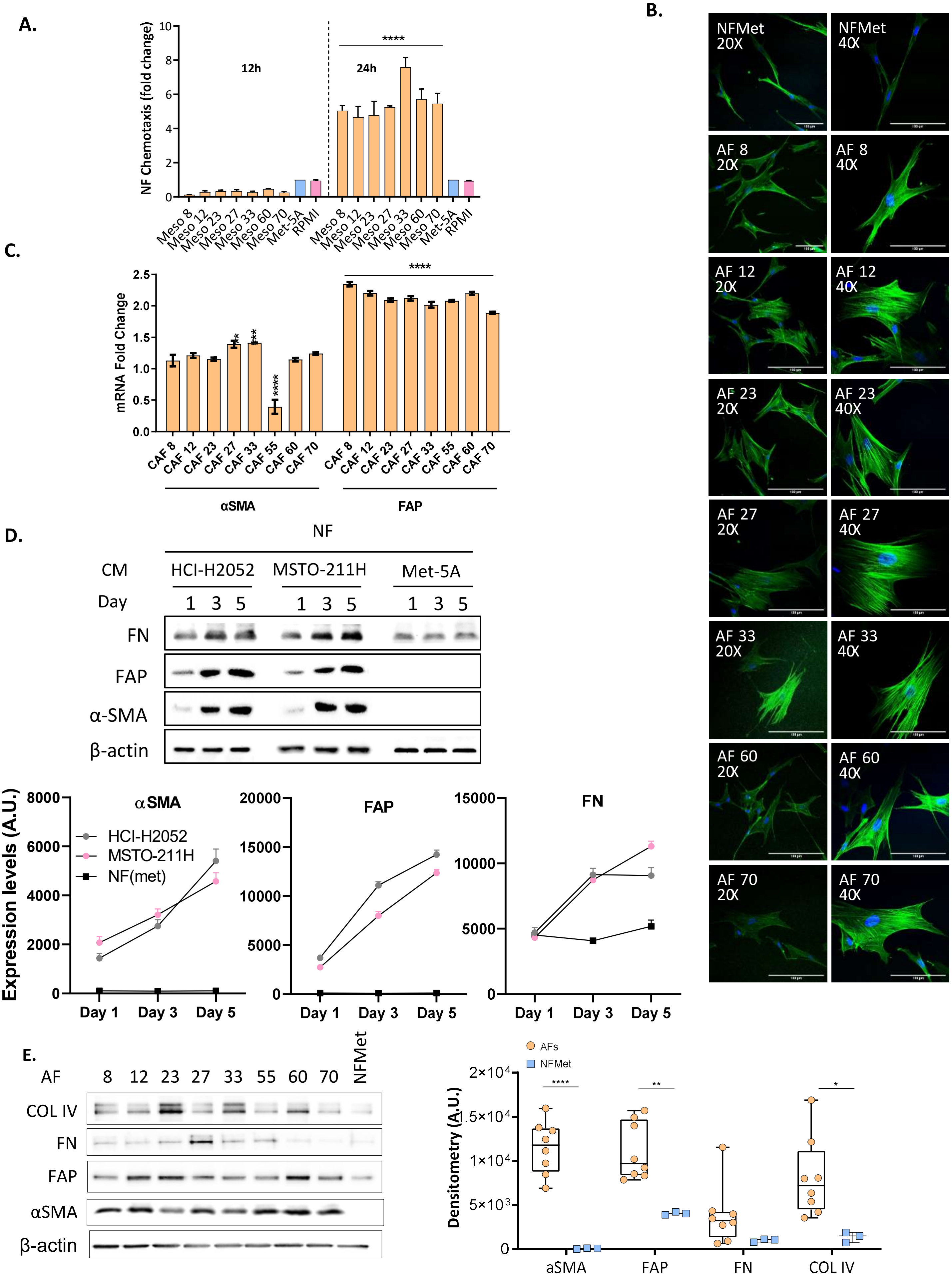
A.
NF Chemotaxis (fold change)
12h
24h
****
Meso 8
Meso 12
Meso 23
Meso 27
Meso 33
Meso 60
Meso 70
Met-5A
RPMI
B.
NFMet 20X
NFMet 40X
AF 8 20X
AF 8 40X
AF 12 20X
AF 12 40X
AF 23 20X
AF 23 40X
AF 27 20X
AF 27 40X
AF 33 20X
AF 33 40X
AF 60 20X
AF 60 40X
AF 70 20X
AF 70 40X
C.
mRNA Fold Change
CAF 8
CAF 12
CAF 23
CAF 27
CAF 33
CAF 55
CAF 60
CAF 70
αSMA
FAP
D.
NF
CM
HCI-H2052
MSTO-211H
Met-5A
Day
1 3 5
FN
FAP
α-SMA
β-actin
Expression levels (A.U.)
αSMA
FAP
FN
HCI-H2052
MSTO-211H
NF(met)
Day 1
Day 3
Day 5
E.
AF
8 12 23 27 33 55 60 70 NFMet
COL IV
FN
FAP
αSMA
β-actin
AFs
NFMet
Densitometry (A.U.)
aSMA
FAP
FN
COL IV

Supp Fig 2: (A) Fold change in the number of fibroblasts migrating towards secretions from the indicated cell type at two different timepoints. Non-conditioned medium (RPMI) was used a control. Data represent the mean ± SD of 3 biological replicates normalised to results obtained for Met-5A conditions. **(B)** Representative confocal microscopy images of fibroblasts treated for 72 h with the conditioned media of the indicated cell lines (see number). NFMet; naïve fibroblasts treated with secretions from Met-5A cells. AF; activated fibroblasts. F-actin was stained with Alexa488-Phalloidin (green) and nuclear DNA with Hoechst (scale bar: 100µm). Images representative of 3 biological replicates. **(C)** 72h post seeding, RNA was extracted from the indicated AFs and the expression levels of αSMA and FAP determined by RT-qPCR. Results were normalised to those obtained for fibroblast exposed to conditioned medium from Met-5A cells. Data represent mean fold changes ± SEM of 3 independent experiments. **(D)** Fibroblasts were exposed to the conditioned medium (CM) of the indicated cell lines and proteins were extracted at various time points prior to analysis by SDS-PAGE/Western blotting. Lower panel: quantification over 3 biological replicates for the indicated protein normalised for β-actin used as loading control. **(E)** Following 72h of treatment with CM from MPM cell lines (see number) or Met-5A cells (Met), AFs or NFMets were collected, washed, and passaged twice in non-conditioned medium before protein extracts were analysed by Western-blotting. Right panel: quantification for the indicated protein normalised for β-actin used as loading control. Statistics: Two-way ANOVA with multiple comparisons (**P<0.01, ***P<0.005, ****P<0.001).

Supp Fig 3

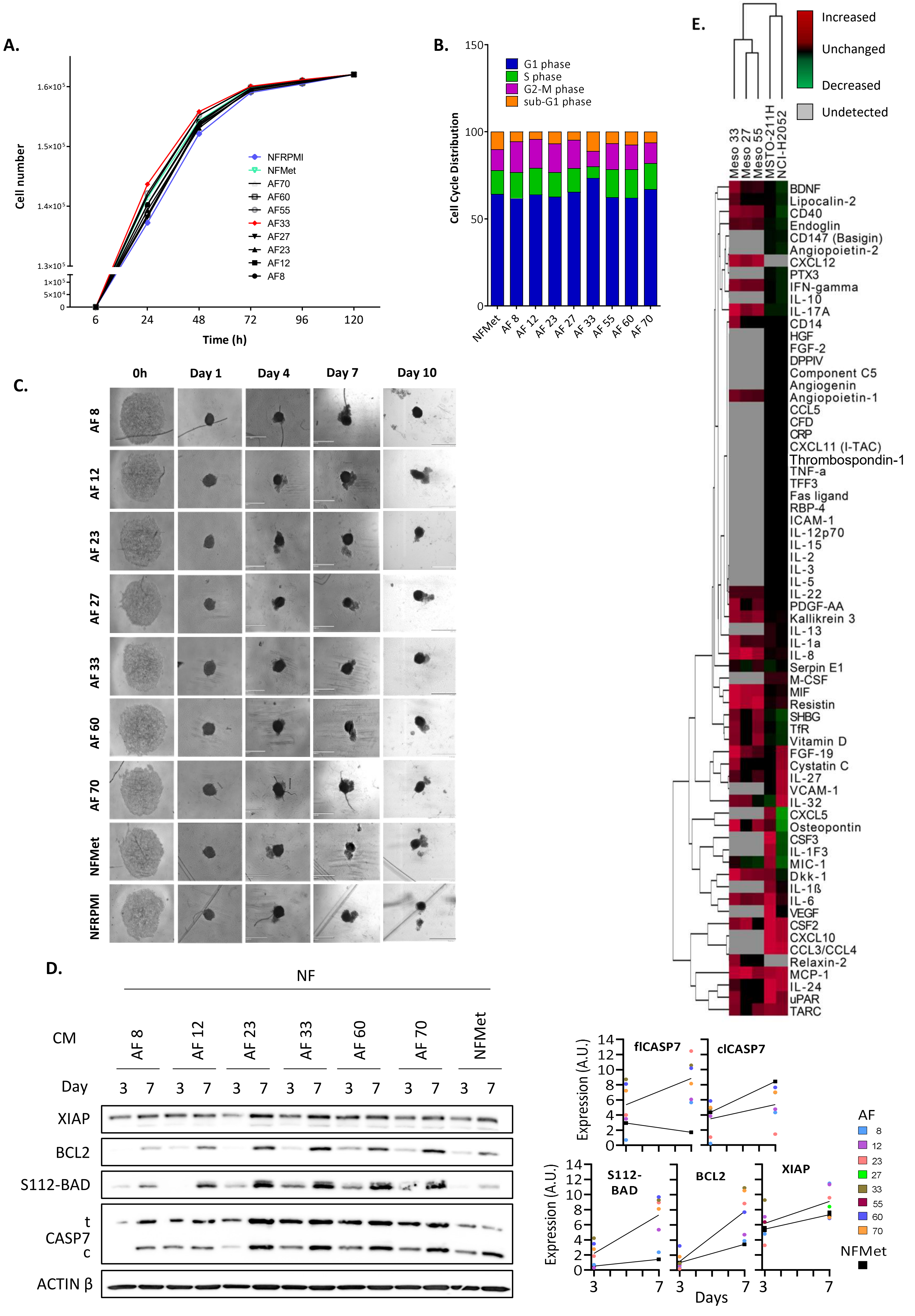
A.
Cell number
1.6×10⁵
1.5×10⁵
1.4×10⁵
1.3×10⁵
1×10⁵
5×10⁴
0
6
24
48
72
96
120
Time (h)
NFRPMI
NFMet
AF70
AF60
AF55
AF33
AF27
AF23
AF12
AF8
B.
Cell Cycle Distribution
150
100
50
0
G1 phase
S phase
G2-M phase
sub-G1 phase
NFMet
AF 8
AF 12
AF 23
AF 27
AF 33
AF 55
AF 60
AF 70
C.
0h
Day 1
Day 4
Day 7
Day 10
AF 8
AF 12
AF 23
AF 27
AF 33
AF 60
AF 70
NFMet
NFRPMI
D.
NF
CM
AF 8
AF 12
AF 23
AF 33
AF 60
AF 70
NFMet
Day
3 7 3 7 3 7 3 7 3 7 3 7 3 7
XIAP
BCL2
S112-BAD
t
CASP7
c
ACTIN β
E.
Increased
Unchanged
Decreased
Undetected
Meso 33
Meso 27
Meso 55
MSTO-211H
NCI-H2052
BDNF
Lipocalin-2
CD40
Endoglin
CD147 (Basigin)
Angiopoietin-2
CXCL12
PTX3
IFN-gamma
IL-10
IL-17A
CD14
HGF
FGF-2
DPPIV
Component C5
Angiogenin
Angiopoietin-1
CCL5
CFD
CRP
CXCL11 (I-TAC)
Thrombospondin-1
TNF-a
TFF3
Fas ligand
RBP-4
ICAM-1
IL-12p70
IL-15
IL-2
IL-3
IL-5
IL-22
PDGF-AA
Kallikrein 3
IL-13
IL-1a
IL-8
Serpin E1
M-CSF
MIF
Resistin
SHBG
TfR
Vitamin D
FGF-19
Cystatin C
IL-27
VCAM-1
IL-32
CXCL5
Osteopontin
CSF3
IL-1F3
MIC-1
Dkk-1
IL-1ß
IL-6
VEGF
CSF2
CXCL10
CCL3/CCL4
Relaxin-2
MCP-1
IL-24
uPAR
TARC
Expression (A.U.)
14
12
10
8
6
4
2
0
flCASP7
clCASP7
S112-BAD
BCL2
XIAP
3
7
Days
AF
8
12
23
27
33
55
60
70
NFMet

Supp Figure 3: (A) The proliferation rate in 2D of AFs and naïve fibroblasts (NF) treated with conditioned medium from Met-5A cells (Met) or unconditioned complete medium (RPMI) at 72h was assessed by flow cytometry analysis following CFSE staining. Data are the mean ± SEM from 3 individual biological replicates. **(B)** The cell cycle distribution of AFs and NFMets was determined following PI staining and flow cytometry acquisition. Data are representative of 3 individual experiments. **(C)** The growth rate of 3D microspheres of MRC-5 fibroblasts treated with indicated conditioned medium or complete medium (RPMI) was monitored by microscopy. **(D)** Immunoblot for the expression of apoptosis regulators XIAP, BCL-2, phosphorylated BAD (Ser 112) and total (t) and cleaved (c) Caspase 7. Right panel: quantification over 3 biological replicates for the indicated protein normalised for β-actin used as loading control. **(E)** Comparison of cytokine expression profiling for fibroblasts activated for 72h using condition media of three of our patient-derived mesothelioma cell lines (Meso) and two commonly used commercially-available mesothelioma cell lines. Hierarchical clustering was performed using ClusterMaker under Cytoscape.

Supp Fig 4

A.

GeneSet	Count	P-value	FDR
extracellular matrix organization	40	1.11E-16	5.38E-14
collagen fibril organization	14	1.11E-16	5.38E-14
cell adhesion	23	1.23E-13	3.96E-11
endodermal cell differentiation	7	3.03E-09	4.88E-07
proteolysis	14	1.17E-07	1.41E-05
cellular response to amino acid stimulus	7	1.34E-07	1.43E-05
extracellular matrix disassembly	7	7.88E-07	7.64E-05
angiogenesis	11	1.36E-06	1.20E-04
positive regulation of cell-substrate adhesion	5	2.55E-06	1.74E-04
dermatan sulfate biosynthetic process	4	2.72E-06	1.74E-04
sequestering of TGFbeta in extracellular matrix	3	4.69E-06	2.49E-04
negative regulation of angiogenesis	6	4.70E-05	1.52E-03
chondroitin sulfate biosynthetic process	4	4.75E-05	1.52E-03
positive regulation of cell migration	9	5.16E-05	1.56E-03
collagen biosynthetic process	3	5.20E-05	1.56E-03
response to mechanical stimulus	5	6.92E-05	1.94E-03
cellular response to transforming growth factor beta stimulus	5	6.92E-05	1.94E-03
collagen-activated tyrosine kinase receptor signaling pathway	3	9.39E-05	2.53E-03
integrin-mediated signaling pathway	6	9.89E-05	2.57E-03
negative regulation of endopeptidase activity	6	1.11E-04	2.79E-03
negative regulation of endodermal cell differentiation	2	1.17E-04	2.79E-03
basement membrane organization	3	1.53E-04	3.22E-03
supramolecular fiber organization	3	1.53E-04	3.22E-03
glycosaminoglycan biosynthetic process	4	1.72E-04	3.61E-03
cell migration	8	1.99E-04	3.99E-03
epithelial to mesenchymal transition	4	2.35E-04	4.46E-03
negative regulation of metallopeptidase activity	2	2.63E-04	4.73E-03
collagen catabolic process	4	3.42E-04	5.81E-03
negative regulation of cell adhesion	4	3.73E-04	6.35E-03
positive regulation of cell division	4	4.43E-04	6.97E-03
leukocyte chemotaxis involved in inflammatory response	2	4.65E-04	6.97E-03
response to drug	7	5.12E-04	7.68E-03
response to cytokine	4	6.08E-04	9.12E-03
cell-matrix adhesion	5	6.47E-04	9.70E-03

B.

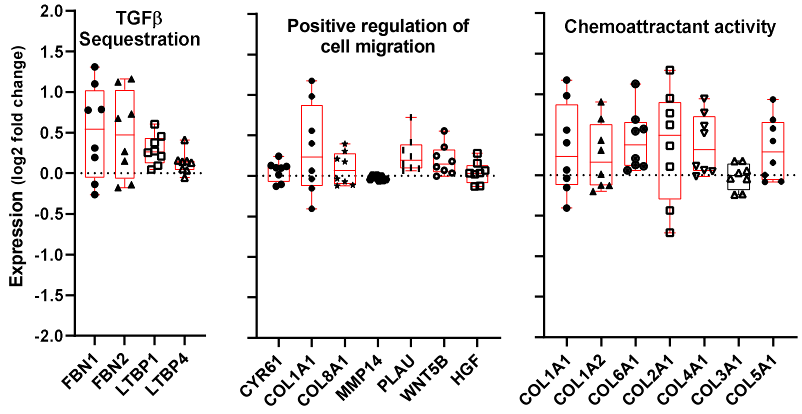

C.

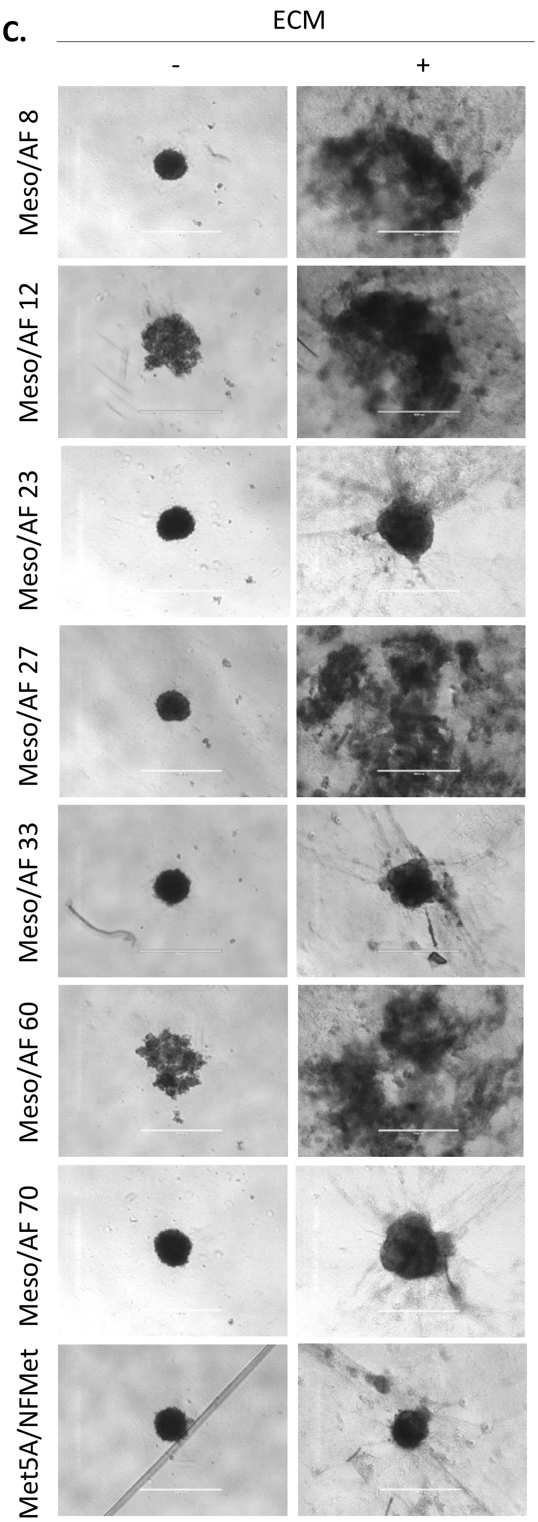

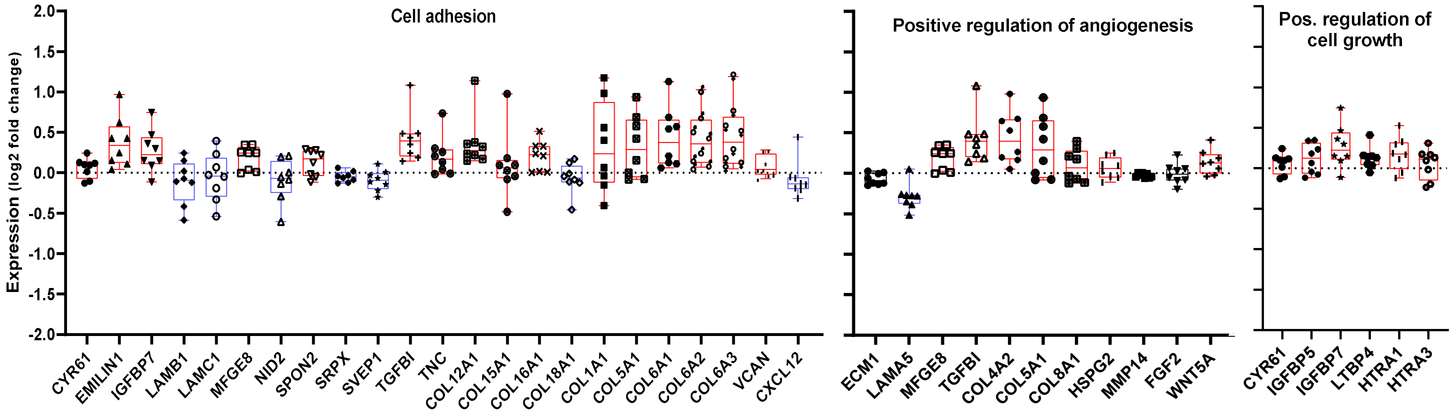

Supp Figure 4: (A) Gene ontology analysis for biological processes enriched in the functional interaction network shown in Fig 4B, performed in ReactomeFI under Cytoscape. Only gene sets with FDR<0.001 are shown. **(B)** Expression of individual genes within the genesets from selected biological processes in (A) are shown. Dots represent individual ECM samples analysed and horizontal bar the median. Red boxes indicate conditions with median over and blue boxes samples with median under log2 fold change of 0. **(C)** Images of MPM/AF co-culture spheroids grown in the presence or absence of ECM derived from the corresponding AF cell line at 72h post seeding (4X magnification). Images are representative of 3 independent biological replicates.

Supp Fig 5

Target	Provider	Catalog #
Aurora A/B/C P Thr288/Thr232/Thr198	Cell Signaling Technologies	2914
Axl (C2B12)	Cell Signaling Technologies	4939
Bad P Ser112	Cell Signaling Technologies	9291
Bad P Ser136	Cell Signaling Technologies	9295
Bak	Epitomics	1542-1
Bax	Epitomics	1063
Bcl-2	Epitomics	1017-1
beta-actin	Cell Signaling Technologies	4970
beta-Catenin	Cell Signaling Technologies	9562
beta-Catenin P Ser33,Ser37,Thr41	Cell Signaling Technologies	9561
beta-Catenin P Thr41,Ser45	Cell Signaling Technologies	9565
Bid	Abcam/Epitomics	ab32060
Bim	Epitomics	1036
c-Abl P Y245	Cell Signaling Technologies	2868
c-Abl P Y412 (247C7)	Cell Signaling Technologies	2865
c-Abl	Cell Signaling Technologies	2862
c-Jun P Ser73	Cell Signaling Technologies	9164
c-Myc	Cell Signaling Technologies	5605
EGFR (D38B1) XP ®	Cell Signaling Technologies	4267
EGFR P Tyr1086		369700
EGFR P Tyr1173	Cell Signaling Technologies	4407
EGFR P Y992	Cell Signaling Technologies	2235
ErbB-1/EGFR	Cell Signaling Technologies	2232
ErbB-2/Her2/EGFR P Tyr1248/Tyr1173	Cell Signaling Technologies	2244
ErbB-3/Her3/EGFR	Cell Signaling Technologies	4754
ErbB-3/Her3/EGFR P Tyr1289	Cell Signaling Technologies	4791
FAK1	Cell Signaling Technologies	3285
FAK1 P Y397	Cell Signaling Technologies	3283
FGF19 EPR8407(2)]	abcam	ab154185
Fibronectin [F14]	Abcam	ab45688
FLT3 P Tyr591 P Tyr591	Cell Signaling Technologies	3461
Fox01 P	Cell Signaling Technologies	9461
FOXA1	Millipore (Upstate)	AB4124
FOXO1 (C29H4)	Cell Signaling Technologies	2880
FOXO3a (75D8)	Cell Signaling Technologies	2497
FRA1 (R20)	Santa Cruz	sc-605
GATA3 (D13C9)	Cell Signaling Technologies	5852
GSK-3-alpha/beta P Ser21/Ser9	Cell Signaling Technologies	9331
HDAC4/5/6/7 P	Cell Signaling Technologies	3443
Hexokinase II	Cell Signaling Technologies	2867
IGF-1R beta	Cell Signaling Technologies	3027
IGF-1R beta P Tyr1162,Tyr1163	Invitrogen (Biosource)	44-804G
IkB-alpha	Cell Signaling Technologies	4812
IkB-alpha P Ser32	Cell Signaling Technologies	2859
IKK alpha/beta P Ser176/Ser177	Cell Signaling Technologies	2078
Integrin alpha 4	Cell Signaling Technologies	4600
Integrin Beta 1 [EP1041Y]	abcam	ab52971
Integrin beta3	Cell Signaling Technologies	4702
Integrin beta4	Cell Signaling Technologies	4707
JAK1	Cell Signaling Technologies	3332
Lamin A/C	Cell Signaling Technologies	2032
M-CSF P Tyr723	Cell Signaling Technologies	3155
MEK 1/2 P Ser217/221	Cell Signaling Technologies	9121
MEK1/2	Cell Signaling Technologies	9122
Met	Cell Signaling Technologies	4560
Met P Tyr1234	Signalway	11227-1
Met P Tyr1349	Signalway	11238
MSK1 P Ser376	Cell Signaling Technologies	9591
mTOR	Cell Signaling Technologies	2972
mTOR P Ser2448	Cell Signaling Technologies	2971
mTOR P Ser2481	Millipore (Upstate)	09-343SP
NFkB p105/p50	GeneTex	GTX110585
NFkB p65 Ser536	Cell Signaling Technologies	3033
p21 CIP/WAF1	Cell Signaling Technologies	2946
p38 MAPK	Cell Signaling Technologies	9212
p38 MAPK PThr180,Tyr182	Cell Signaling Technologies	9211
p44/42 MAPK (ERK1/2)	Cell Signaling Technologies	9102
p44/42 MAPK (ERK1/2) P Thr202/Thr185,Tyr204/Tyr187	Cell Signaling Technologies	4370
p70 S6 Kinase	Cell Signaling Technologies	9202
p70 S6 Kinase P Thr389	Cell Signaling Technologies	9205S
p70 S6 Kinase P Thr421,Ser424	Cell Signaling Technologies	9204
p90 S6 kinase (Rsk1-3)	Santa Cruz	sc-231
p90 S6 kinase (Rsk1-3) P Thr359,Ser363	Cell Signaling Technologies	9344
PARP cleaved Asp214	Cell Signaling Technologies	9541
PDGFR P Tyr751	Cell Signaling Technologies	4549
PDGFR P Tyr1021	Cell Signaling Technologies	2227
PKA	Abcam	ab26322
PKA RII P Ser96	Epitomics	1151-1
PKA substrate P (RRXS/T) (100G7E)	Cell Signaling Technologies	9624
PKC (pan) P Ser660 (beta-2)	Cell Signaling Technologies	9371
PKC substrate P (R/K)X(S*)(Hyd)(R/k)	Cell Signaling Technologies	2261
PKC-alpha P Thr638	Abcam	ab32502
PKC-gamma P Thr514	GeneTex	GTX25778
PKC-zeta	Cell Signaling Technologies	9372
PKC-zeta/lambda P Thr410/403	Cell Signaling Technologies	9378
Puma	Cell Signaling Technologies	4976
PYK2 [EP206Y]	abcam	ab81266
S6 Ribosomal Protein	Cell Signaling Technologies	2217
S6 Ribosomal protein P Ser235,Ser236	Cell Signaling Technologies	2211
S6 Ribosomal protein p Ser240,Ser244	Cell Signaling Technologies	2215
Smad1 (D59D7)	Cell Signaling Technologies	6944
Smad1/5 P Ser463/Ser465	Cell Signaling Technologies	9516
Smad2 (C86F7)	Cell Signaling Technologies	3122
Smad2/3 P Ser465/Ser423,Ser467/Ser425	Cell Signaling Technologies	8828
Smad3 P Ser423,Ser425	Cell Signaling Technologies	9520
Src	Cell Signaling Technologies	2109
Src (family) P Tyr416	Cell Signaling Technologies	2101
SRF (D71A9)	Cell Signaling Technologies	5147
Stat1 P Ser727	Invitrogen (Biosource)	44-382G
Stat1 P Tyr701	Cell Signaling Technologies	9171
Stat3 P Ser727	Cell Signaling Technologies	9134
Stat3 P Tyr705	Cell Signaling Technologies	9138
Stat5	Invitrogen (Biosource)	44-368G
Stat5 P Tyr694	Cell Signaling Technologies	9351
Stat6 P Tyr641	Cell Signaling Technologies	9361
TGF beta (56E4)	Cell Signaling Technologies	3709
Tyk2 P Tyr1054,Tyr1055	Cell Signaling Technologies	9321
VEGFR P Tyr1059	Cell Signaling Technologies	3817
VEGFR P Tyr1175	Cell Signaling Technologies	2478
YAP P Ser127	Cell Signaling Technologies	4911
YAP1 [EP1674Y]	Abcam	ab52771

Supp Figure 5: Details of antibodies used in our RPPA.

Supp Fig 6

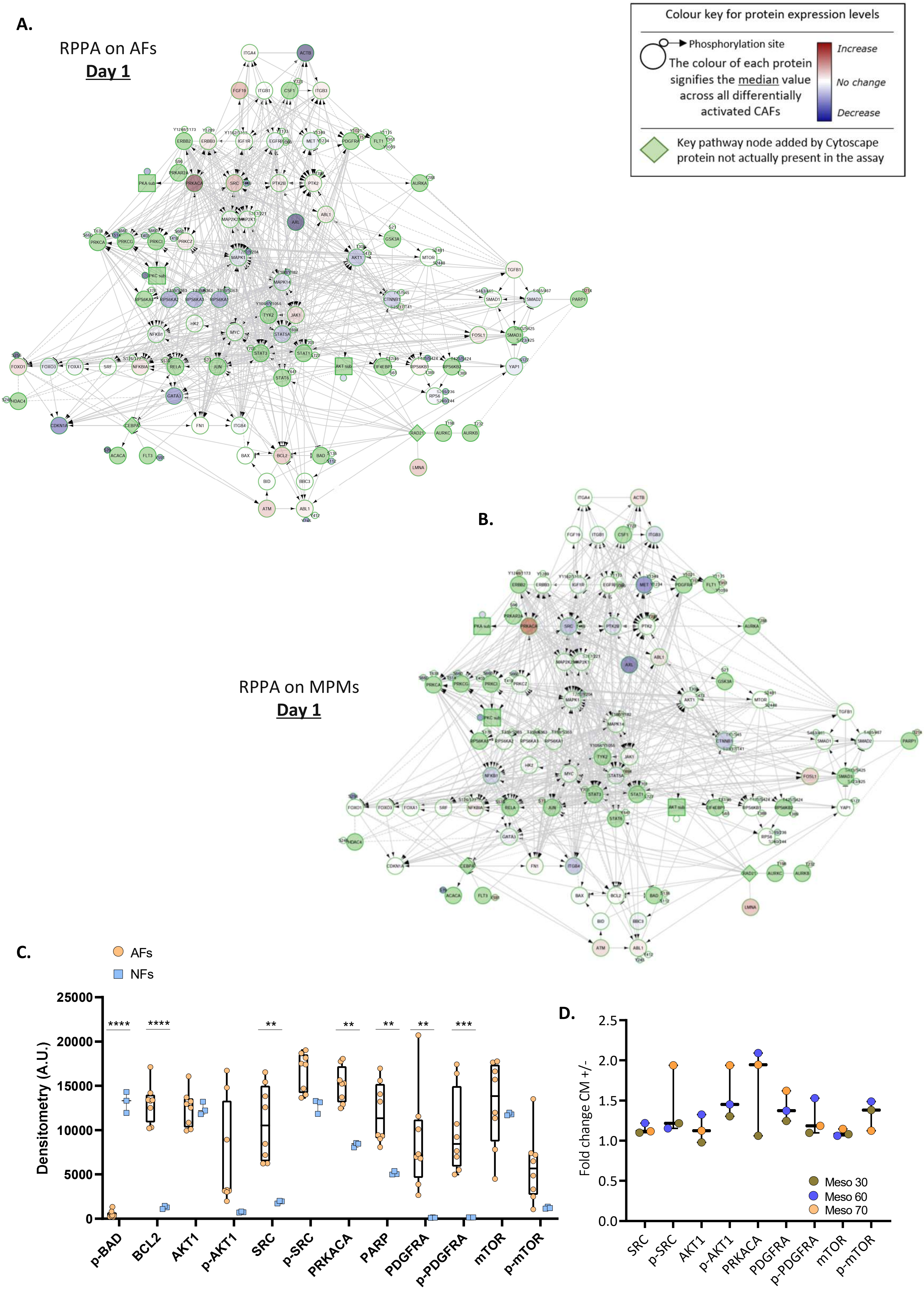
A.
RPPA on AFs
Day 1
Colour key for protein expression levels
Phosphorylation site
The colour of each protein signifies the median value across all differentially activated CAFs
Increase
No change
Decrease
Key pathway node added by Cytoscape protein not actually present in the assay
B.
RPPA on MPMs
Day 1
C.
AFs
NFs
Densitometry (A.U.)
25000
20000
15000
10000
5000
0
****
****
**
**
**
**
***
p-BAD
BCL2
AKT1
p-AKT1
SRC
p-SRC
PRKACA
PARP
PDGFRA
p-PDGFRA
mTOR
p-mTOR
D.
Fold change CM +/-
2.5
2.0
1.5
1.0
0.5
0.0
Meso 30
Meso 60
Meso 70
SRC
p-SRC
AKT1
p-AKT1
PRKACA
PDGFRA
p-PDGFRA
mTOR
p-mTOR

Supp Figure 6: (A-B) Lysates from NFs incubated for 24h with CM from MPM cells (A) or from MPM cells treated for 24h with CM of the corresponding AFs (B) were analysed by RPPA. Log2 fold changes in protein expression or post-translational modification over Met5A's CM-treated NF (A) or NF's CM-treated MPM cells (B) were averaged over all cell lines and data imported into Cytoscape to build a functional interaction network using the Reactome FI plugin. (C-D) Quantification of blots shown in Fig 5C-D. Dots represent the average over 3 biological replicates of cell types shown in corresponding Western blots. Signal for the indicated protein was normalised to that of VCL (C) or Actin B (D) used as loading control. Statistics: Two-way ANOVA (**P<0.01, ***P<0.005, ****P<0.001).

Supp Fig 7

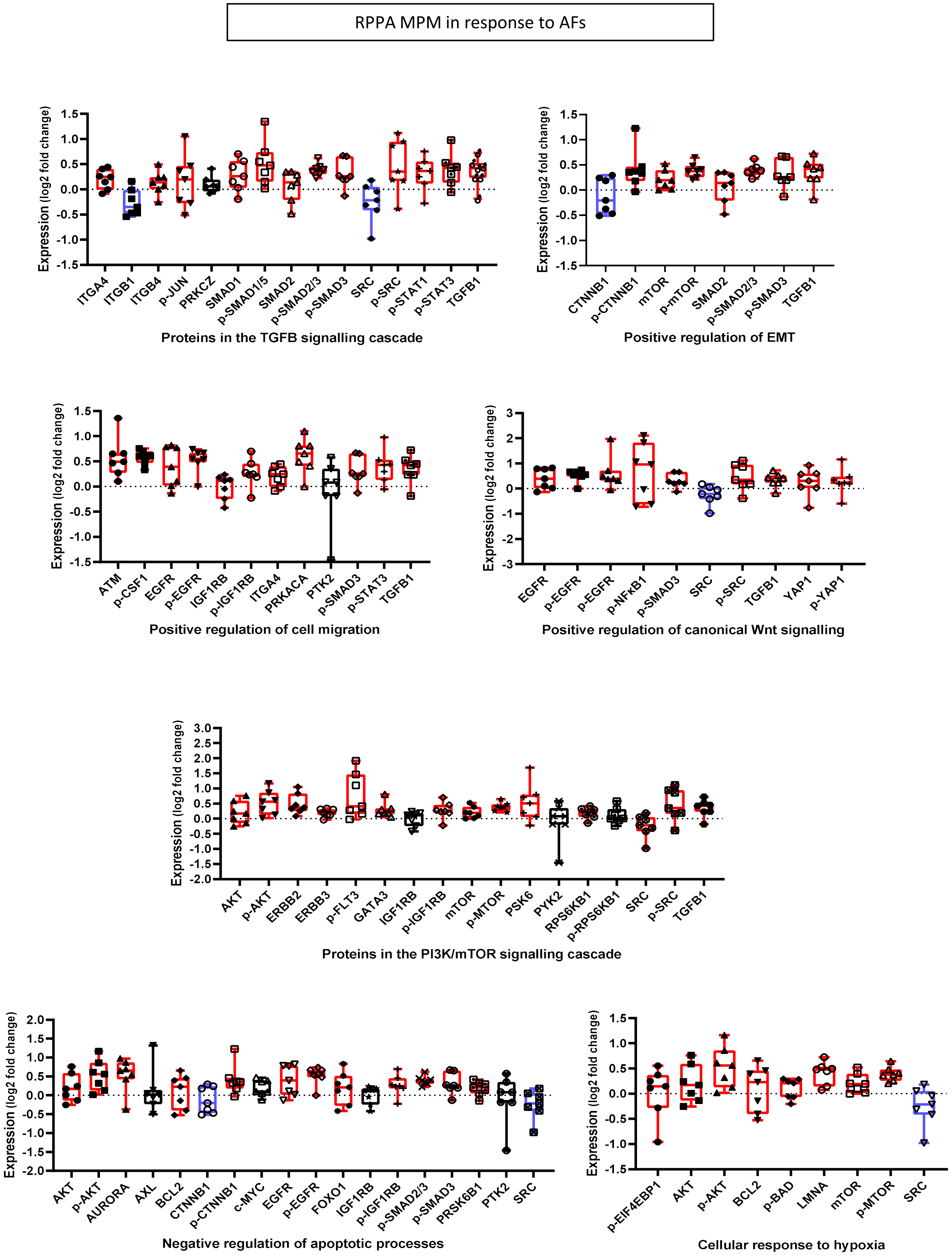

Supp Fig 7: Box plots show log2 fold change for genes in enriched biological processes gene sets revealed by gene ontology analysis of the network shown in Fig 5A using Reactome FI under cytoscape. Only processes with FDR<0.001 are shown. Dots represent individual samples analysed and horizontal bar is the median. Red boxes indicate conditions with median over and blue boxes samples with median under log2 fold change of 0.

Supp Fig 8

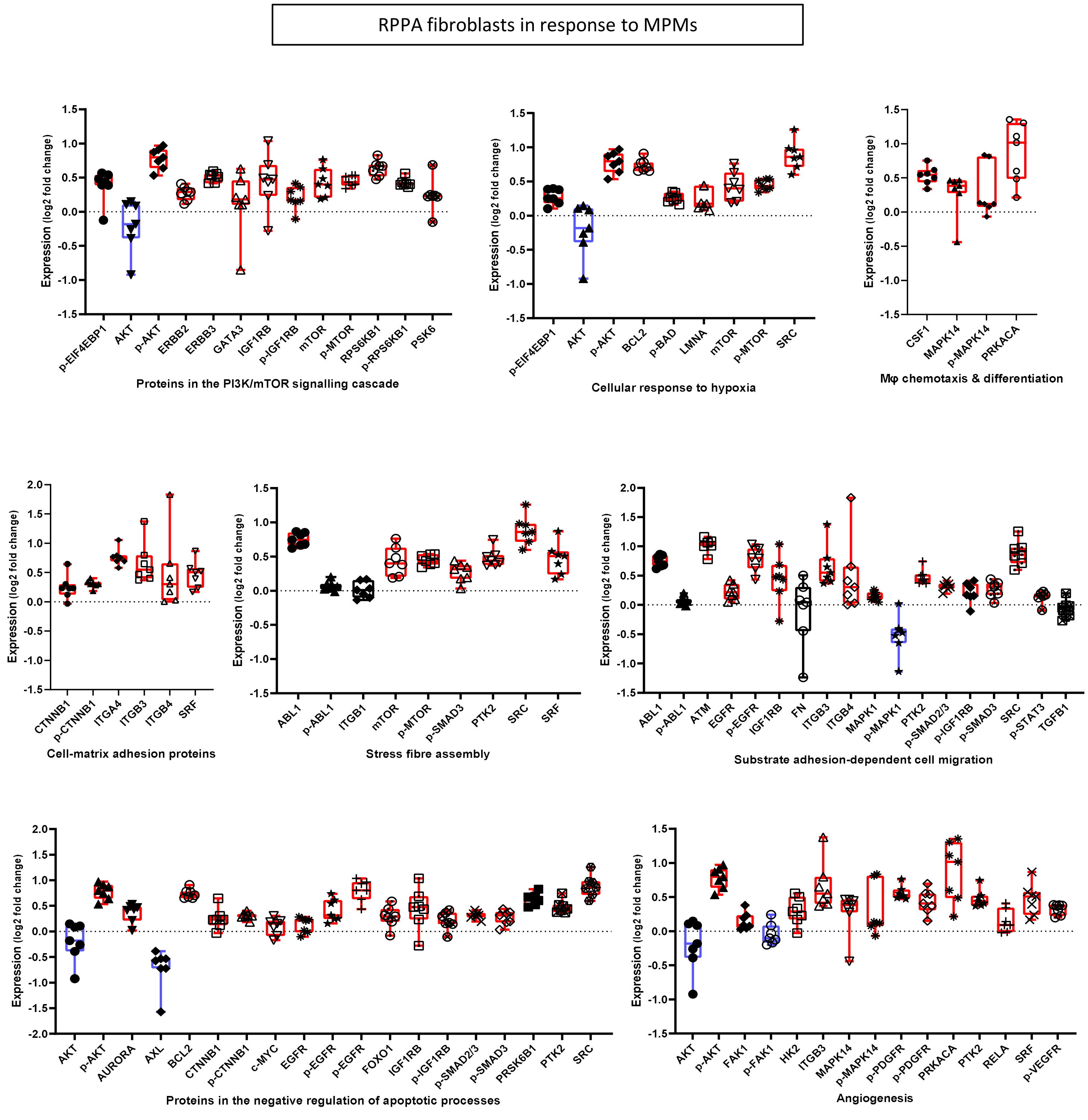

Supp Fig 8: Box plots show log2 fold change for genes in enriched biological processes gene sets revealed by gene ontology analysis of the network shown in Fig 5B using Reactome FI under cytoscape. Only processes with FDR<0.001 are shown. Dots represent individual samples analysed and horizontal bar is the median. Red boxes indicate conditions with median over and blue boxes samples with median under log2 fold change of 0.

Supp Fig 9

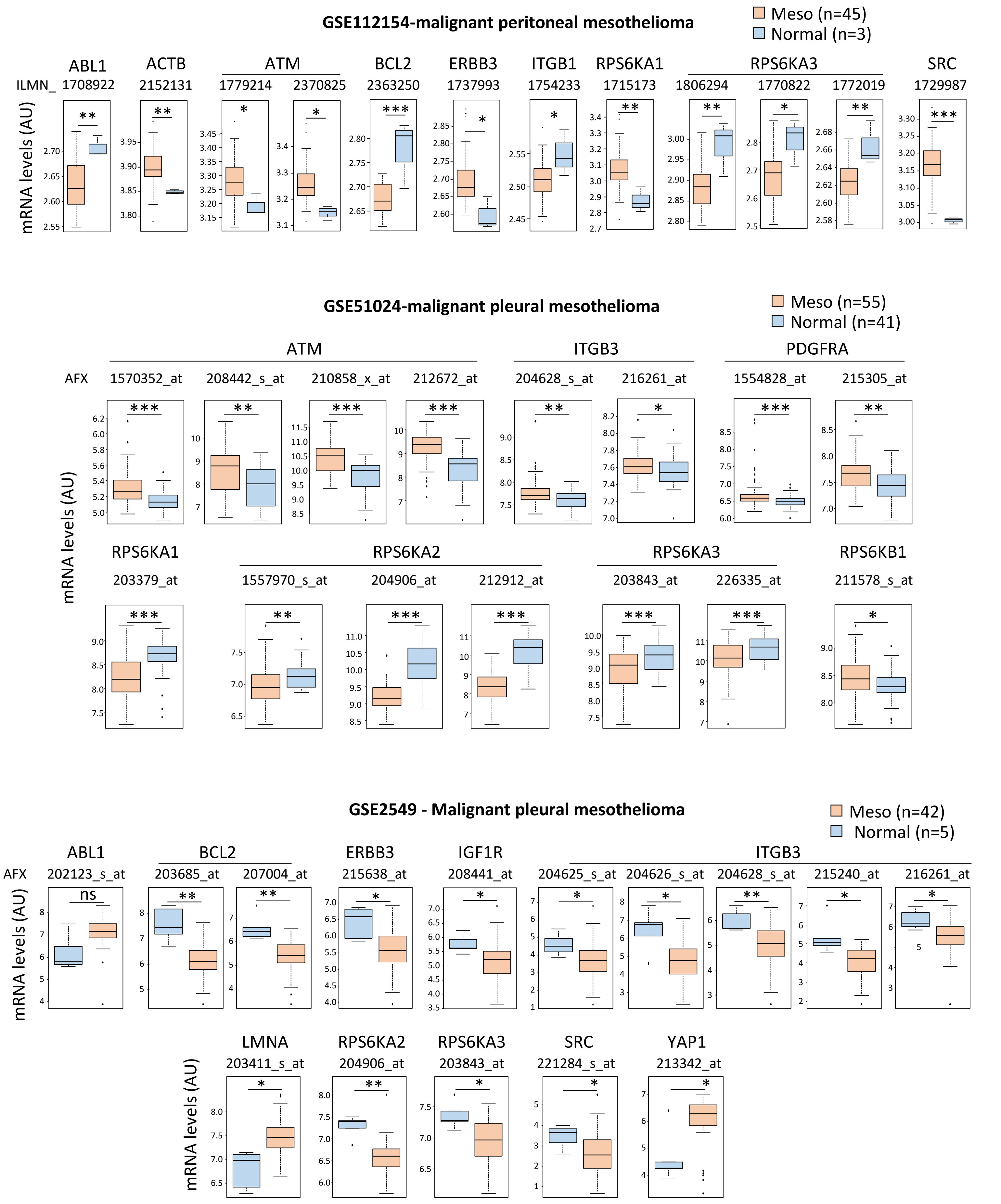

Supp Fig 9: Public microarray datasets from the GEO database were analysed for changes in expression of the genes corresponding to the proteins found modulated in the network shown in Fig 5B following crosstalk between CAFs and mesothelioma cells. The datasets and associated short description, analysis platform (ILMN; Illumina, AFX; Affimetrix) and corresponding probes for the genes analysed are indicated. Statistics: t-test (*P<0.05, **P<0.01, ***P<0.005).

Drug Name	Intended targets [Secondary targets]	MPM trial
**Afuresertib**	AKT1, AKT2, AKT3	-
**Blu9931**	FGFR4, [FGFR3]	-
**Cediranib**	PDGFRA, PDGFRB, [VEGFR1, VEGFR2, VEGFR3, FGFR1, BCL-ABL, SRC, c-KIT, EGFR, ERB2, CSF-1R]	Phase 2
**Cisplatin**	N-heterocyclic bases on DNA	Standard-of care in combination with pemetrexed
**Dasatinib**	SRC kinases, [BCL-ABL, LYN, FYN, YES, c-KIT, PDGFRA, PDGFRB, EPHA2]	Phase 2
**Linagliptin**	DPP4, [DPP2, DPP8, DPP9]	-
**Masitinib**	PDGFRA, PDGFRB, [ABL, LYN, c-KIT]	-
**Nintedanib**	FGFR1, FGFR2, FGFR3, [LCK, VEGFR1, VEGFR2, VEGFR3, FLT-3, CSF-1R]	Phase 2
**Olaparib**	PARP1, PARP2	Phase 2
**Pemetrexed**	DHFR, [TYMS]	Standard-of care in combination with cisplatin
**Saracatinib**	SRC kinases, [PDGFRA, PDGFRB, FGFR1, BCL-ABL, LYN, FYN, YES, c-KIT, EPHA2, FGR, VEGFR1, VEFGR2, VEGFR3, EGFR]	Phase 1/2
**TRC105**	ENG	-
**Vismodegib**	SMO, [SMO, PTCH, ABCG2, PGP, MRP-1, MDK]	-
**Vistusertib**	mTORC1, mTORC2 [PI3K α/β/γ/δ, pS6 (S235/236)]	-

Supp Fig 10: Drugs selected for our experiments based on targets showing changes in expression/phosphorylation in Fig 5A-B (intended targets). The table also shows secondary targets reported for these compounds as well as their state of clinical development in mesothelioma.

Supp Fig 11

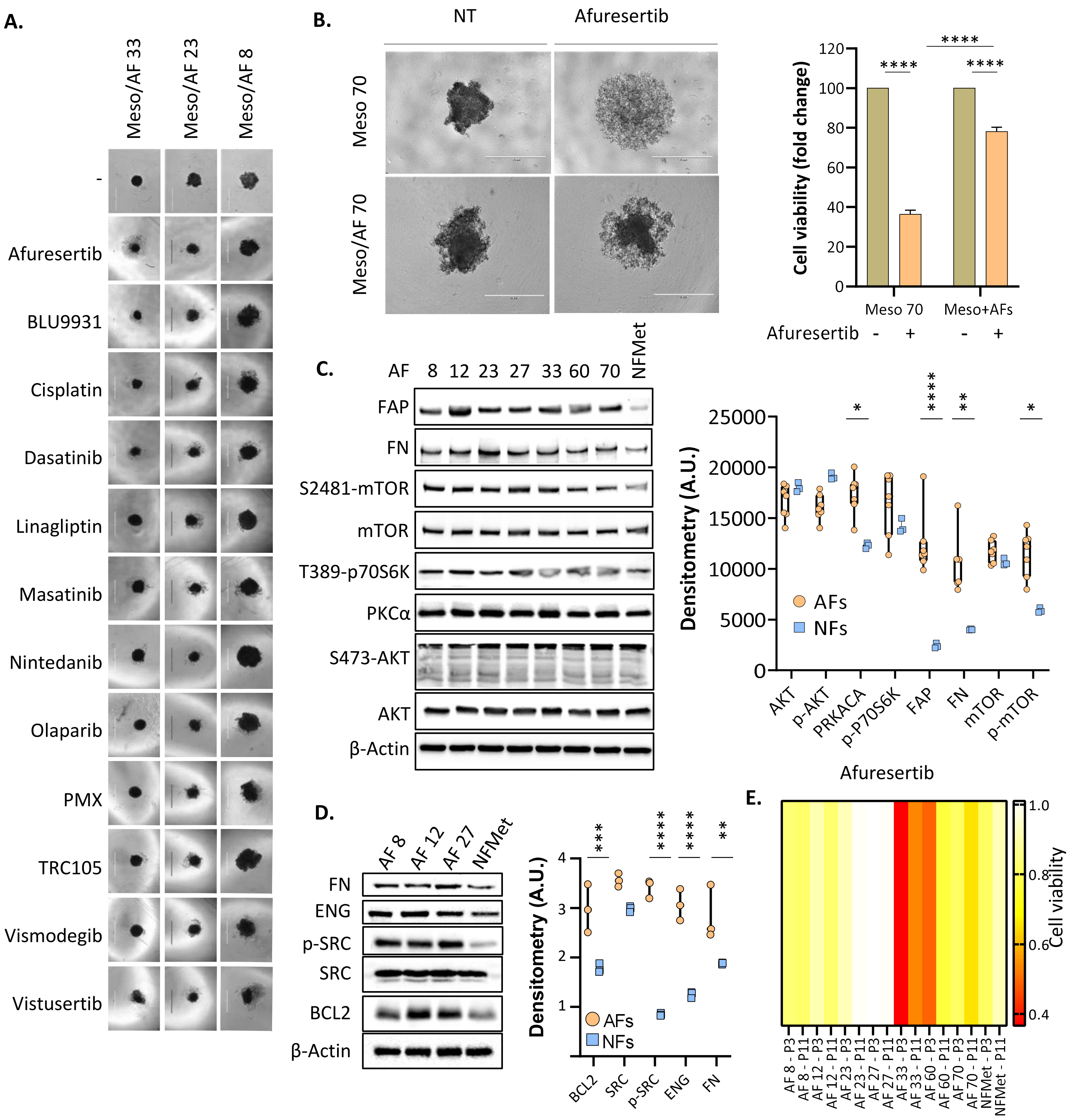

Supp Figure 11: (A) 3D microspheres composed of both AFs and MPM cells at a 1:1 ratio. Images representative of 4 independent experiments. **(B)** Comparison of cell viability measurements between Afuresertib-treated spheroids composed solely of Meso 70 cells and spheroids composed of equal number of MPM cells and AFs. Right panel; data show a representative of 4 independent biological replicates, with mean ± SEM of 3 technical replicates per condition. Statistics: two-way ANOVA with multiple comparisons (****; P<0.0001). **(C-D)** The expression/phosphorylation of targets identified in Fig 5A and C in AFs was analysed by Western blotting after 11 passages in conditioned medium from the corresponding mesothelioma cell lines (left panel). Right panels; Blots were quantified and signal for the indicated proteins normalised to that of β-Actin used a loading control. **(E)** Comparison between cell viability measurements from drug-treated AF microspheres. AFs marked with P3 underwent 3 consecutive culturing passages, while AFs marked with P11 underwent 11 consecutive culturing passages (1.25 month) in conditioned medium from the corresponding MPM cell lines. Cell viability was measured 72h post drug treatment using Cell Titer Glo and the heatmap shows fold change normalized to the non-treated (NT) microsphere for the corresponding condition.

Supp Fig 12

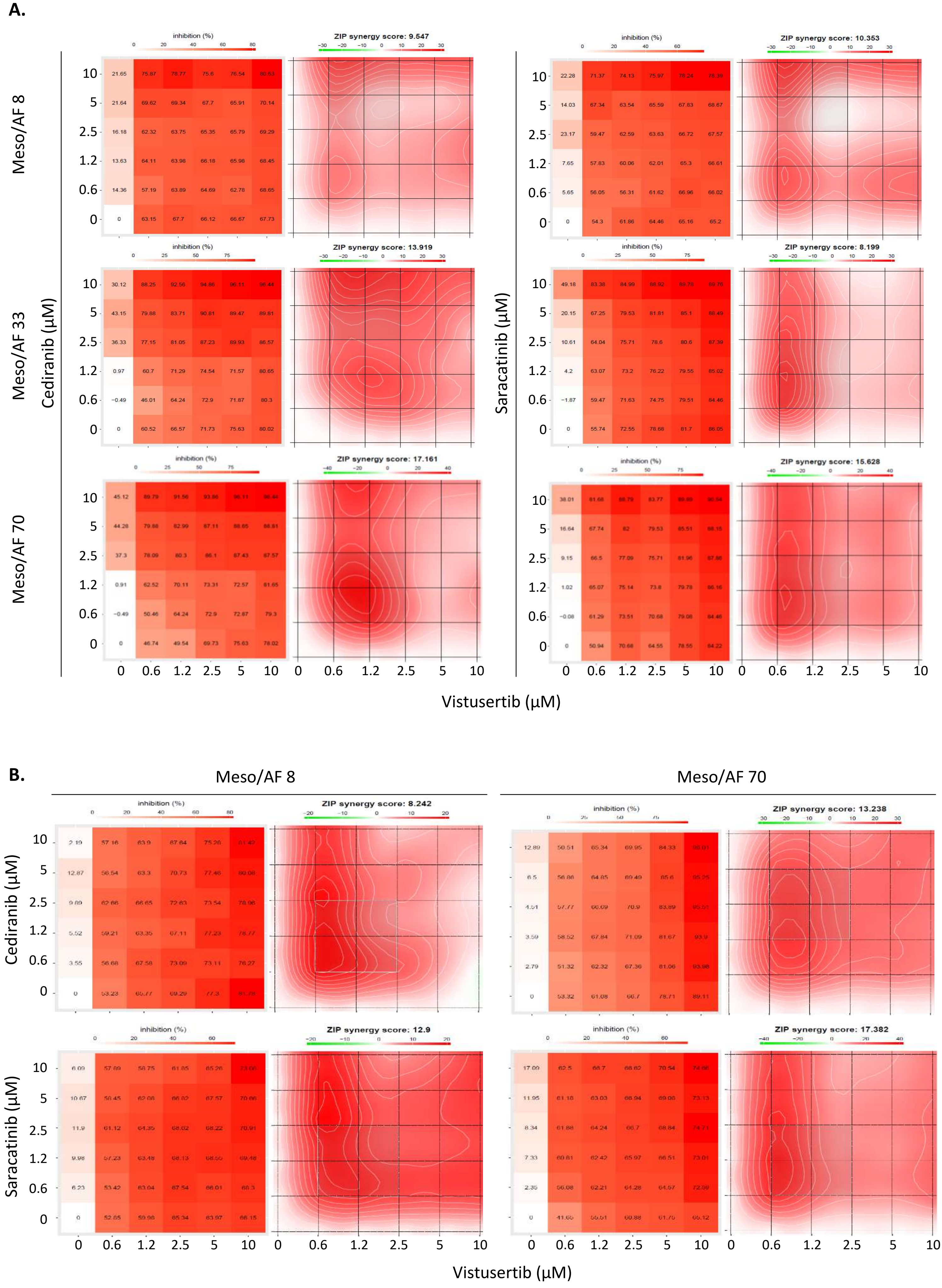

A.
Meso/AF 8
Meso/AF 33
Meso/AF 70
Cediranib (μM)
Saracatinib (μM)
inhibition (%)
ZIP synergy score: 9.547
ZIP synergy score: 10.353
ZIP synergy score: 13.919
ZIP synergy score: 8.199
ZIP synergy score: 17.161
ZIP synergy score: 15.628
Vistusertib (μM)
B.
Meso/AF 8
Meso/AF 70
Cediranib (μM)
Saracatinib (μM)
ZIP synergy score: 8.242
ZIP synergy score: 13.238
ZIP synergy score: 12.9
ZIP synergy score: 17.382
Vistusertib (μM)

Supp Fig 12: Drug synergy analysis for combinations of cediranib or saracatinib with vistusertib using SynergyFinder 2.0. MPM/AF microspheres using AFs activated for 11 passages **(A)** or 20 passages **(B)** in conditioned medium from mesothelioma cells were treated with the indicated drugs for 72h before cell viability determination using Cell Titer Glo. Left panels represent cell viability with red colour intensity inversely correlating with survival. Right panels represent drug interaction as a function of concentration. Green to red continuous colour mapping corresponds to negative to positive synergy scores, respectively, with isolines delineating areas of equivalent drug interaction. The greyed squares highlight area of maximum drug synergy. Each graph is accompanied by the averaged ZIP synergy score for the overall drug interaction.

Supp Fig 13

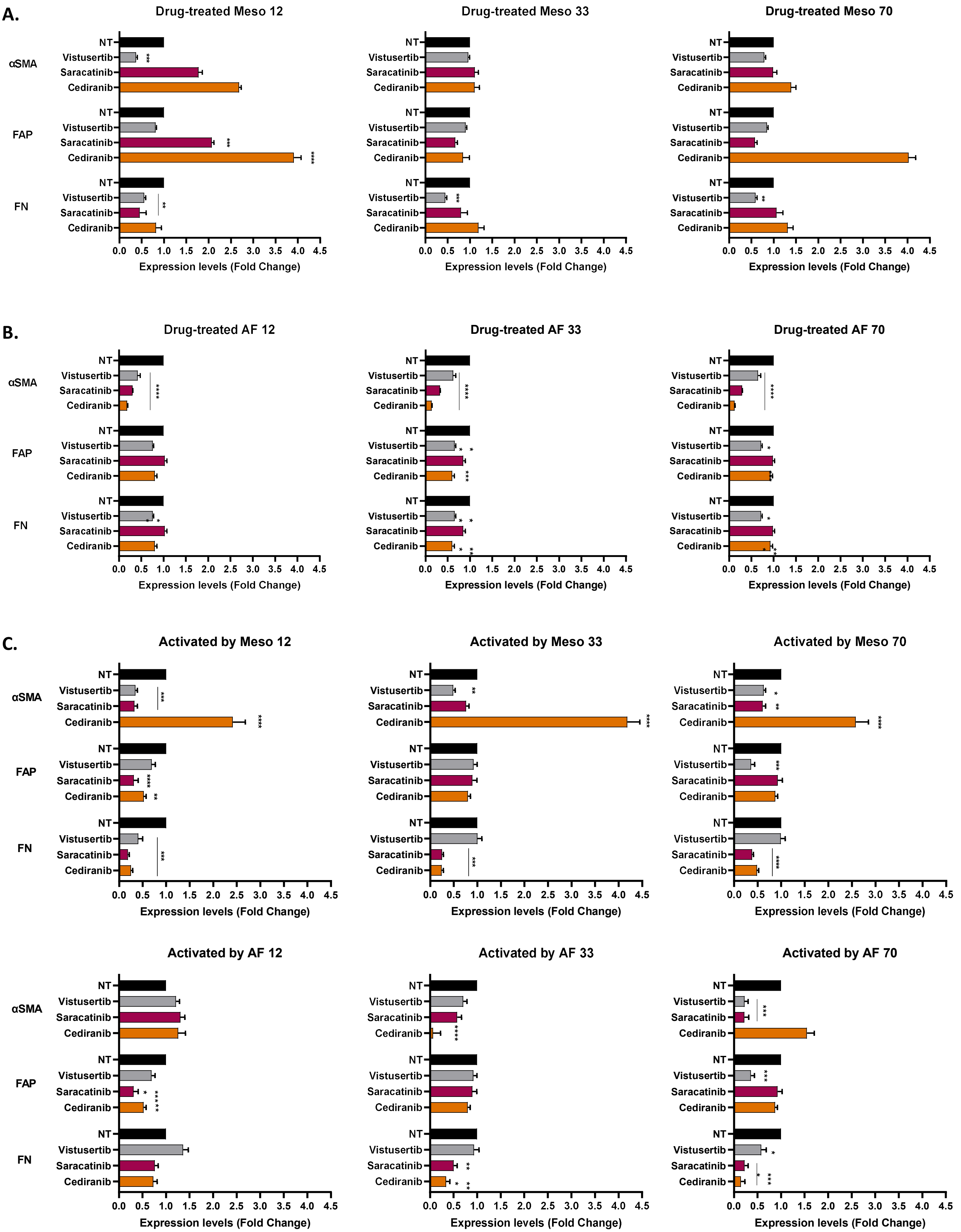
A.
Drug-treated Meso 12
Drug-treated Meso 33
Drug-treated Meso 70
αSMA
FAP
FN
NT
Vistusertib
Saracatinib
Cediranib
Expression levels (Fold Change)
B.
Drug-treated AF 12
Drug-treated AF 33
Drug-treated AF 70
C.
Activated by Meso 12
Activated by Meso 33
Activated by Meso 70
Activated by AF 12
Activated by AF 33
Activated by AF 70

Supp Fig 13: The impact of vistusertib, saracatinib and cediranib on activation of productive fibroblasts was tested. The indicated mesothelioma cell lines **(A)** or AFs **(B)** were treated with 10µM of individual drugs before their drug-devoid conditioned media was used to incubate naïve fibroblasts. **(C)** Naïve fibroblasts were treated with 10µM of individual drugs before being exposed to conditioned media from the indicated mesothelioma cells or AFs. (A-C) Cell lysates from incubated fibroblasts at 72h were analysed by SDS-PAGE/Western blotting for the indicated proteins. Three independent biological replicates were quantified by densitometry and the signal for the investigated proteins normalized to that of β-actin used as a loading control. The data show the mean ± SEM of 3 independent experiments. Statistics: One-way ANOVA (*; P<0.05, **; P<0.01, ***; P<0.005 ****; P<0.001).

Supp Fig 14

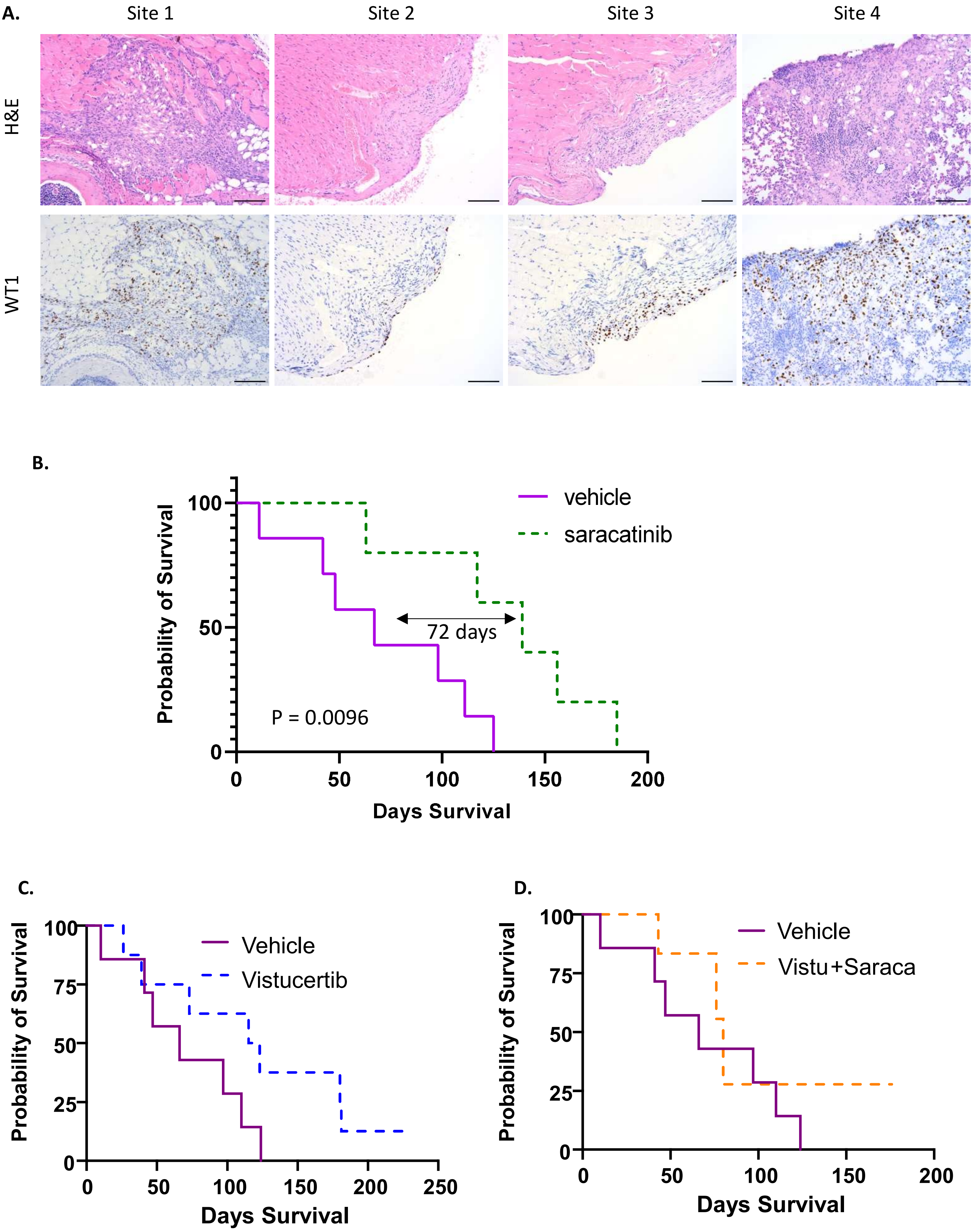

Supp Fig 14: (A) Pictures of H&E staining and WT1 immunohistochemistry of 4 tumour sites from a *Nf2/Bap1/Cdkn2a* triple-floxed mouse at 75 days post induction. Effect of saracatinib (B), vistusertib (15mg/kg/day) (C) or saracatinib/vistucertib (3.75mg/kg/day and 5mg/kg/day) combination (D) on the survival of *Nf2/Bap1/Cdkn2a* triple-floxed mice exposed to asbestos.
