# Supplementary material for: Crosstalk with lung fibroblasts shapes the growth and therapeutic response of mesothelioma cells
Source: Cell Death Dis. 2023 Nov 8;14(11):725. doi: 10.1038/s41419-023-06240-x (PMC10632403; doi:10.1038/s41419-023-06240-x)
Supplement: Supplementary file 3 — Original blots [file 41419_2023_6240_MOESM3_ESM.pptx]

## Slide 1
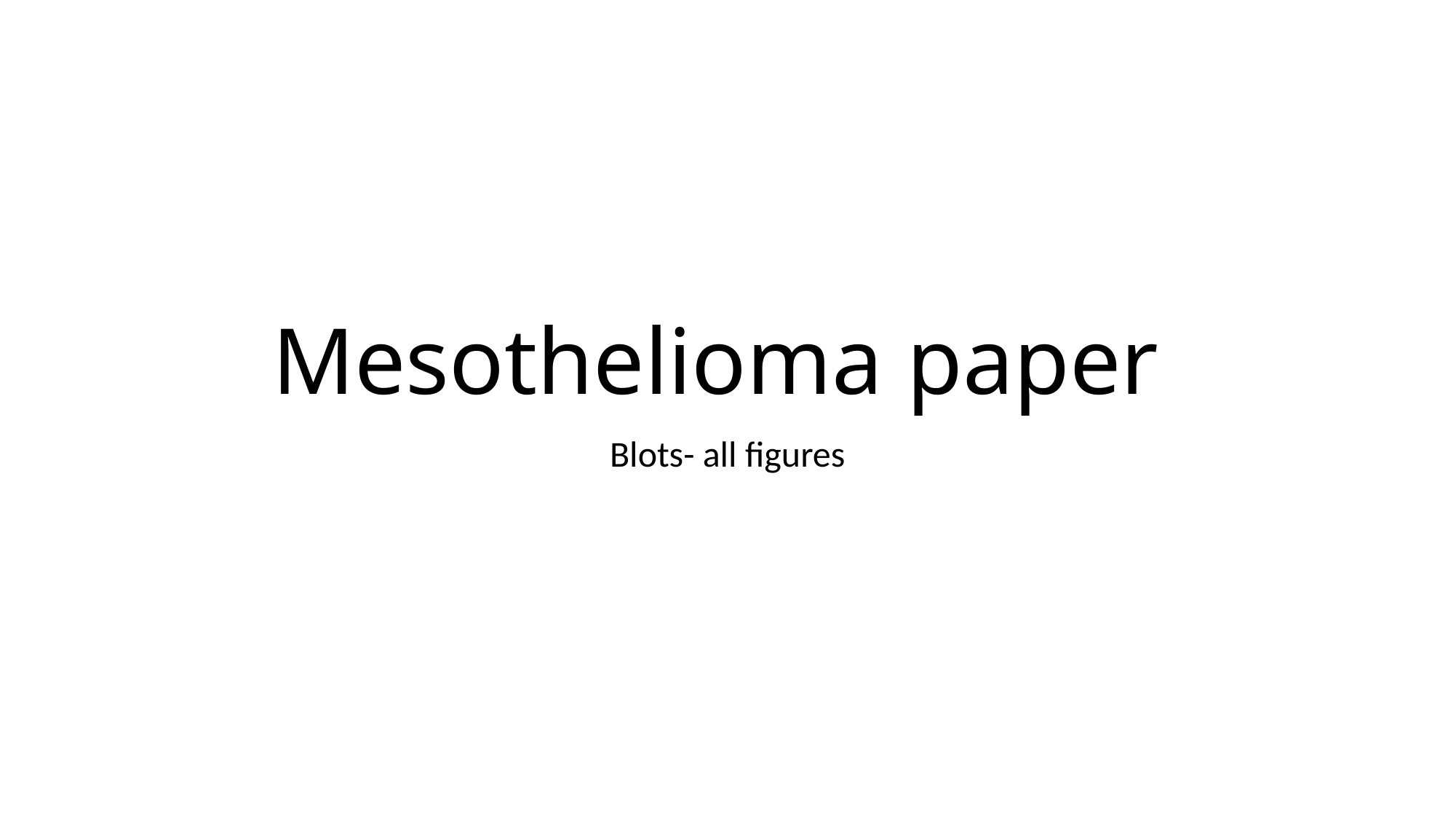

# Mesothelioma paper
Blots- all figures

## Slide 2
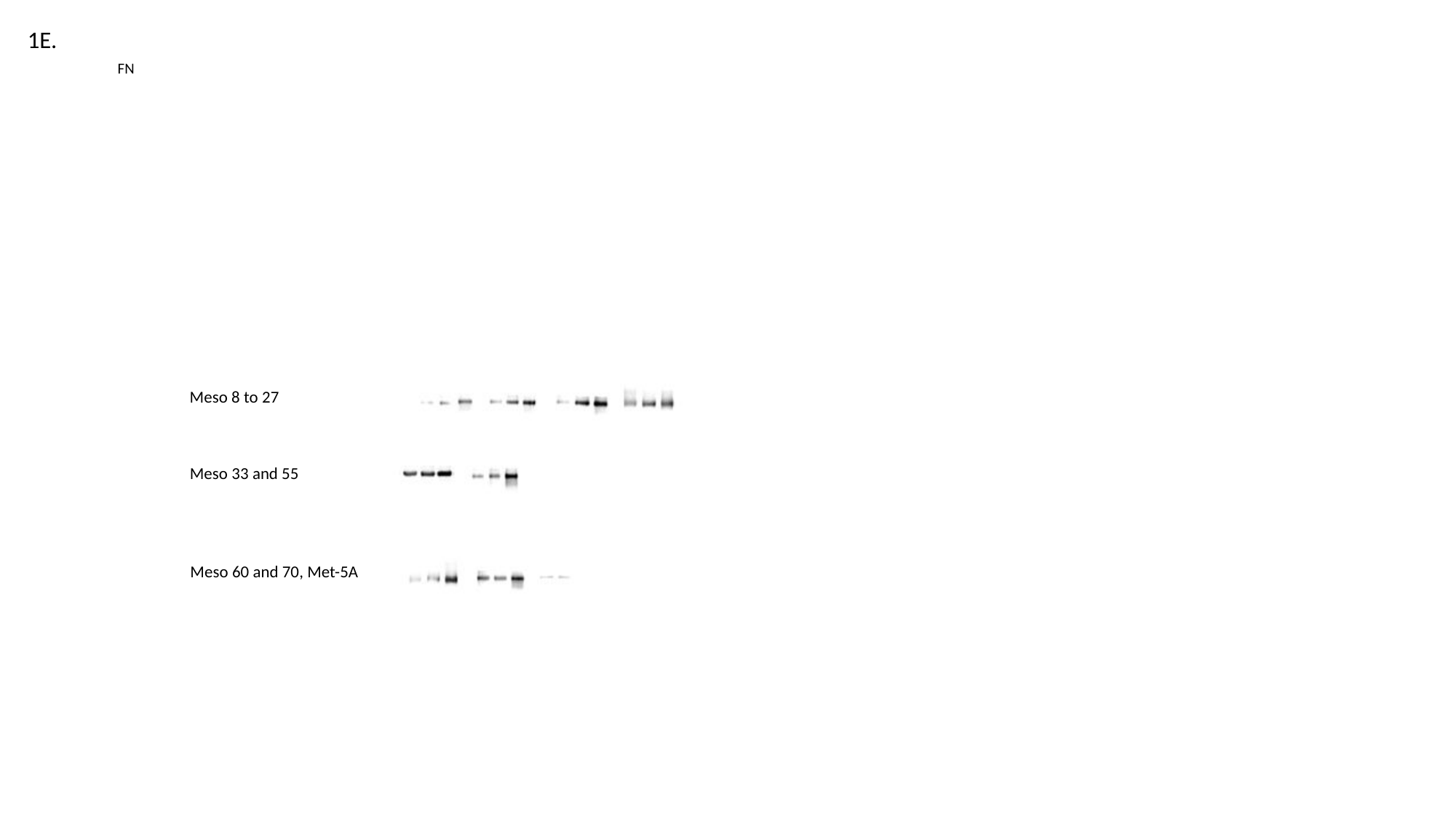

1E.
FN
Meso 8 to 27
Meso 33 and 55
Meso 60 and 70, Met-5A

## Slide 3
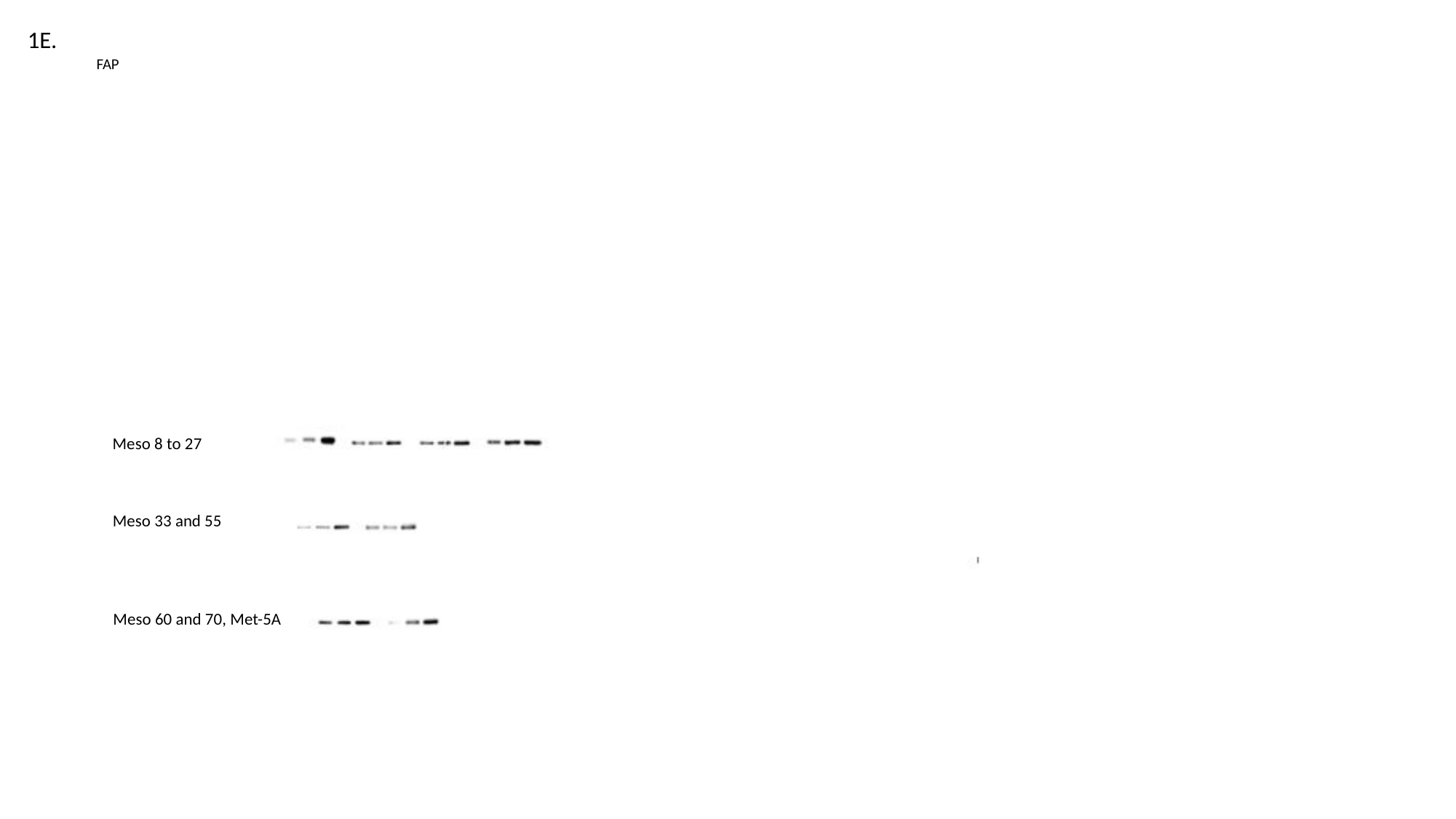

1E.
FAP
Meso 8 to 27
Meso 33 and 55
Meso 60 and 70, Met-5A

## Slide 4
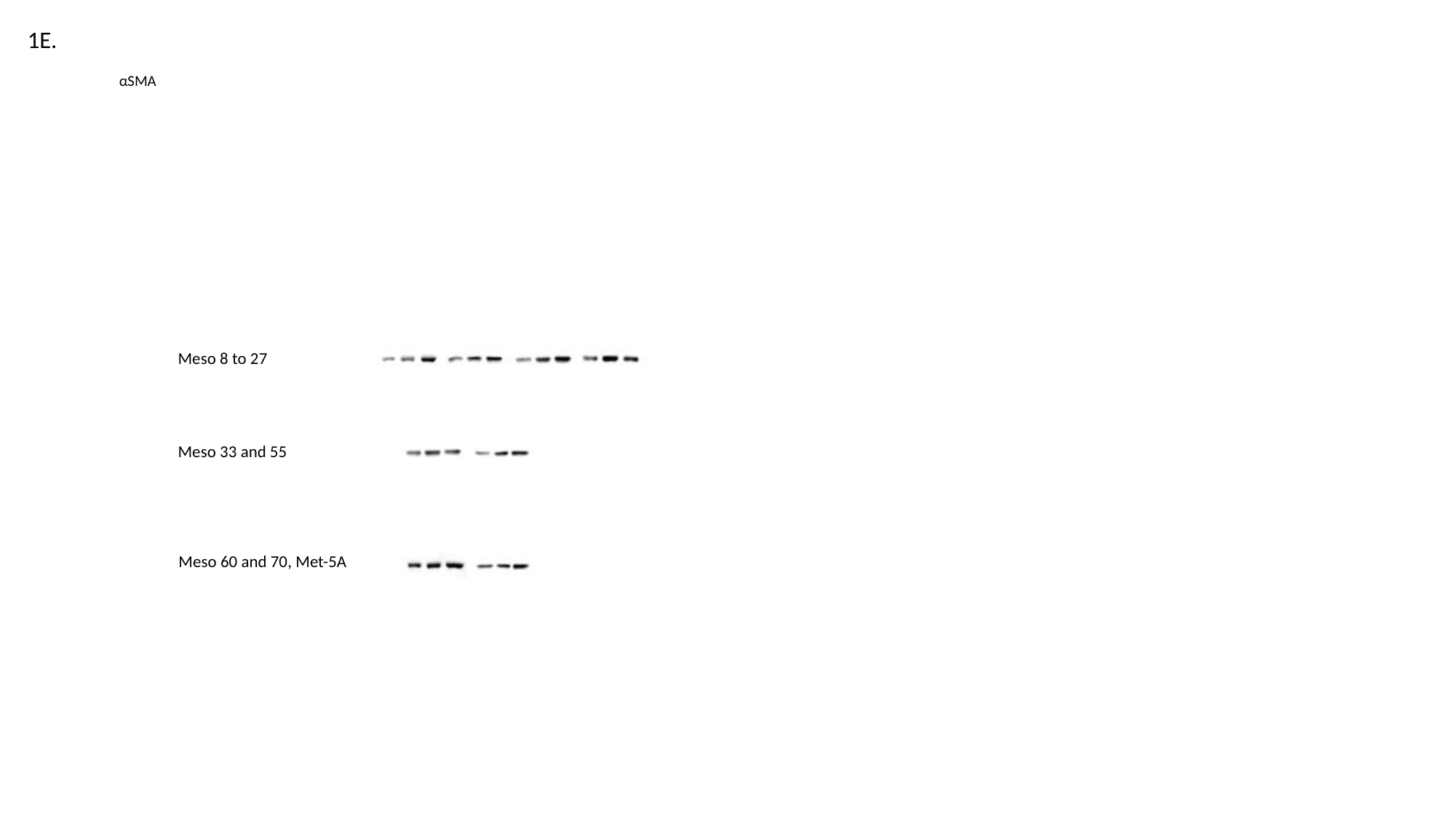

1E.
αSMA
Meso 8 to 27
Meso 33 and 55
Meso 60 and 70, Met-5A

## Slide 5
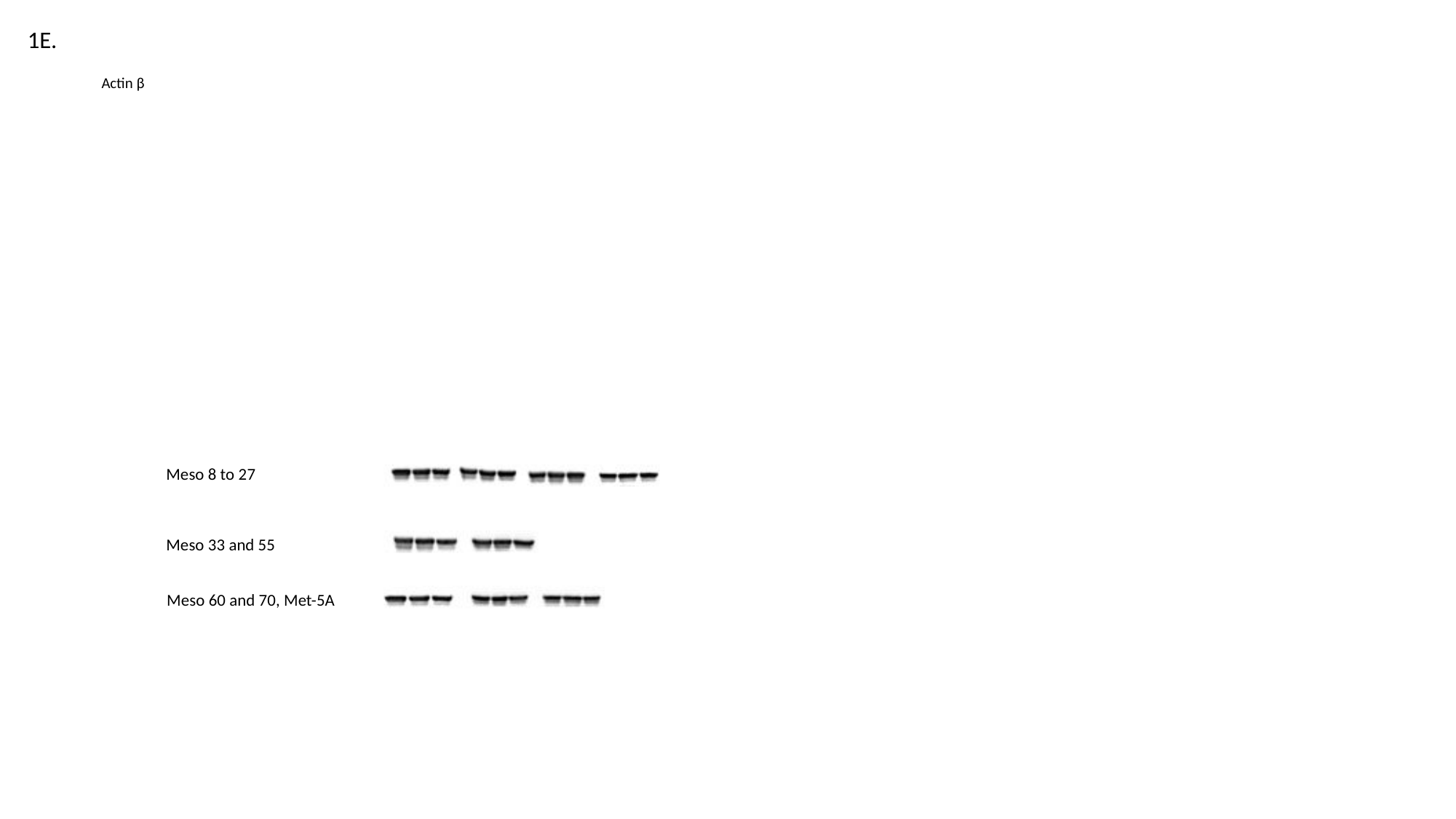

1E.
Actin β
Meso 8 to 27
Meso 33 and 55
Meso 60 and 70, Met-5A

## Slide 6
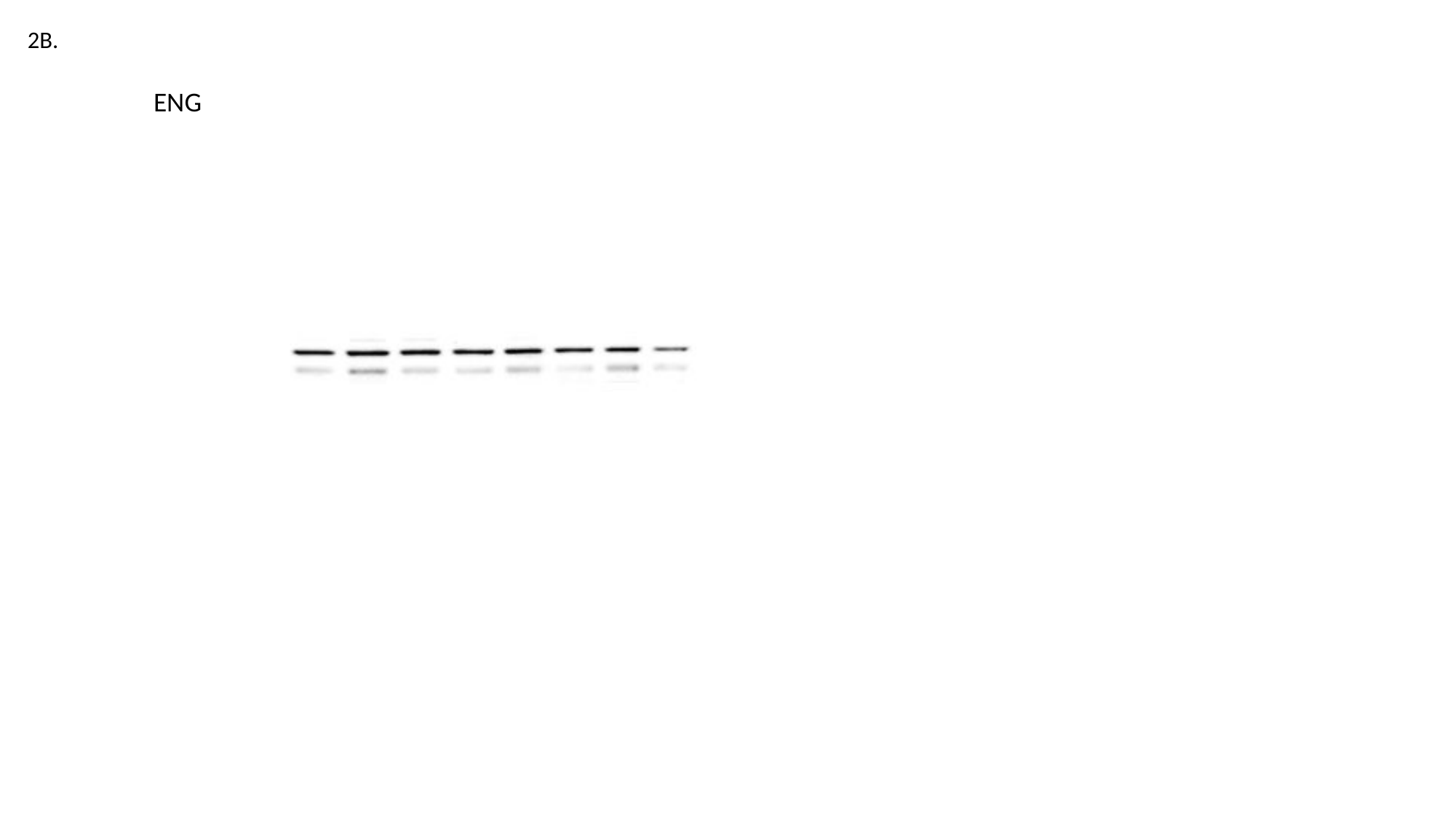

2B.
ENG

## Slide 7
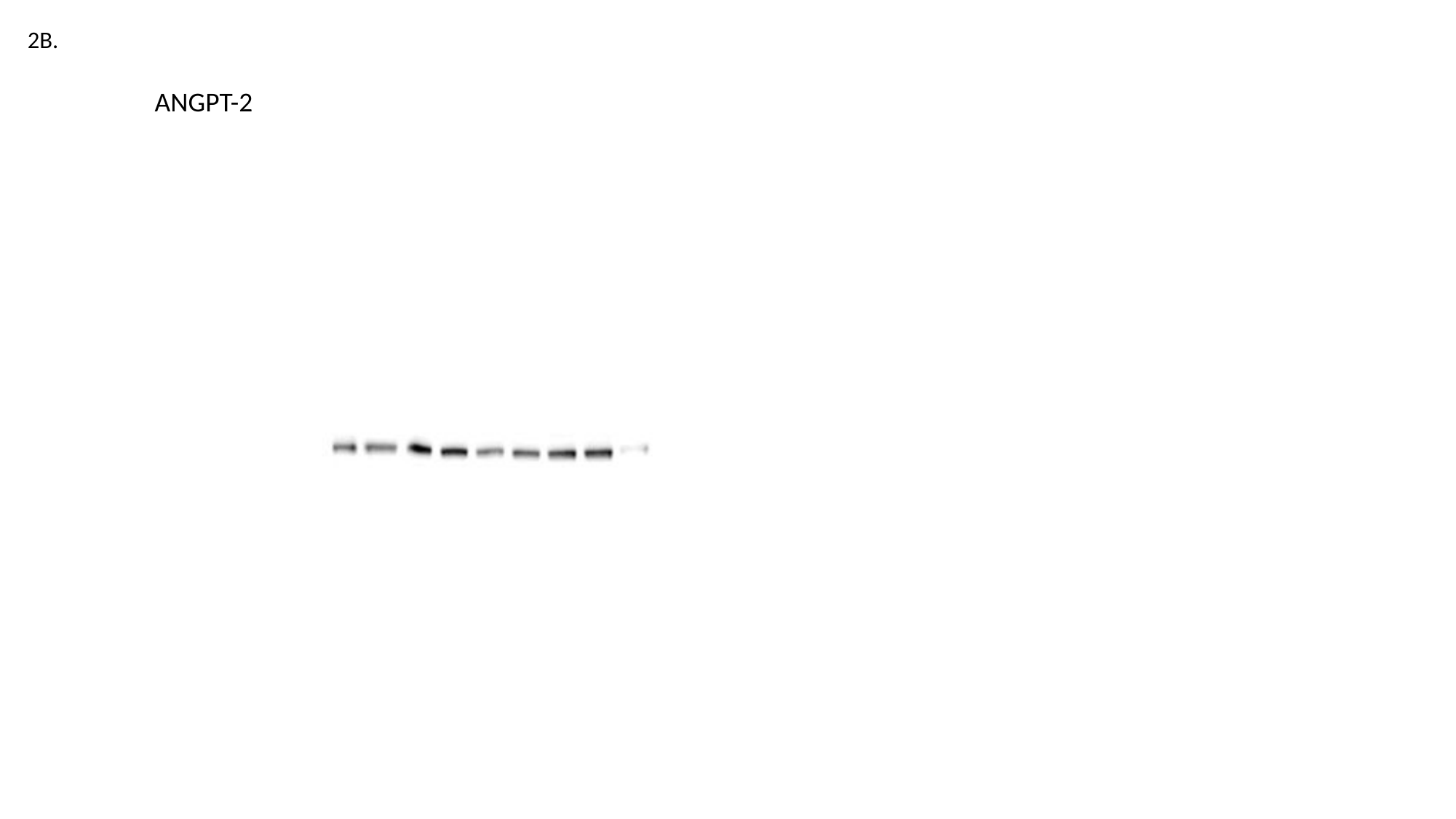

2B.
ANGPT-2

## Slide 8
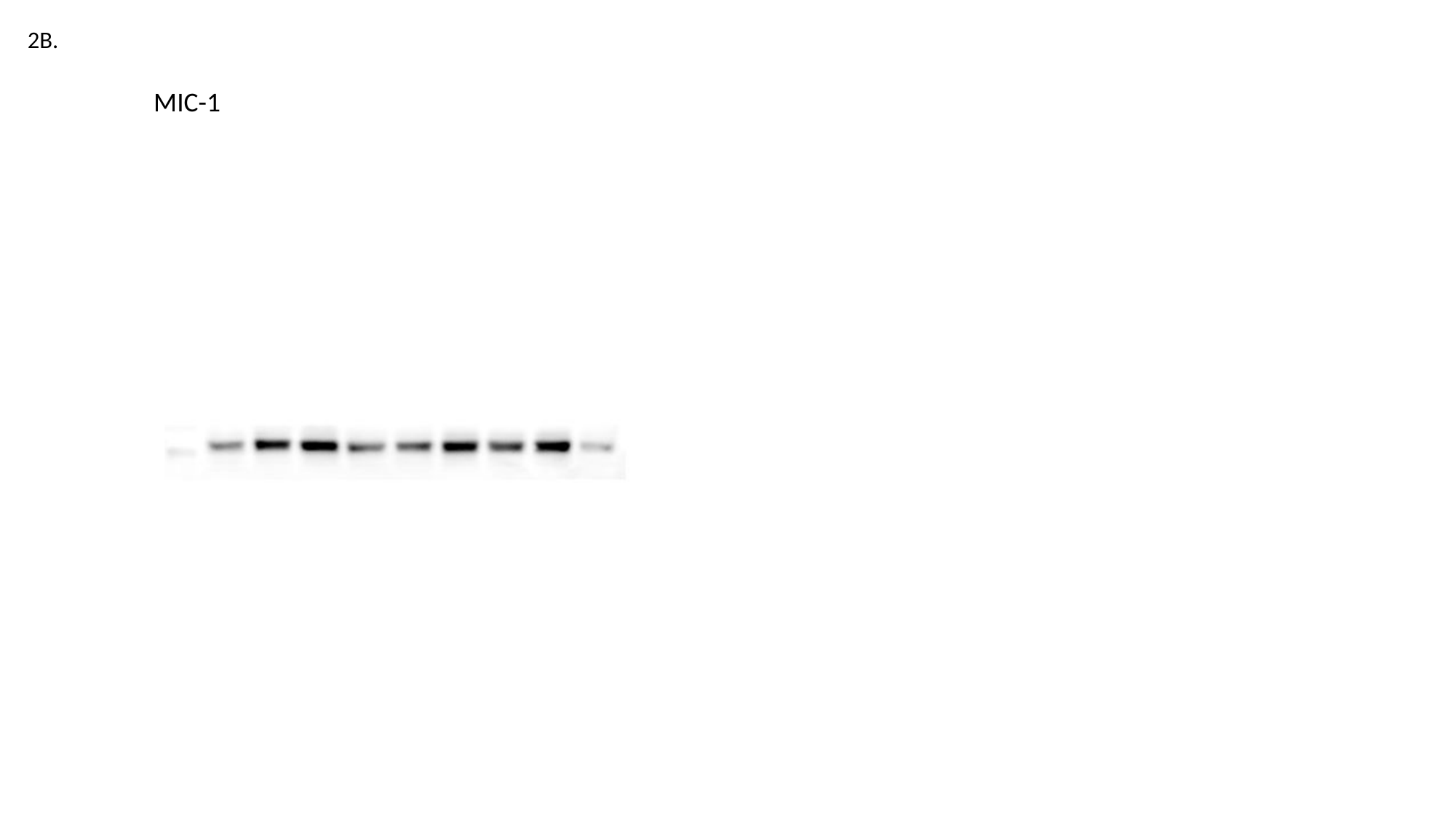

2B.
MIC-1

## Slide 9
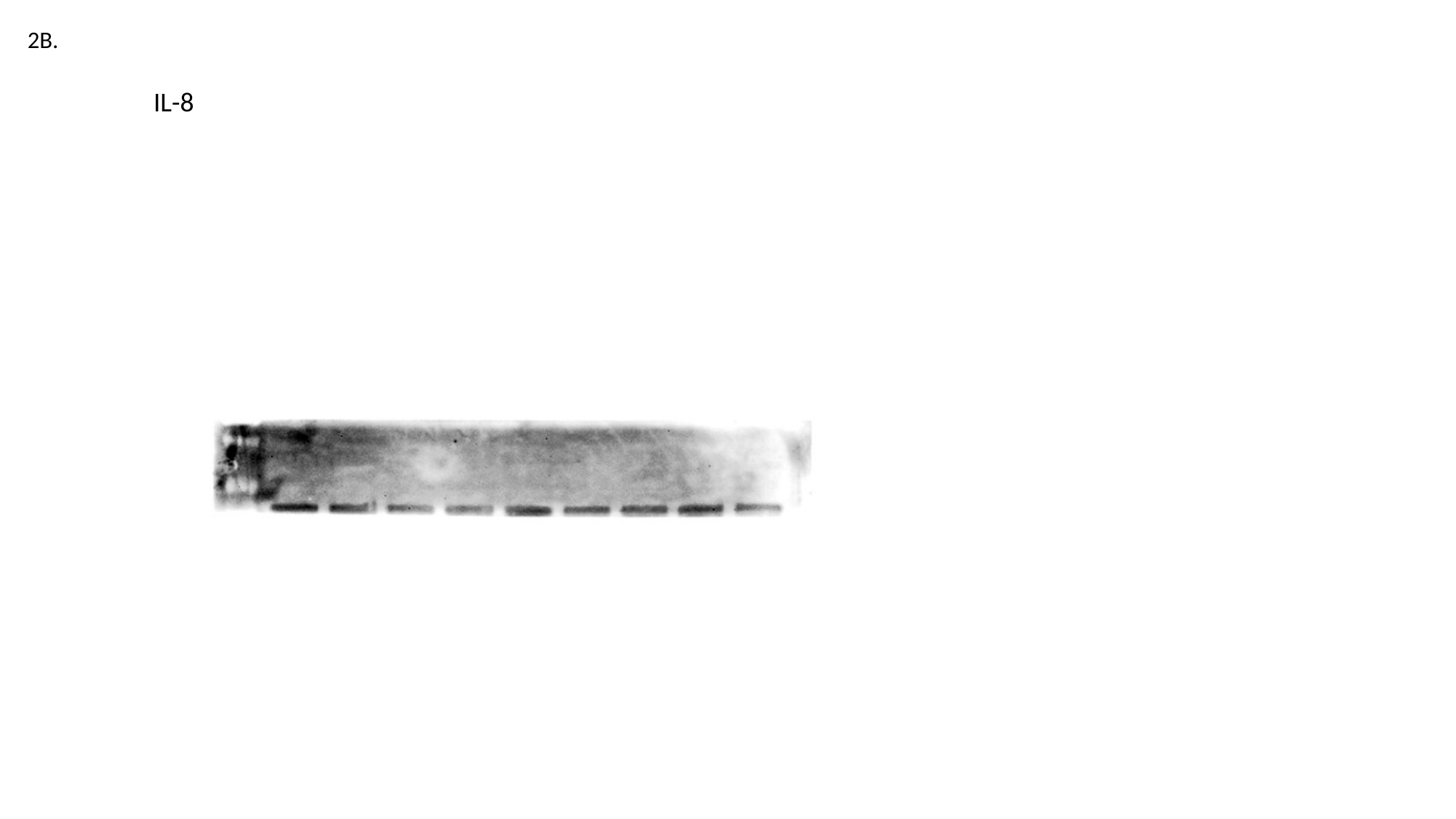

2B.
IL-8

## Slide 10
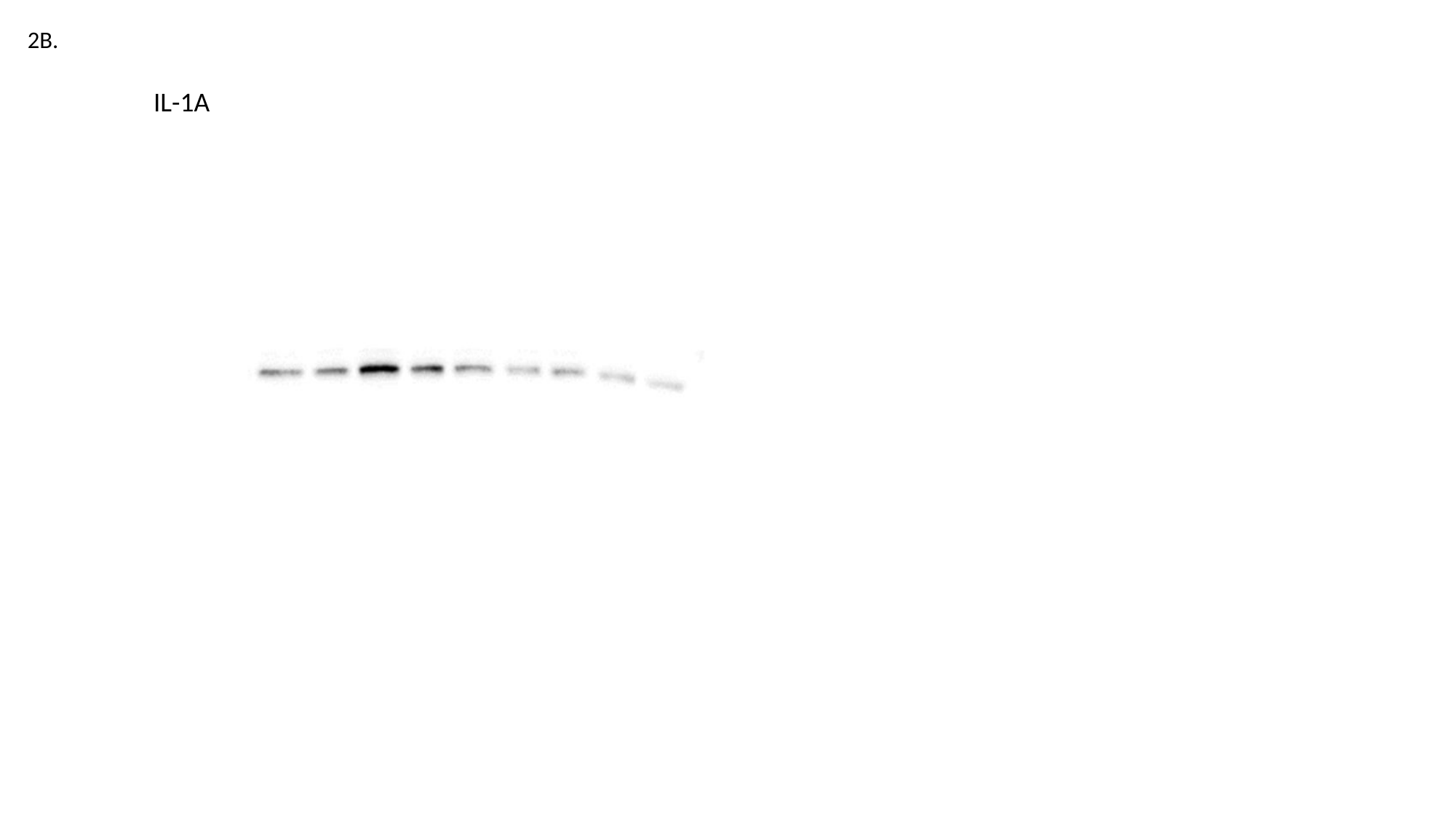

2B.
IL-1A

## Slide 11
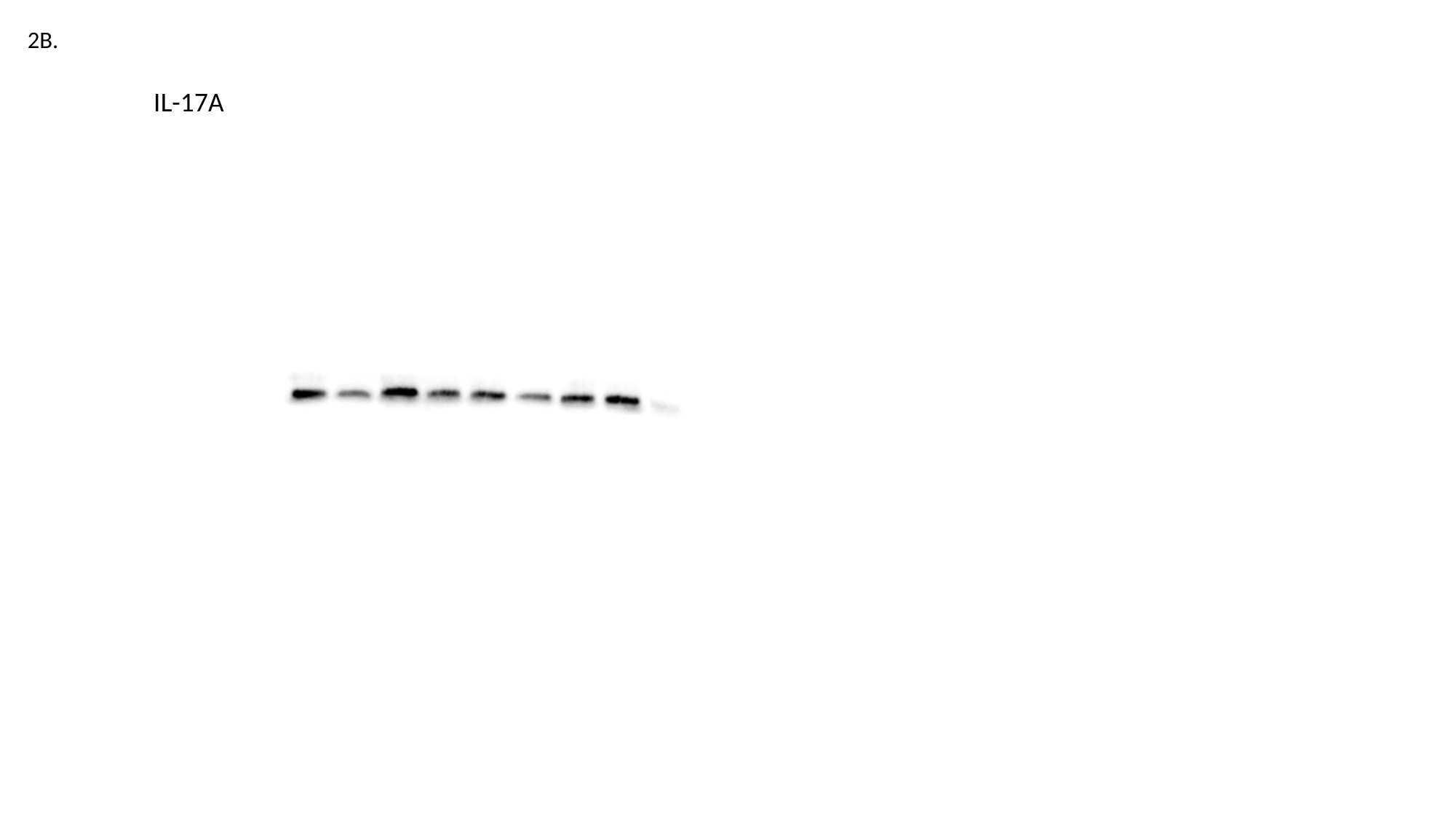

2B.
IL-17A

## Slide 12
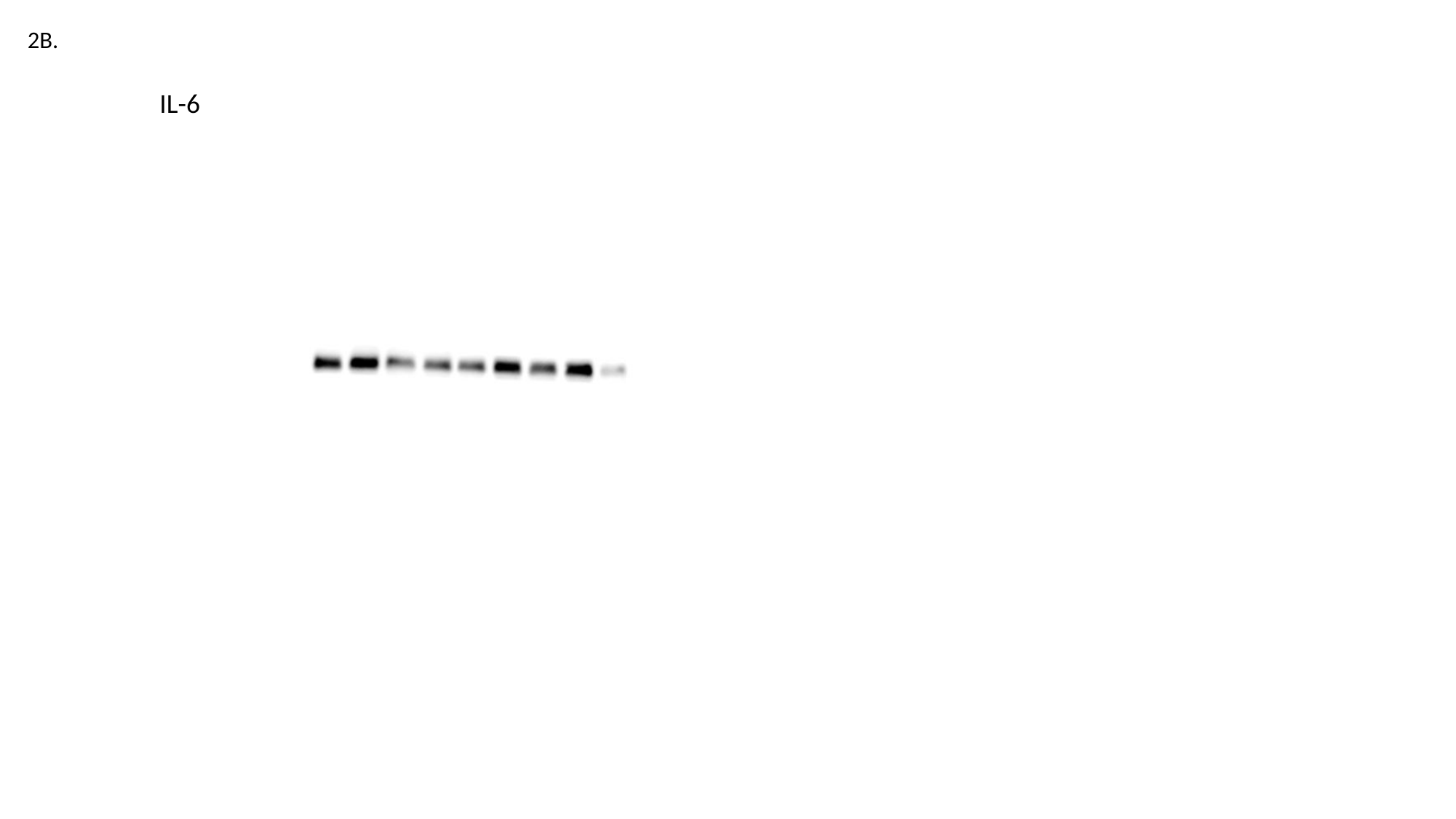

2B.
IL-6

## Slide 13
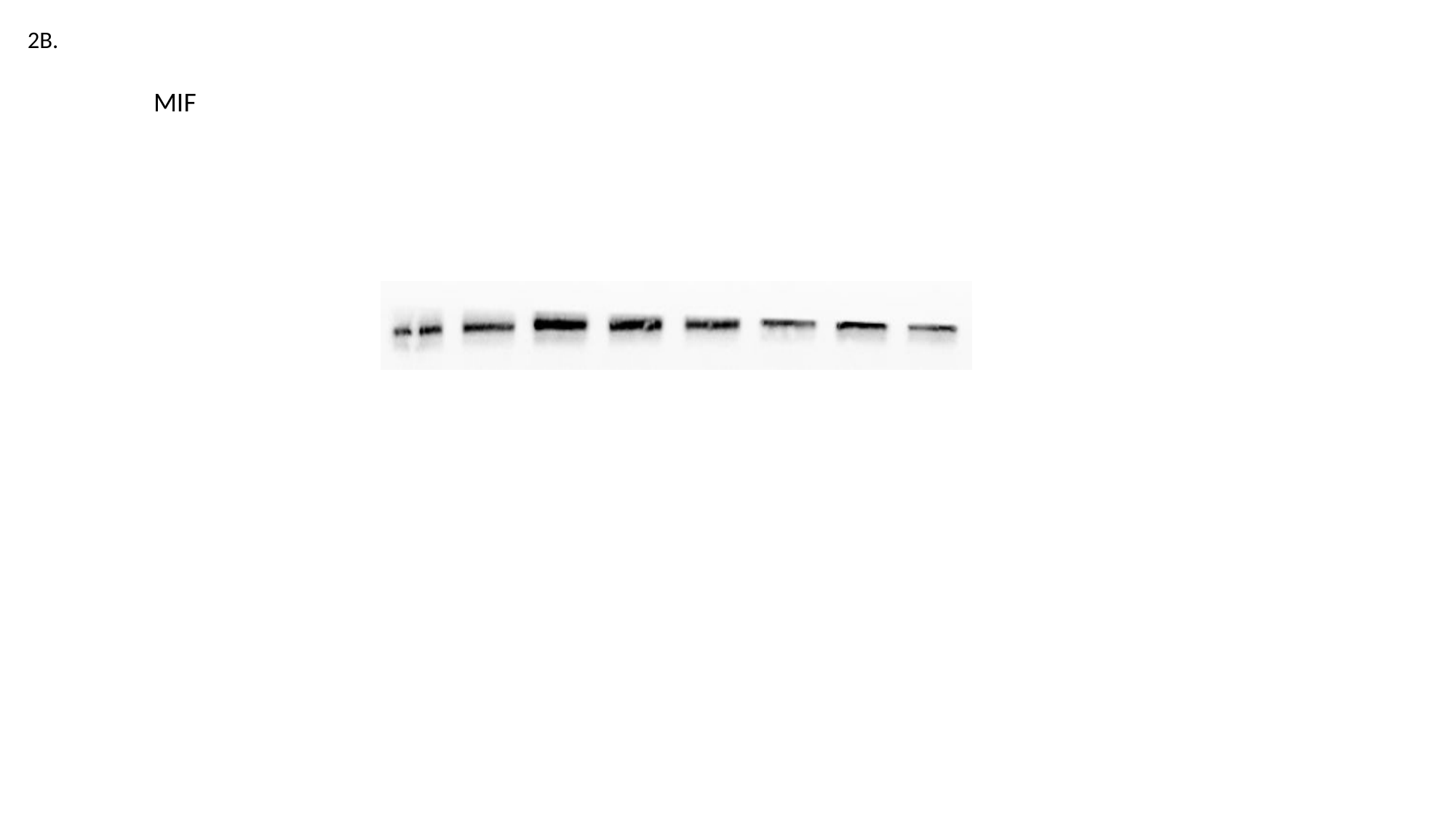

2B.
MIF

## Slide 14
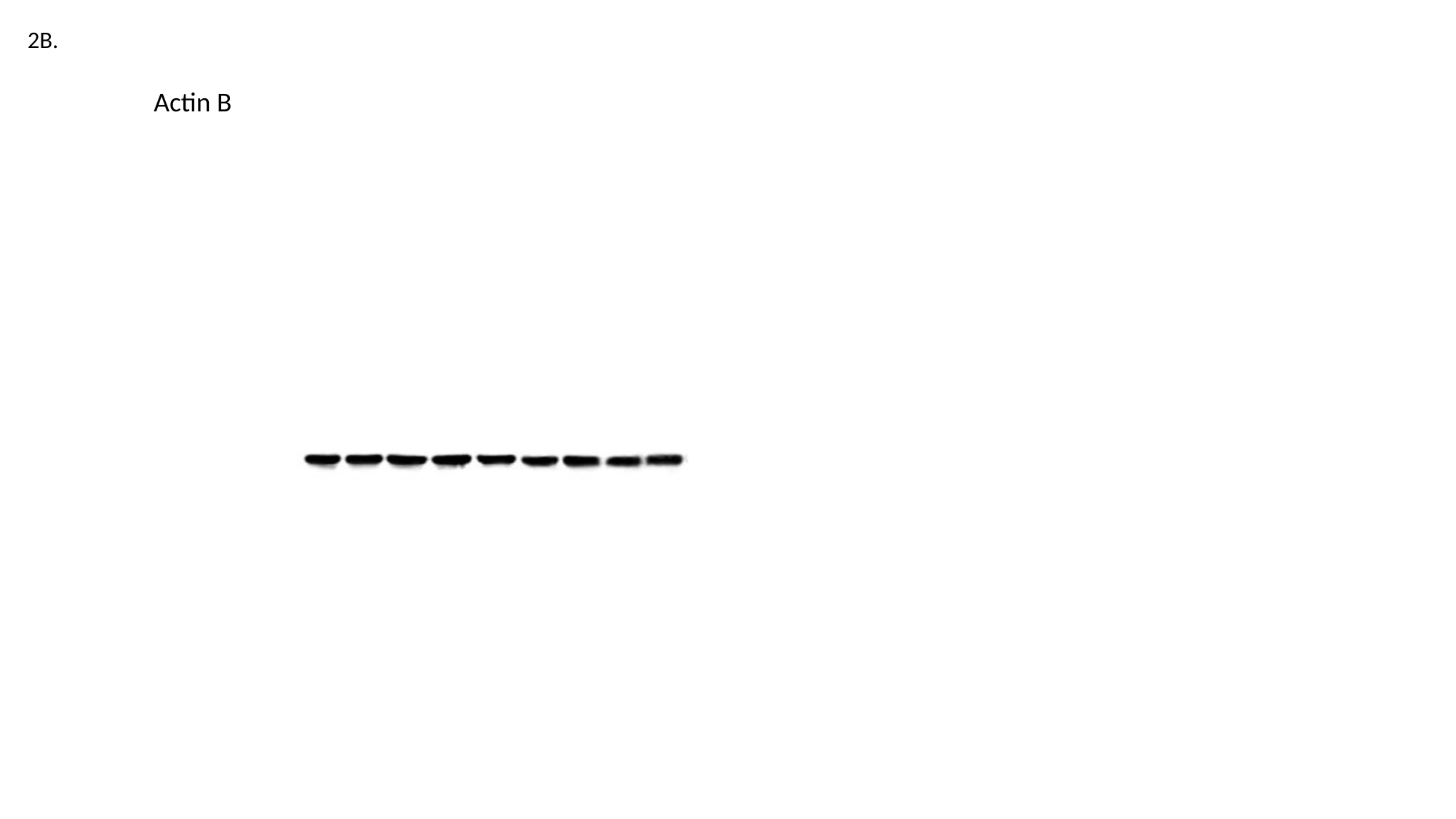

2B.
Actin B

## Slide 15
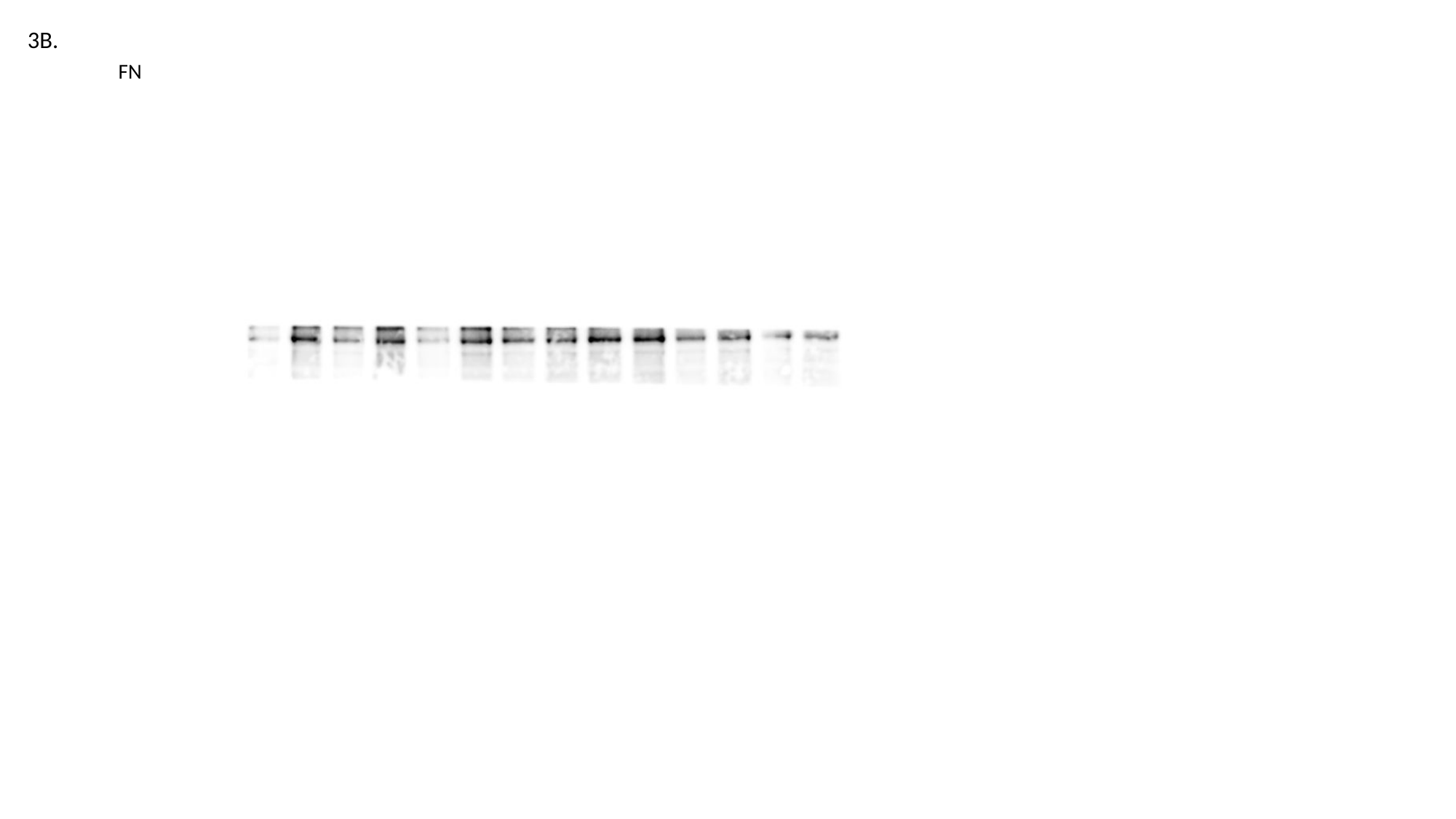

3B.
FN

## Slide 16
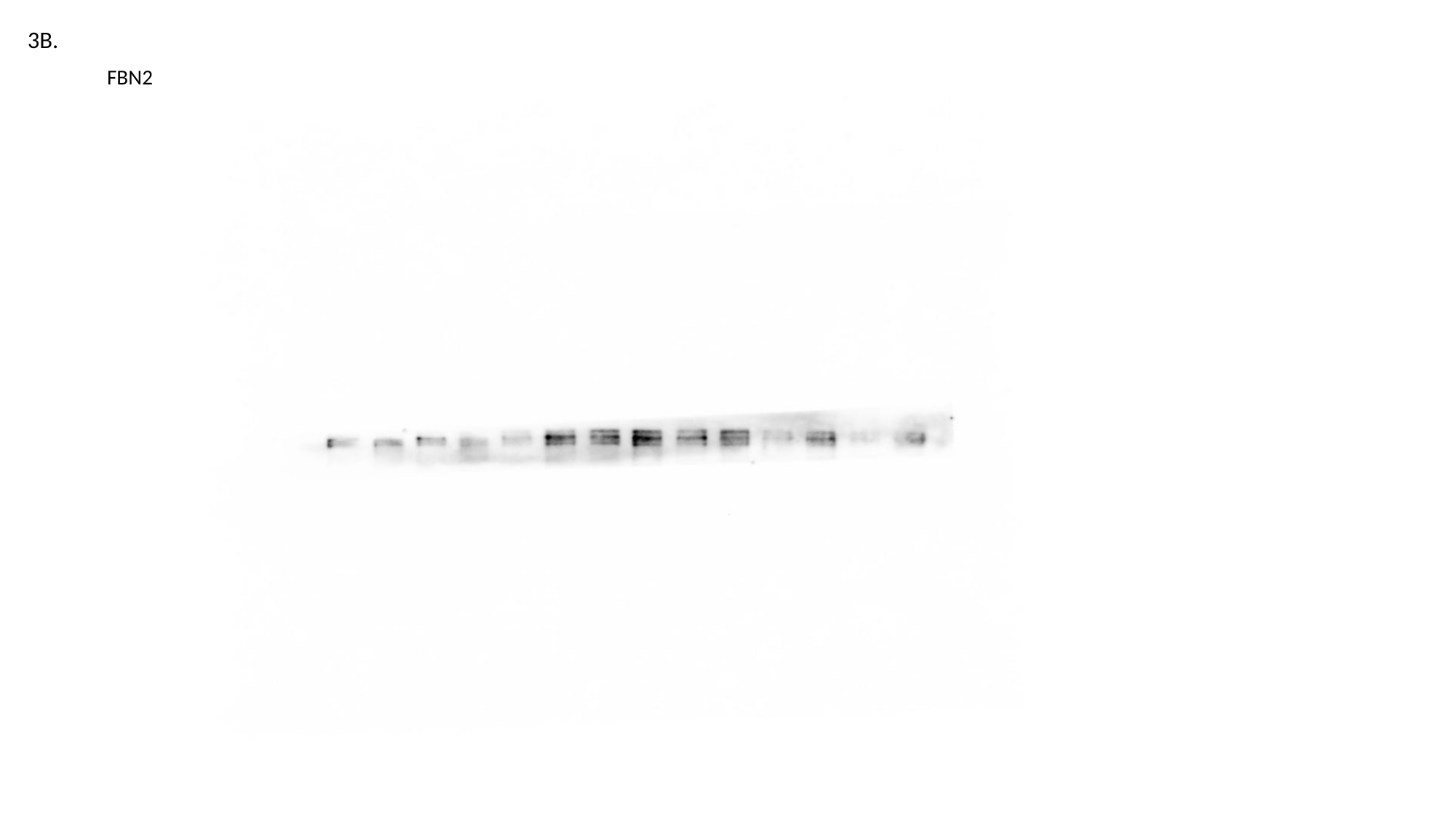

3B.
FBN2

## Slide 17
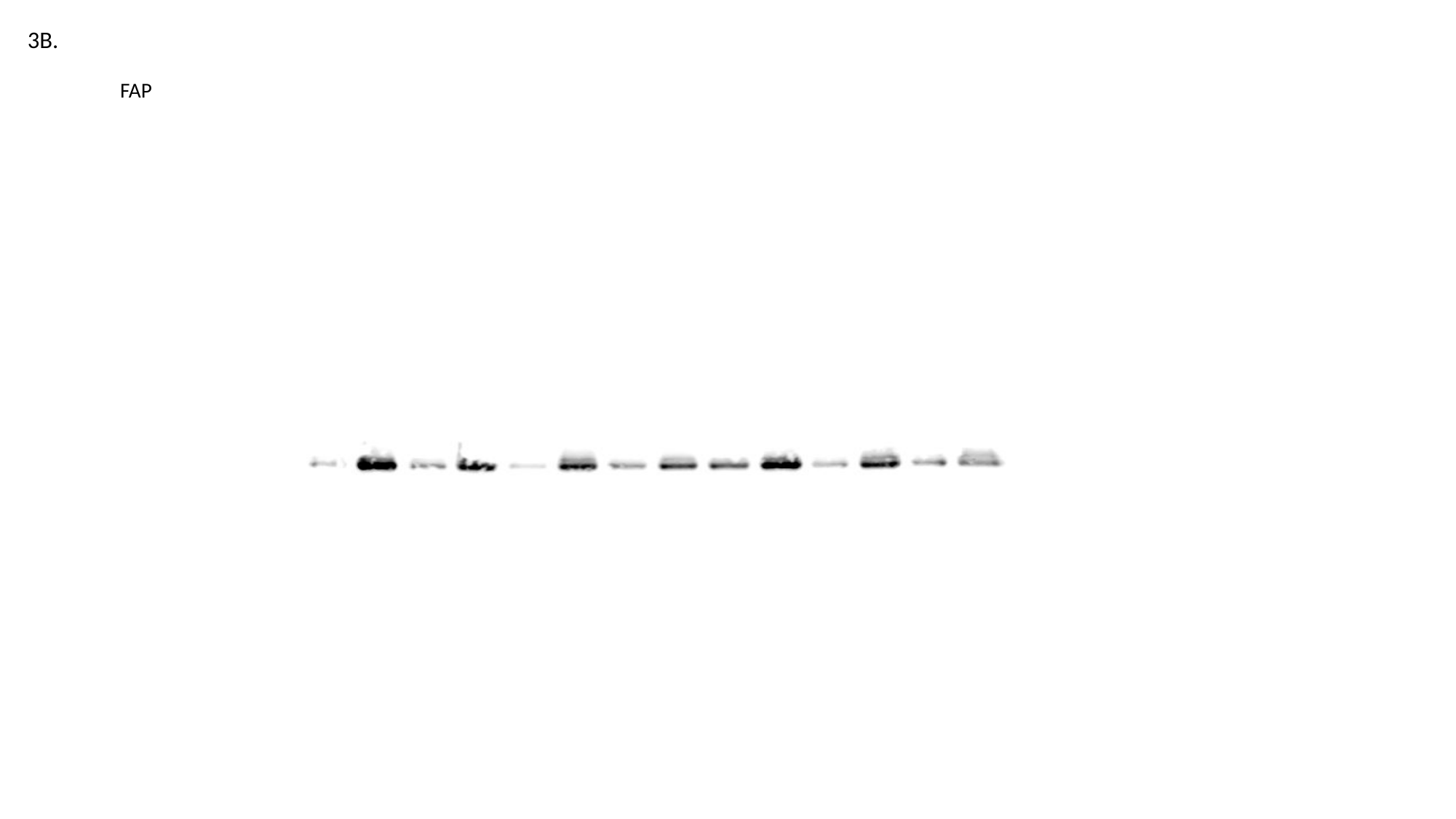

3B.
FAP

## Slide 18
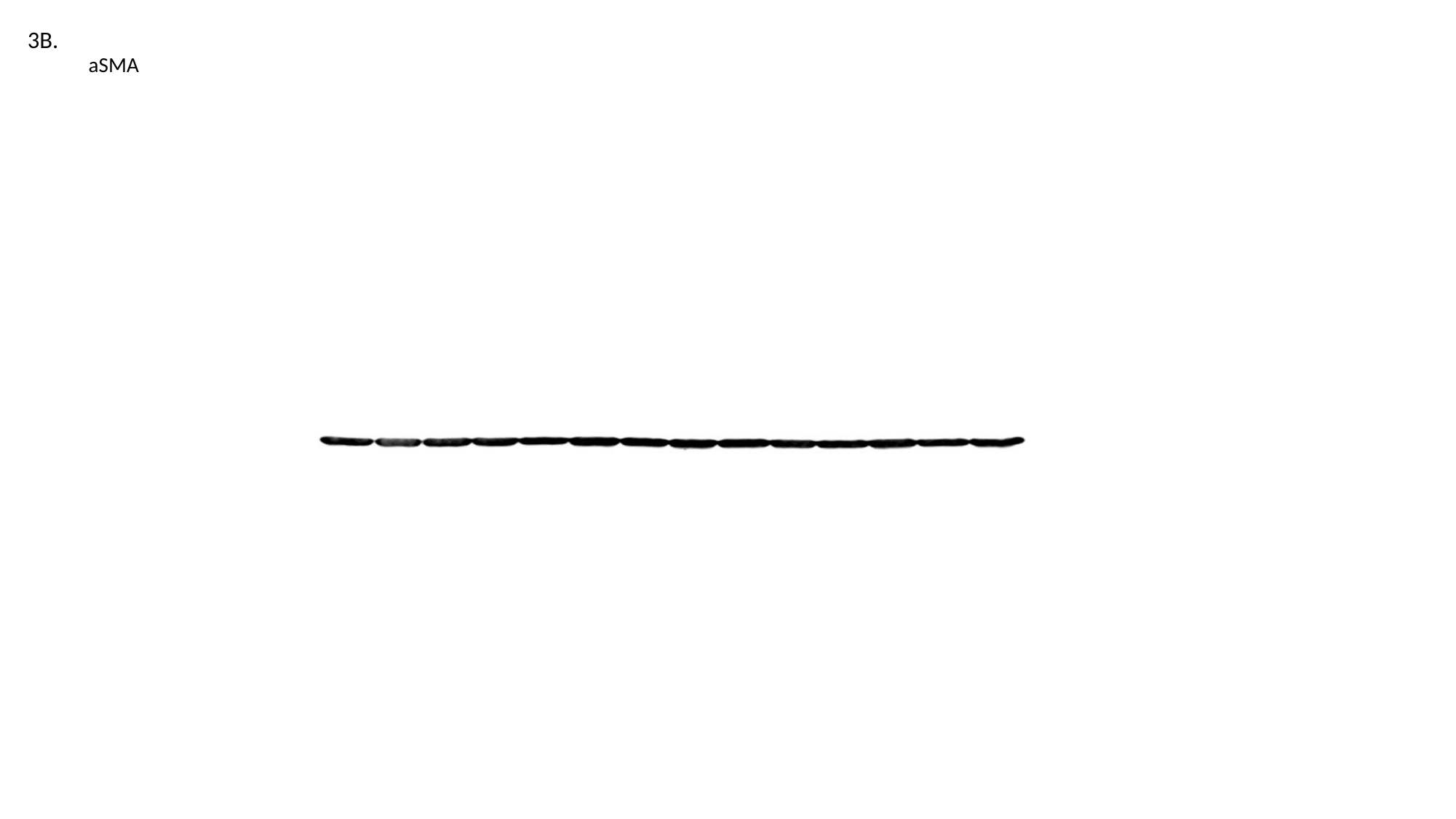

3B.
aSMA

## Slide 19
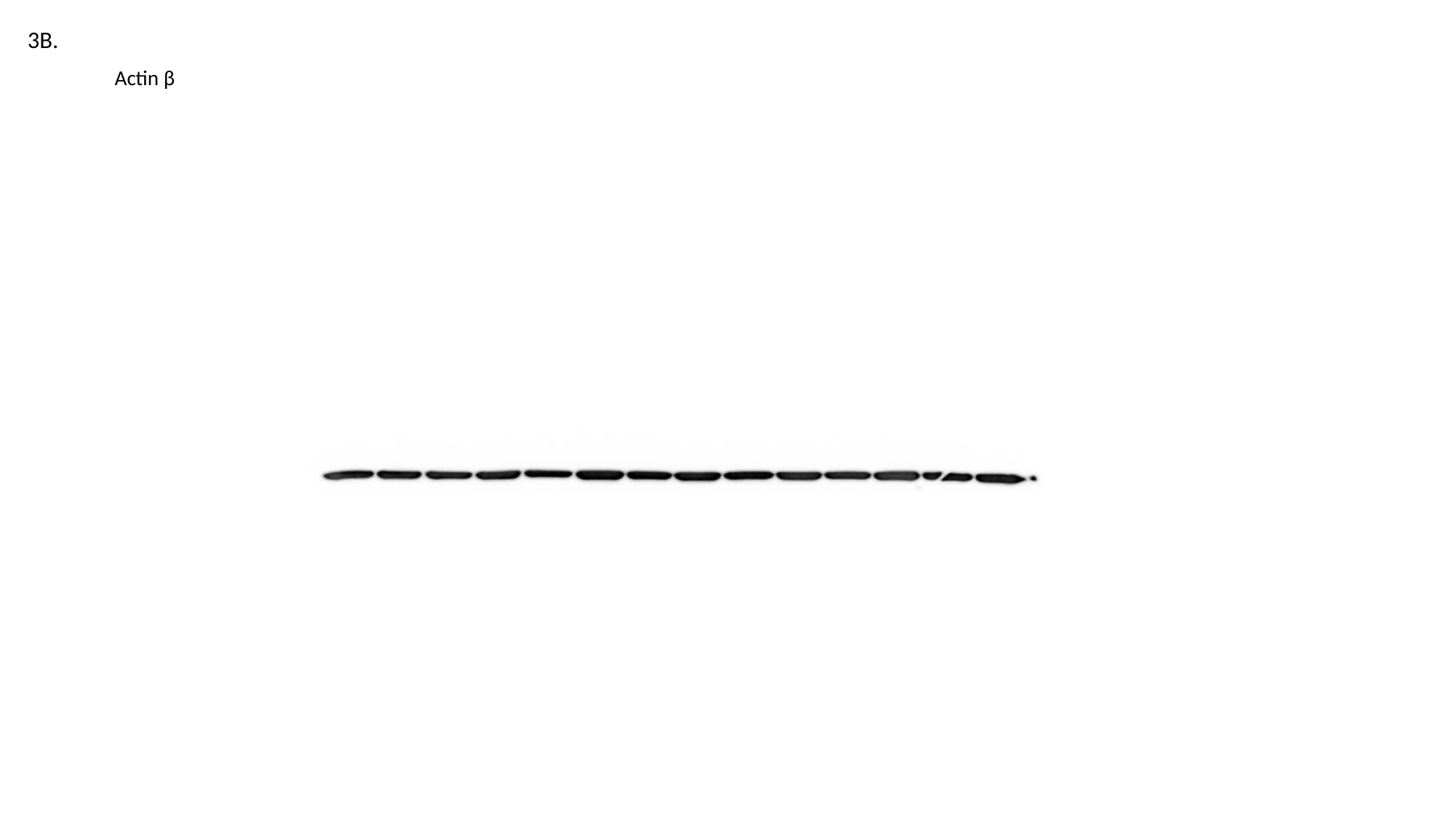

3B.
Actin β

## Slide 20
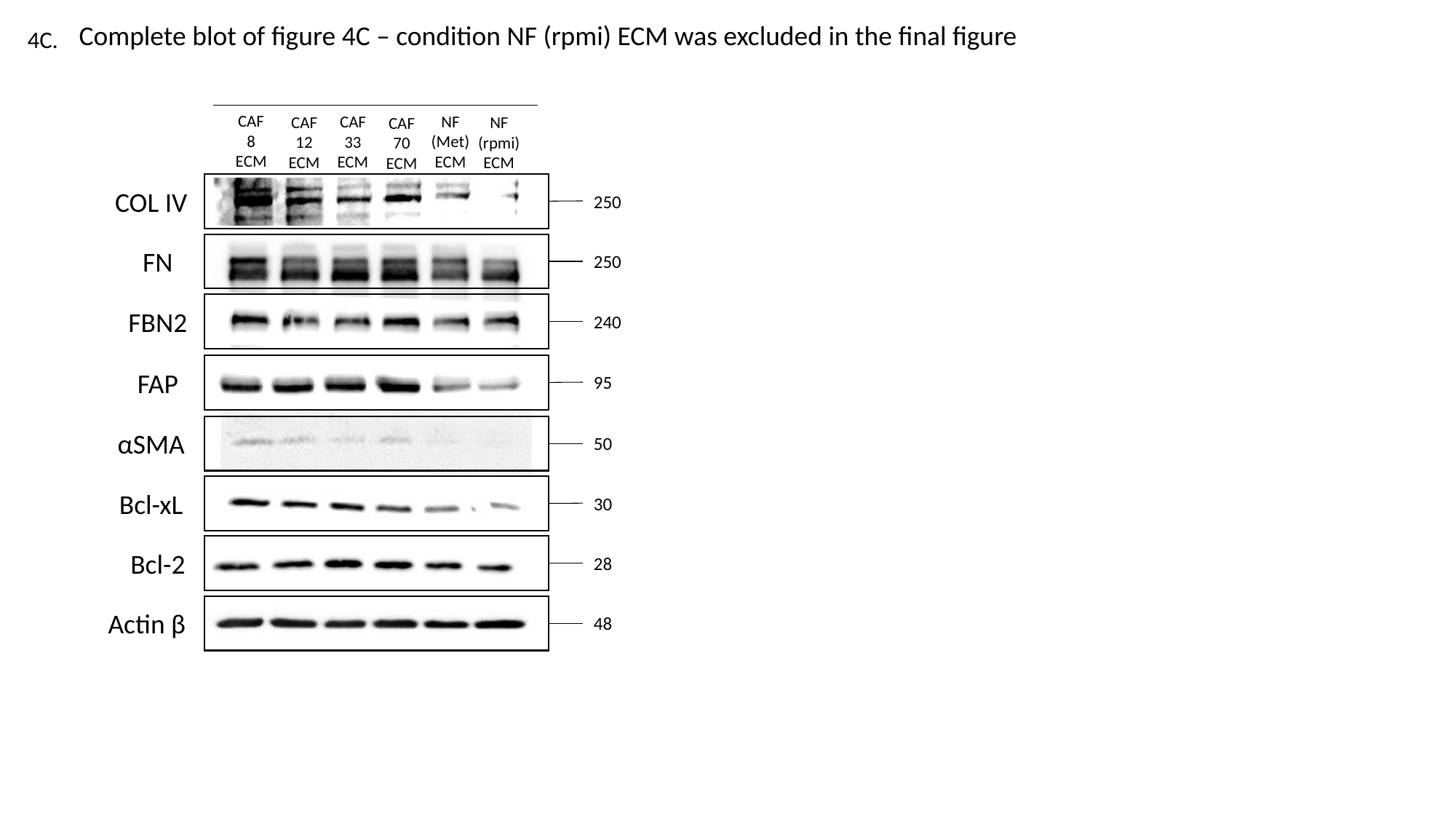

Complete blot of figure 4C – condition NF (rpmi) ECM was excluded in the final figure
4C.
CAF
8
ECM
NF
(Met)
ECM
CAF
33
ECM
CAF
12
ECM
NF
(rpmi)
ECM
CAF
70
ECM
COL IV
250
FN
250
FBN2
240
FAP
95
αSMA
50
Bcl-xL
30
Bcl-2
28
Actin β
48

## Slide 21
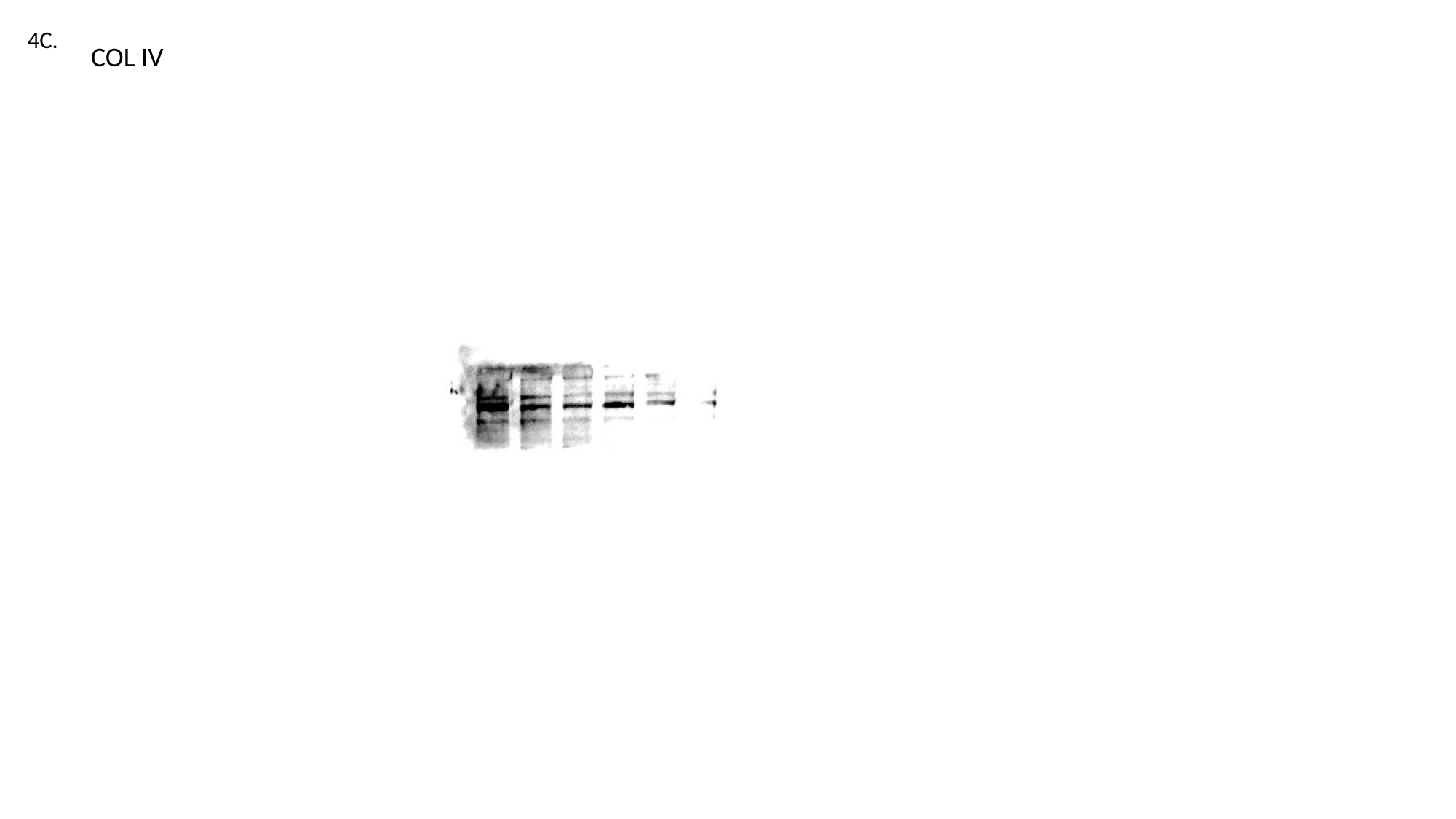

4C.
COL IV

## Slide 22
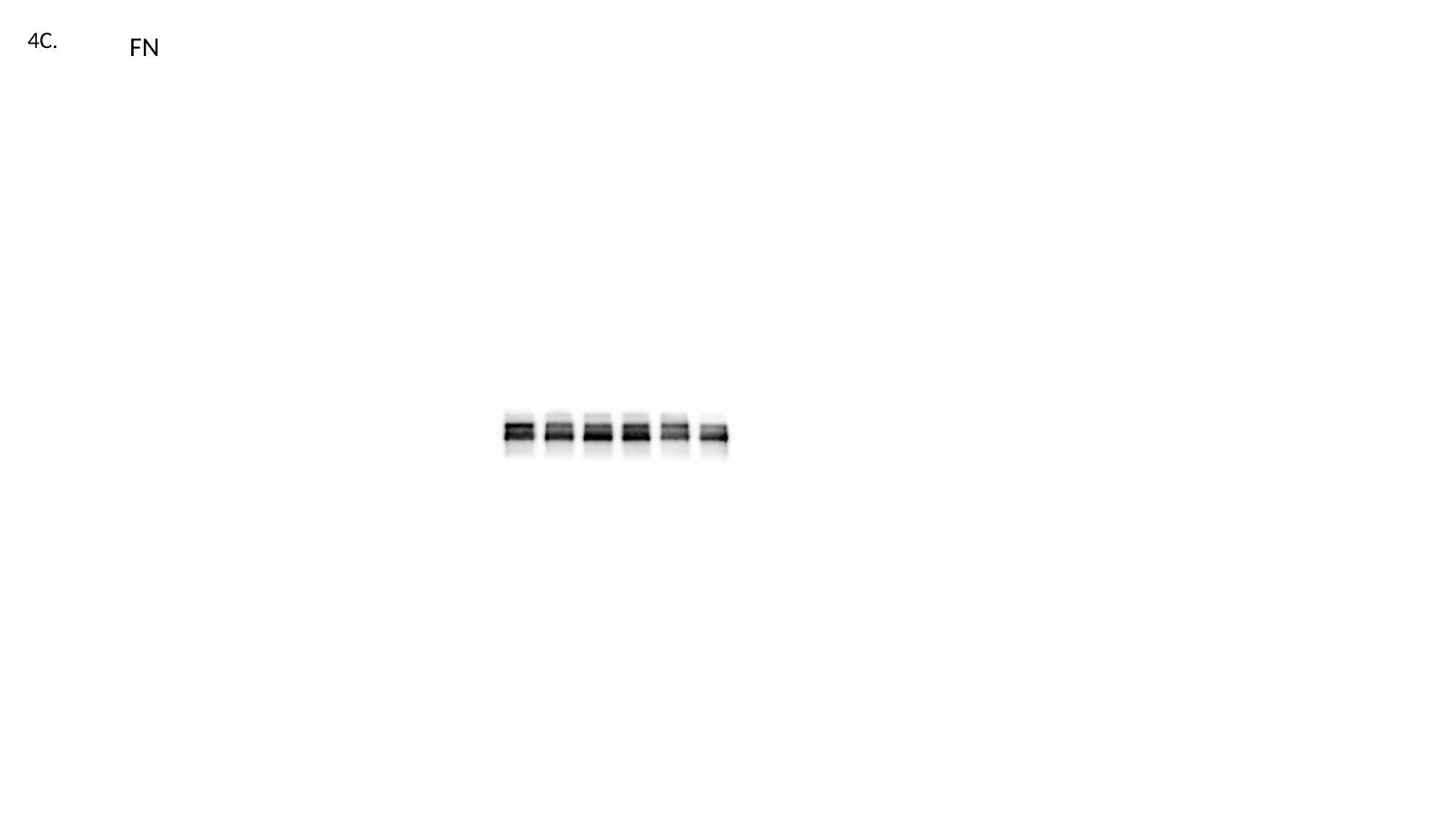

4C.
FN

## Slide 23
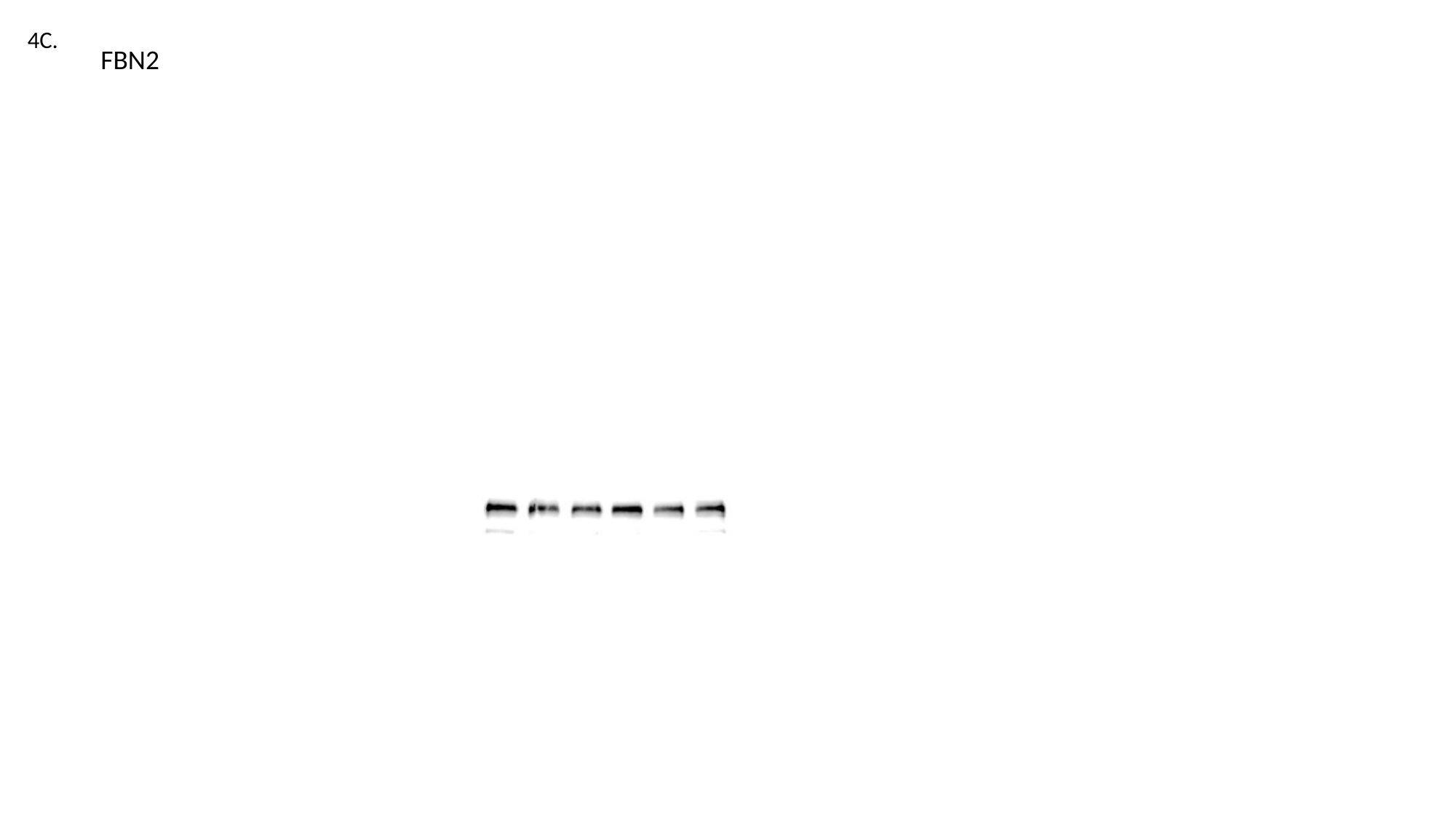

4C.
FBN2

## Slide 24
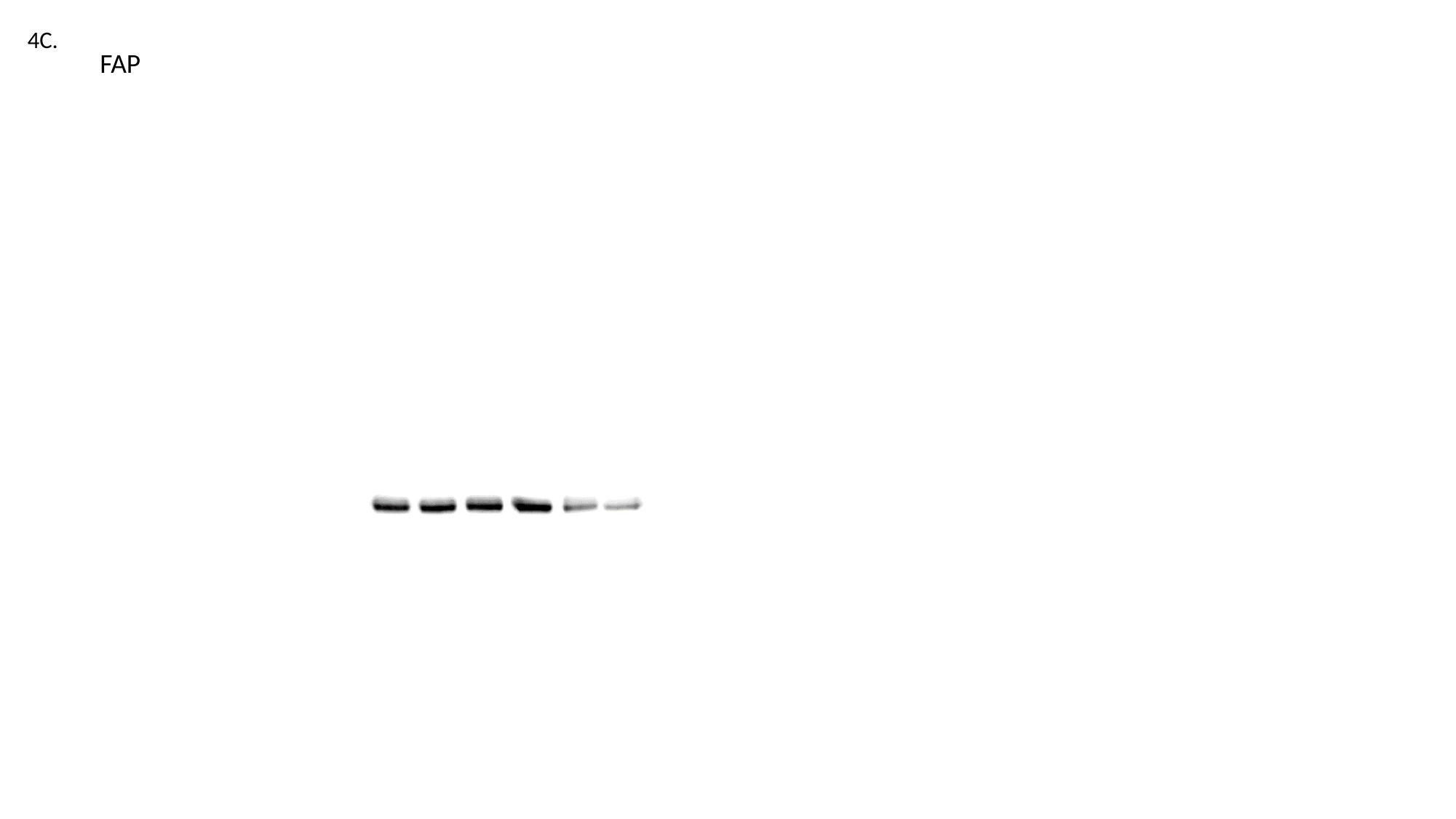

4C.
FAP

## Slide 25
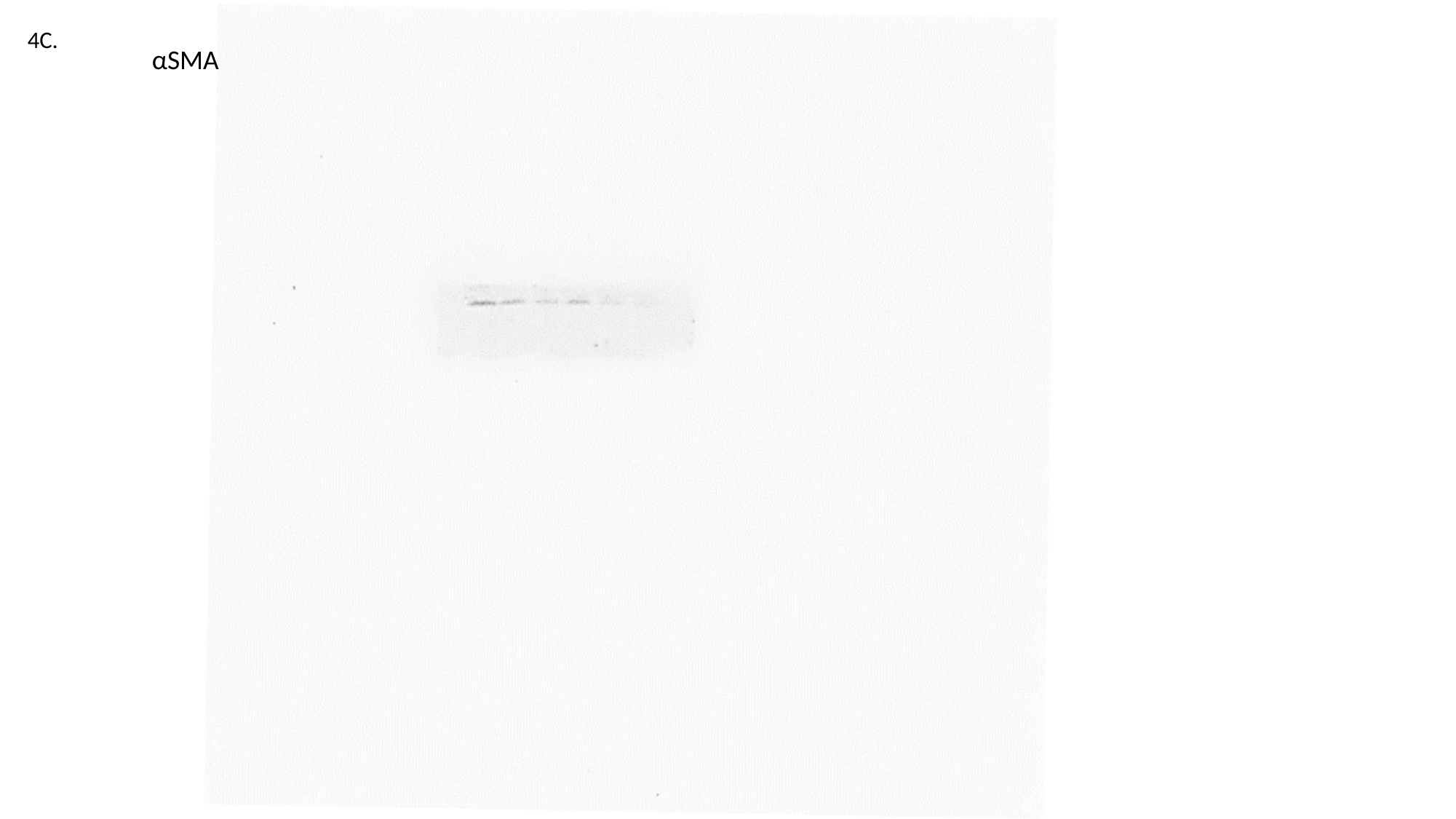

4C.
αSMA

## Slide 26
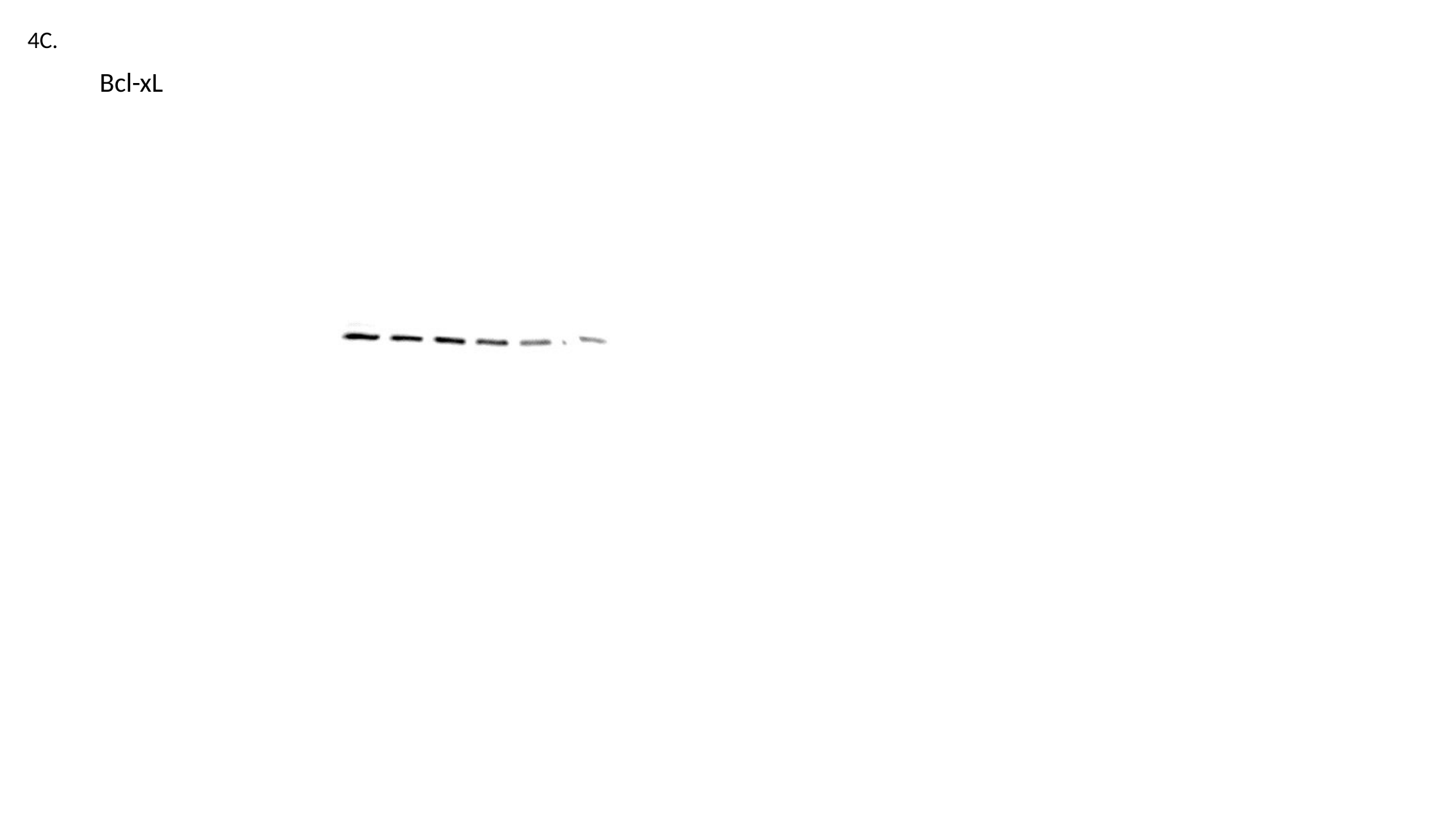

4C.
Bcl-xL

## Slide 27
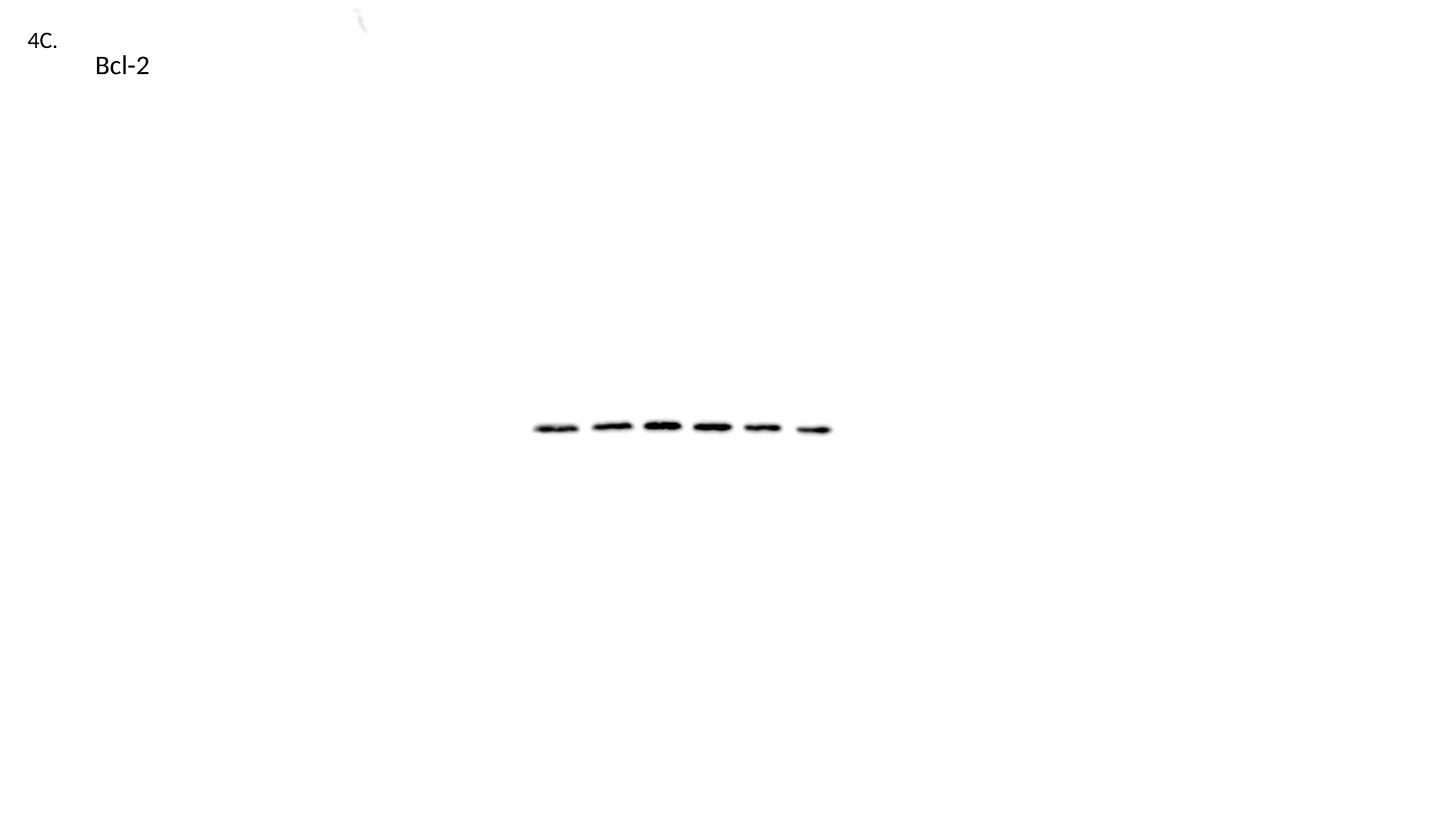

4C.
Bcl-2

## Slide 28
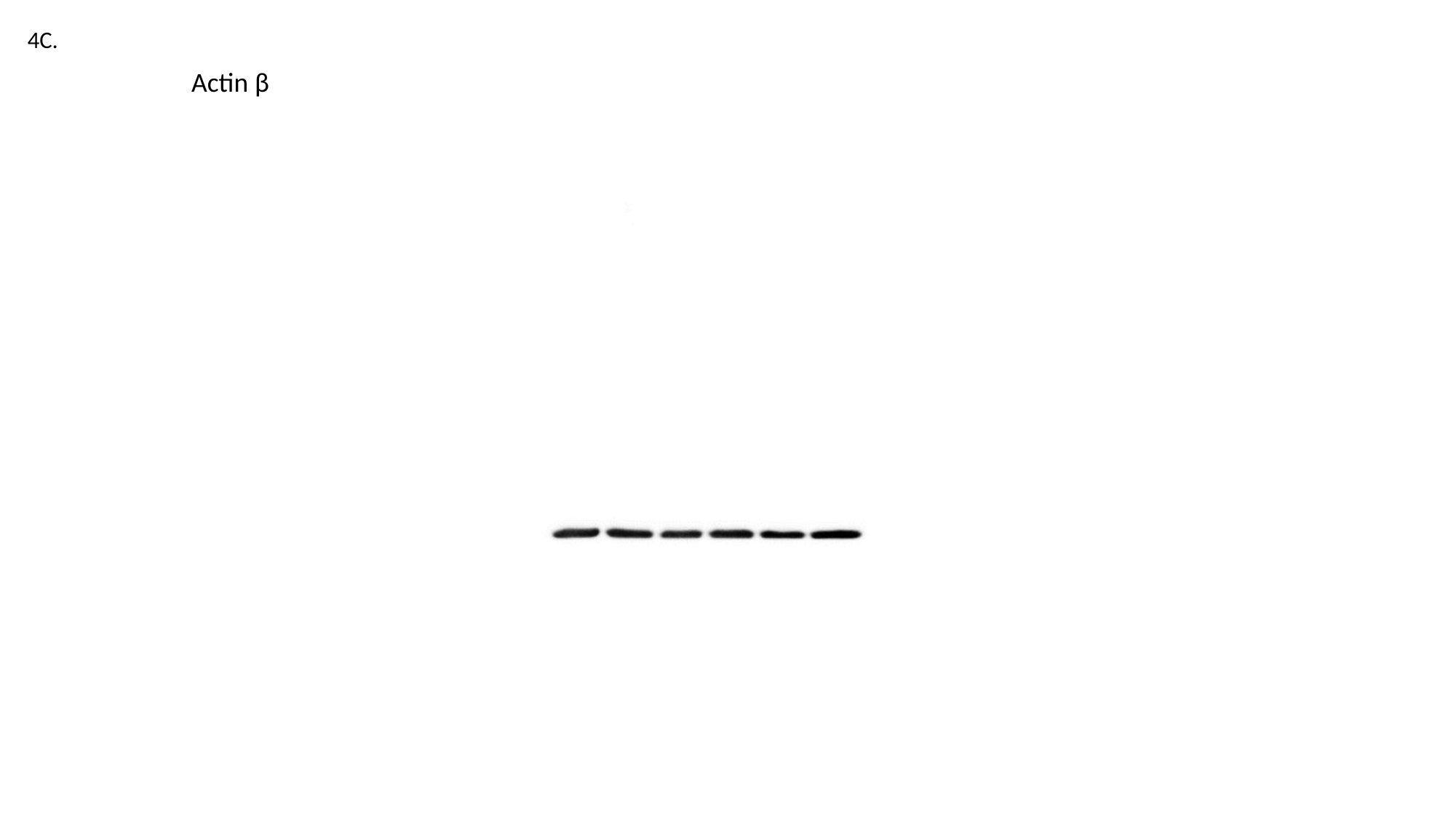

4C.
Actin β

## Slide 29
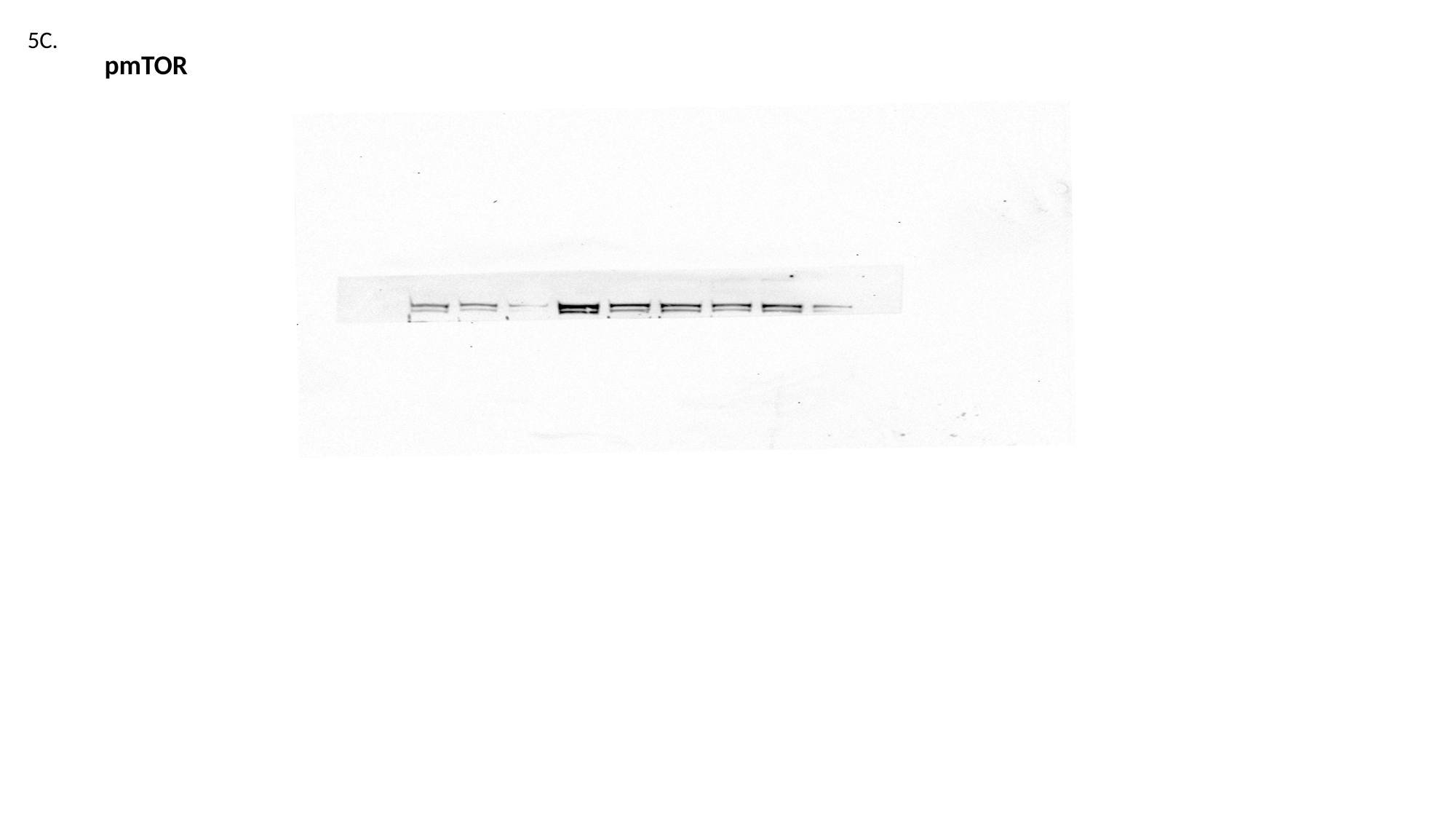

5C.
pmTOR

## Slide 30
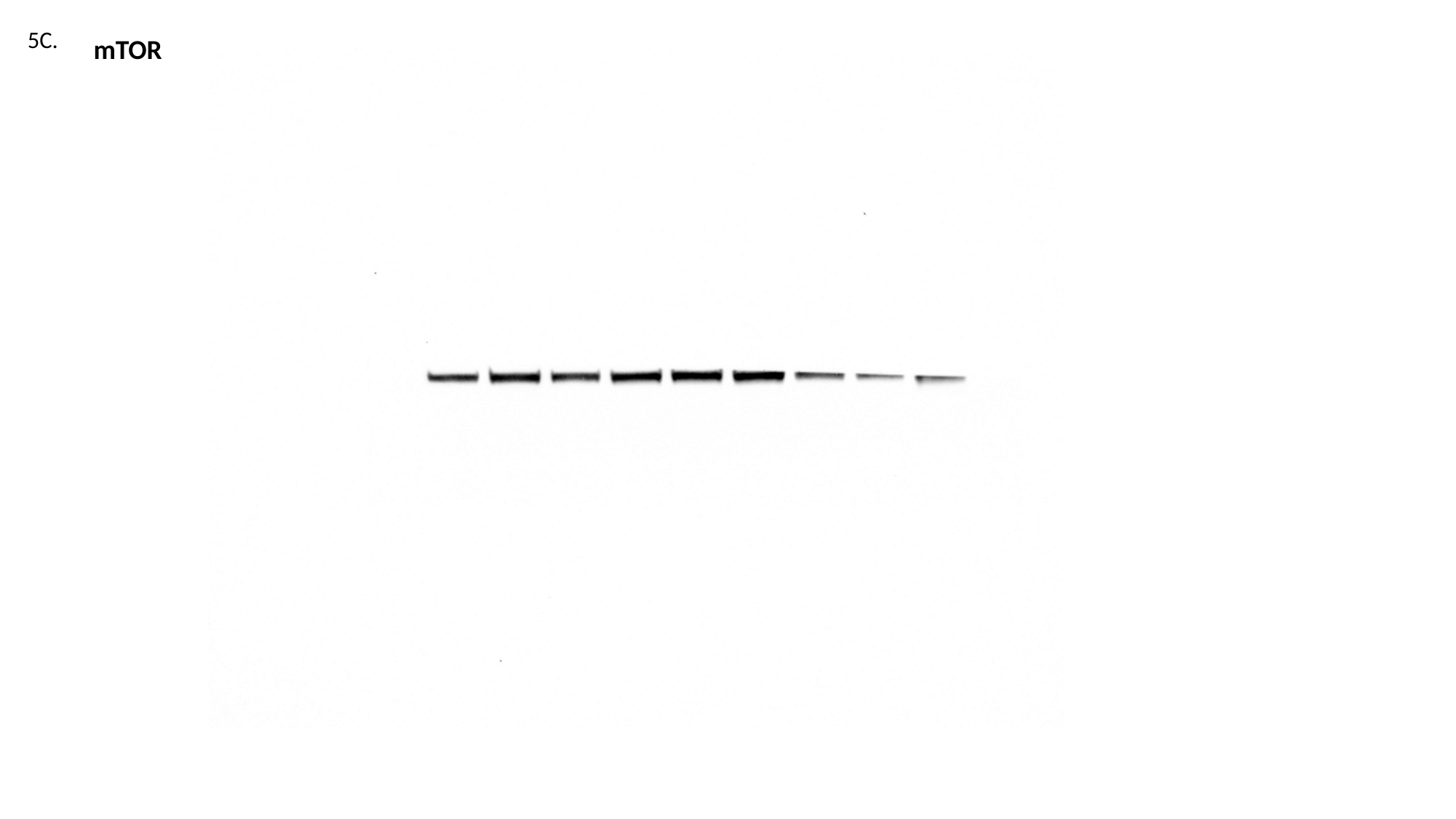

5C.
mTOR

## Slide 31
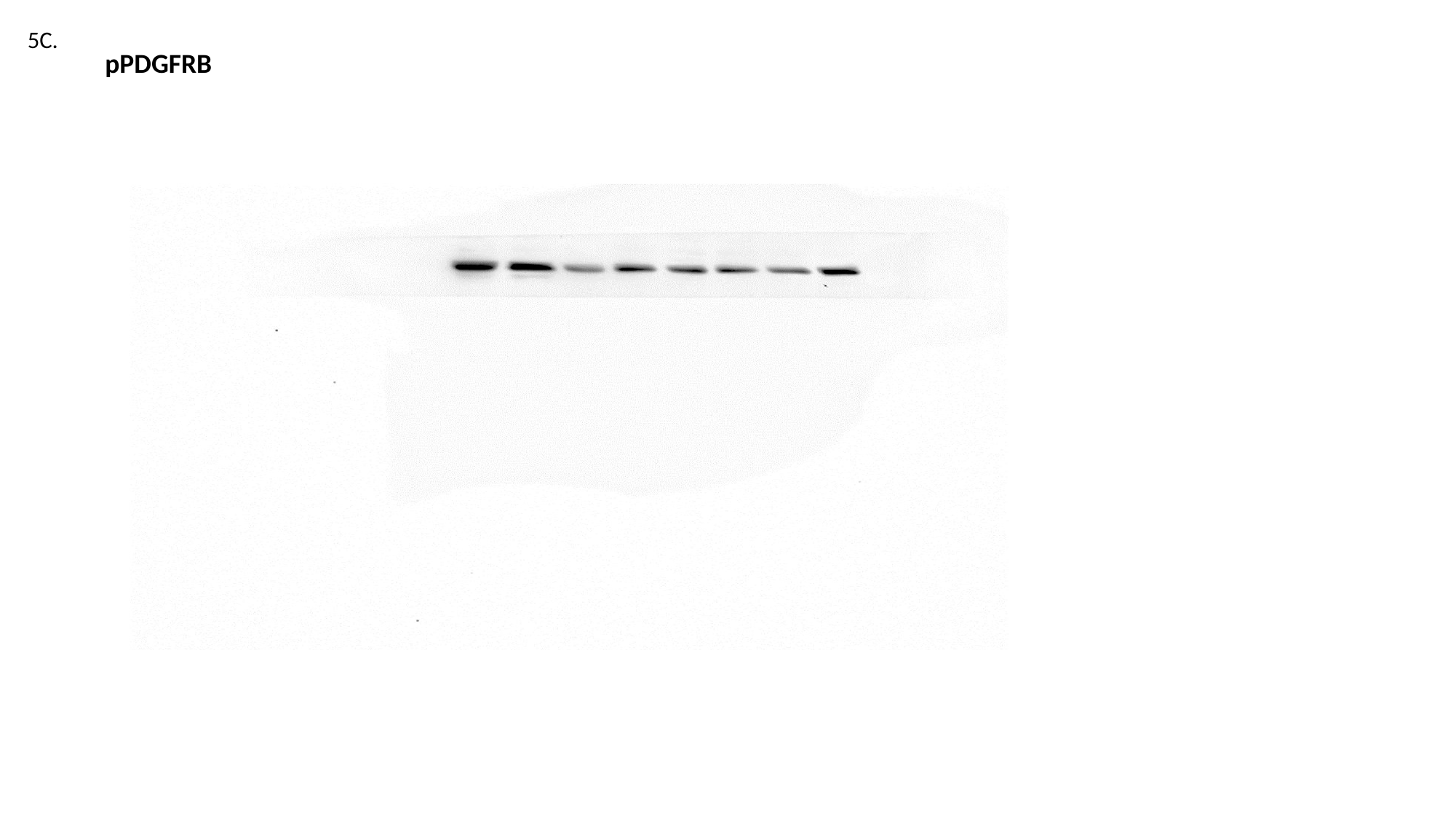

5C.
pPDGFRB

## Slide 32
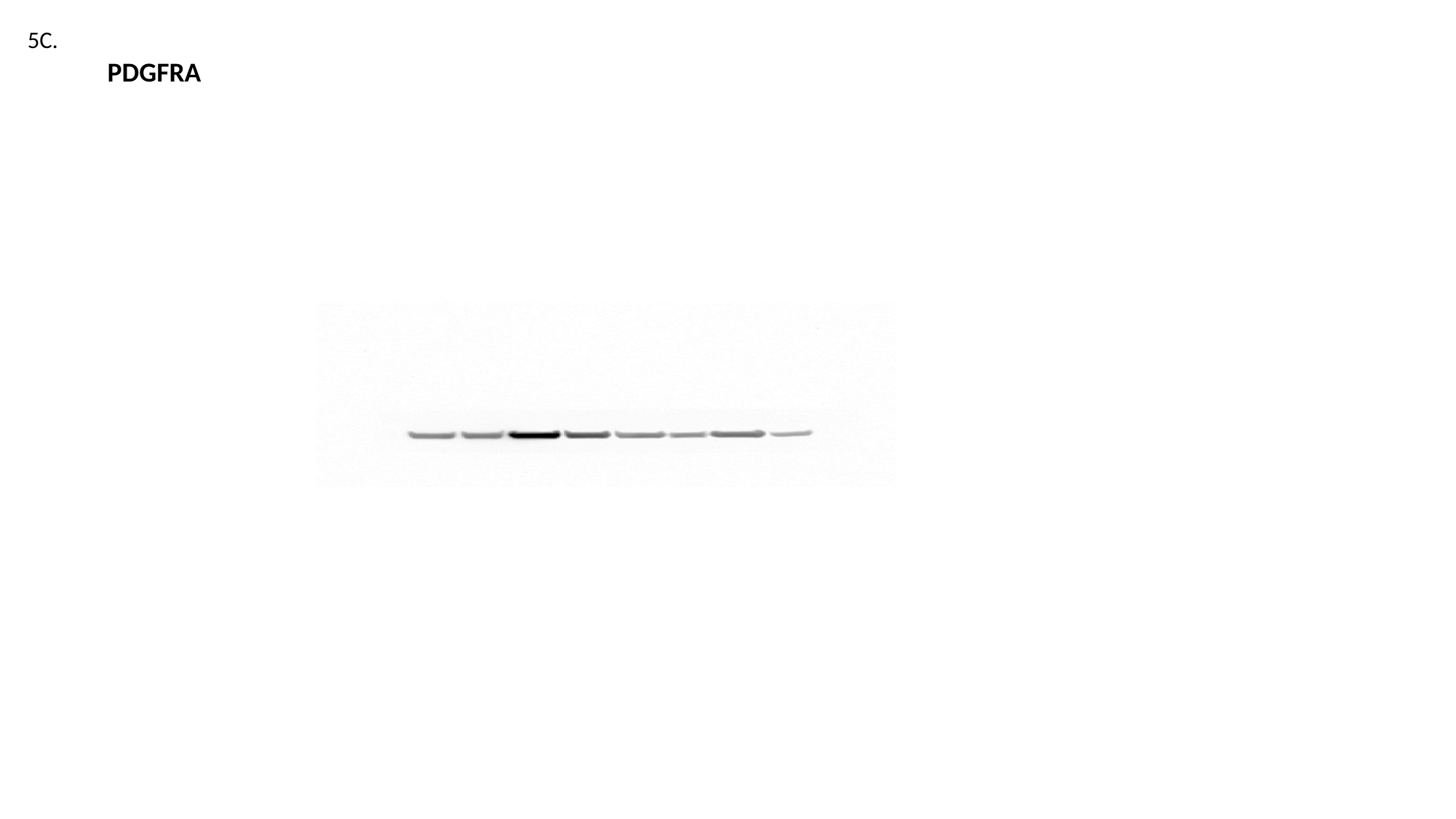

5C.
PDGFRA

## Slide 33
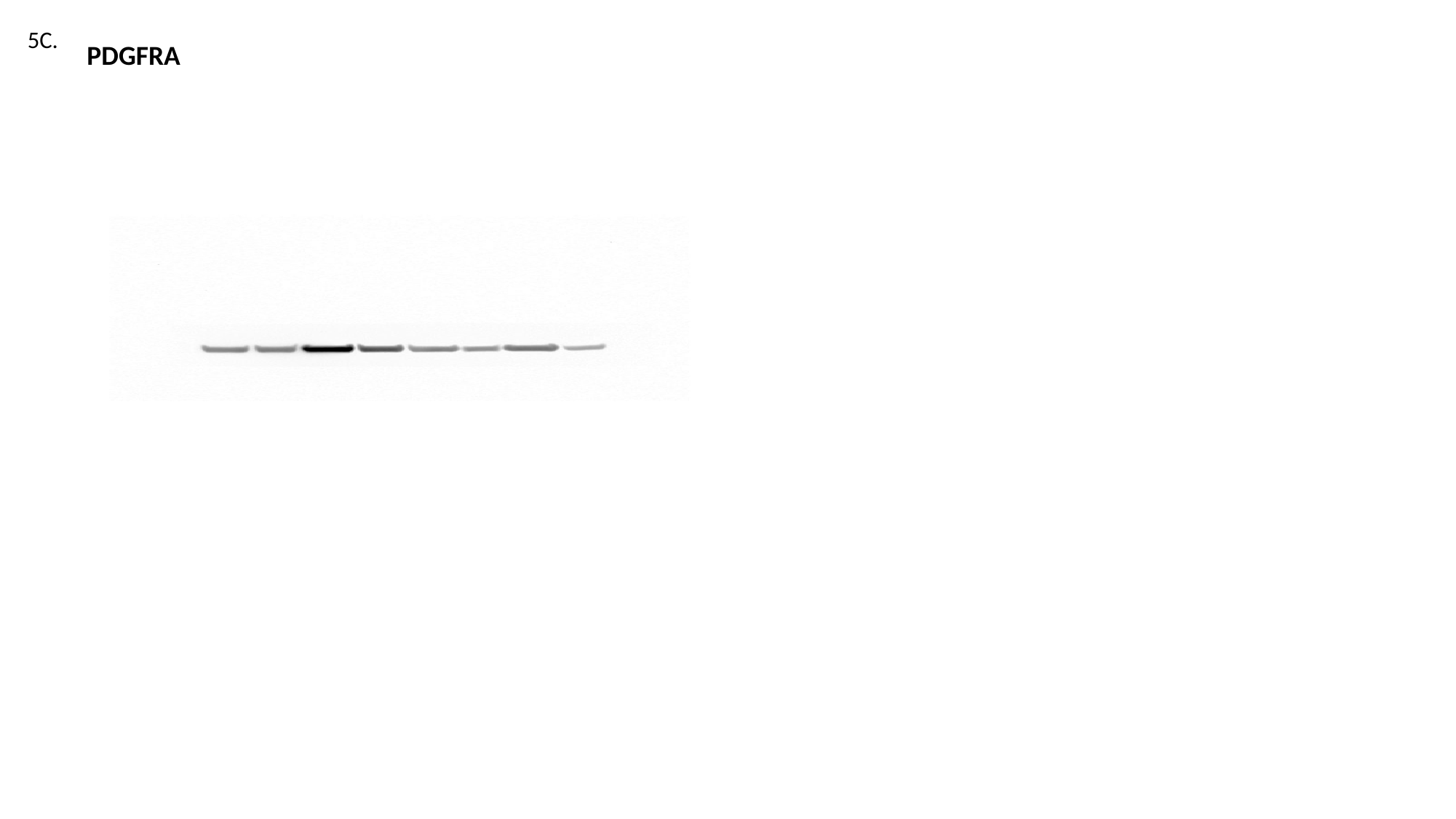

5C.
PDGFRA

## Slide 34
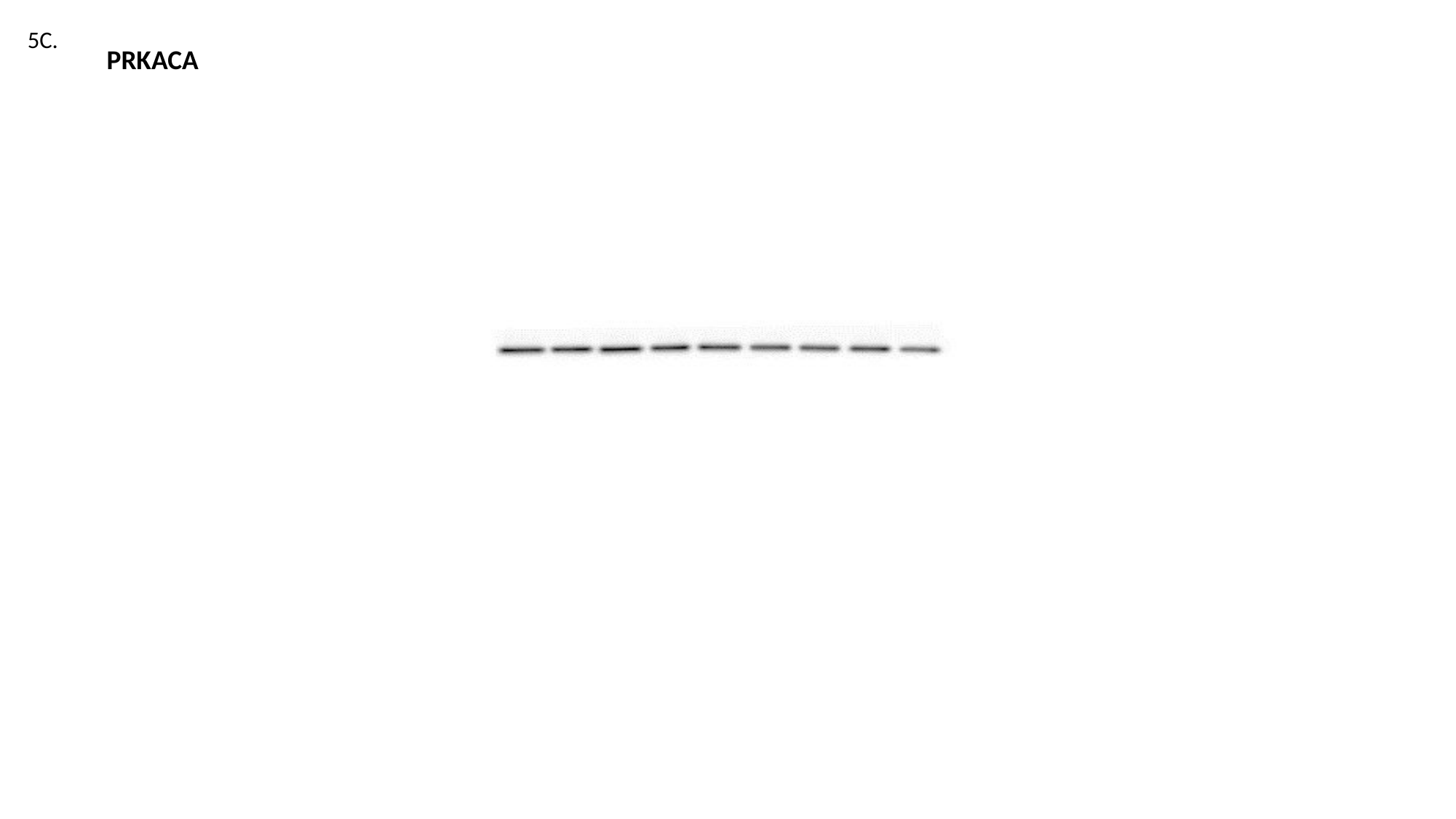

5C.
PRKACA

## Slide 35
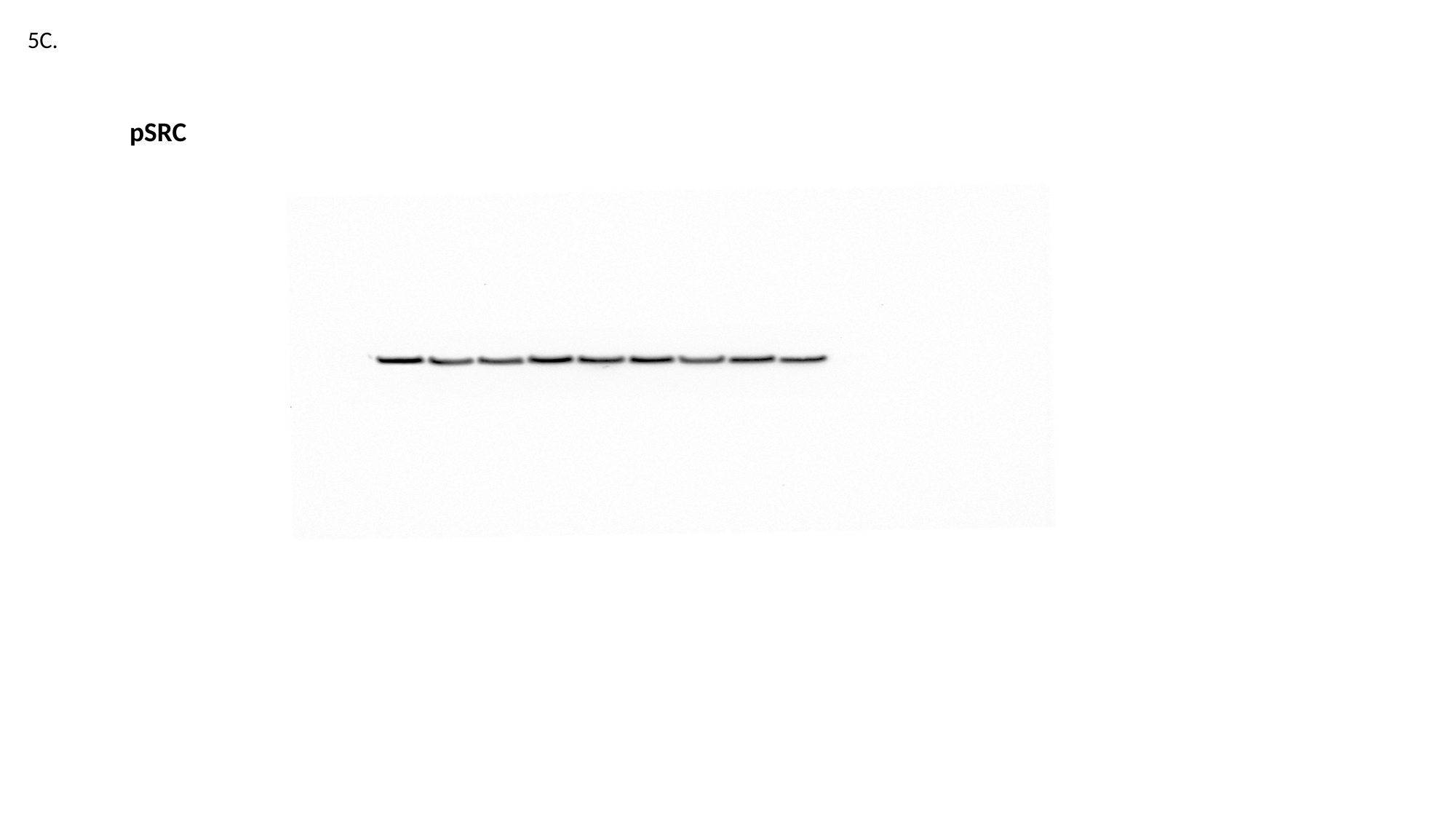

5C.
pSRC

## Slide 36
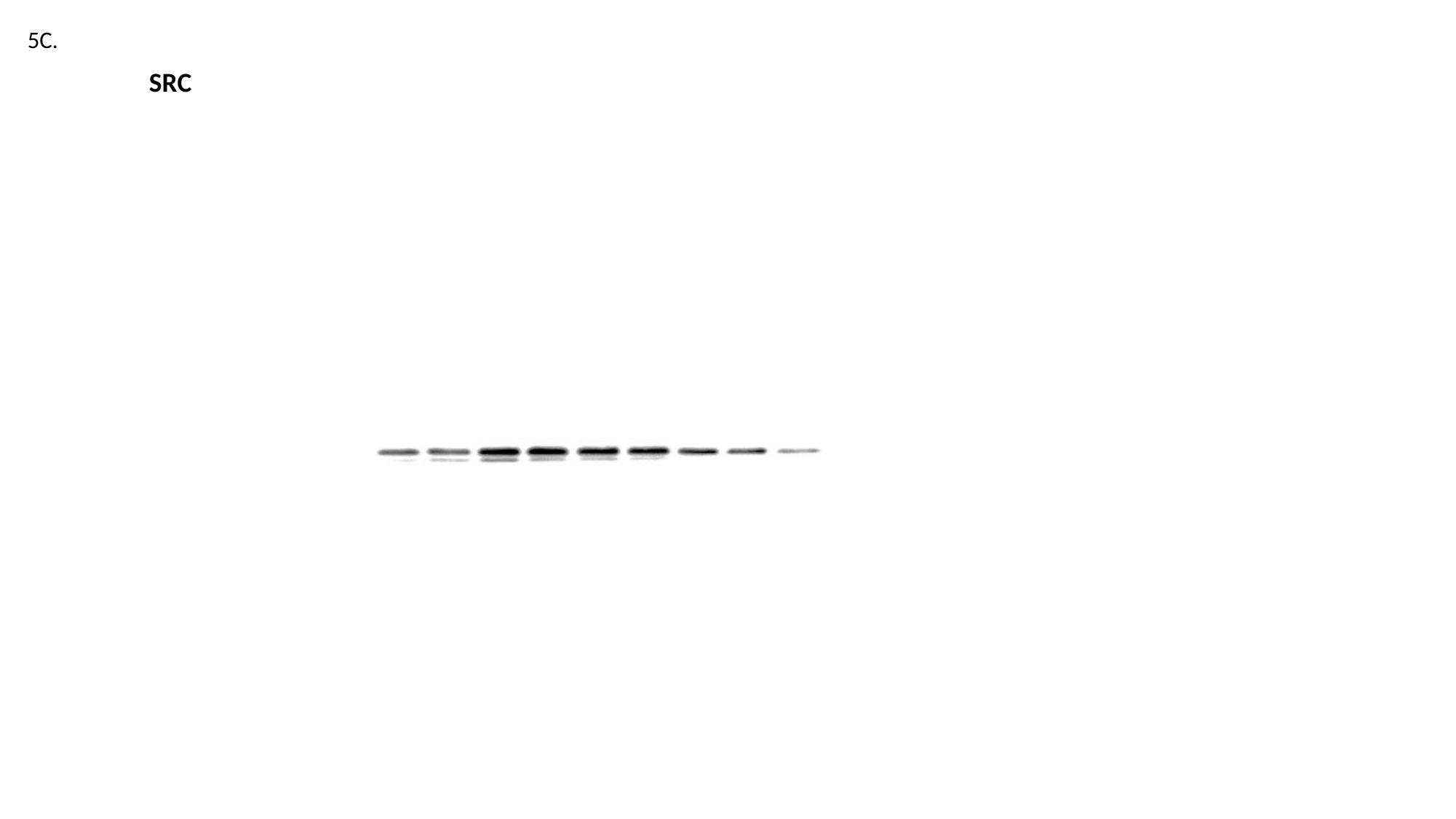

5C.
SRC

## Slide 37
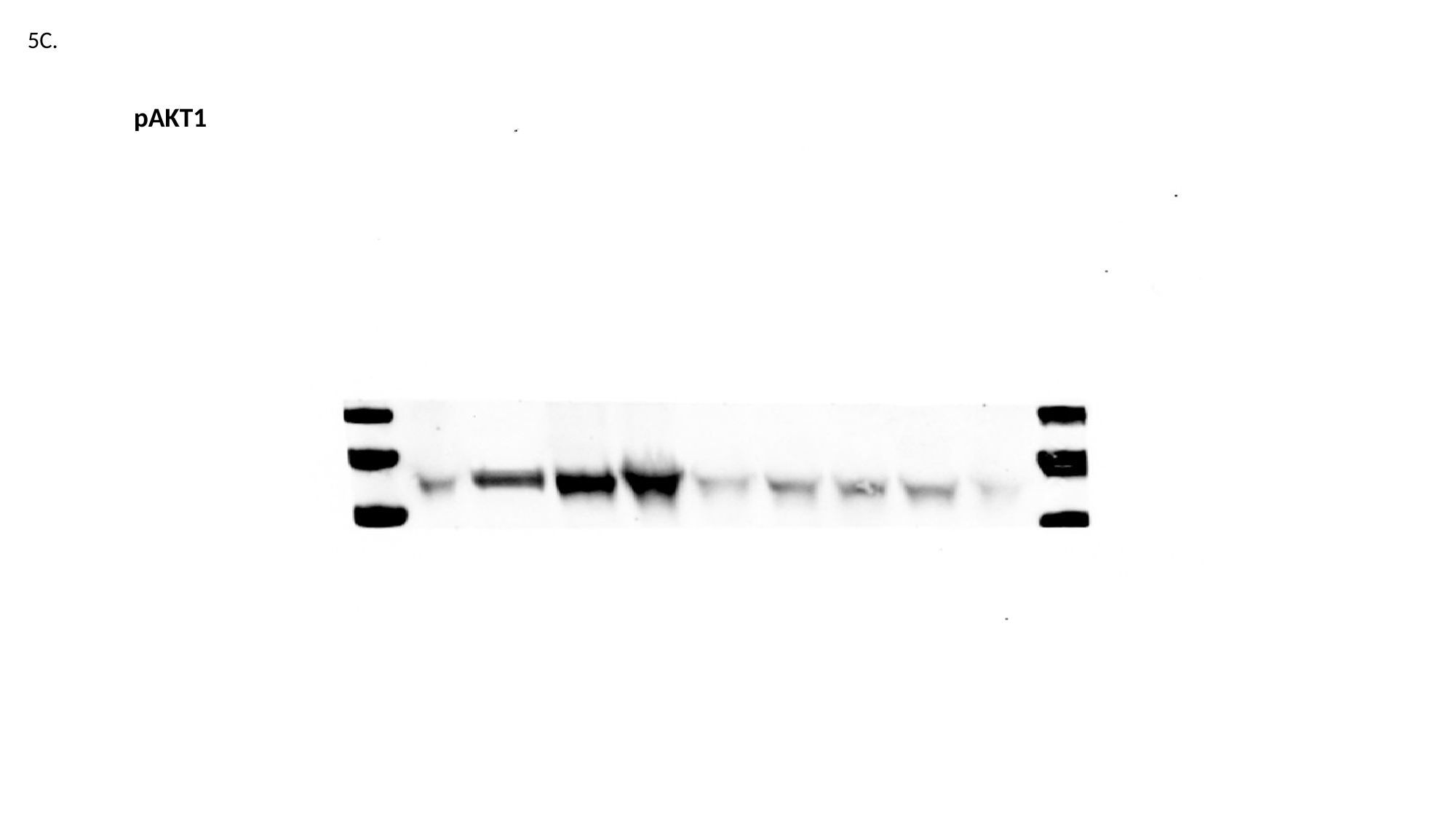

5C.
pAKT1

## Slide 38
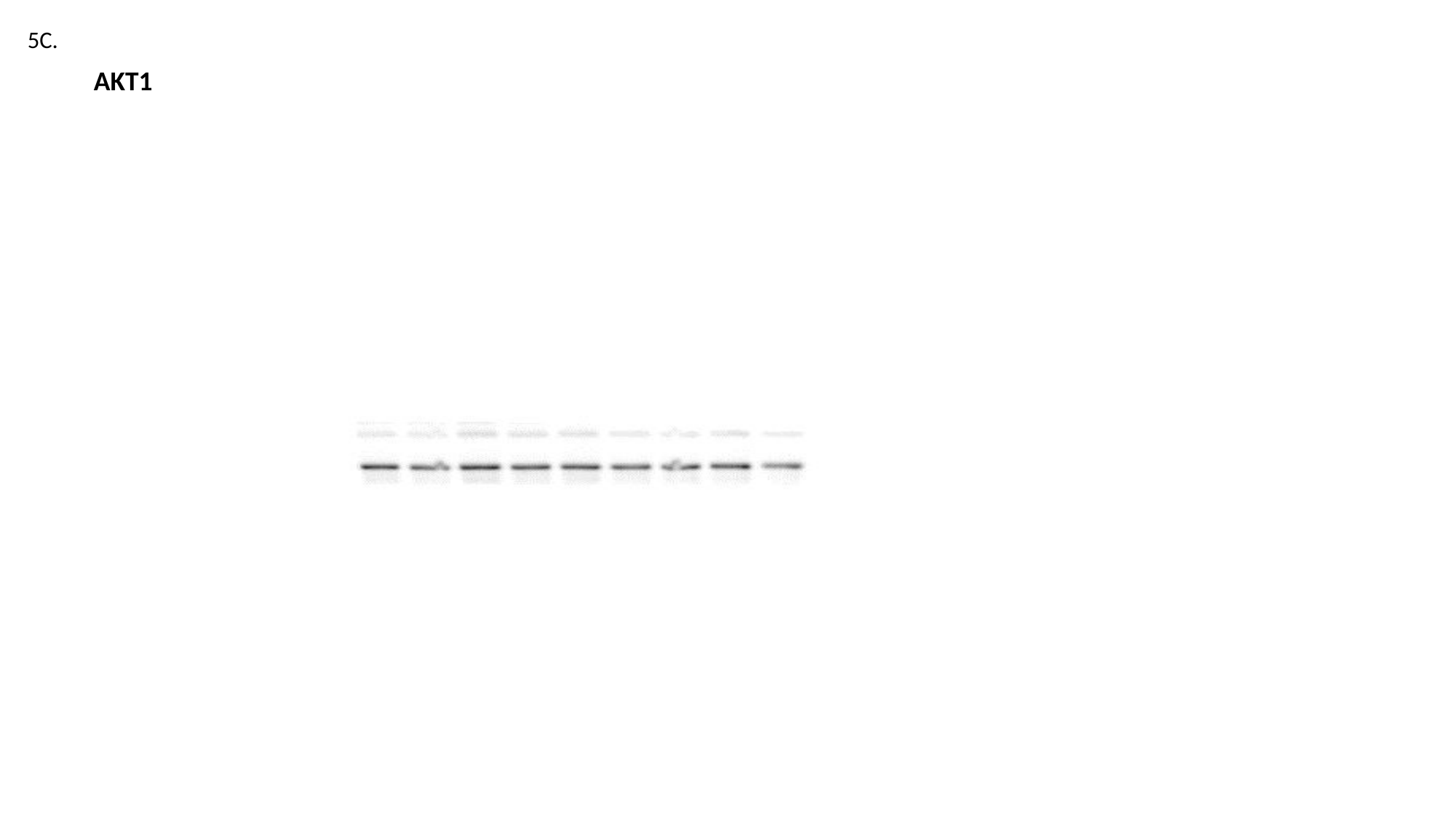

5C.
AKT1

## Slide 39
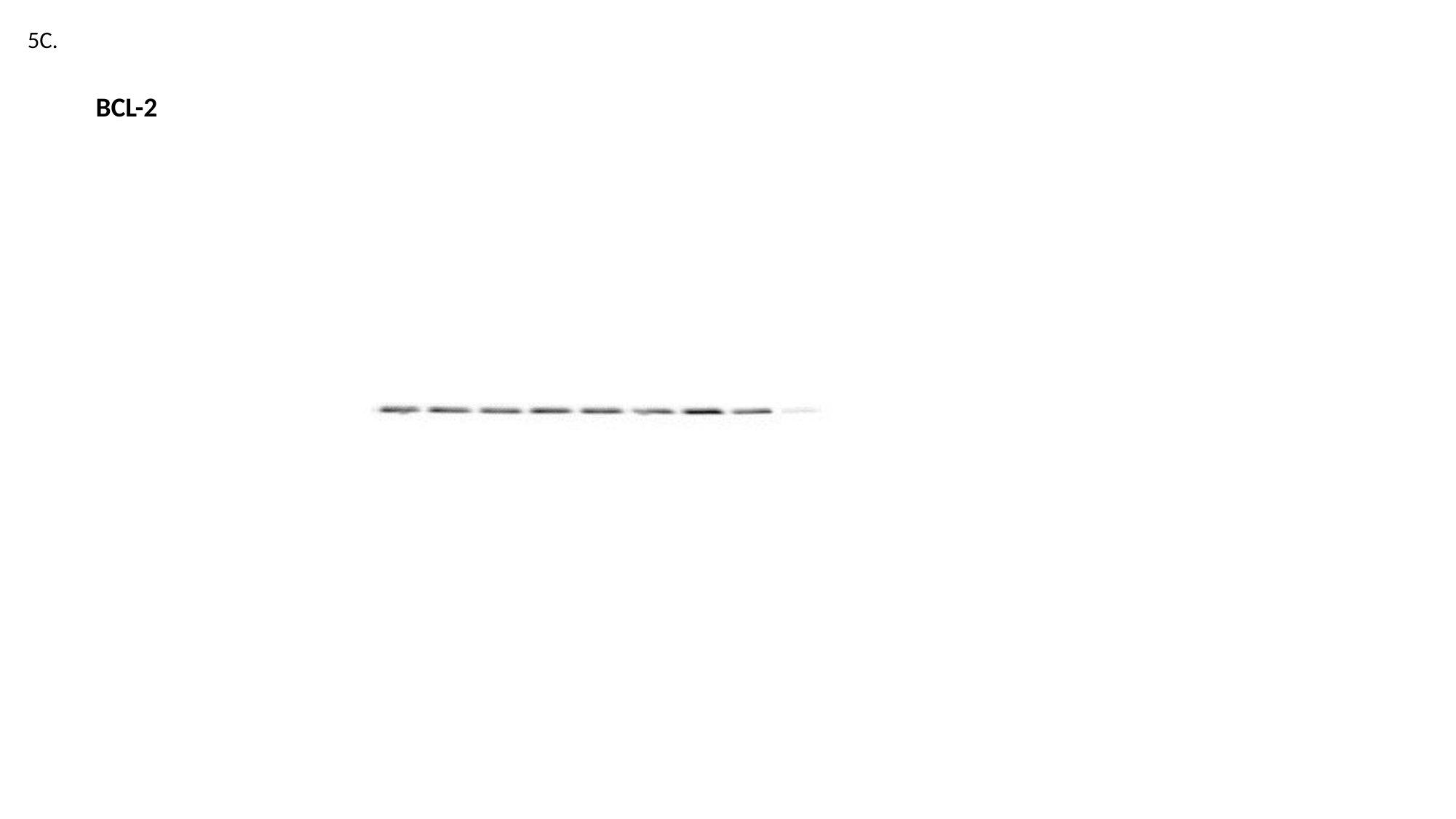

5C.
BCL-2

## Slide 40
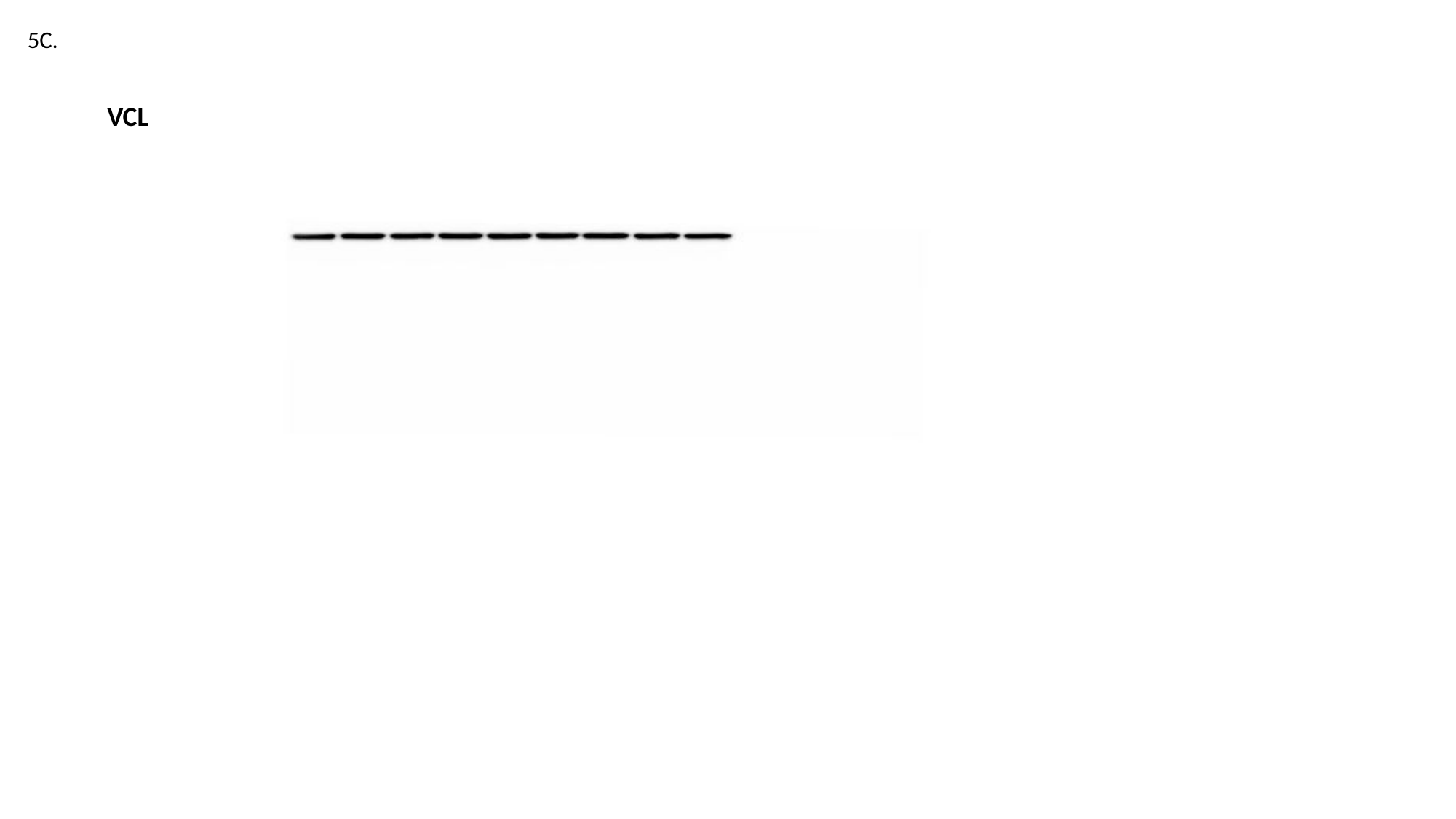

5C.
VCL

## Slide 41
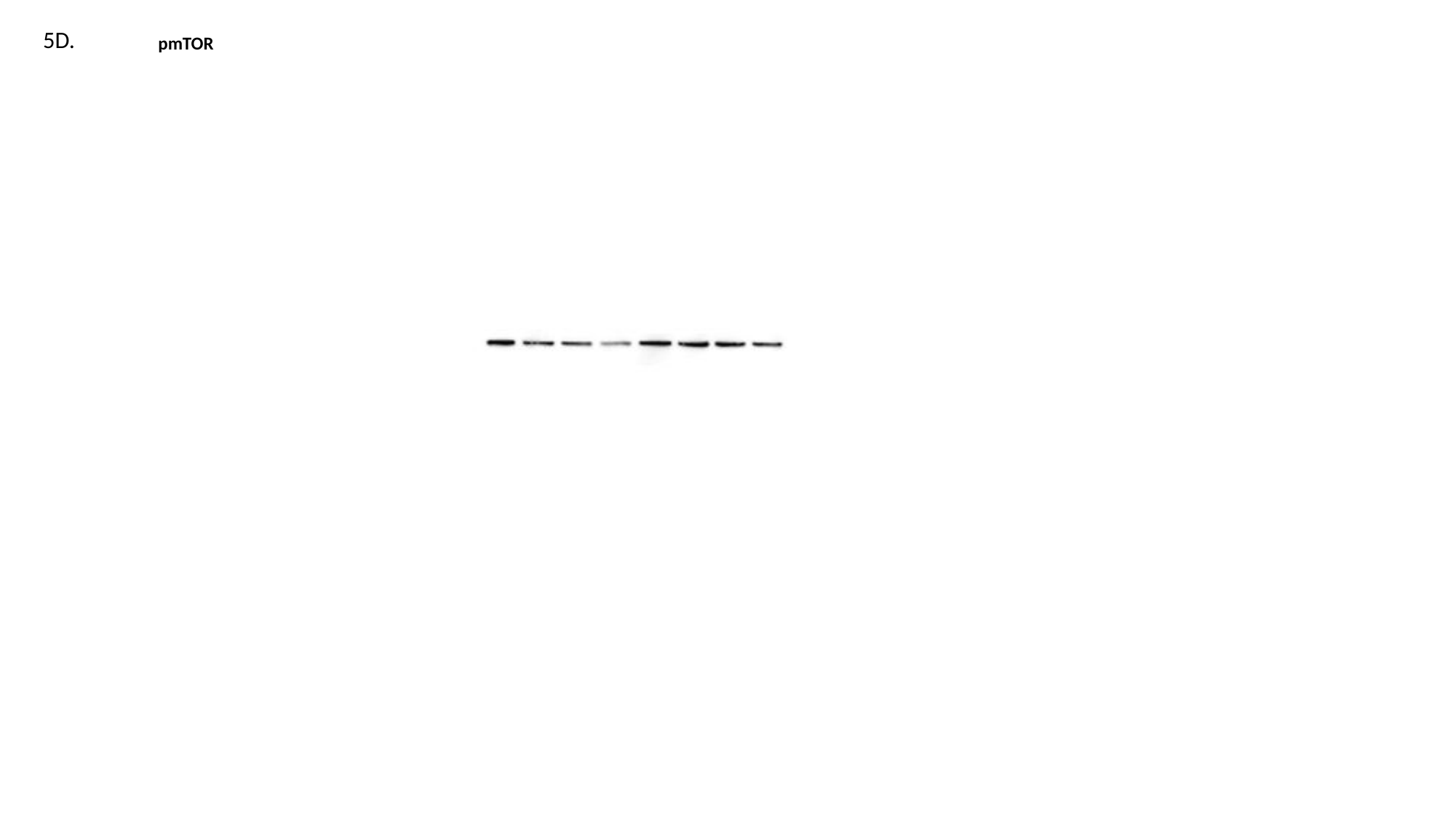

5D.
pmTOR

## Slide 42
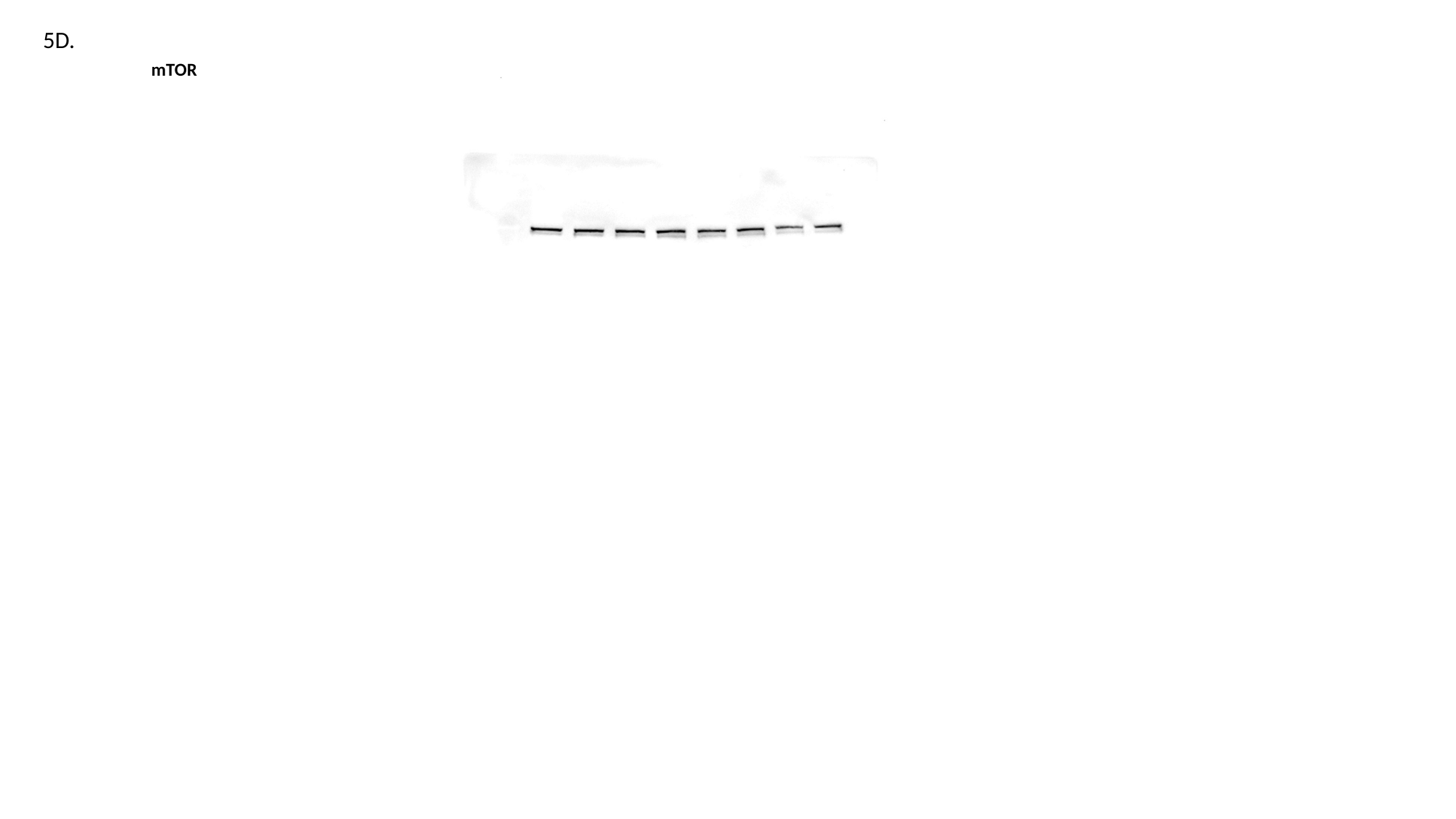

5D.
mTOR

## Slide 43
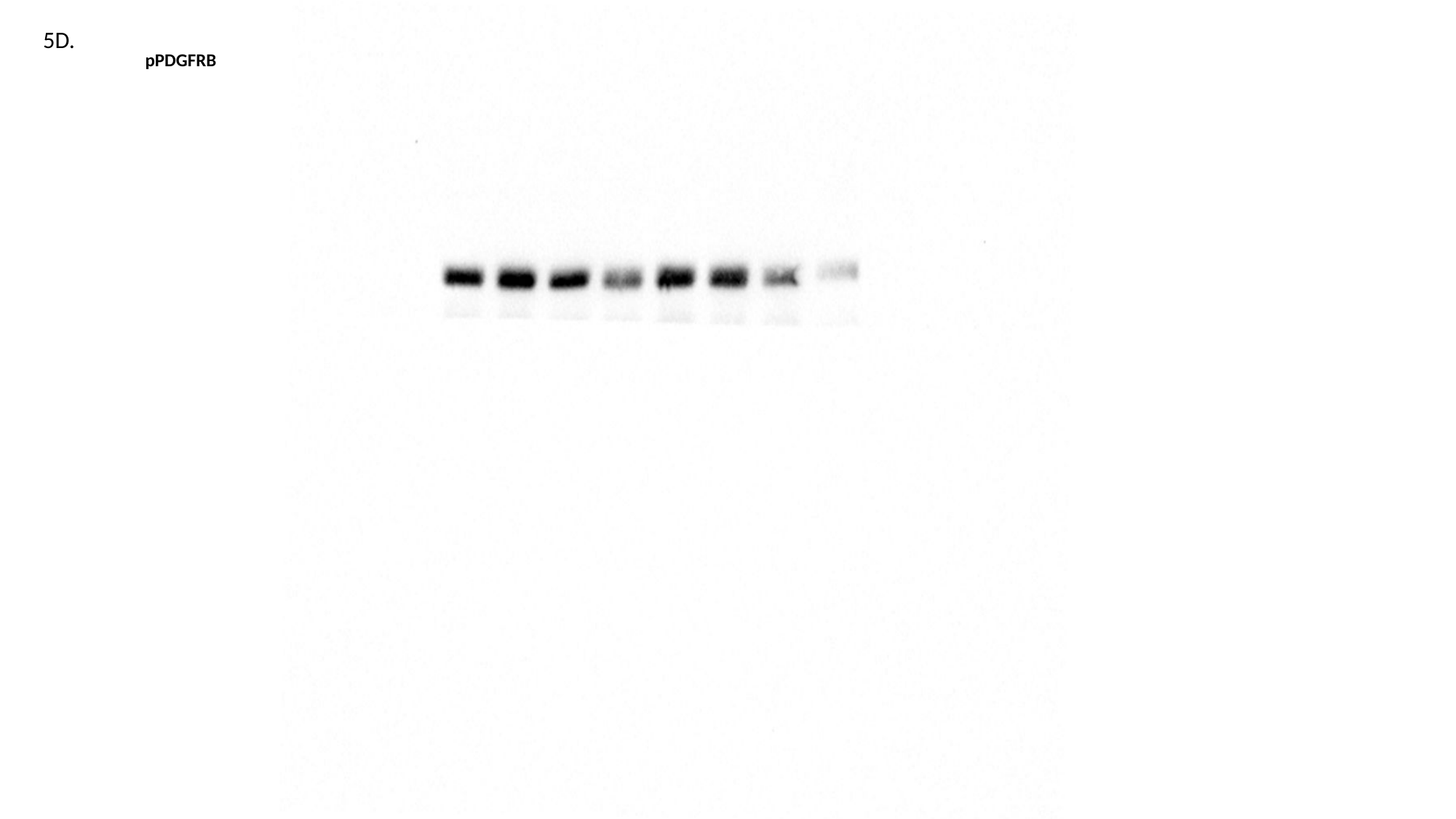

5D.
pPDGFRB

## Slide 44
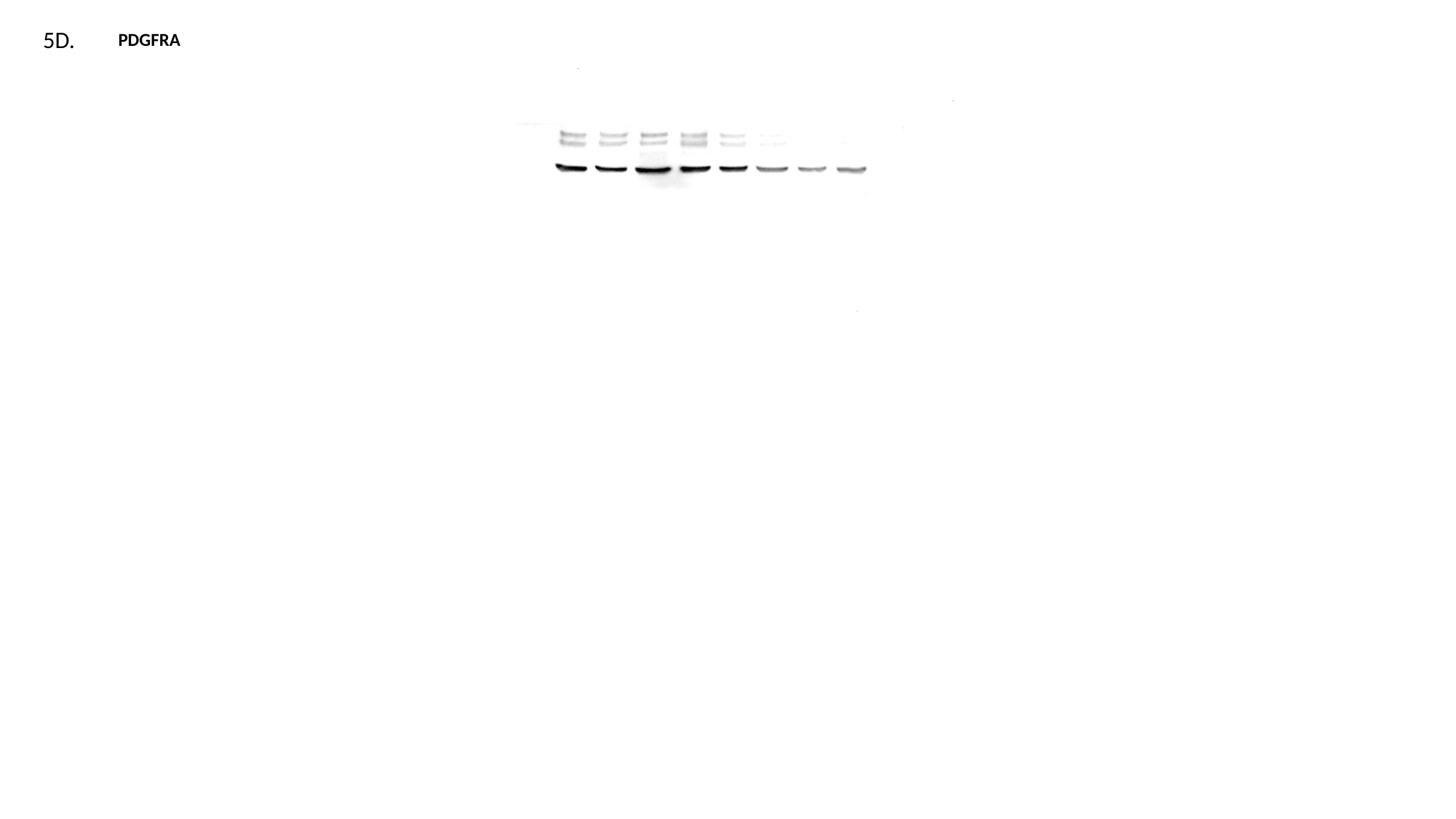

5D.
PDGFRA

## Slide 45
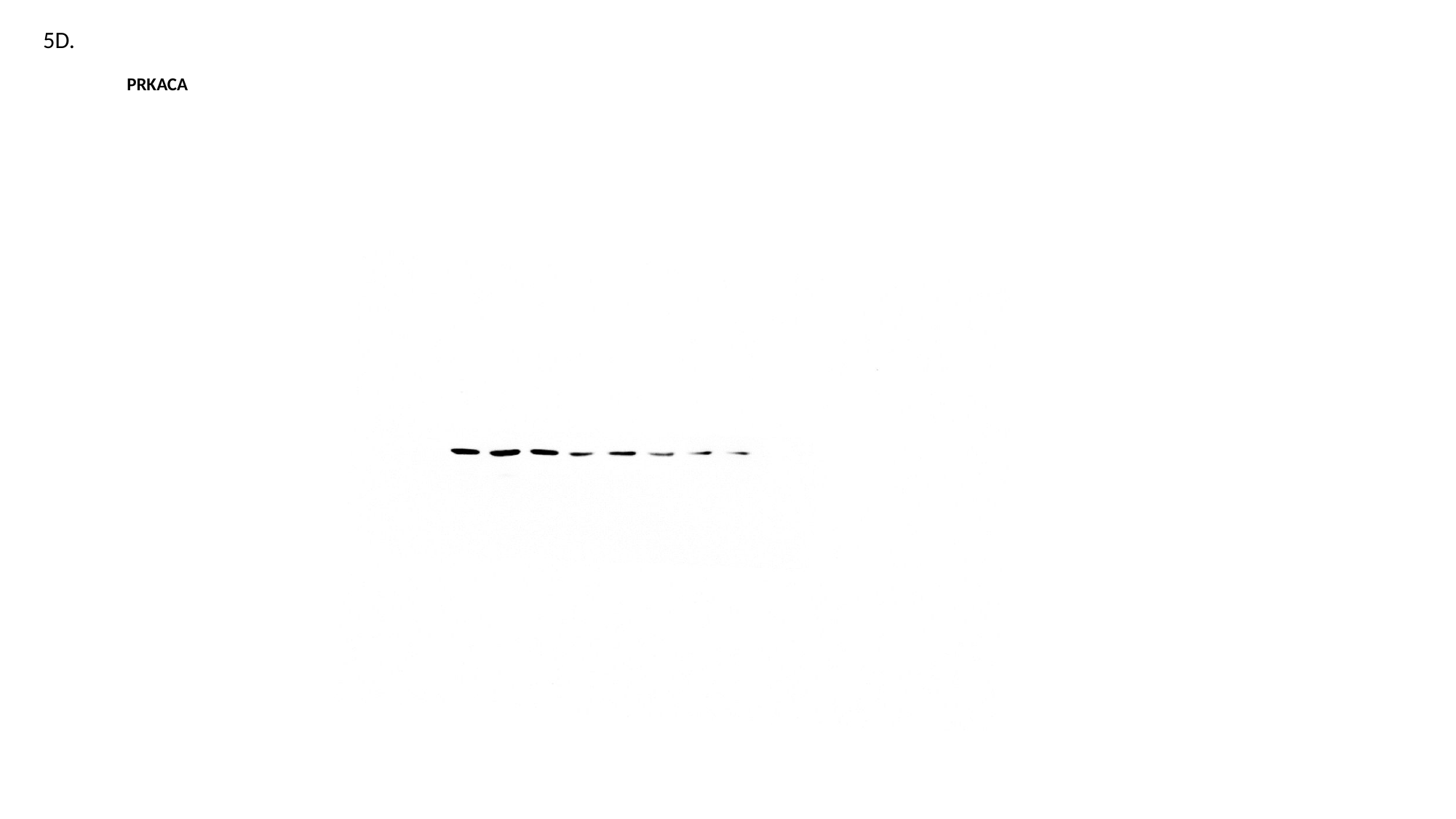

5D.
PRKACA

## Slide 46
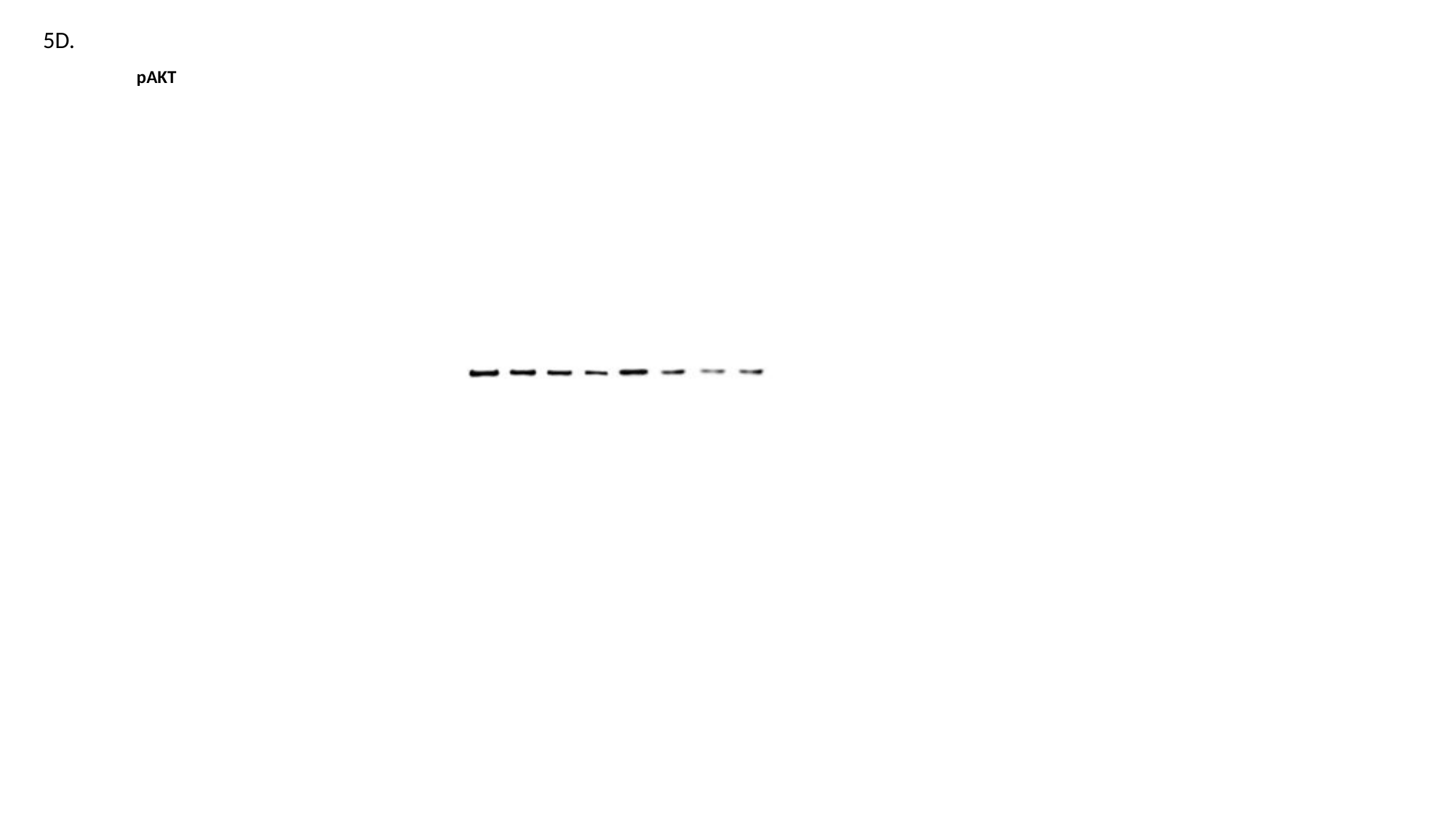

5D.
pAKT

## Slide 47
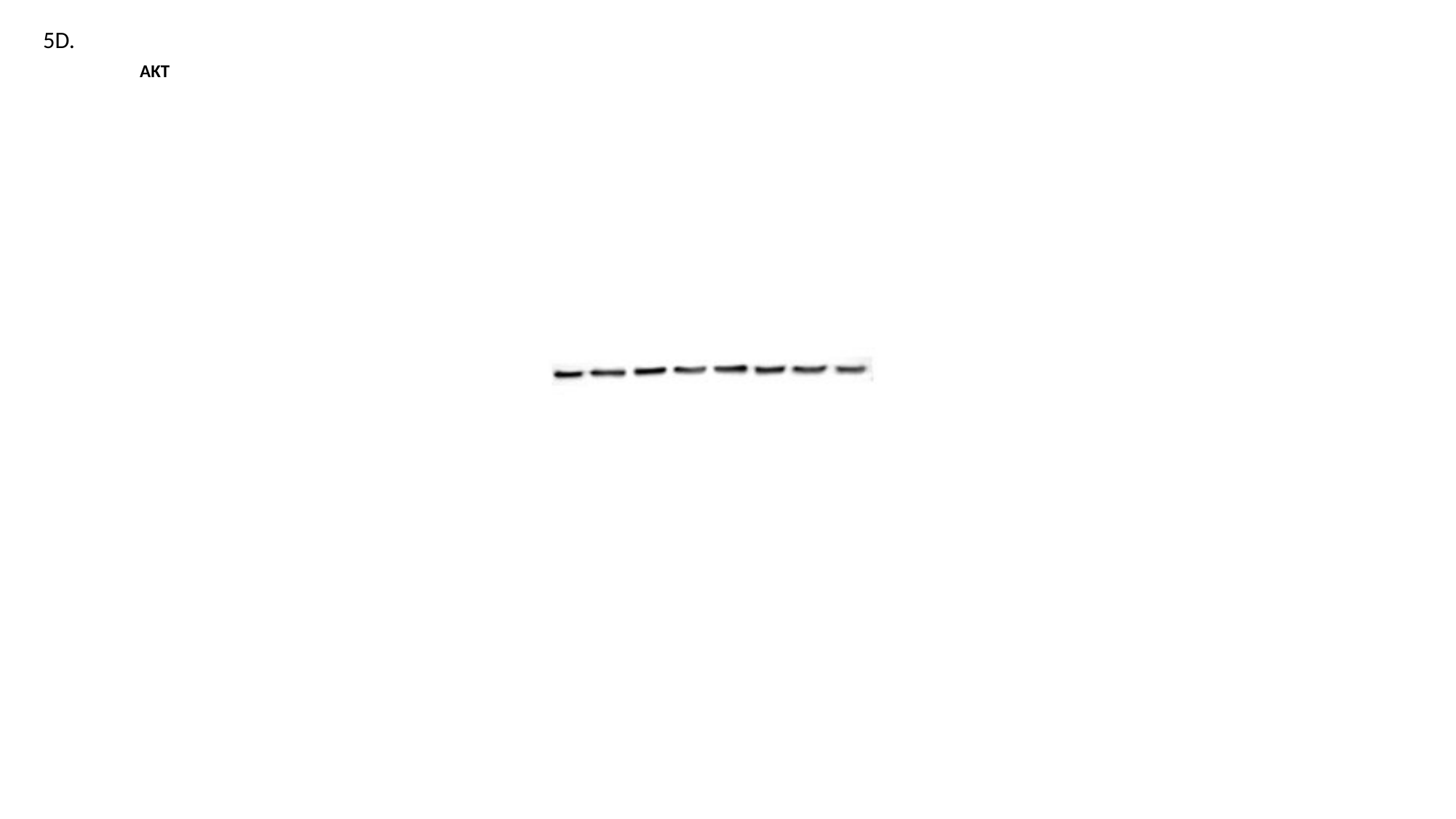

5D.
AKT

## Slide 48
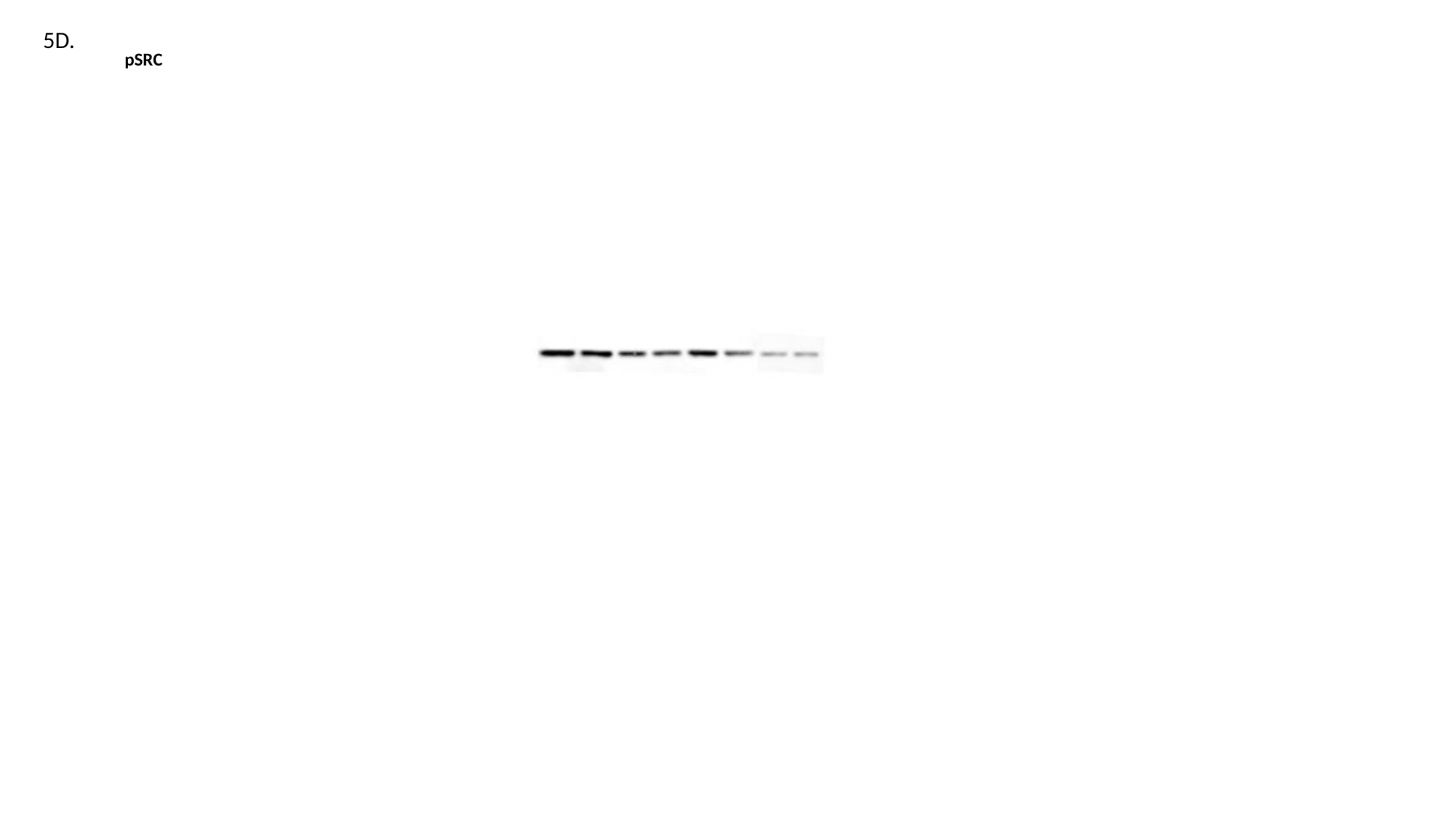

5D.
pSRC

## Slide 49
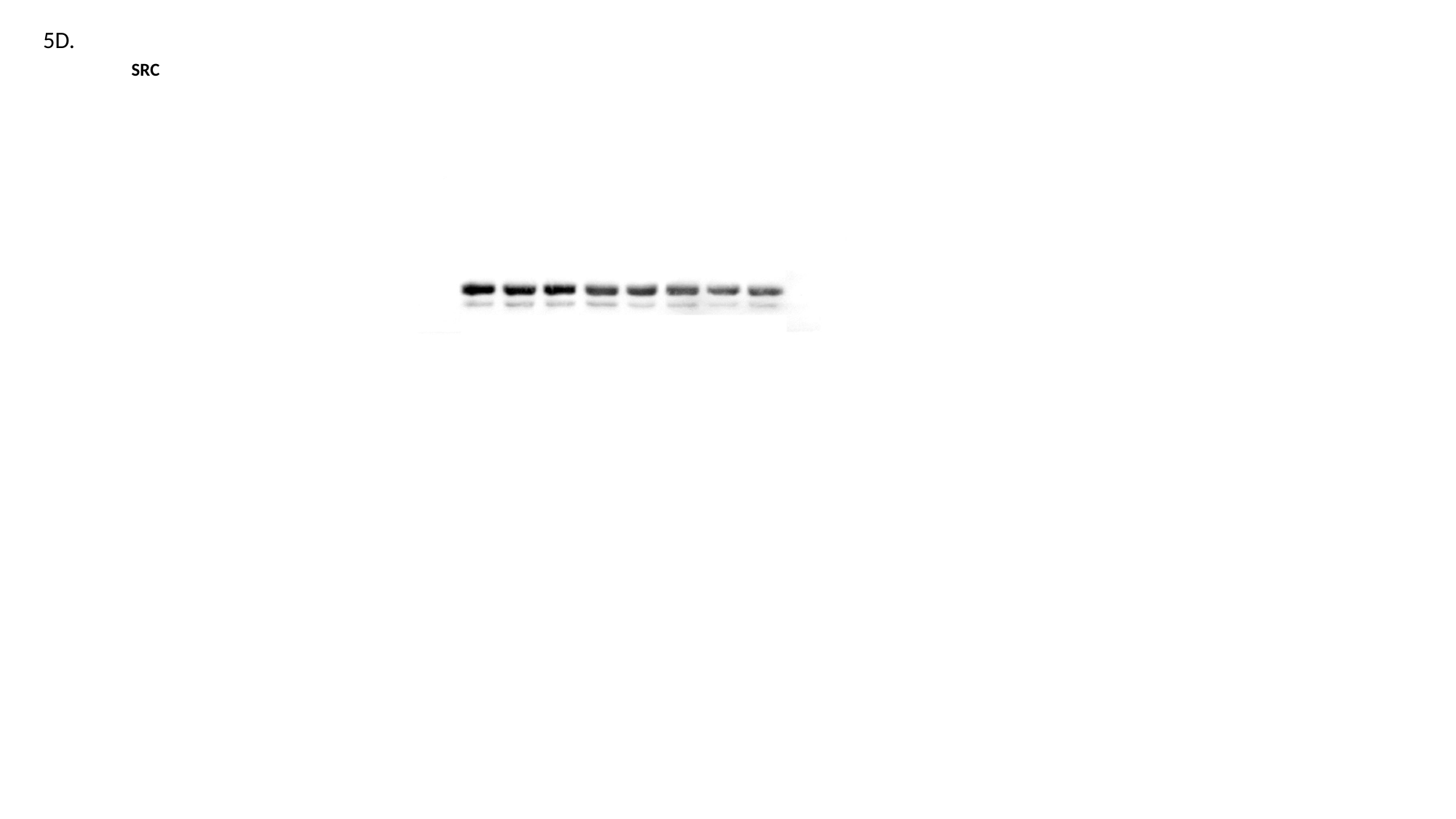

5D.
SRC

## Slide 50
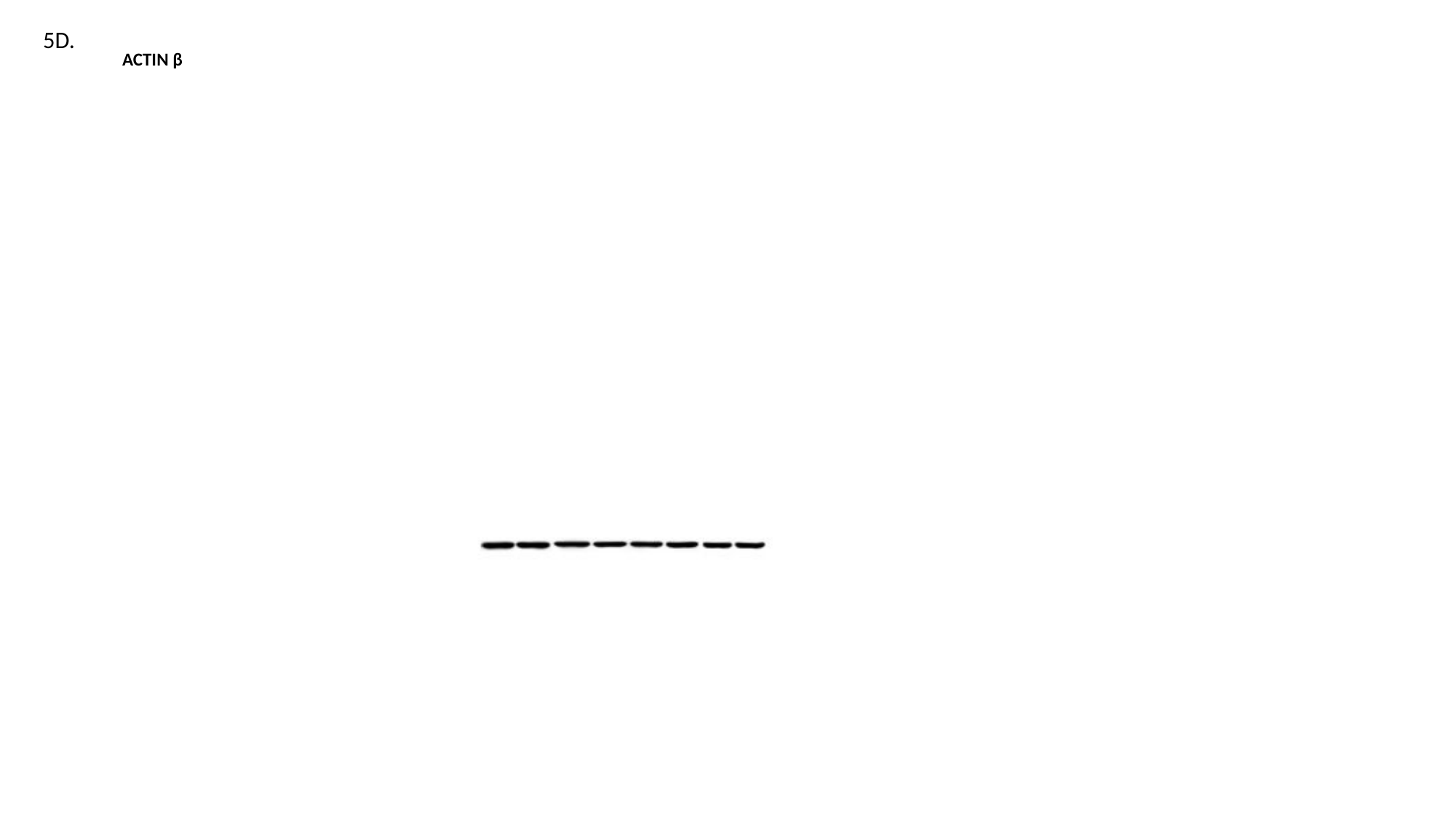

5D.
ACTIN β

## Slide 51
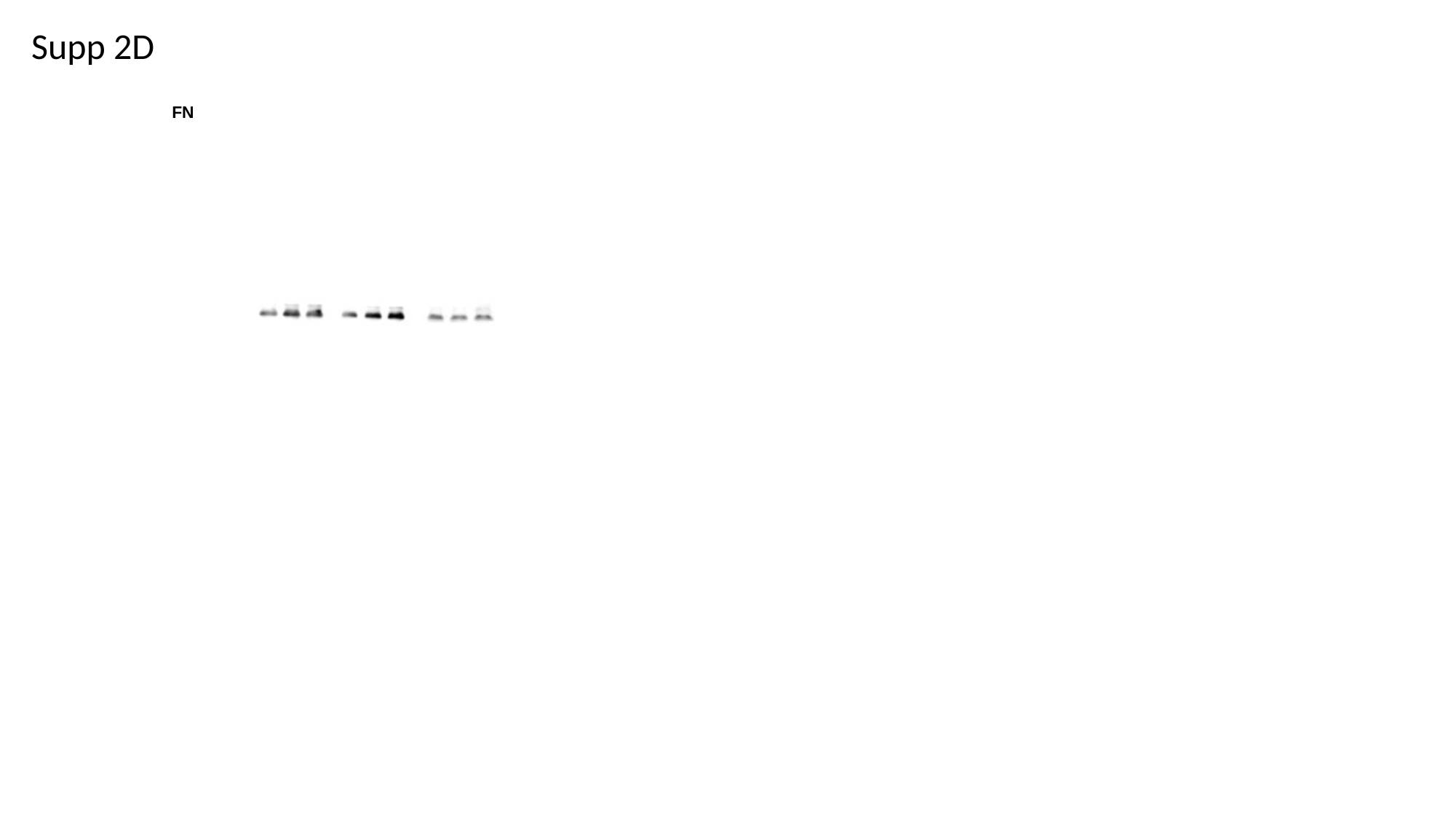

Supp 2D
FN

## Slide 52
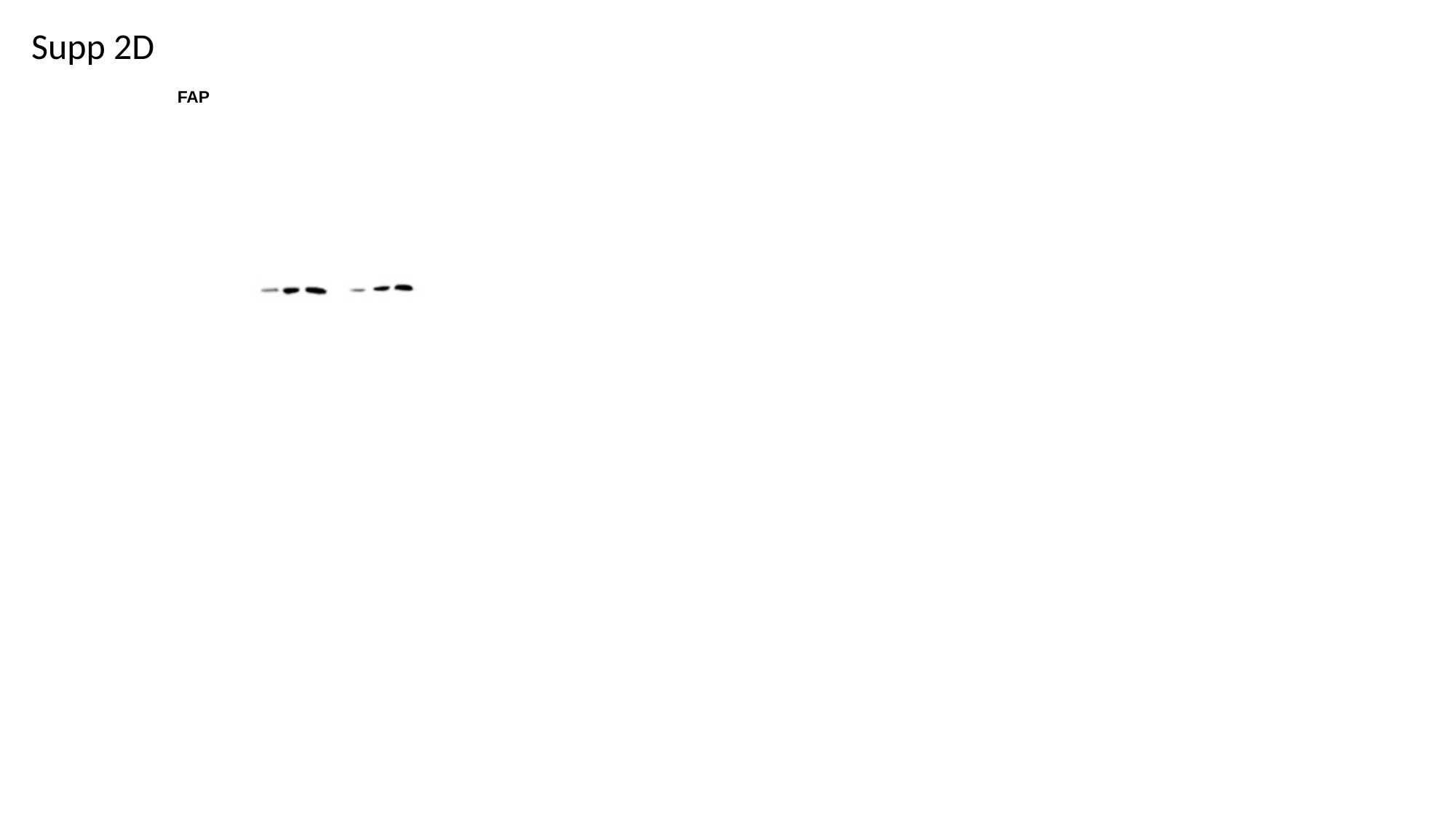

Supp 2D
FAP

## Slide 53
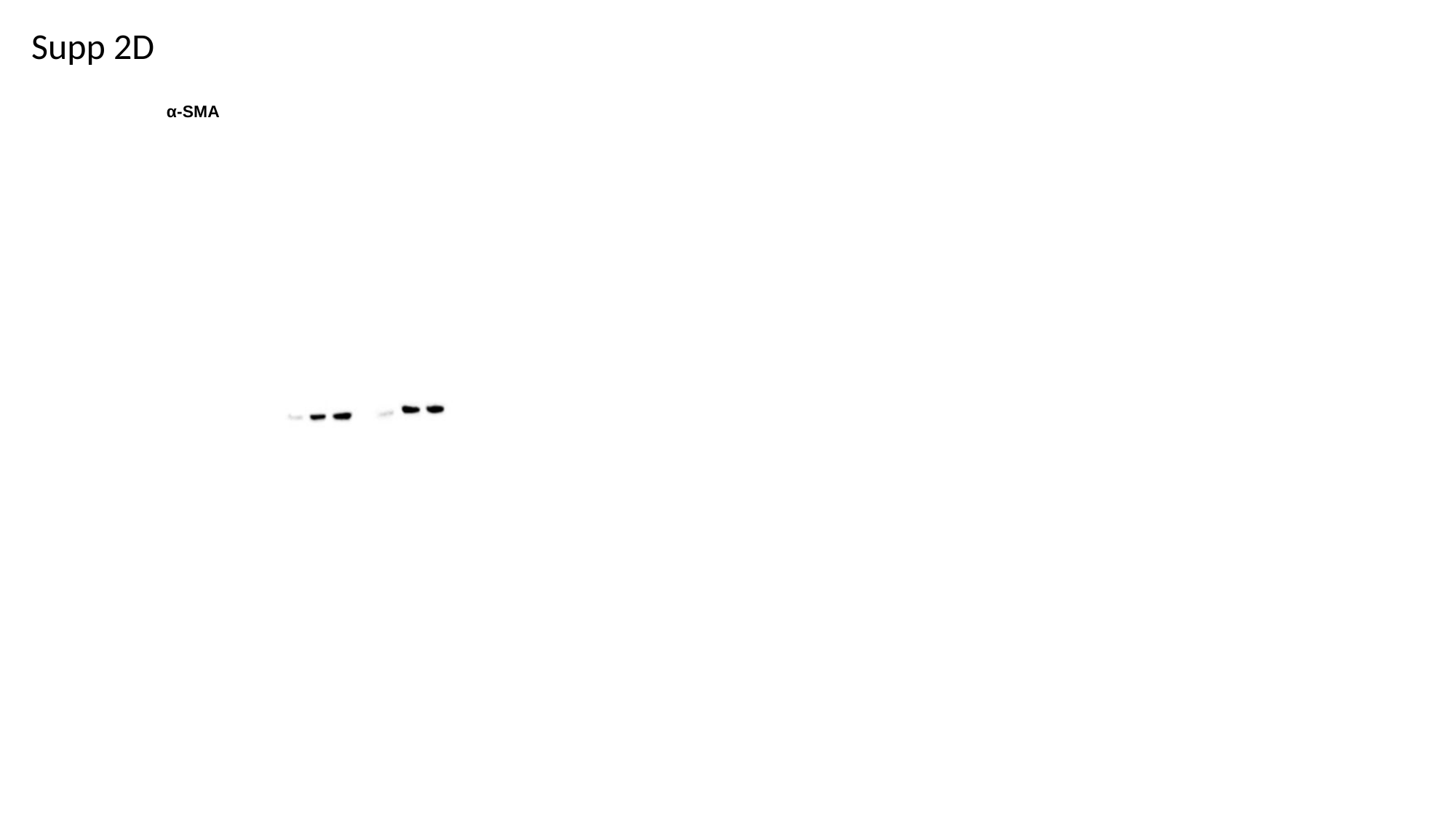

Supp 2D
α-SMA

## Slide 54
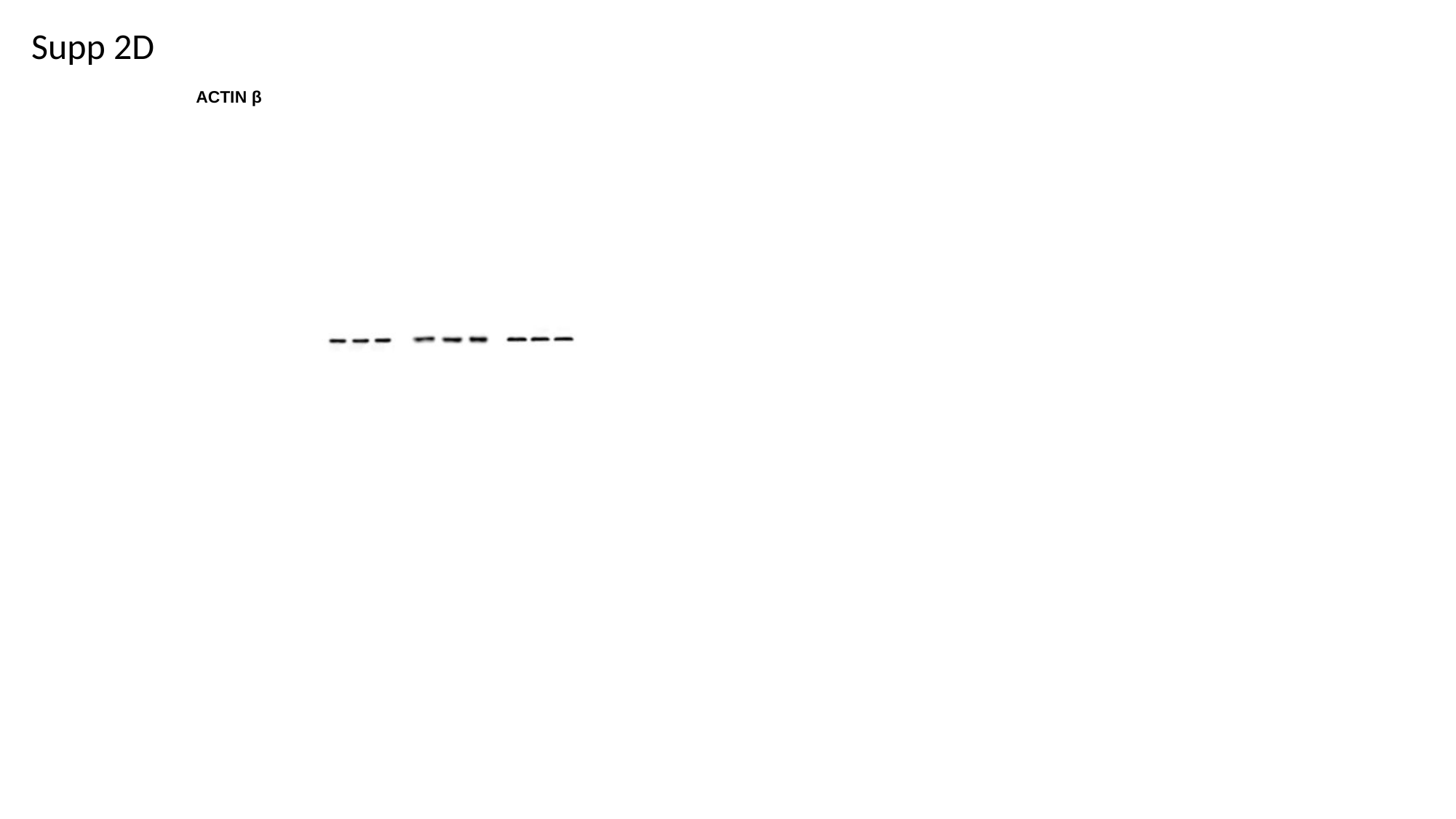

Supp 2D
ACTIN β

## Slide 55
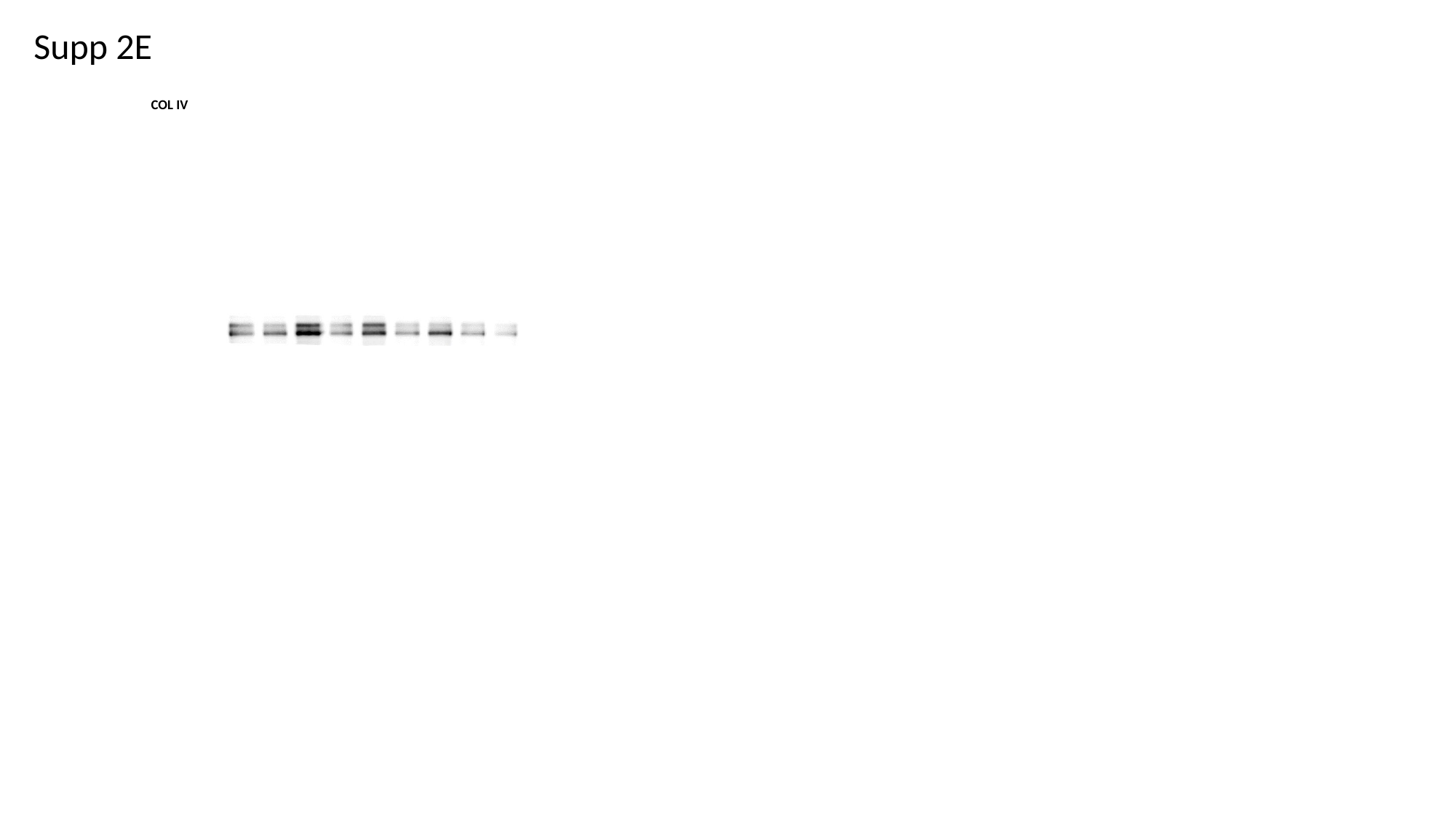

Supp 2E
COL IV

## Slide 56
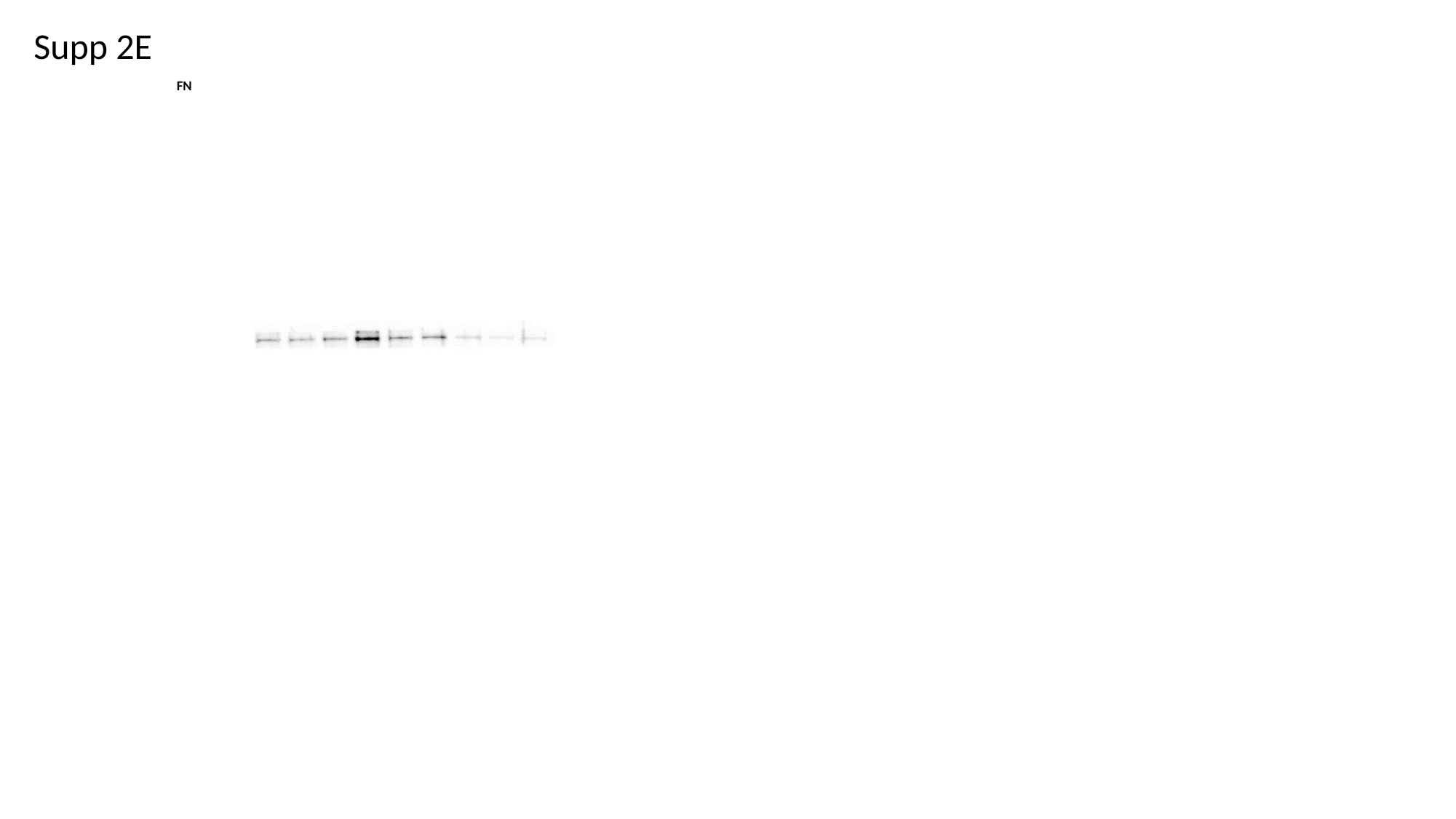

Supp 2E
FN

## Slide 57
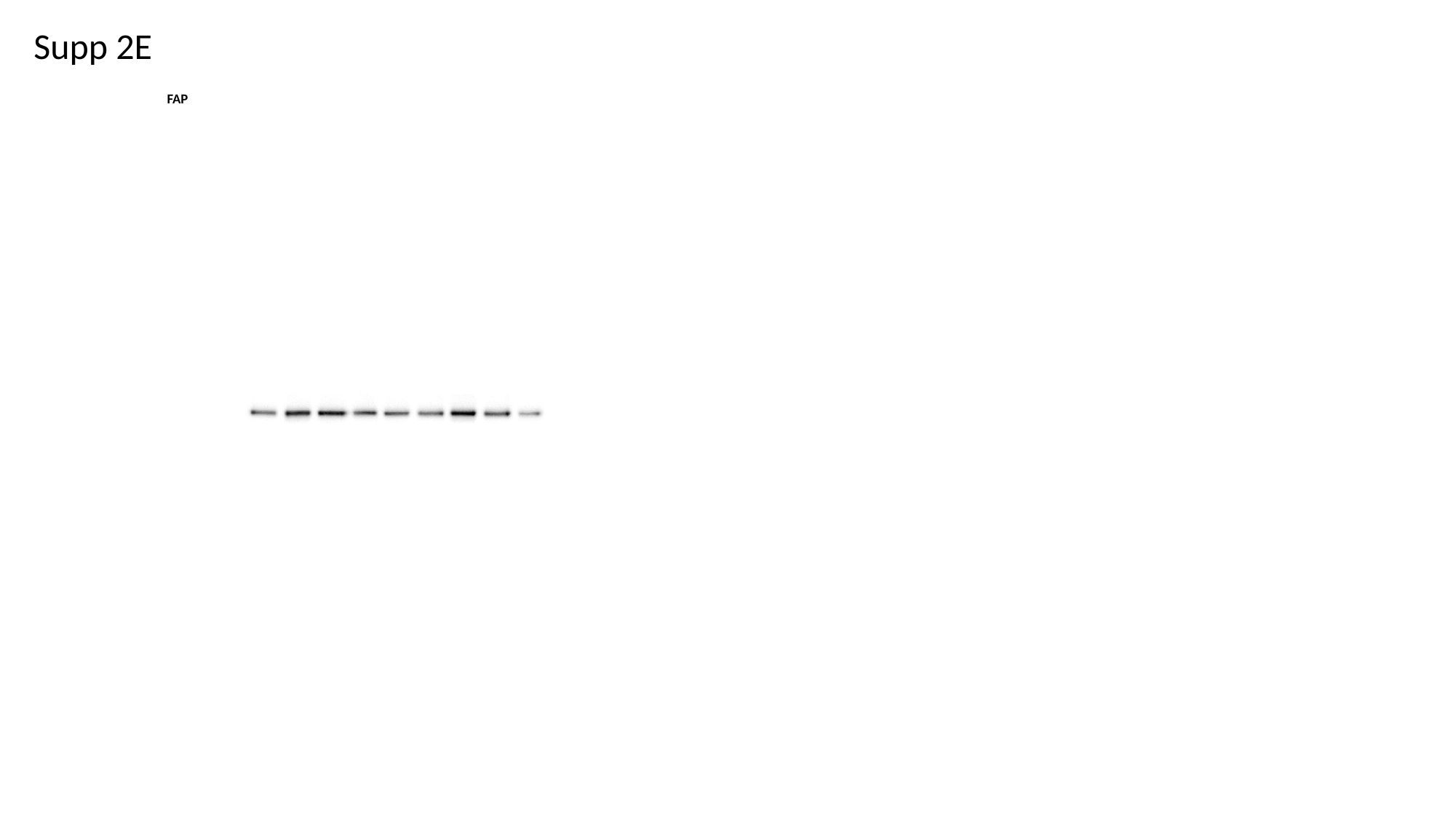

Supp 2E
FAP

## Slide 58
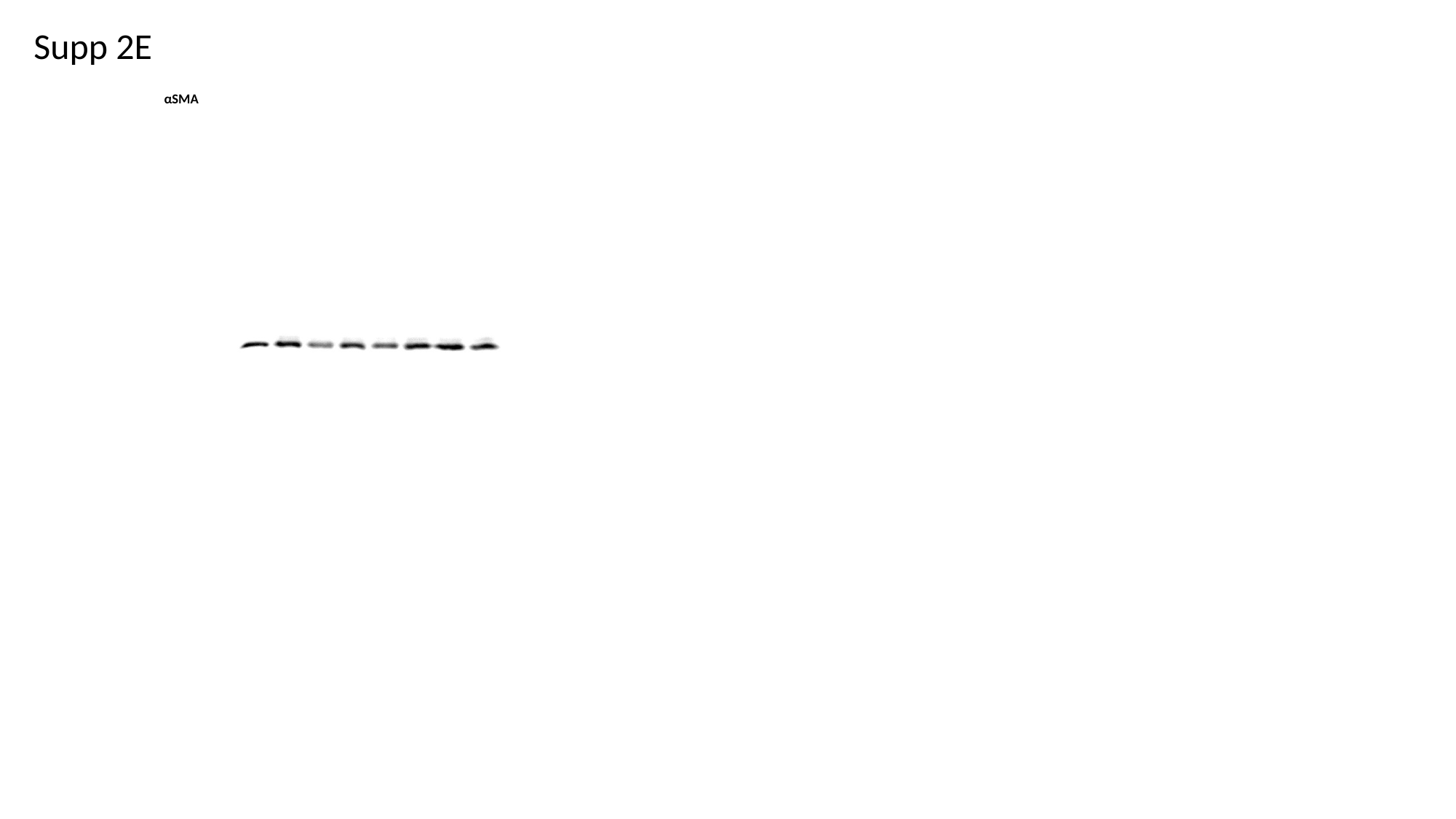

Supp 2E
αSMA

## Slide 59
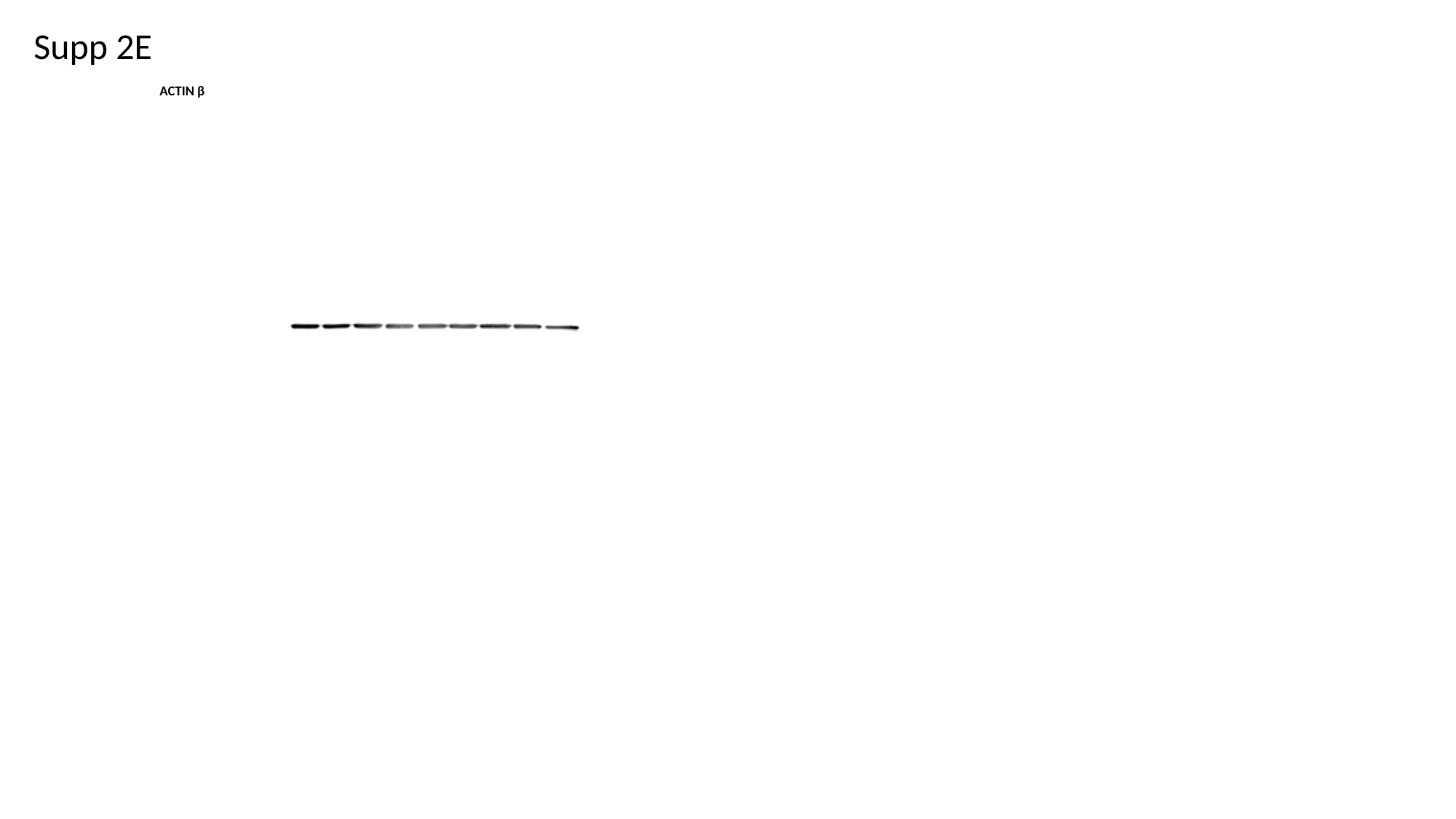

Supp 2E
ACTIN β

## Slide 60
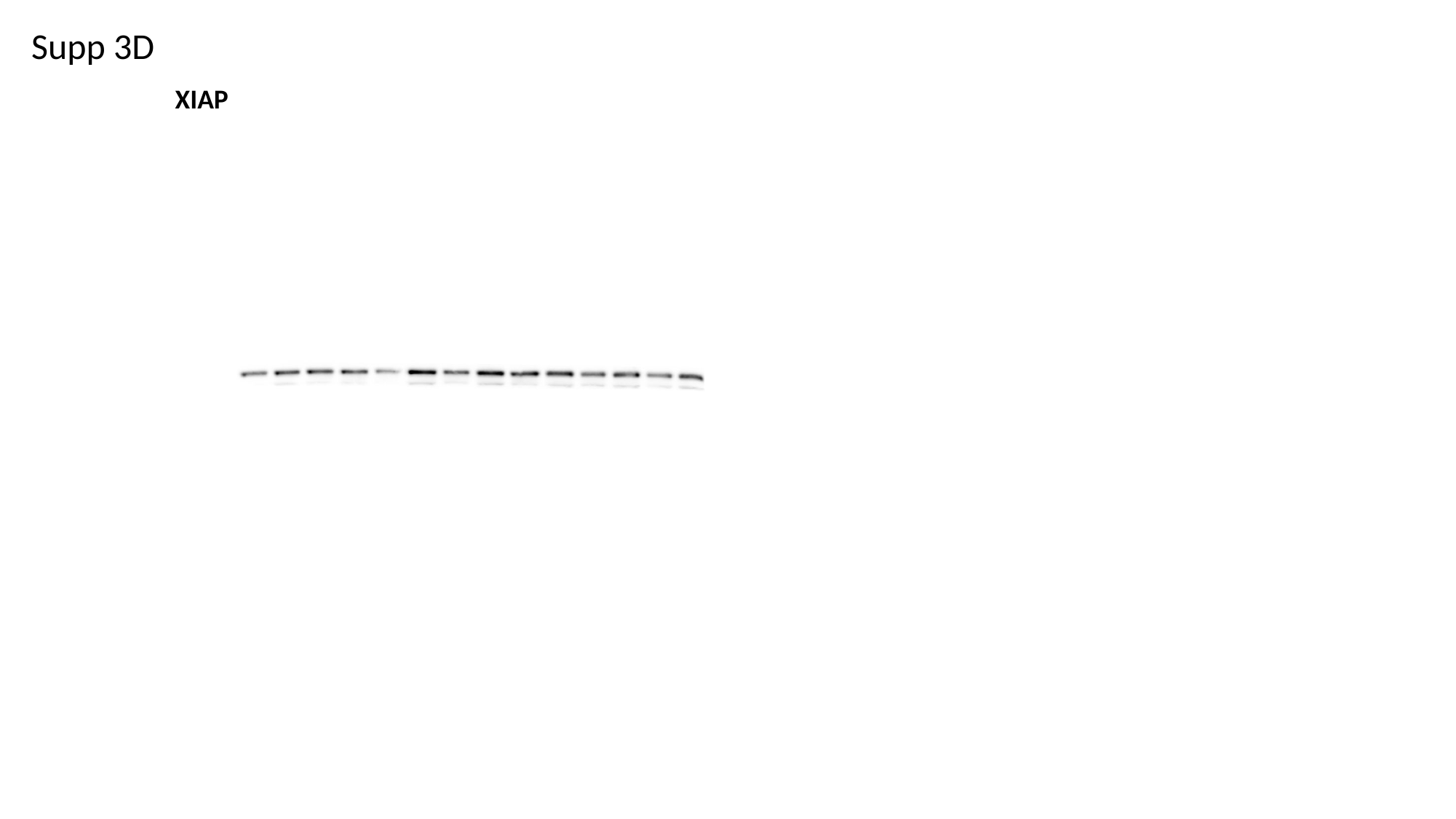

Supp 3D
XIAP

## Slide 61
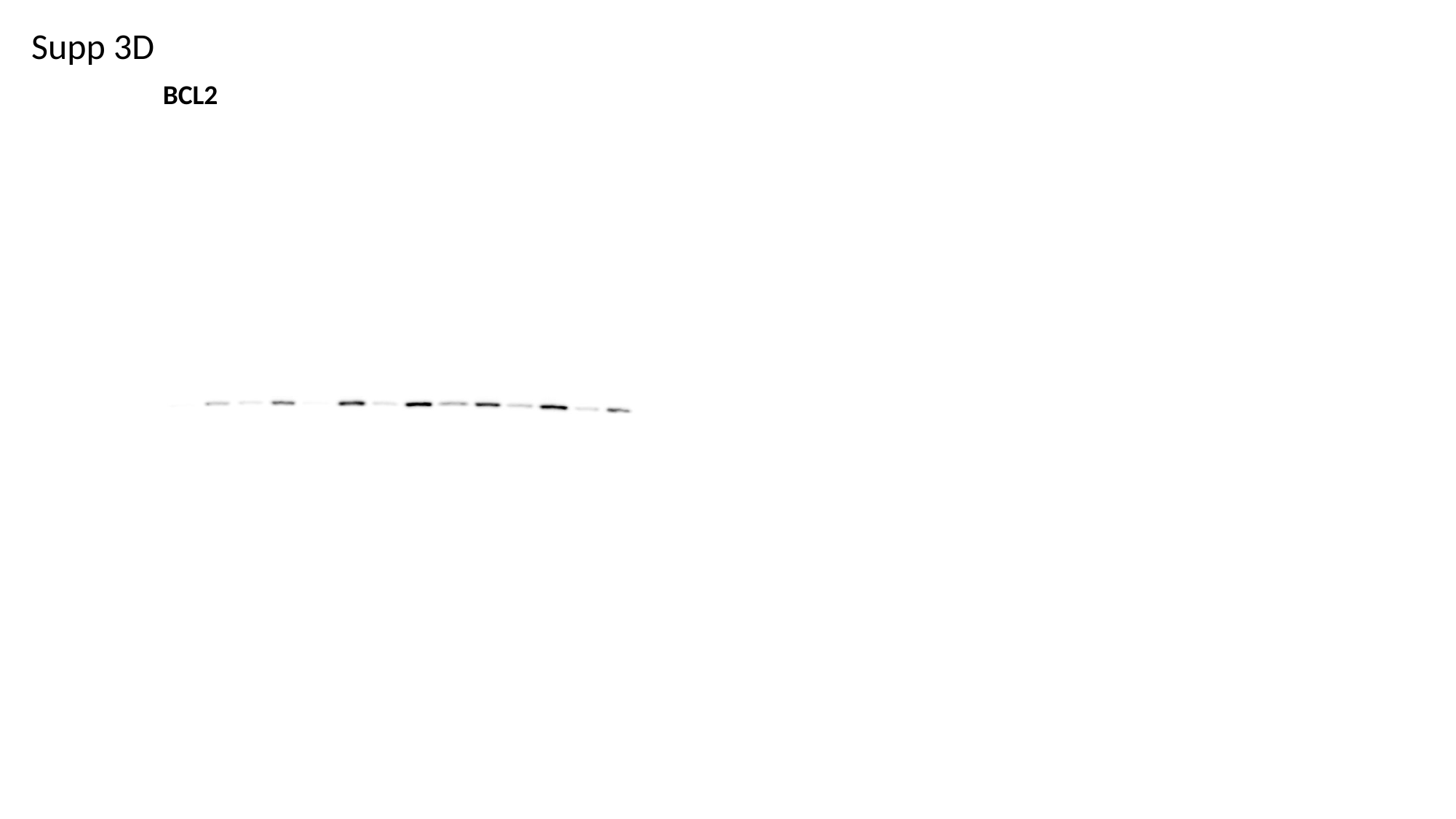

Supp 3D
BCL2

## Slide 62
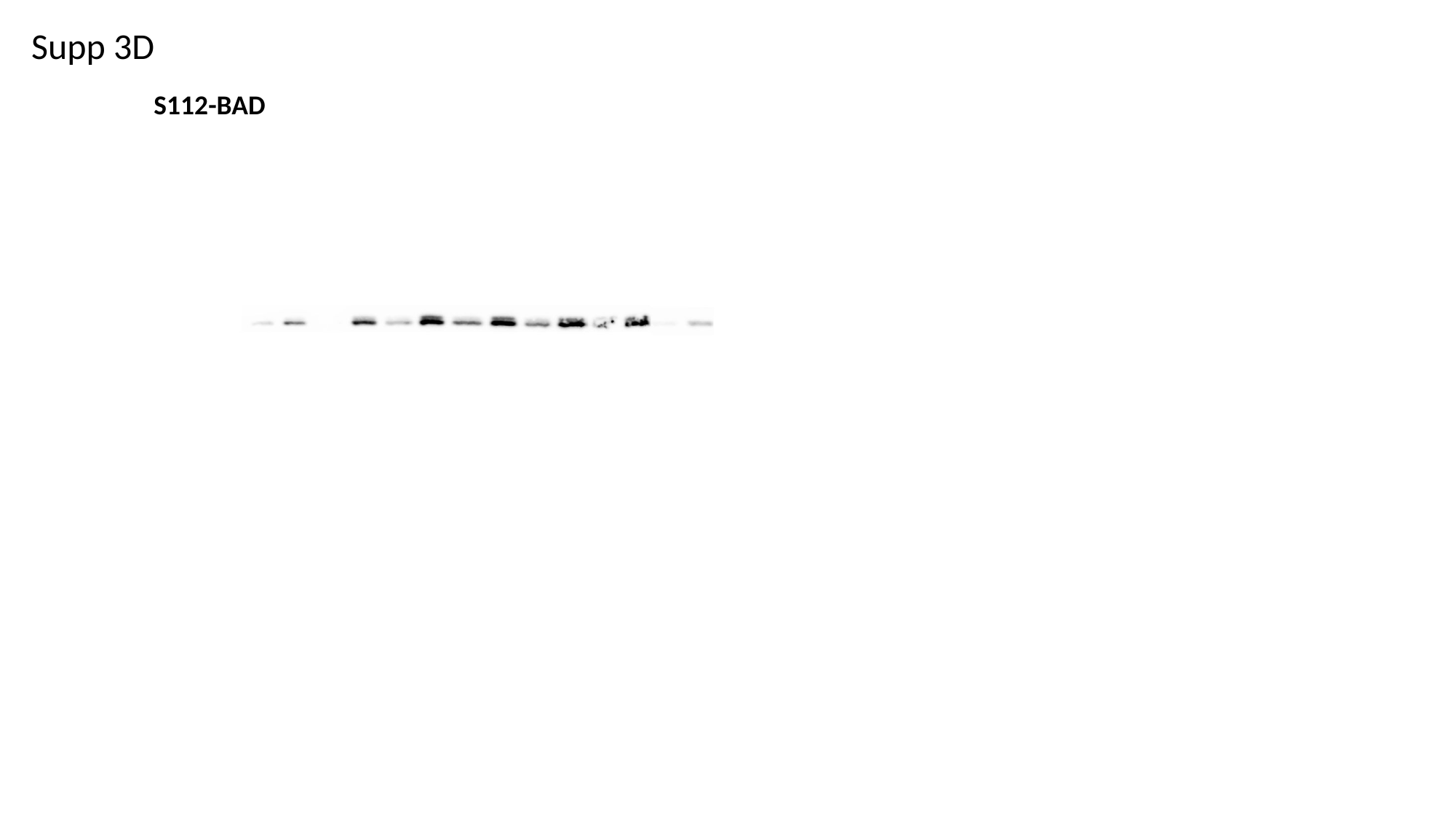

Supp 3D
S112-BAD

## Slide 63
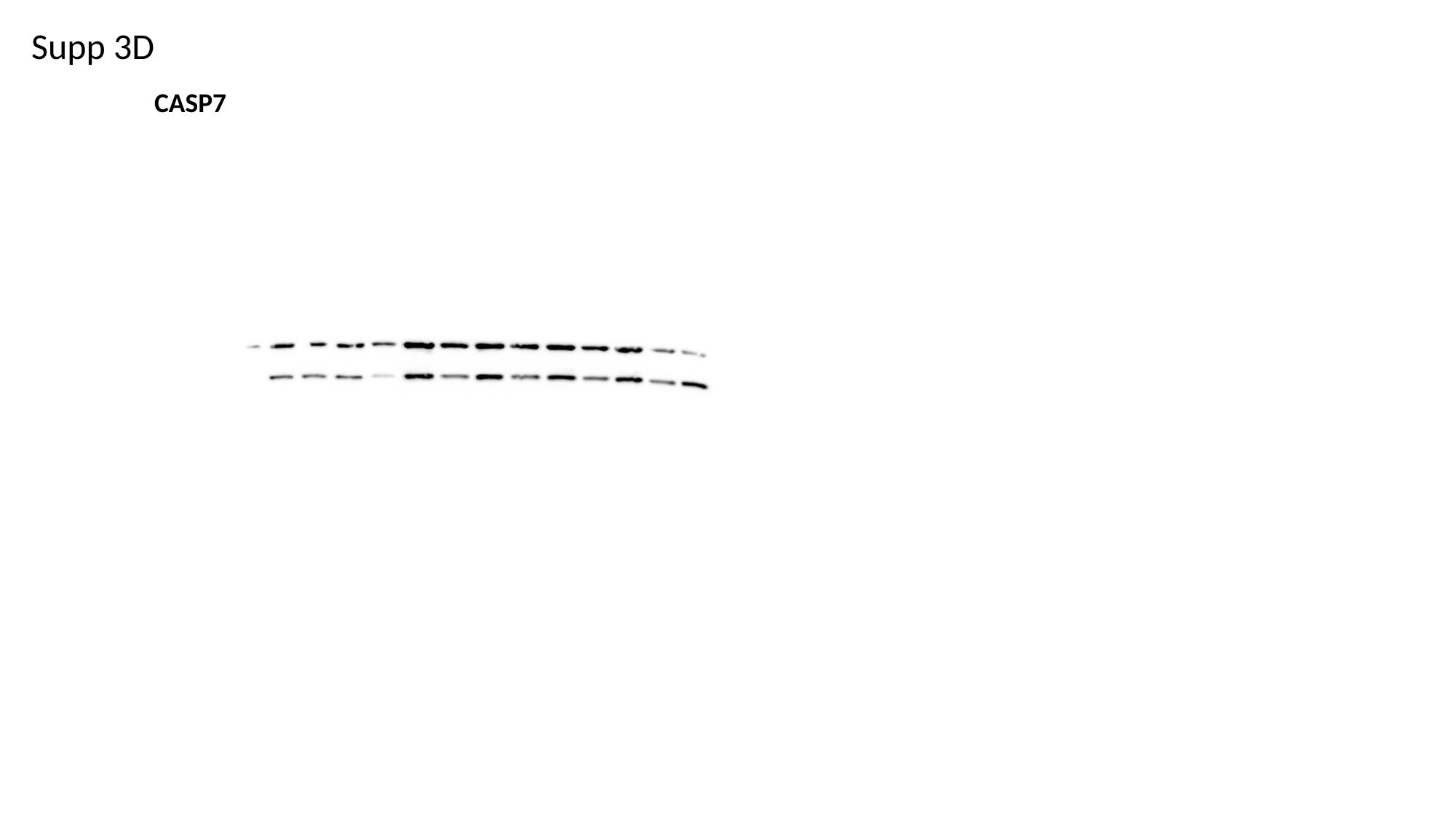

Supp 3D
CASP7

## Slide 64
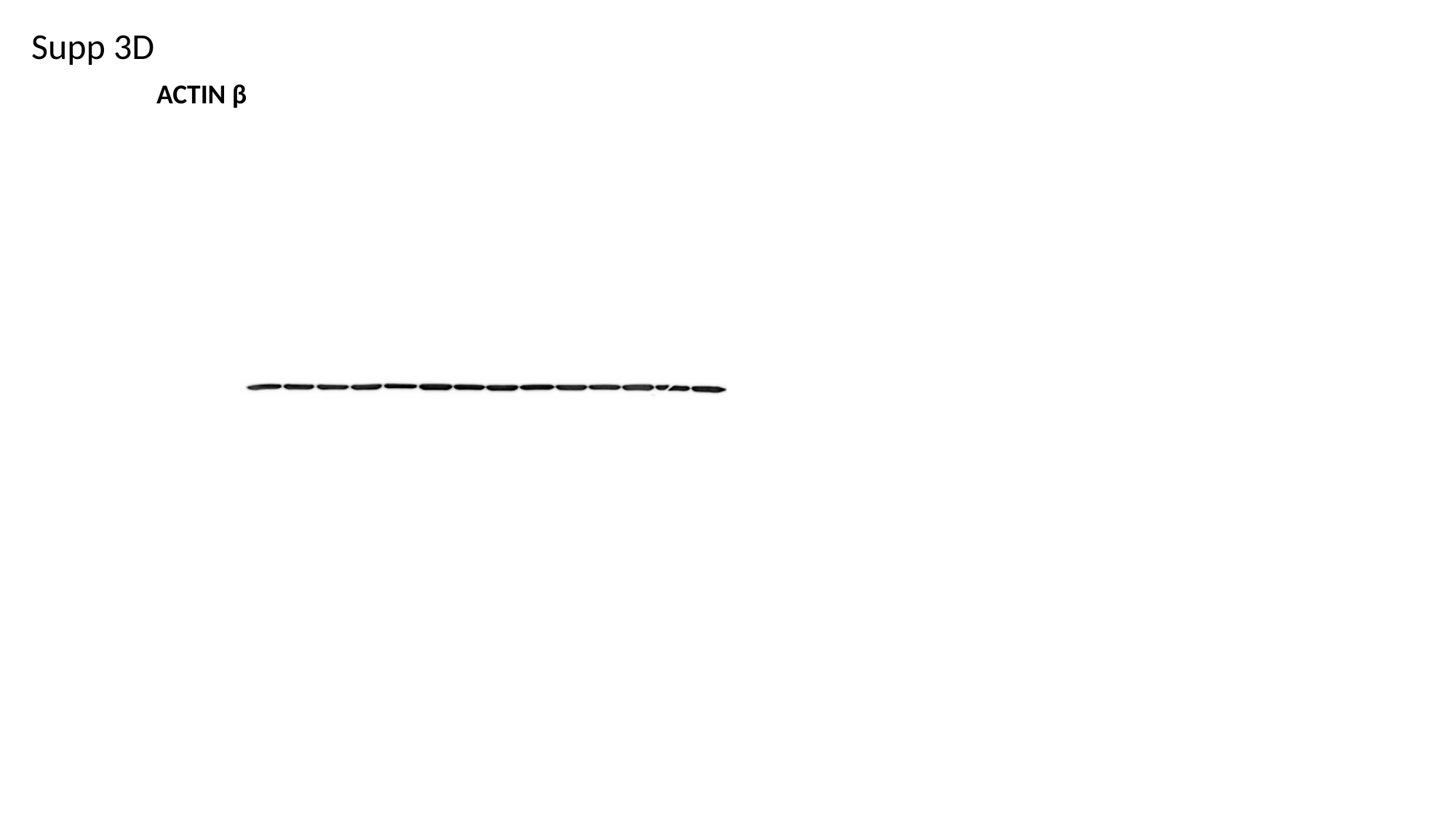

Supp 3D
ACTIN β

## Slide 65
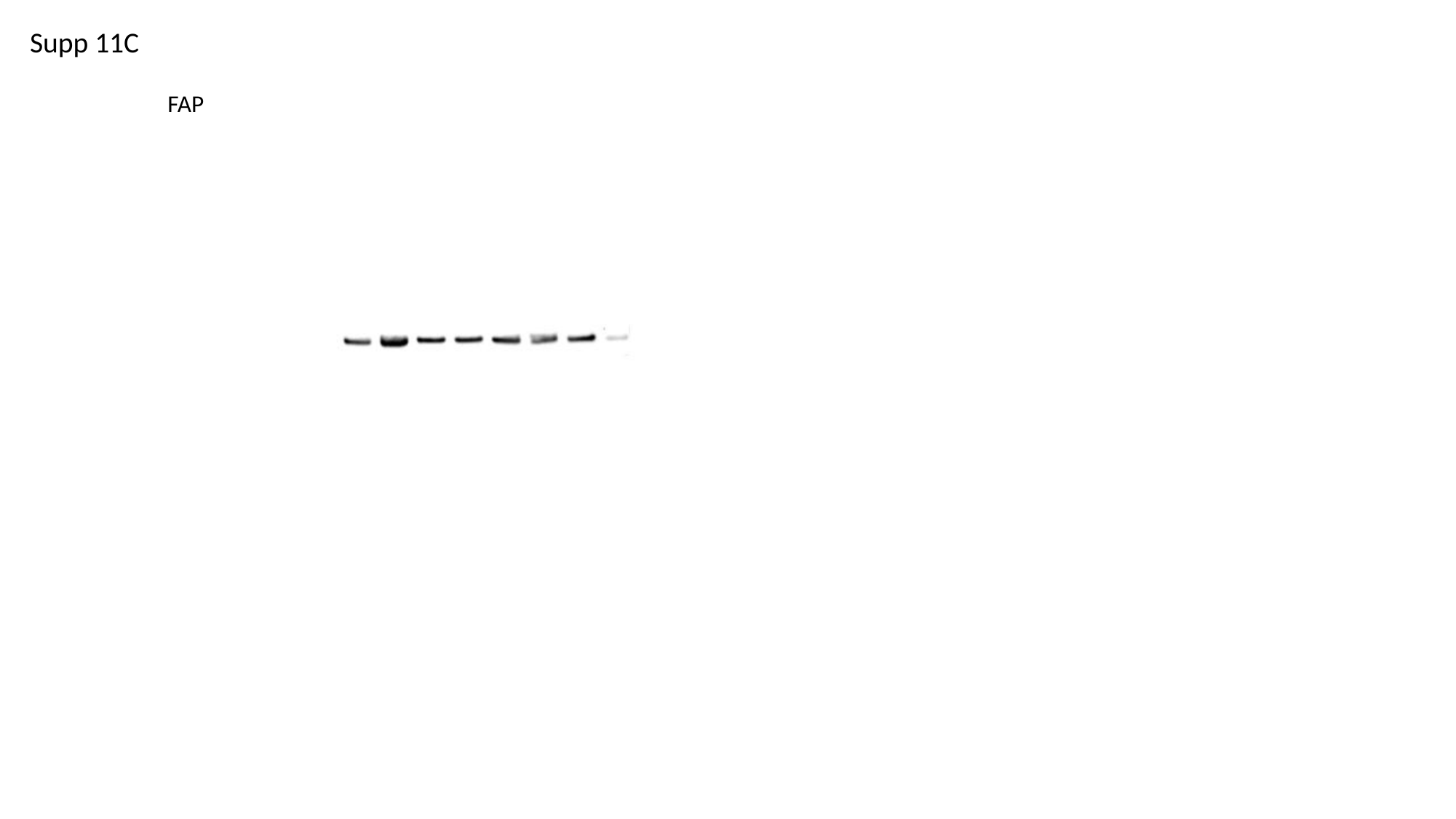

Supp 11C
FAP

## Slide 66
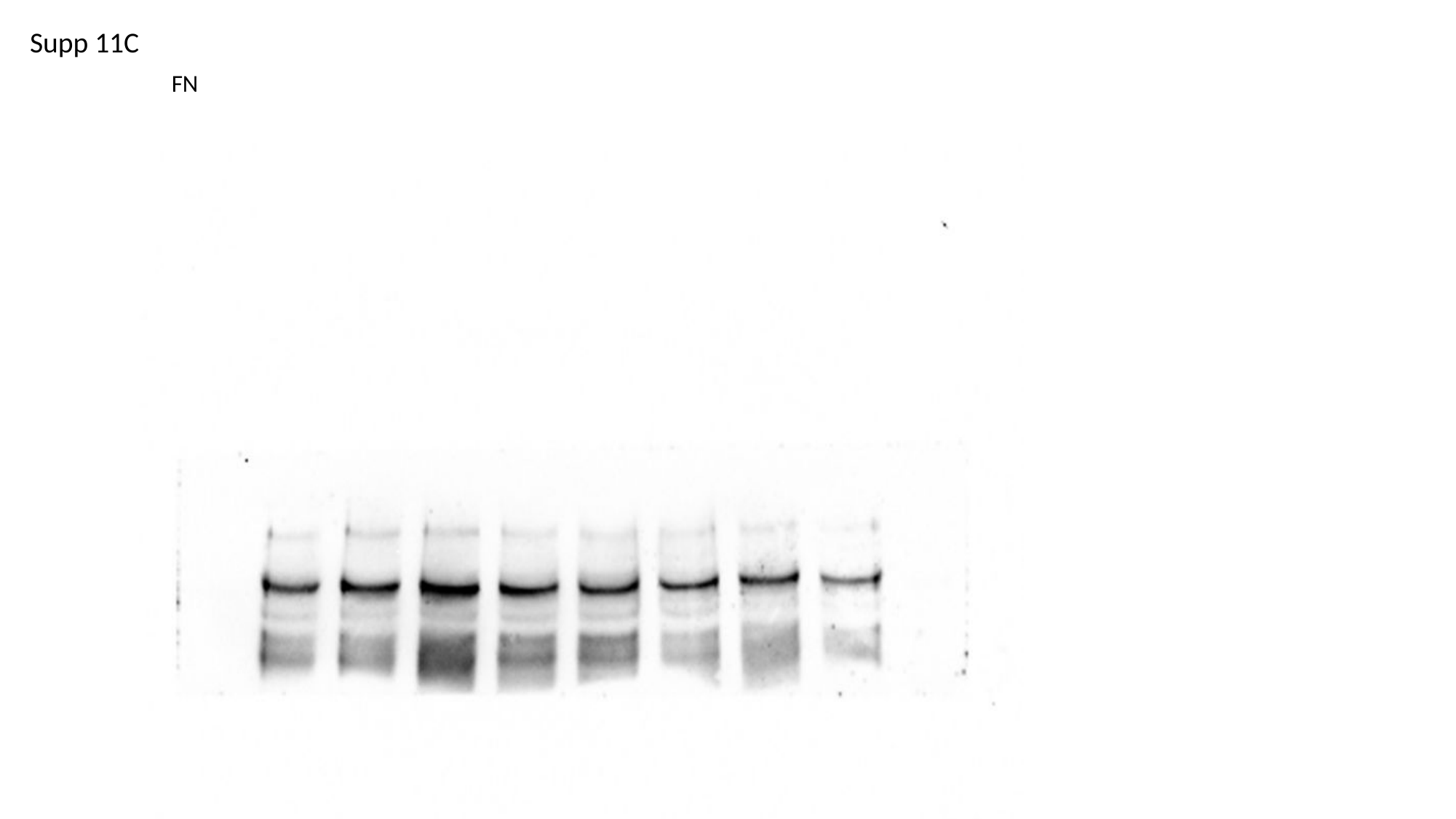

Supp 11C
FN

## Slide 67
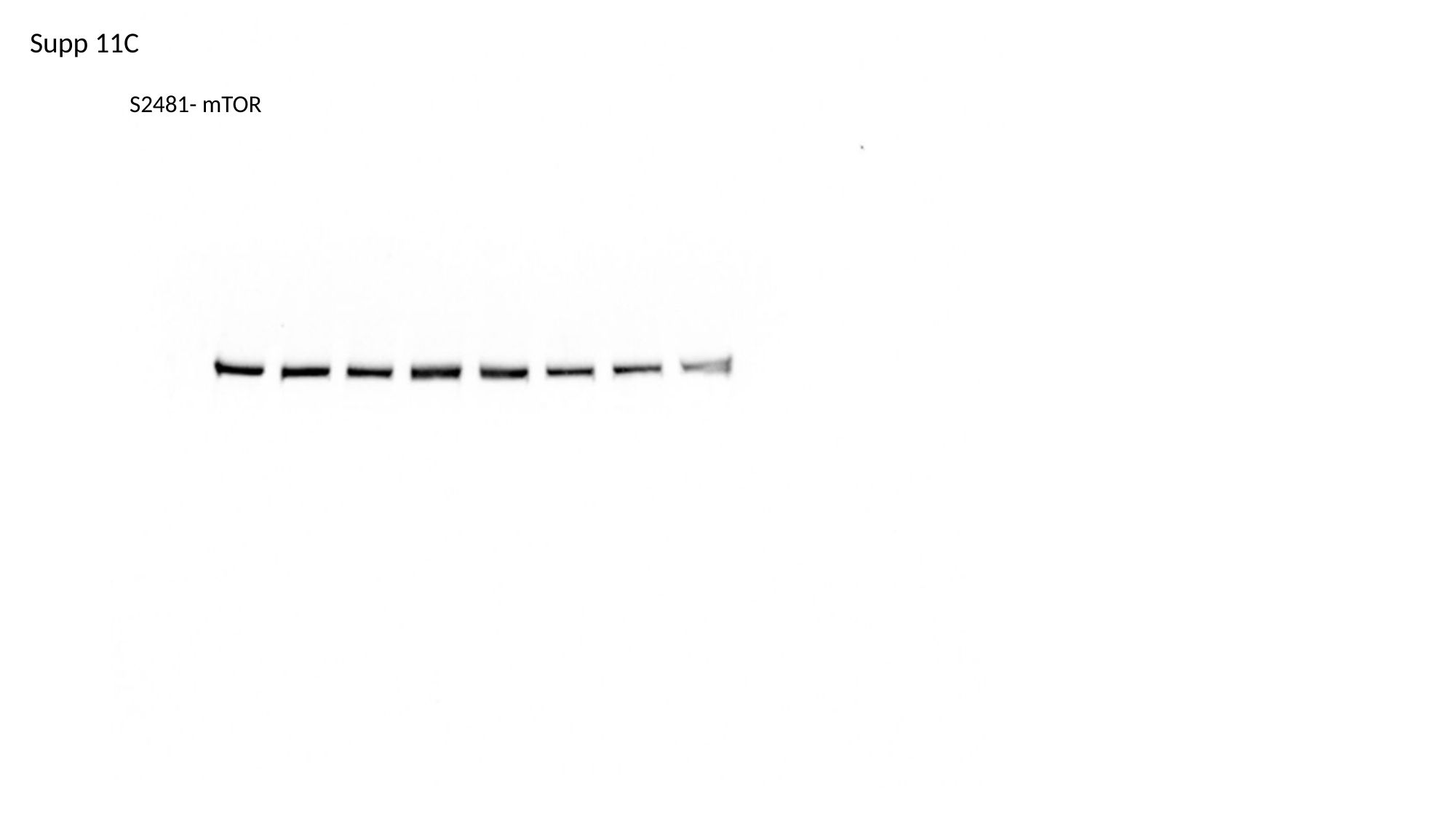

Supp 11C
S2481- mTOR

## Slide 68
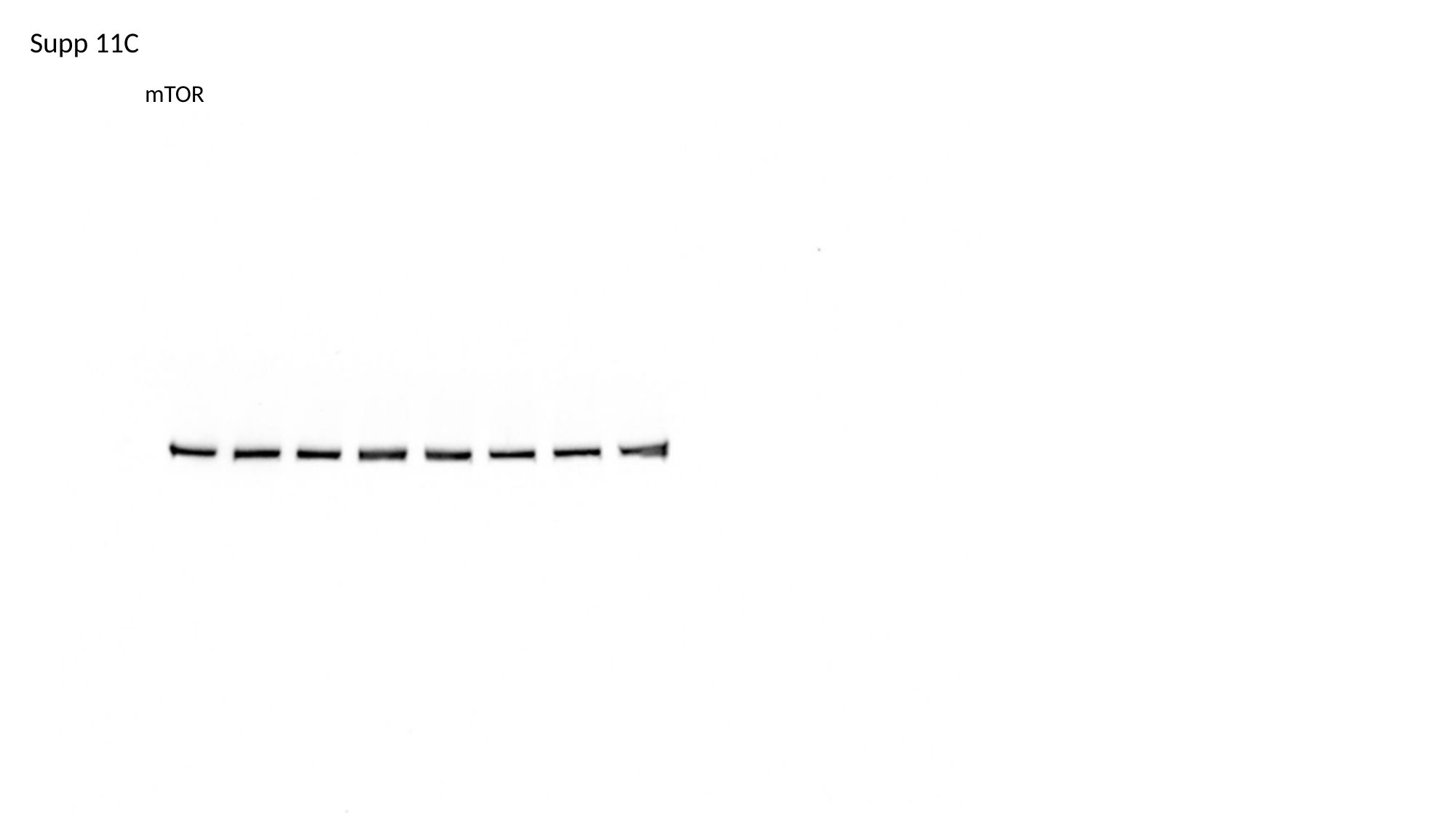

Supp 11C
mTOR

## Slide 69
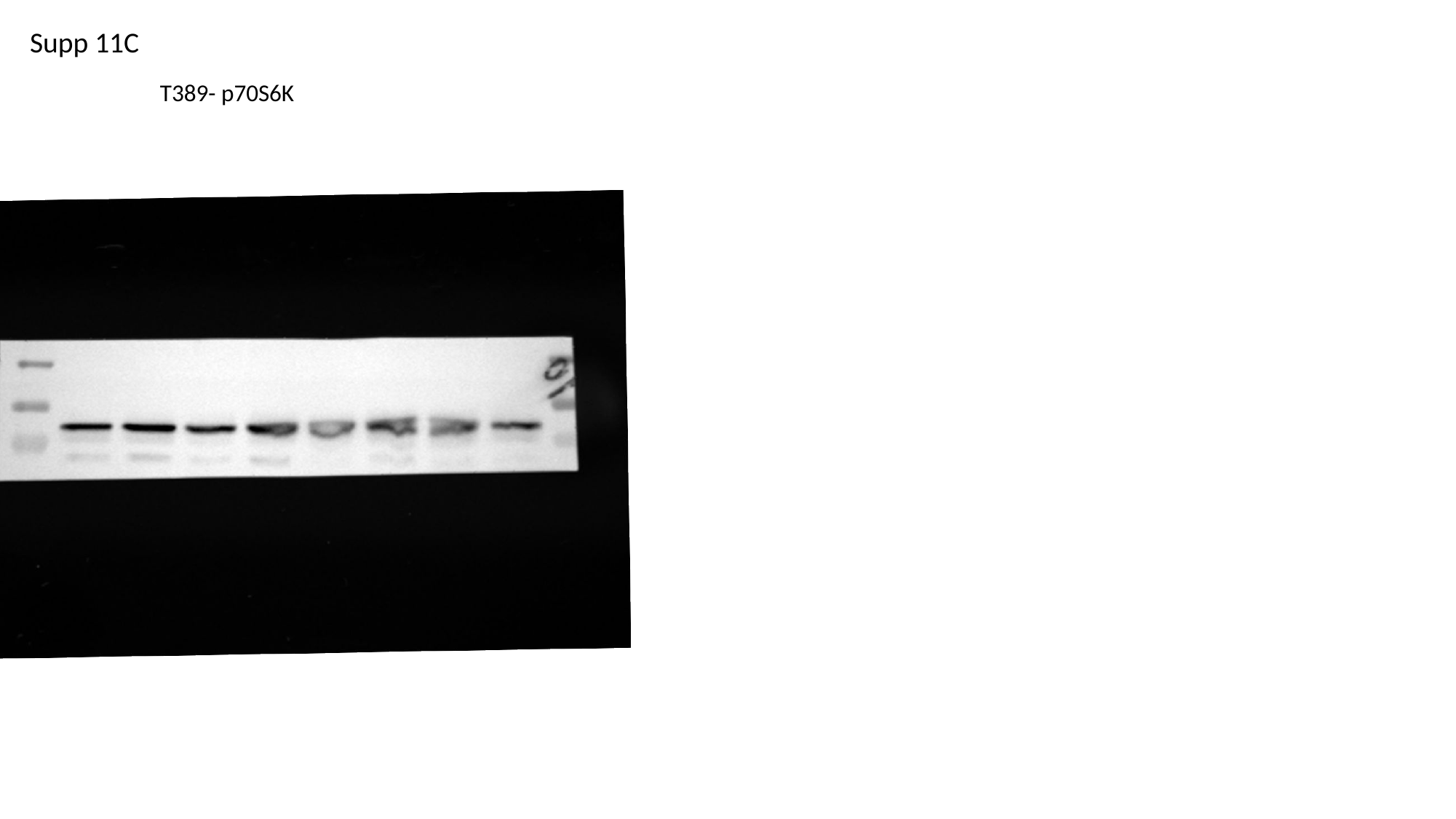

Supp 11C
T389- p70S6K

## Slide 70
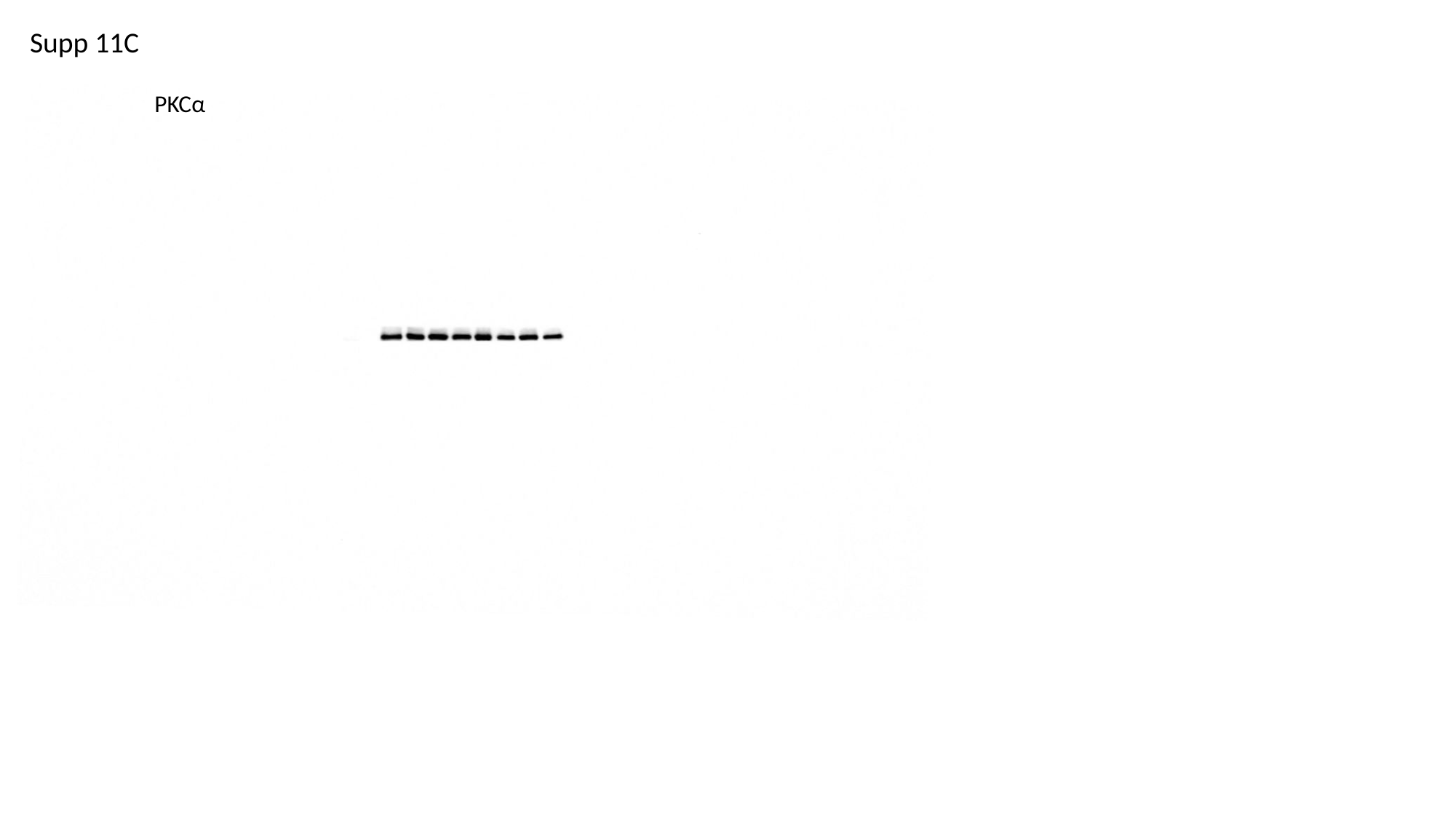

Supp 11C
PKCα

## Slide 71
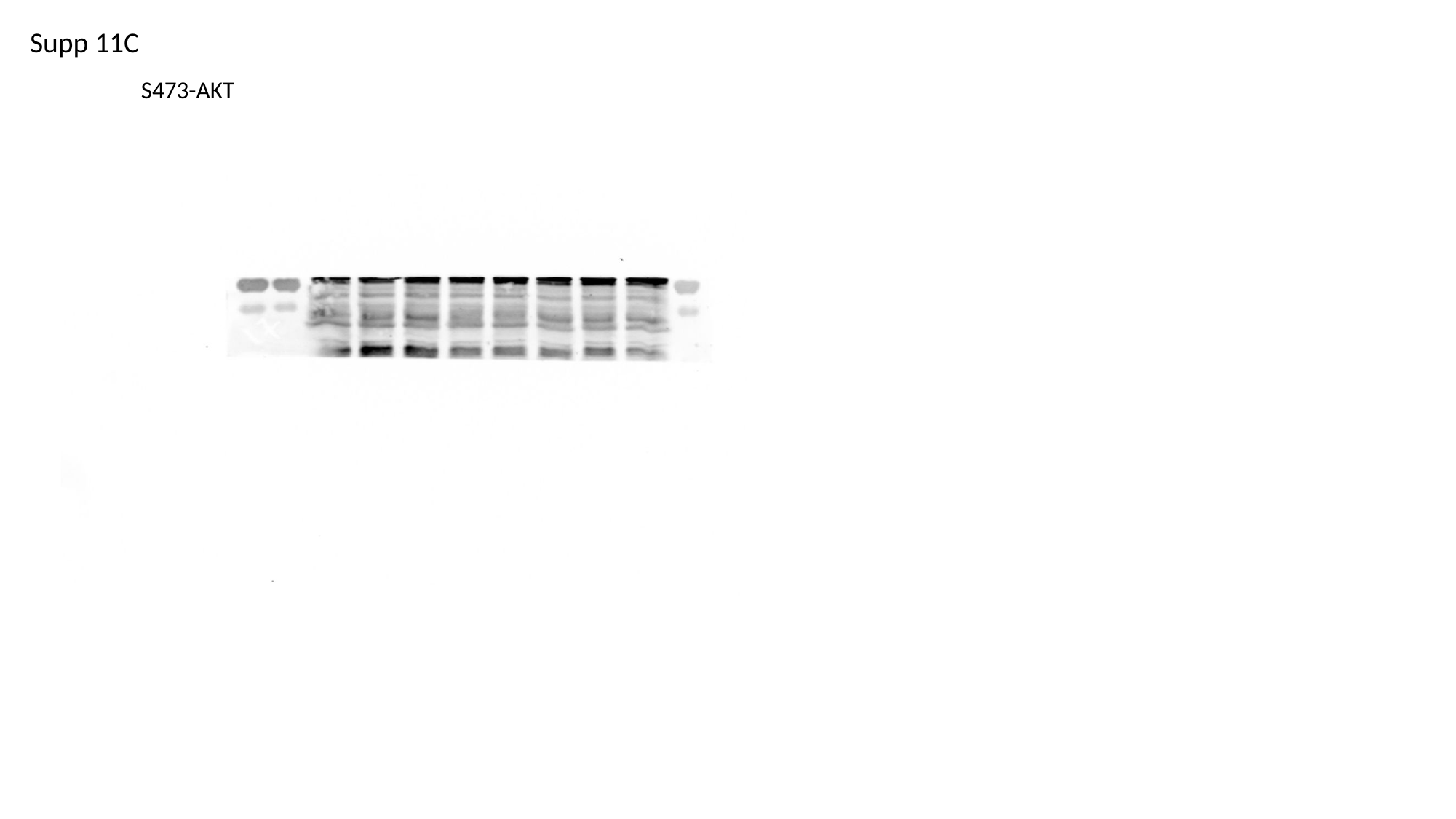

Supp 11C
S473-AKT

## Slide 72
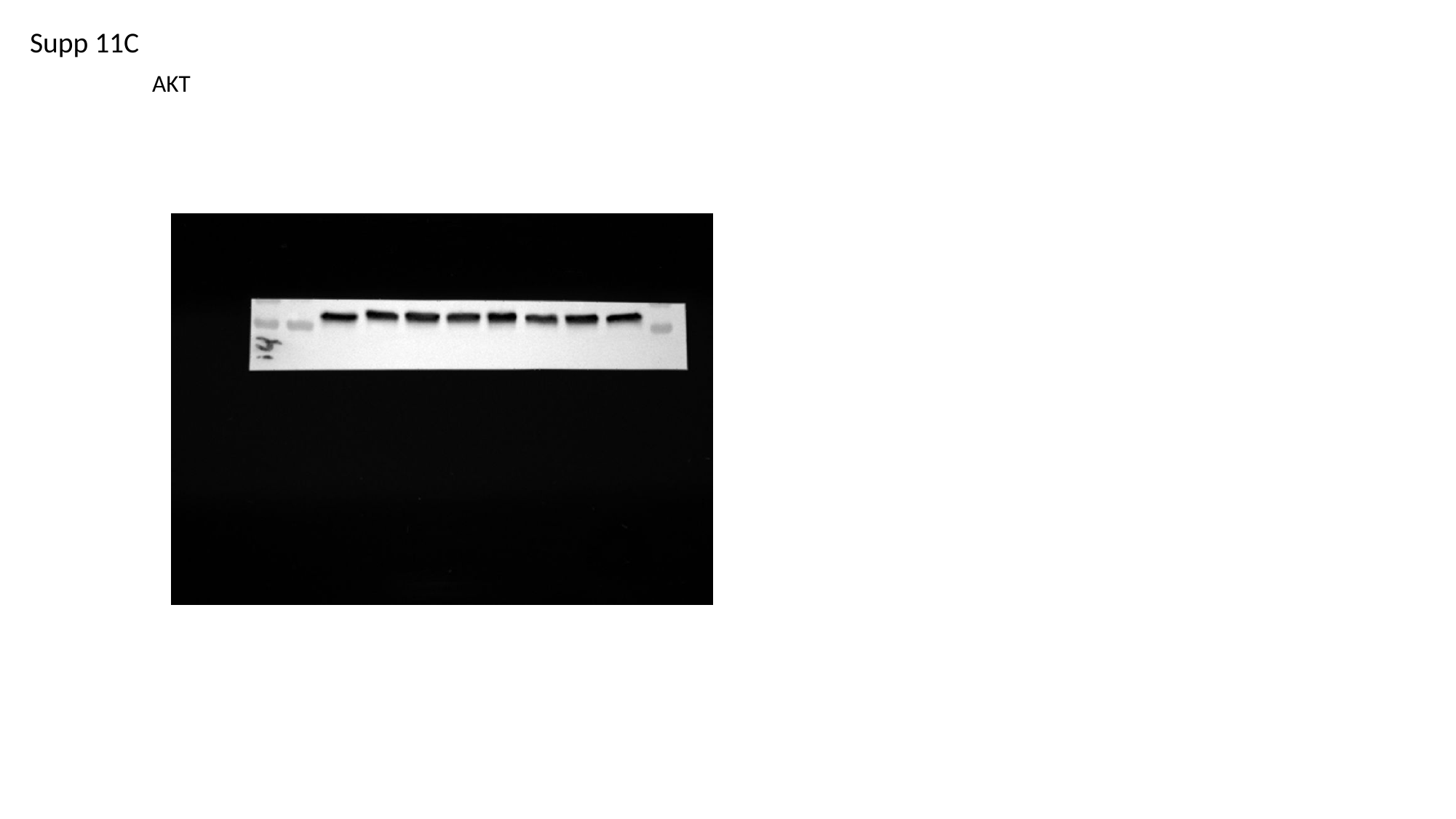

Supp 11C
AKT

## Slide 73
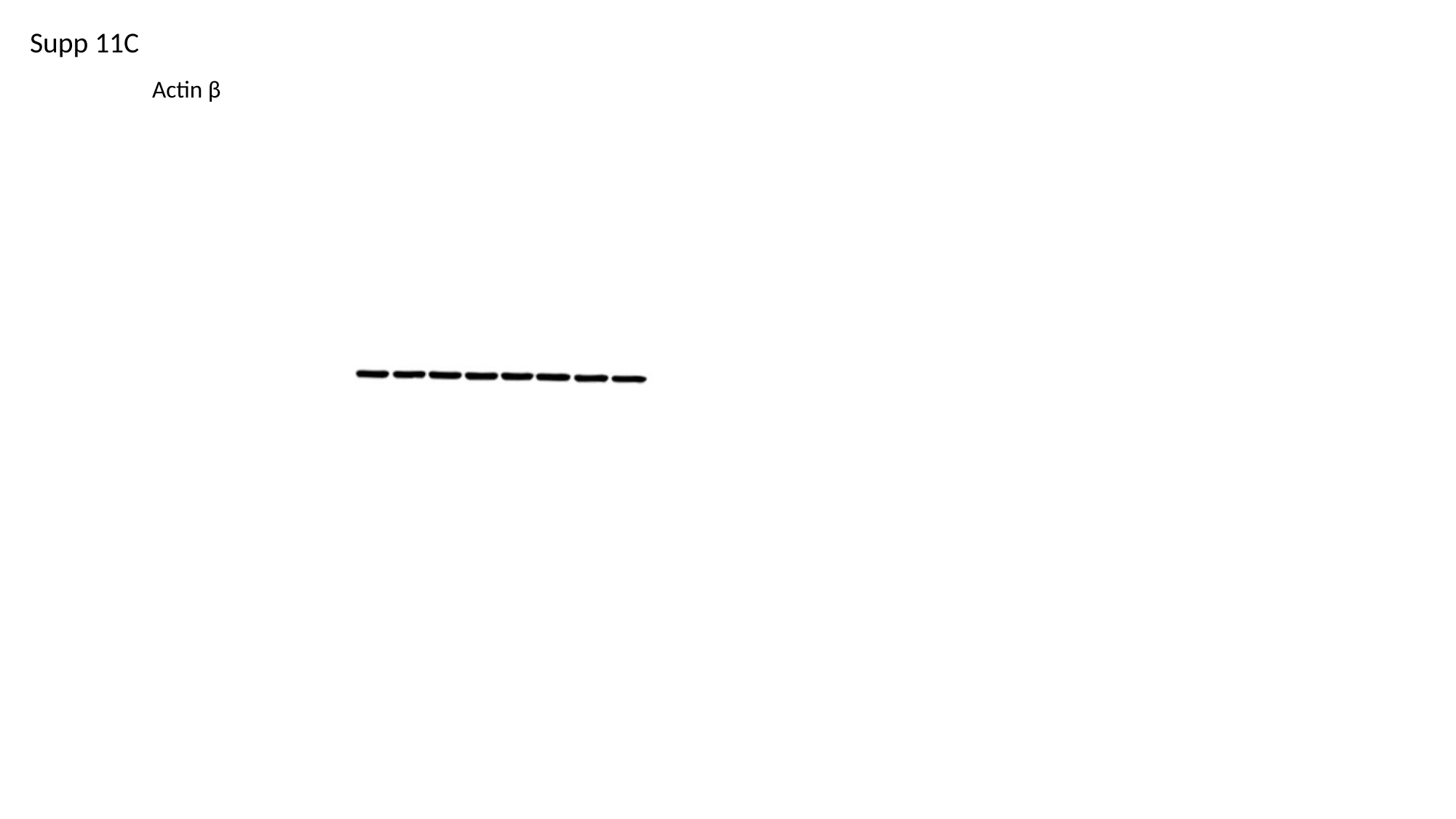

Supp 11C
Actin β

## Slide 74
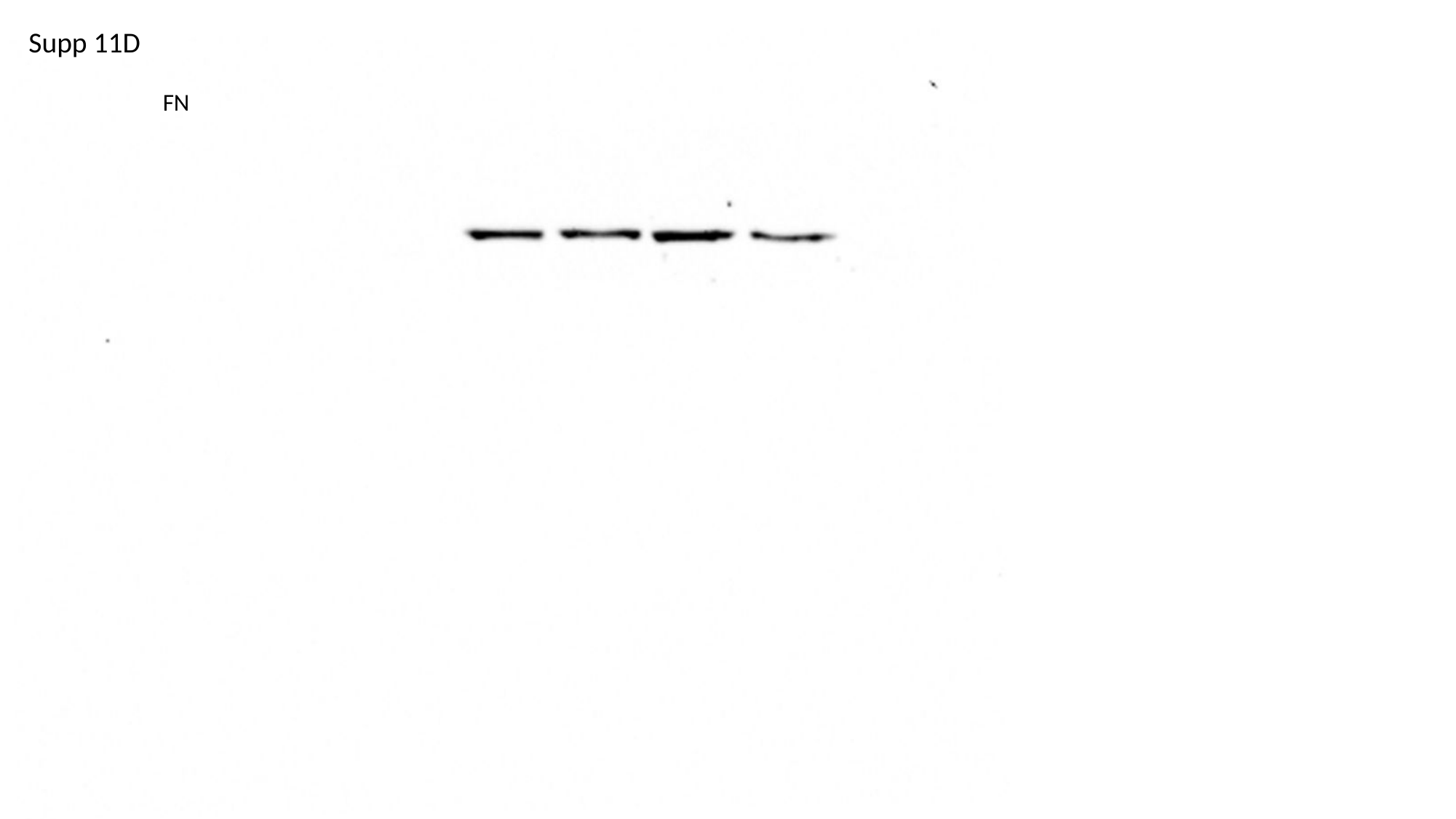

Supp 11D
FN

## Slide 75
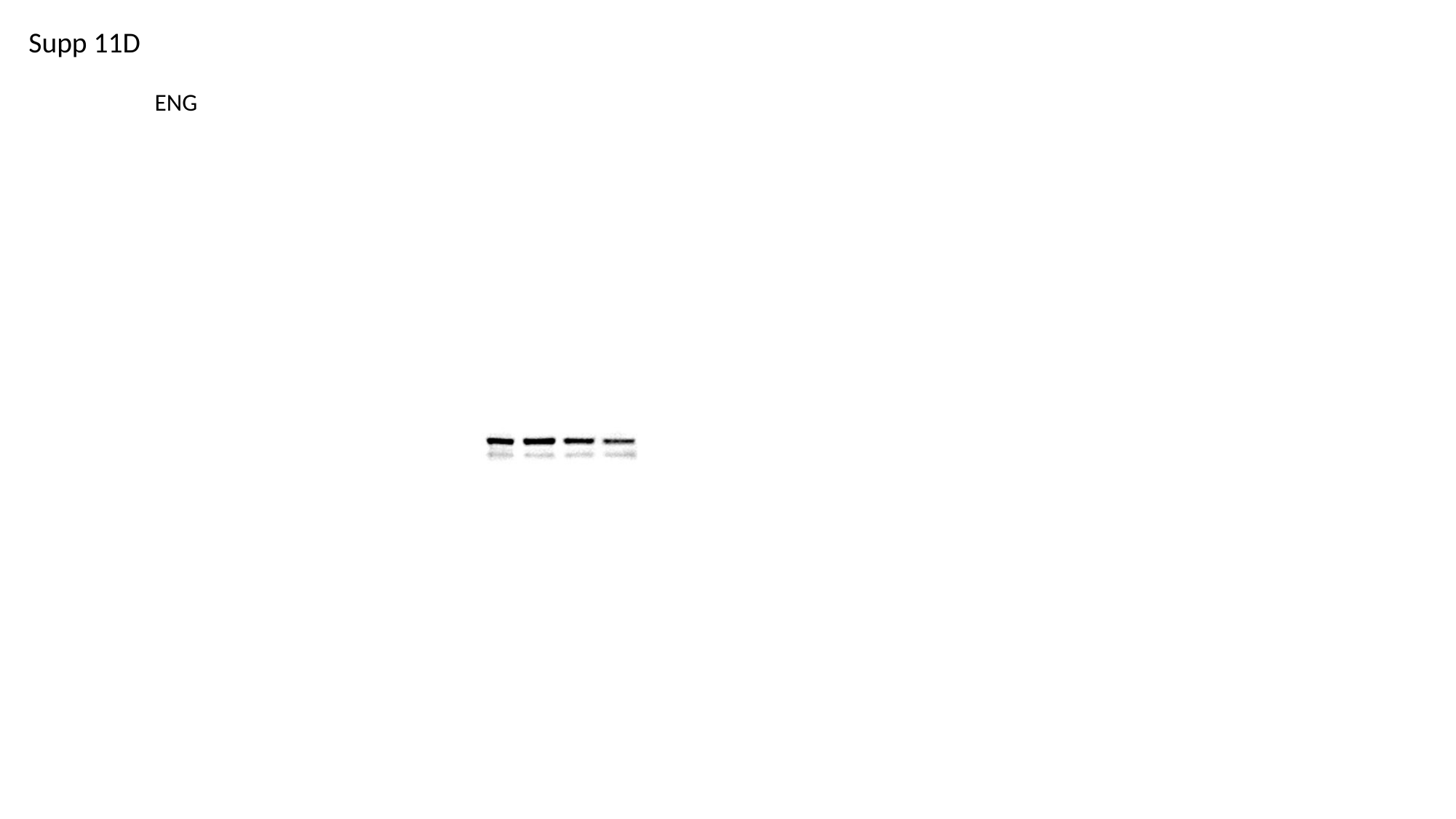

Supp 11D
ENG

## Slide 76
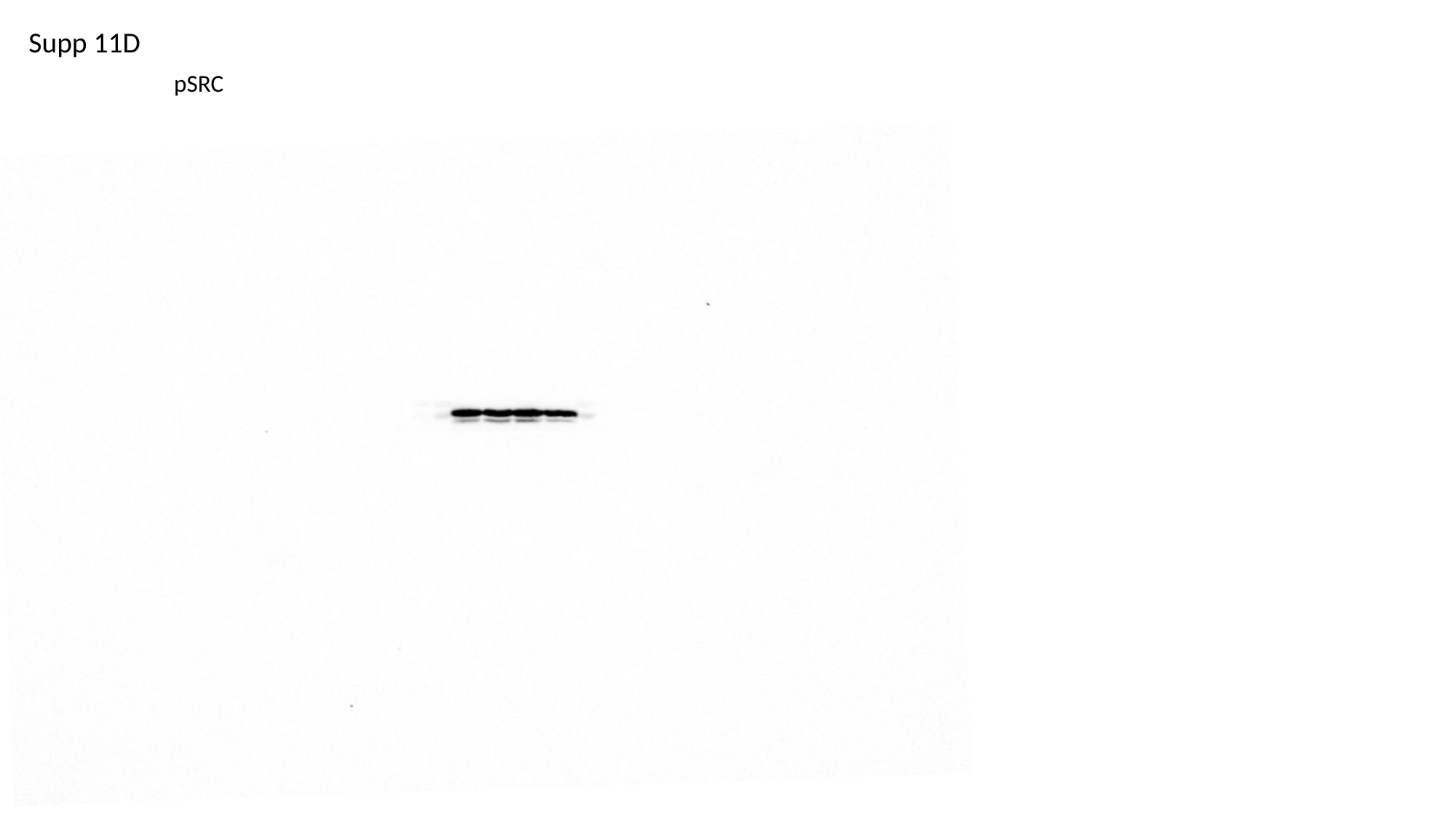

Supp 11D
pSRC

## Slide 77
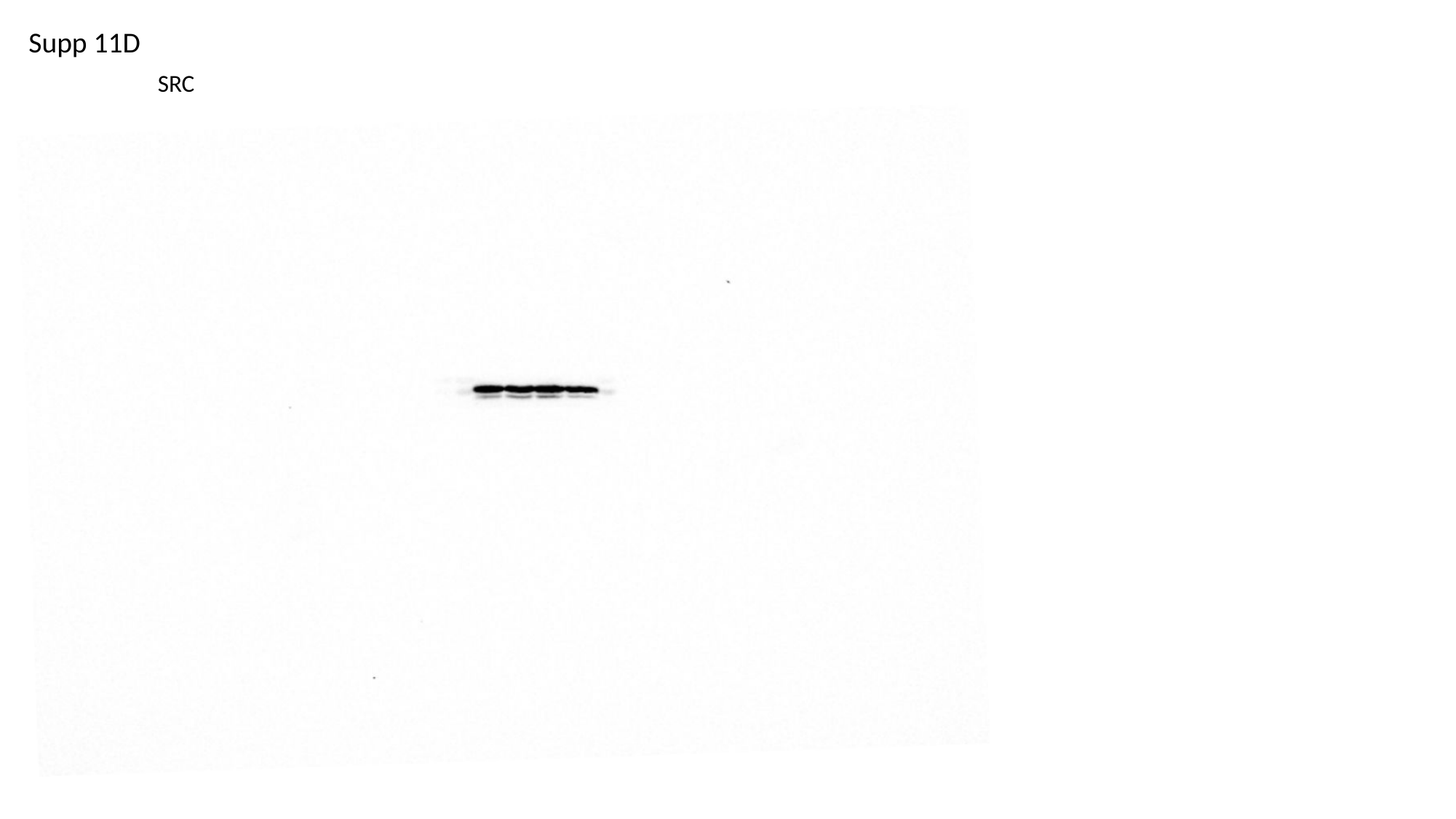

Supp 11D
SRC

## Slide 78
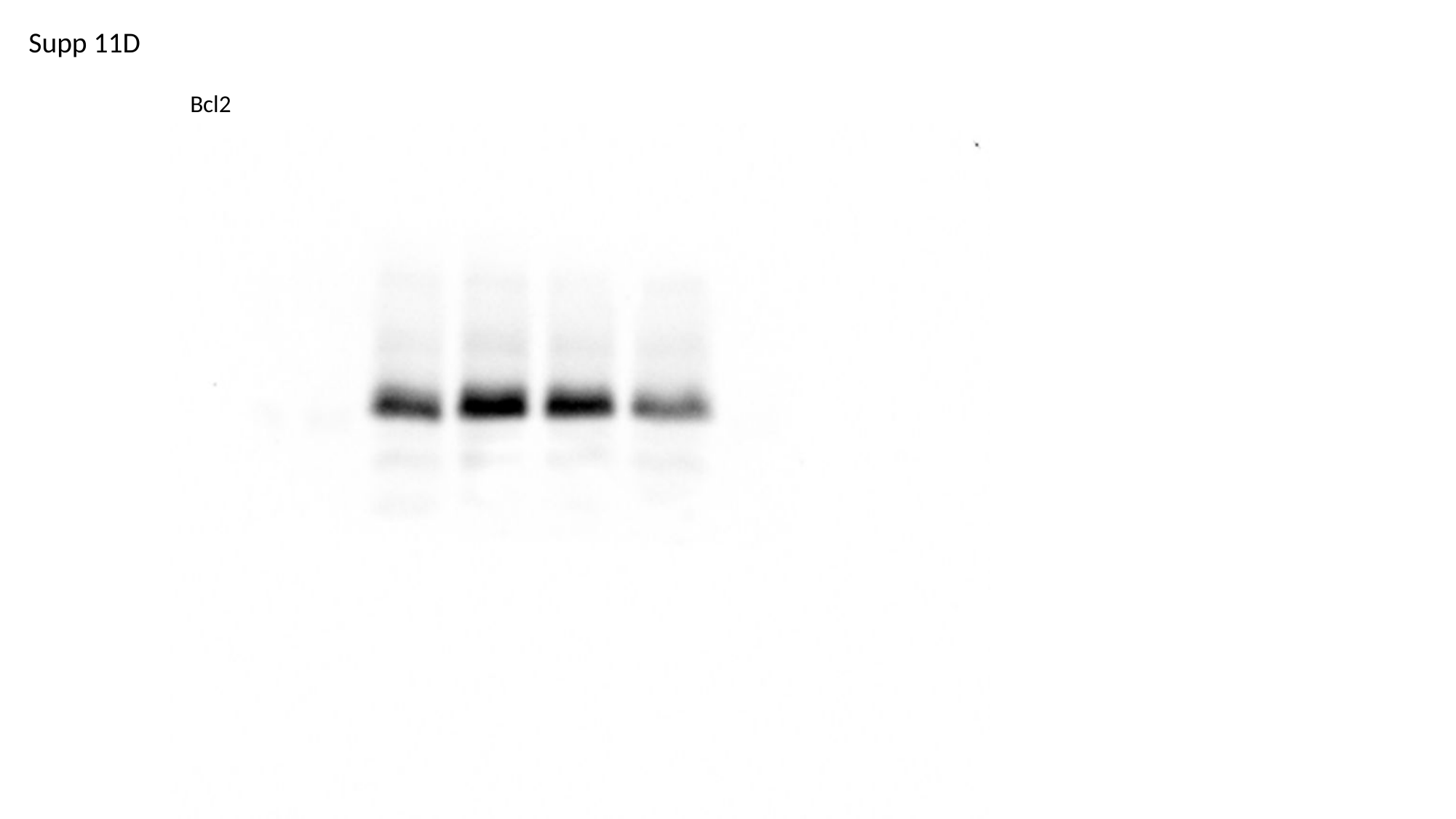

Supp 11D
Bcl2

## Slide 79
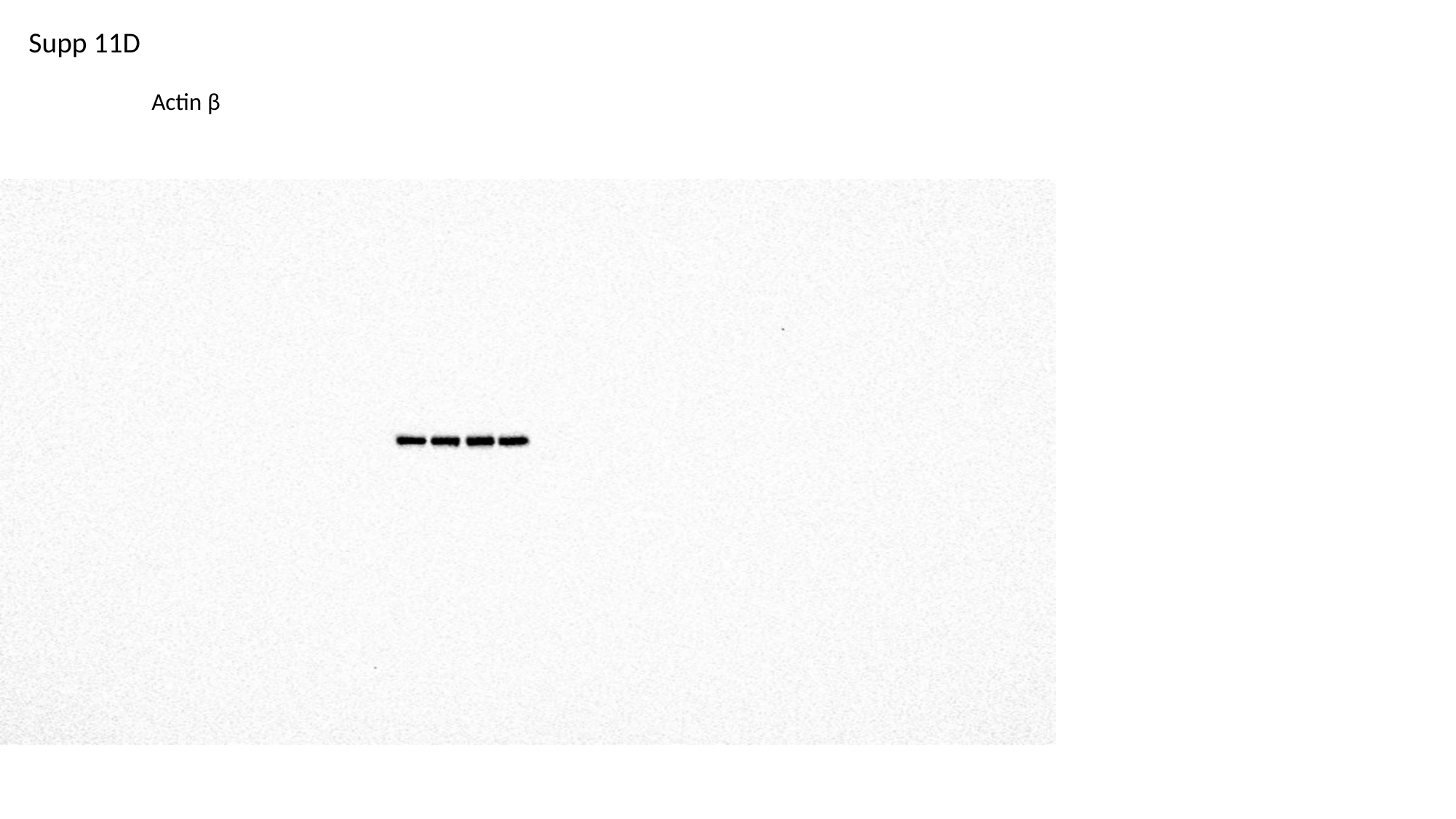

Supp 11D
Actin β
